# Recruitment of hexahydroquinoline as anticancer scaffold targeting inhibition of wild and mutants EGFR (EGFR^WT^, EGFR^T790M^, and EGFR^L858R^)

**DOI:** 10.1080/14756366.2023.2241674

**Published:** 2023-08-07

**Authors:** Mahmoud G. Abo Al-Hamd, Haytham O. Tawfik, Omeima Abdullah, Koki Yamaguchi, Masaharu Sugiura, Ahmed B. M. Mehany, Mervat H. El-Hamamsy, Tarek F. El-Moselhy

**Affiliations:** a Department of Pharmaceutical Chemistry, Faculty of Pharmacy, Tanta University, Tanta, Egypt; b Pharmaceutical Chemistry Department, College of Pharmacy, Umm Al-Qura University, Makkah, Saudi Arabia; c Faculty of Pharmaceutical Sciences, Sojo University, Kumamoto, Japan; d Zoology Department, Faculty of Science, Al-Azhar University, Cairo, Egypt

**Keywords:** Hexahydroquinoline, wild and mutant EGFR inhibition, anticancer, X-ray crystallography, molecular docking

## Abstract

Hexahydroquinoline (HHQ) scaffold was constructed and recruited for development of new series of anticancer agents. Thirty-two new compounds were synthesised where x-ray crystallography was performed to confirm enantiomerism. Thirteen compounds showed moderate to good activity against NCI 60 cancer cell lines, with GI % mean up to 74% for **10c**. Expending erlotinib as a reference drug, target compounds were verified for their inhibiting activities against EGFR^WT^, EGFR^T790M^, and EGFR^L858R^ where compound **10d** was the best inhibitor with IC_50_ = 0.097, 0.280, and 0.051 µM, respectively, compared to erlotinib (IC_50_ = 0.082 µM, 0.342 µM, and 0.055 µM, respectively). Safety profile was validated using normal human lung (IMR-90) cells. **10c** and **10d** disrupted cell cycle at pre-G1 and G2/M phases in lung cancer, HOP-92, and cell line. Molecular docking study was achieved to understand the potential binding interactions and affinities in the active sites of three versions of EGFRs.

## HIGHLIGHTS

- New 32 hexahydroquinoline (HHQ) analogues **6a–i**, **8a–m**, **10a–d**, and **12a–f** having the same features of EGFR inhibitors were synthesised in racemic mixtures.
- The antiproliferative activities were assessed towards 60 cancer cell lines which were efficiently inhibited by compound **10c**.
- Compound **10d** remarkably inhibited EGFR^WT^, EGFR^T790M^, and EGFR^L858R^.
- Cell cycle analysis and Annexin V-based flow cytometry in the HOP-92 lung cancer cells were performed.
- The safety profile of compounds **10c** and **10d** was validated using normal human lung (IMR-90) cells.
- Molecular docking studies revealed that the *S*-isomers exhibited higher affinity than *R*-isomers to active sites.

## Graphical abstract

**Figure:**
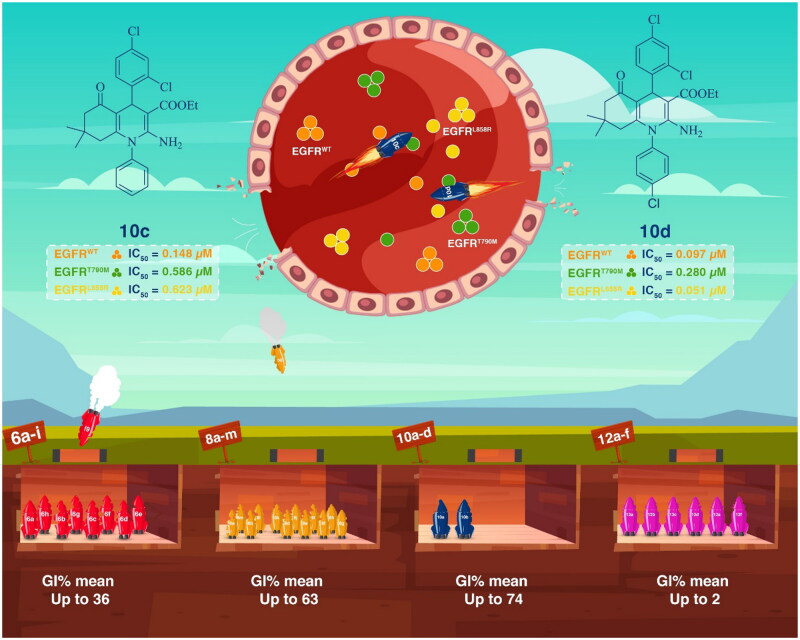

## Introduction

Despite massive attempts to develop novel anticancer agents, cancer remains one of the worst diseases in the world^1^. The development of resistance to several highly successful anticancer agents is a significant hurdle in the treatment of cancer^2^. As a result, developing new anticancer drugs has gained great momentum in the sector to address the issue of resistance^3^^,^^4^. According to the GLOBOCAN 2020 cancer incidence and mortality estimates, there were 19.3 million new cancer and 10 million cancer deaths^5^. One of the current strategies to manage cancer overgrowth is inhibiting a *trans*-membrane glycoprotein called Epidermal growth factor receptor (EGFR) due to its essential role in intracellular signalling, morphogenesis, and differentiation^6–8^.

**Figure 1.**
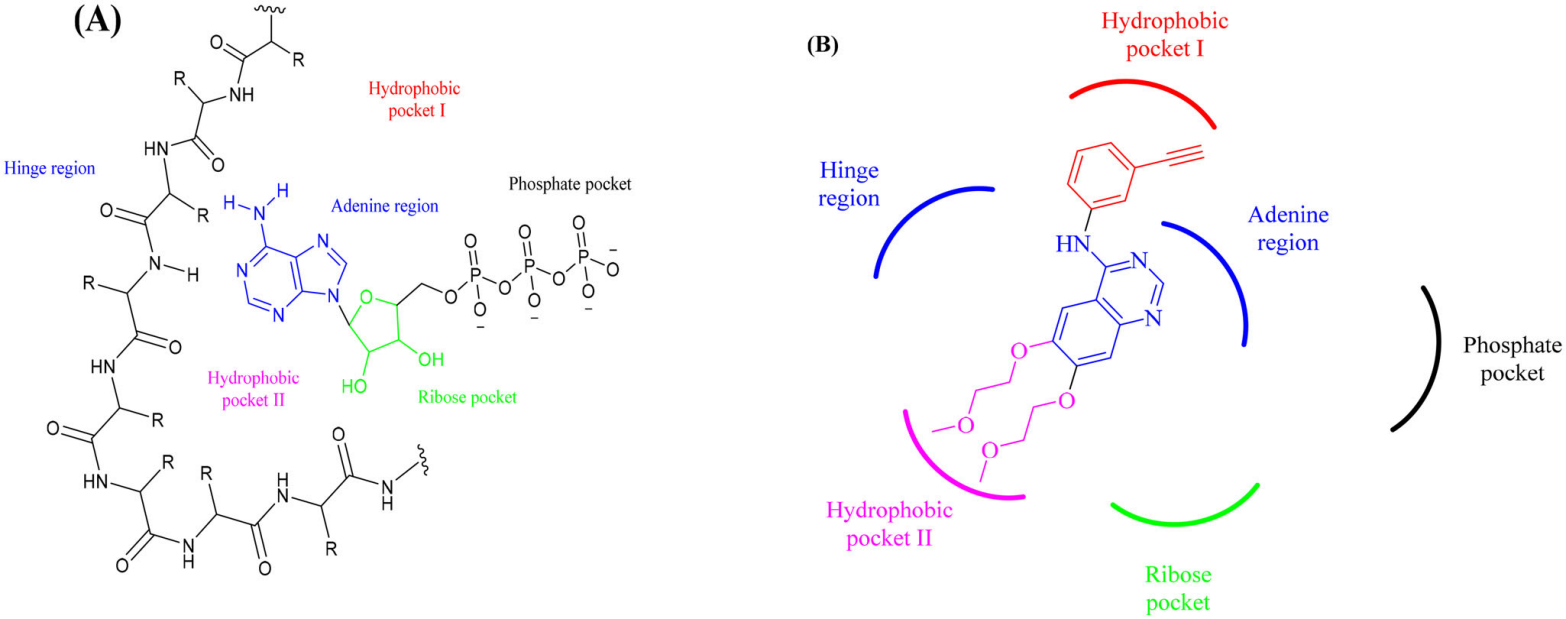
(A) ATP binding site at the EGFR viewing essential pharmacophore features. (B) The chemical structure of erlotinib (first-generation EGFR-TKI) could only reside in three pockets of the ATP binding sites.

Contrary to normal cells with tightly controlled EGFR pathways, tumour cells exhibit dysregulated EGFR signalling due to receptor overexpression and/or mutation^9^. This causes angiogenesis to rise and proliferation under unfavourable conditions, leading to the development of many cancers, such as NSC lung cancer^10^, breast cancer^11^, prostate cancer^12^, and colon cancer^13^. EGFR is a valid therapeutic target as a result^14^. There are three generations of EGFR tyrosine kinase inhibitors (TKIs), each developed to overcome a mutation to the previous one^15^. According to studies, one of the most frequent resistance mechanisms to first-generation EGFR-TKIs was the T790M “gatekeeper” mutation in EGFR, contributing to 3% of EGFR mutations^16^. A further known route of resistance to first-generation EGFR-TKIs is the L858R mutation, therefore, it was necessary to develop new molecules to overcome these mutations^17^^,^^18^. The mechanism by which EGFR-TKIs produce their action is by competing with ATP for the EGFR’s ATP-binding site^19–21^ (Figure 1(A)). The reference drug erlotinib (first-generation EGFR-TKI) works by occupying the essential pockets of the ATP-binding site^22^ (Figure 1(B)). HHQ is a well-known scaffold for developing new physiologically active compounds due to its synthetic flexibility^23^. HHQ scaffold shares common pharmacophoric features with ATP, as the HHQ nucleus itself can occupy the same pocket of adenine base of ATP and different substituents on the HHQ ring can play the same role of other parts of the ATP, making it easy to develop EGFR inhibitors that compete with ATP at its active site^24^. HHQ derivatives have a wide range of biological activities, such as anti-inflammatory^25^, antifungal^26^, and also promising anticancer activity^27^; therefore, many projects started to investigate them as anticancer agents. HHQ derivatives were found to exhibit their anticancer activity *via* different mechanisms, such as inhibition of topoisomerase^28^, cell cycle arrest in the G2 phase^29^, and inhibition of tyrosine kinases (EGFR)^30^.

## Rational and design

**Figure 2.**
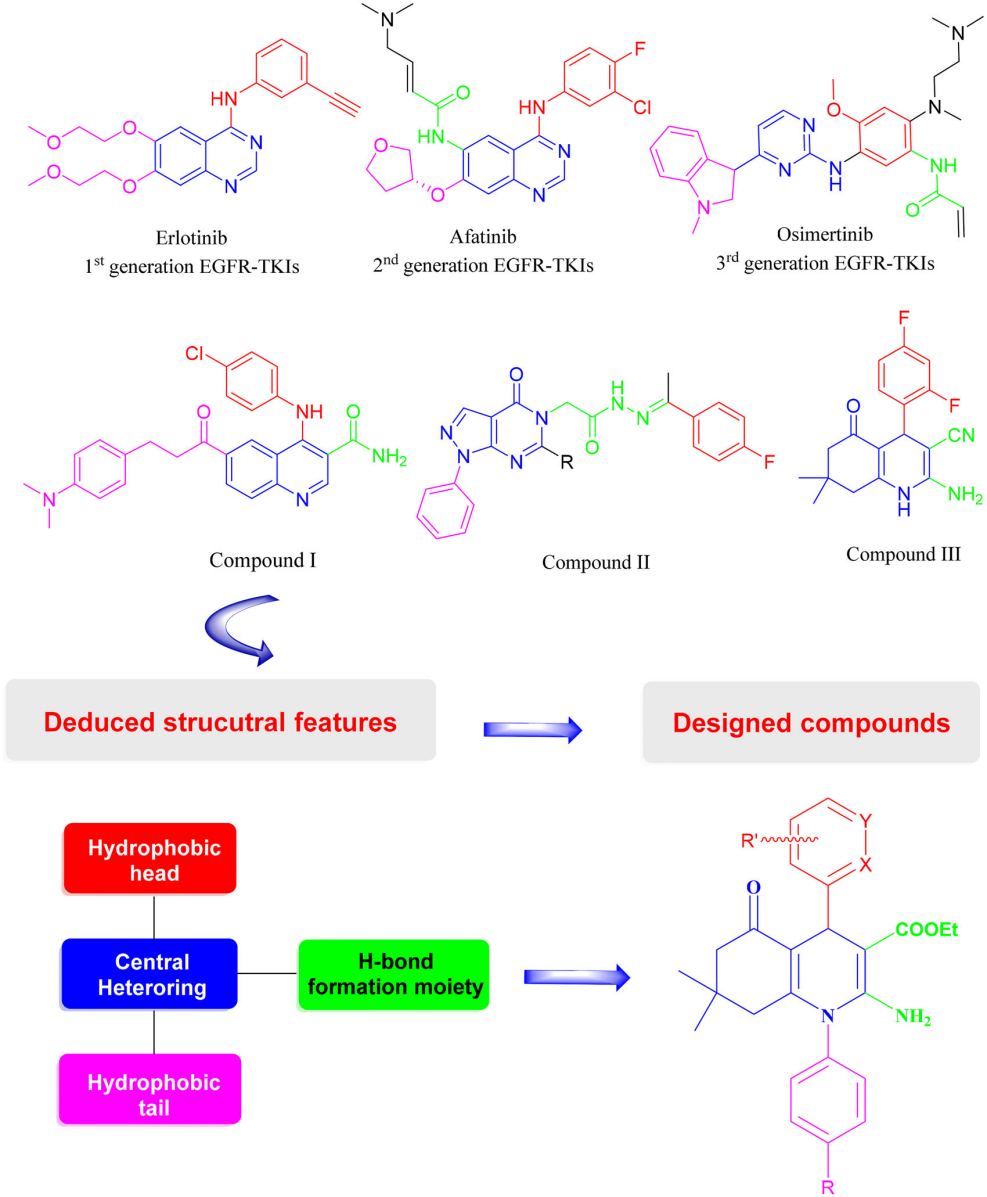
The rational design of new EGFR-TKIs integrating the structural features deduced from the chemical structures of approved EGFR-TKIs (erlotinib, afatinib, and osimertinib) and compounds (**I**–**III**) with reported antitumor activity.

EGFR has an extracellular catalytic domain to which ATP can bind, leading to the activation of EGFR^31^^,^^32^. Small molecules can compete with ATP preventing its binding to the catalytic domain^33^^,^^34^. The approved EGFR inhibitors are divided into; the first-generation agents, erlotinib, and gefitinib^35^; the second-generation agents, afatinib^36^, and dacomitinib^37^; and most recently, osimertinib^38^, a third-generation EGFR-TKI. Besides, many published compounds displayed promising EGFR inhibitory activity, such as compound **I** and compound **II**, which reported anticancer activity against breast cancer^39^. Moreover, compound **II** revealed anticancer activity against breast carcinoma^40^. Compound **III** showed anticancer activity against non-small cell lung cancer^29^ (Figure 2). By reviewing the approved EGFR inhibitors, we have explored that they have shared the following pharmacophoric features; central heterocyclic ring, hydrophobic head, and hydrophobic tail that are similar to those of ATP but lacking a bioisostere of the sugar moiety. Accordingly, we have designed a series of HHQ derivatives satisfying the reported pharmacophoric features: central heterocyclic ring, HHQ ring (occupies the adenine binding region and blue coloured moiety); ester and primary amino groups that interact in the similar manner of ribose moiety (form H-bond with the ATP binding site and green coloured moiety); hydrophobic head (resides in a hydrophobic region I and red coloured moiety); finally a hydrophobic tail, phenyl ring (occupies hydrophobic region II and pink coloured moiety)^41^ (Figure 2).

## Results and discussion

### Chemistry

**Scheme 1.**
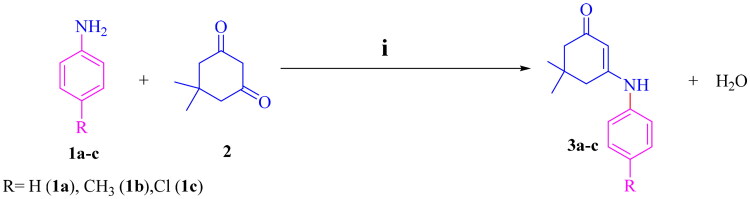
Chemical synthesis of intermediates **3a–c.** Reagents and conditions: (i) *gl.* acetic acid (few drops), heat under reflux in DCM for 8 h.

**Scheme 2.**
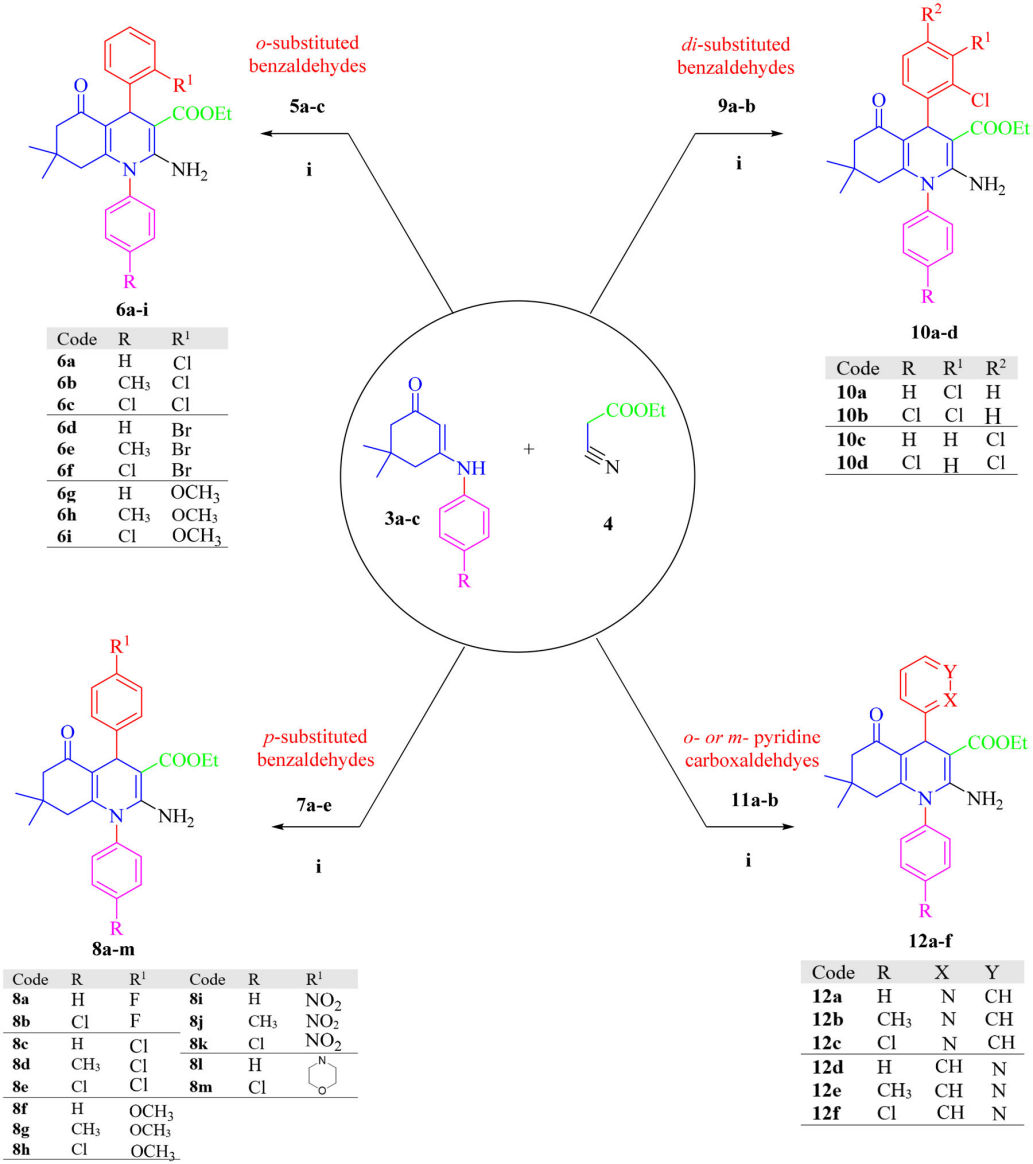
Synthesis of HHQ analogues **6a–i**, **8a–m**, **10a–d**, and **12a–f**. Reagents and conditions: (i) piperidine (few drops), heat under reflux in *abs.* ethanol for 24 h.

The synthetic pathway to prepare the racemic mixture of 32 target compounds is outlined in Schemes 1 and 2. Intermediates, **3a–c**, were synthesised via condensation reaction of aniline derivatives **1a–c** and dimedone **2** in acidic media using dichloromethane (DCM) as a solvent^42^ (Scheme 1). These intermediates, **3a–c**, were reacted with ethyl cyanoacetate **4** and appropriate aryl aldehydes; *o*-substituted benzaldehydes **5a–c**, *p*-substituted benzaldehydes **7a–e**, disubstituted benzaldehydes **9a,b**, or pyridine carboxaldehydes (*ortho* or *meta*) **11a,b**, in absolute ethanol as a solvent and piperidine as a catalyst *via* cyclocondensation mechanism^43^^,^^44^ to yield targeted compounds, **6a–i**, **8a–m**, **10a–d**, and **12a–f** as a racemic mixture (Scheme 2). This reaction was accomplished in two steps; the first step was Knoevenagel condensation between aryl aldehyde and ethyl cyanoacetate leading to the formation of unsaturated nitrile^45^. The second step was achieved *via* Michael’s addition of intermediates **3a–c** to unsaturated nitrile and closure of the HHQ ring. The structures of synthesised target compounds were confirmed by IR, ^1^H NMR, ^13^C NMR, mass spectroscopy, elemental analysis, and x-ray crystallography to confirm enantiomerism. The following illustrations demonstrate that the target compounds were successfully synthesised: for ^1^H NMR spectra, the appearance of both sharp singlets at the range of 4.84–5.44 ppm corresponds to the proton at position 4 in the HHQ ring, a broad peak at the range of 5.97–6.82 ppm, related to NH_2_ at position 2 in the HHQ ring. The disappearance of the NH peak of the intermediates **3a–c** at about 6.50 ppm^46^^,^^47^ and the aldehydic proton at the 9.00–10.00 ppm range was recognised^48^. Regarding the ^13^C NMR spectra, the appearance of a peak in the range of 33.19–36.85 ppm is associated with the carbon atom at position 4 in the HHQ ring. In addition, the disappearance of the peaks at 100–120 ppm for the nitrile of ethyl cyanoacetate^46–48^ and 190–220 ppm for the carbonyl of the aldehyde^49^ was reported and supported by IR spectra, where the peaks at 2300–2250 cm^−1^ (nitrile of ethyl cyanoacetate)^50^ and 3200–3100 cm^−1^ (NH of intermediates **3a–c**) were vanished^51^. The molecular weights of target compounds and the mass spectroscopy results were compatible. Three elements; C, H, and N, underwent elemental analysis to ascertain their percentages, which were within ± 0.4% of theoretical values. The Supplementary file provides the spectra of the synthesised compounds.

### Crystal structure description for compound 6f

**Figure 3.**
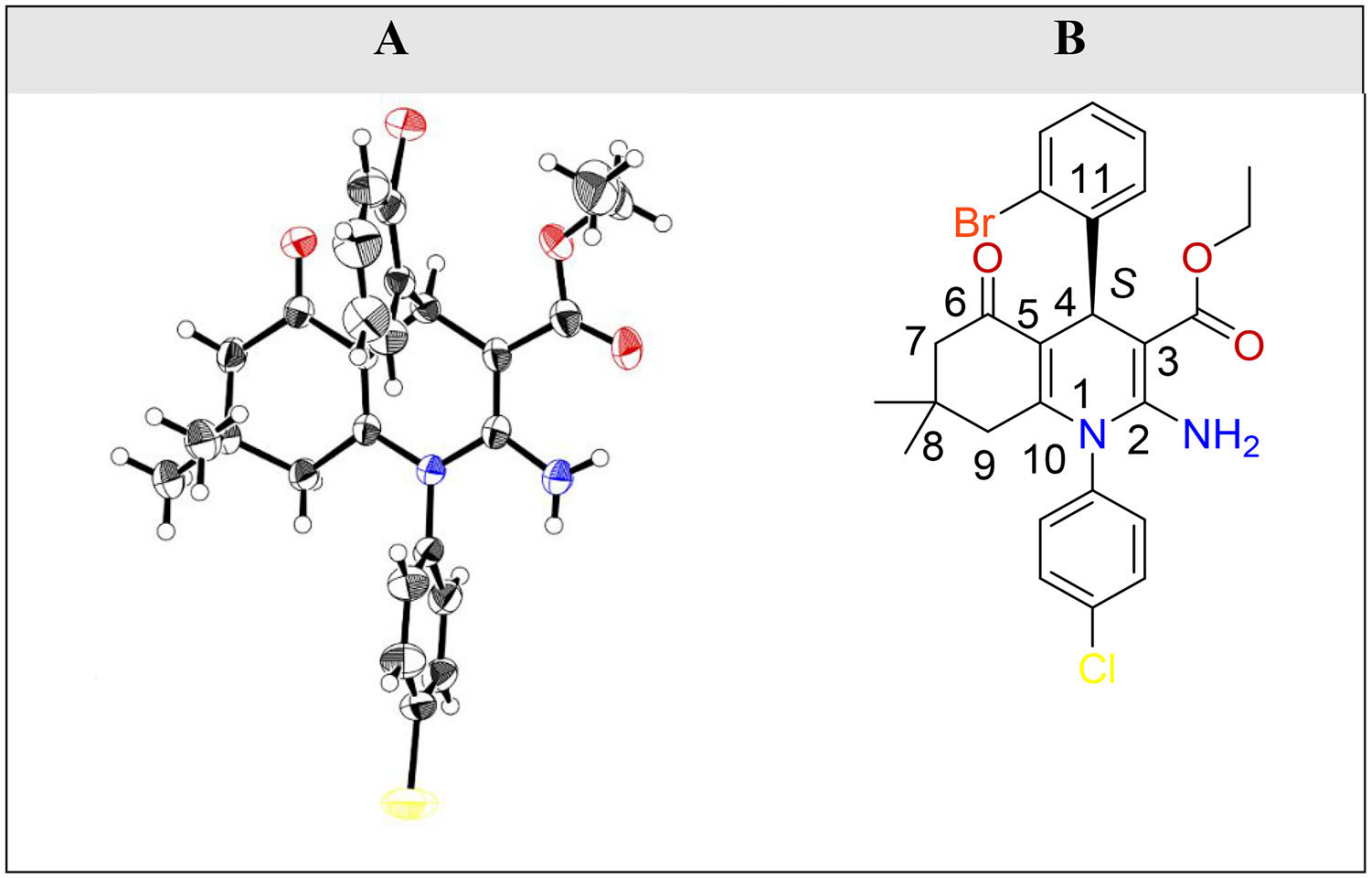
(A) ORTEP diagram of the *S*-enantiomer as a representative for the racemic mixture of compound **6f** obtained from single crystal x-ray data drawn at 50% thermal ellipsoid probability. Red colour: oxygen atom, brick red colour: bromine atom, blue colour: nitrogen atom, and yellow colour: chlorine atom. (B) Chemical structure of *S*-enantiomer of compound **6f**.

We have made several attempts to prepare high-quality crystals for target compounds *via* demanding different solvents, such as methanol, acetone, and chloroform. A good-sized crystals of compound **6f** was successfully used as a representative compound to approve the enantiomerism of our target compounds^52^. A prism crystal of compound **6f** (accession number 2163585) was obtained by crystallisation from methanol at room temperature. The carbon atom at position 4 in the HHQ ring of compound **6f** is chiral; therefore, we may obtain either a pure enantiomer or a racemic mixture as a final product. Therefore, there is no method better than X-ray crystallography to confirm the enantiomerism of our final product^53^. X-ray crystallography revealed a two-component disorder for compound **6f** where the phenyl fragment at C8 was found to adopt two orientations that differ by a 180° rotation around the C4–C11 link; the cyclohexenone ring adopts an envelope configuration, the 1,4-dihydropyridine ring adopts a flattened boat configuration and the phenyl ring at N1 almost perpendicular to the C2/C3/C5/C10 plane^54^^,^^55^. The ORTEP diagram of the *R*-enantiomer for compound **6f** is presented below (Figure 3(A)). Using the visualiser of discovery studio software, we have found that both *S*-enantiomer and *R*-enantiomer are existing. The *S*-enantiomer has an HHQ ring in the plane; the ester and amino groups are directed towards the right-hand side, the two dimethyl groups are directed towards the left-hand side, and the phenyl group at position 4 in the HHQ ring is behind the plane. The *R*-enantiomer has the phenyl group at position 4 in the HHQ ring in front of the plane (Figure 3(B)).

### Biological evaluation

#### In vitro preliminary anticancer activity at a single dose against 60 NCI cell lines

**Figure 4.**
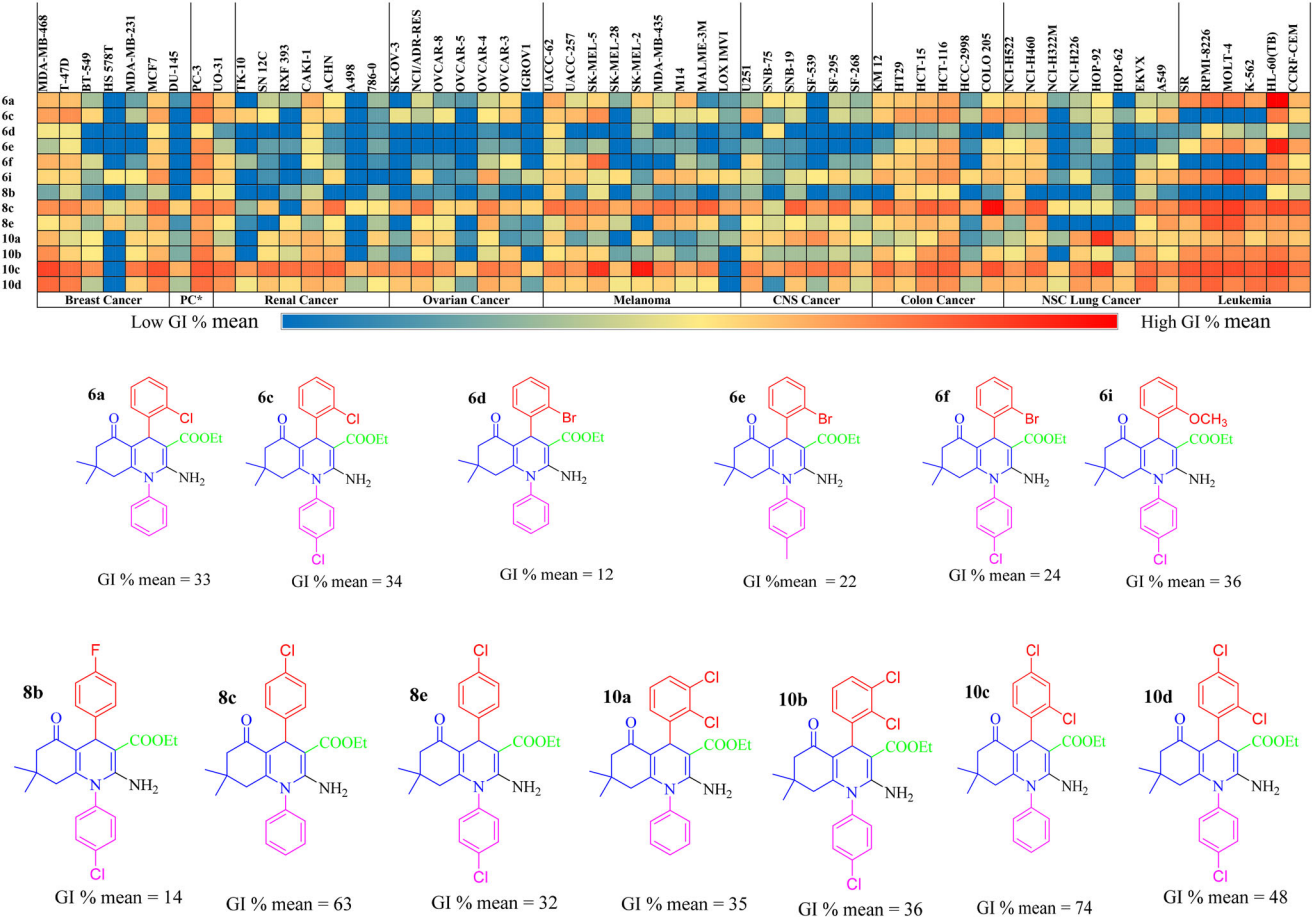
Heat map data representing GI % mean of the active 13 compounds across the NCI-60 human cancer cell line panels, including their structures. *Prostate cancer.

Thirty-two newly synthesised HHQ derivatives were subjected to preliminary anticancer screening at the USA National Cancer Institute (NCI)^56^. HHQ derivatives were examined *in vitro* at one dose anticancer activity against total NCI 60 cancer cell line panels that include nine different types of cancers; leukaemia, NSC lung cancer, colon cancer, CNS cancer, melanoma, ovarian cancer, renal cancer, prostate cancer, and breast cancer^56–59^. Nineteen derivatives failed to show cytotoxic activity against the tested cell lines reporting a GI % mean of less than 10%. These compounds are; **6b**, **6g**, **6h**, **8a**, **8d**, **8f**–**m**, and **12a**–**f** (Tables S1 and S2) and (Figures S100, S105, S106, S108, S111, S113–120, and S129–134). The remaining thirteen derivatives **6a**, **6c**–**f**, **6i**, **8b**–**c**, **8e,** and **10a**–**d** revealed modest to good cytotoxic activity. The data were specified as a mean graph of the treated cells’ percent growth (GI% mean) and represented as a heat map, where cytotoxicity increased from the left (blue) towards the right (red), as shown in Figure 4. Leukaemia and colon cancer were the most sensitive to our target compounds; on the other hand, renal and ovarian cancers were the least sensitive.

**Table 1.** Effects of five doses for *in vitro* anticancer activity results (cytotoxic activities expressed as GI_50_ (µM) for compound **10c** against NCI sixty cancer cell lines.

Subpanel cell lines	GI_50_	Subpanel cell lines	GI_50_	Subpanel cell lines	GI_50_	Subpanel cell lines	GI_50_
Leukaemia		COLO-205	2.06	MDA-MB-435	3.21	RXF 393	1.38
CCRF-CEM	3.10	HCC-2998	2.77	SK-MEL-2	1.70	SN 12 C	3.08
HL-60(TB)	5.95	HCT-116	7.89	SK-MEL-28	6.67	TK-10	5.63
K-562	7.23	HCT-15	1.52	SK-MEL-5	1.24	UO-31	1.15
MOLT-4	9.56	HT-29	1.98	UACC-257	2.88	Prostate cancer	
RPMI-8226	7.07	KM-12	2.36	UACC-62	1.26	PC-3	4.14
SR	1.47	SW-620	2.90	Ovarian cancer		DU-145	6.71
NSC Lung cancer		CNS cancer		IGROV1	7.97	Breast cancer	
A549	1.88	SF-268	5.32	OVCAR-3	2.62	MCF7	1.77
EKVX	2.62	SF-295	2.03	OVCAR-4	2.83	MDA-MB-231	3.34
HOP-62	4.88	SF-539	3.78	OVCAR-5	7.26	HS 578 T	7.68
HOP-92	3.89	SNB-19	2.92	OVCAR-8	3.72	BT-549	4.58
NCI-H226	2.34	SNB-75	5.33	SK-OV-3	3.35	T-47D	1.04
NCI-H23	2.51	U251	2.46	Renal cancer		MDA-MB-468	6.83
NCI-H322-M	7.71	Melanoma		786-0	4.24	LC_50_ values towards 60 cell lines were more than 100 µM	
NCI-H460	1.39	LOX IMVI	3.24	A498	4.54		
NCI-H522	2.22	MALME-3M	2.93	ACHN	1.83		
Colon cancer		M14	3.48	CAKI-1	6.82		

**Table 2.** Inhibitory activities of the most potent 13 compounds with standard drug erlotinib against EGFR^WT^, EGFR^T790M^, and EGFR^L858R^ (the results are reported as the means of IC_50_ values ± standard deviation for three independent replicates).

Code	IC_50_ ± SD (µM)			Code	IC_50_ ± SD (µM)		
	Wild type	Mutant types			Wild type	Mutant types	
	EGFR^WT^	EGFR^T790M^	EGFR^L858R^		EGFR^WT^	EGFR^T790M^	EGFR^L858R^
**6a**	0.358 ± 0.16	0.61 ± 0.005	0.49 ± 0.004	**8c**	0.194 ± 0.09	0.820 ± 0.006	0.812 ± 0.005
**6c**	0.277 ± 0.12	0.53 ± 0.004	0.50 ± 0.004	**8e**	0.300 ± 0.12	0.693 ± 0.005	0.351 ± 0.002
**6d**	0.099 ± 0.02	0.41 ± 0.002	0.25 ± 0.005	**10a**	0.145 ± 0.03	0.532 ± 0.004	0.490 ± 0.003
**6e**	0.176 ± 0.07	0.48 ± 0.003	0.37 ± 0.004	**10b**	0.205 ± 0.09	0.954 ± 0.008	0.878 ± 0.006
**6f**	0.313 ± 0.12	0.59 ± 0.004	0.48 ± 0.003	**10c**	0.148 ± 0.04	0.586 ± 0.003	0.623 ± 0.005
**6i**	0.157 ± 0.03	0.45 ± 0.003	0.23 ± 0.001	**10d**	0.097 ± 0.005	0.280 ± 0.001	0.051 ± 0.001
**8b**	0.186 ± 0.1	0.65 ± 0.004	0.77 ± 0.006	**Erlotinib**	0.082 ± 0.005	0.342 ± 0.001	0.055 ± 0.001

Relying on the GI % mean values (Tables S1 and S2), the 2,3- and 2,4-dichlorophenyl derivatives, **10a–d**, were the most active compounds where 2,4-dichlorophenyl analogues, **10c** and **10d,** displayed higher activity than 2,3-dichlorophenyl derivatives, **10a** and **10b**. Compound **10c** (GI % mean = 74) was the most active analogue and almost revealed cytotoxic activity against 58 cell lines out of the 60 screened cell lines, with activity ranging from moderate to lethal. Compound **10c** exposed lethal anticancer activity against NSC lung cancer; HOP-92, melanoma; SK-MEL-2, SK-MEL-5. It reported strong anticancer activity towards leukaemia, colon, breast, renal, and prostate cancer, while it revealed moderate activity against ovarian cancer. Compound **10d** (GI % mean = 48) disclosed strong anticancer activity against leukaemia, NSC lung cancer; EKVX, NCI-H226, colon cancer; HCT-116, HCT-15, prostate cancer; PC-3 and breast cancer; MCF7, T-47D, MDA-MB-468. Compound **10a** (GI % mean = 35) revealed lethal anticancer activity against NSCL cancer; HOP-92, strong anticancer activity against leukaemia; MOLT-4, colon cancer; HCT-116, CNS cancer; SF-539, prostate cancer; PC-3. Compound **10b** (GI % mean = 36) displayed strong anticancer activity against leukaemia; MOLT-4 colon cancer; HCT-116, HCT-15, prostate cancer; PC-3, and breast cancer; MDA-MB-468.

*Para* substituted phenyl derivatives, **8b,c** and **8e,** arose in the next place in the antitumor activity. Halogenated derivatives (**8b** and **8e)** displayed higher cytotoxicity than non-halogenated analogues (**8i**, **8j,** and **8 l**). Compound **8b** (GI % mean = 14) reported mild cytotoxicity towards NSC lung cancer; NCI-H522, colon cancer; HCT-116, HCT-15, HT29, and melanoma; UACC-62. Analogue **8e** (GI % mean = 32) showed remarkable anticancer activity against leukaemia; CCRF-CEM, HL-60 (TB), K-562, MOLT-4, RPMI-8226, and NSC lung cancer; A549. Compound **8c** (GI % mean = 63) was the most active analogue in this series and revealed a significant broad spectrum against the nine types of cancer as it disclosed lethal activity towards colon cancer; COLO 205 with strong anticancer activity against leukaemia; CCRF-CEM, HL-60 (TB), K-562, MOLT-4, RPMI-8226, SR, NSC lung cancer; A549, EKVX, NCI-H460, NCI-H522, colon cancer; HCT-116, HCT-15, HT29, KM12, SW-620, CNS cancer; SF-295, SF-539, SNB-19, melanoma; M14, MDA-MB-435, SK-MEL-2, SK-MEL-5, UACC-257, UACC-62, ovarian cancer; OVCAR-4, NCI/ADR-RES, renal cancer; ACHN, UO-31, prostate cancer; PC-3, and breast cancer; MCF7, T-47D, MDA-MB-468.

Moreover, *ortho*-substituted phenyl derivatives **6a, 6c–f,** and **6i** showed mild antitumor activity. Halogenated derivatives (**6a** and **6c–f**), except for the methoxy analogue **6i** (GI % mean = 36), were more cytotoxic than non-halogenated analogues (**6 g**–**i**). Compound **6a** (GI % mean = 33) displayed a lethal effect against leukaemia HL-60 (TB), while it revealed strong anticancer activity towards leukaemia; CCRF-CEM, MOLT-4, RPMI-8226, SR, and prostate cancer; PC-3. Compound **6c** (GI % mean = 34) disclosed strong anticancer activity against leukaemia HL-60 (TB), NSLC; NCI-H522 colon cancer; COLO 205, HCT-116, HCT-15, HT29, melanoma; SK-MEL-5, prostate cancer; PC-3, and breast cancer; T-47D, MDA-MB-468. Compound **6d** (GI % mean = 12) reported mild cytotoxicity against most tested cancer cell lines. Compound **6e** (GI % mean = 22) displayed a lethal effect on leukaemia HL-60 (TB), while it revealed strong anticancer activity against leukaemia; CCRF-CEM, MOLT-4, RPMI-8226, colon cancer; HCT-116, and prostate cancer; PC-3. Compound **6f** (GI % mean = 24) exposed strong anticancer activity against leukaemia; HL-60 (TB), melanoma; SK-MEL-5, and prostate cancer; PC-3, while it displayed moderate activity against colon cancer; HCT-116. Compound **6i** (GI % mean = 36) revealed strong anticancer activity against leukaemia; HL-60 (TB), K-562, MOLT-4, RPMI-8226, SR, colon cancer; HCT-116, prostate cancer; PC-3, and breast cancer; MCF7.

##### Structure–activity relationship (SAR)

We have constructed our structure–activity relationship (SAR) study based on NCI single-dose biological evaluation outcomes. Generally, we will discuss the influence of diverse substituents of two important parts on target compounds: the substituted phenyls at position N1 and the aryl groups at position-4 of the HHQ ring.

**Figure 5.**
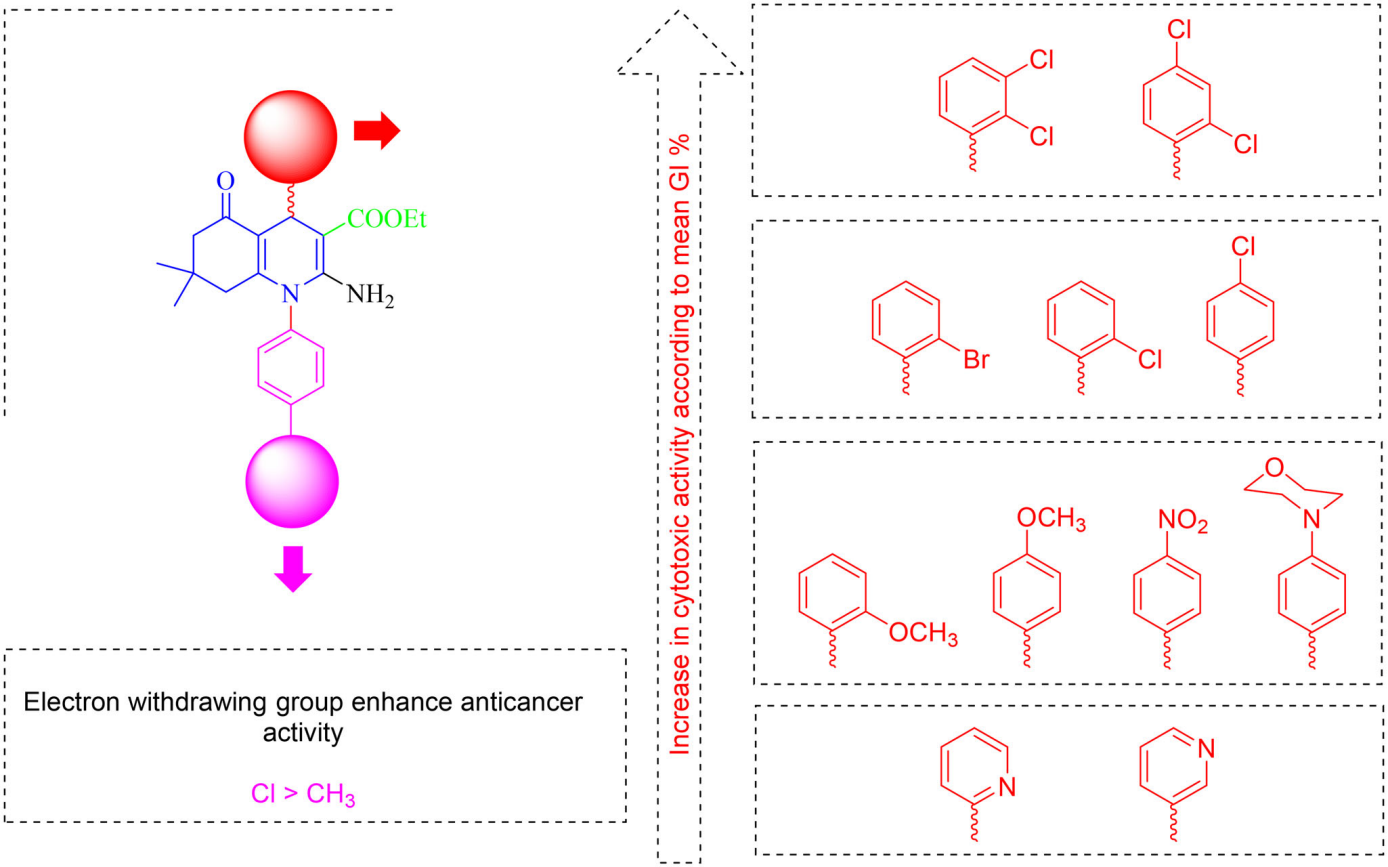
Summary of structure-activity relationship (SAR) of target compounds as *in vitro* anticancer agents against NCI 60 human cancer cell lines relying on the GI % mean values.

1. Regarding substituted phenyls at N1, generally the electron-withdrawing group (R = Cl) enhanced cytotoxic activity compared to the electron-donating group (R = CH_3_). Relying on GI % mean results, we can confirm that:
  - The presence of chlorine atom at the *para* position of phenyl ring at C-4: Analogues with unsubstituted phenyl (**8c**, **10c**) were better in activity than substituted analogues with electron-withdrawing (Cl) (**8e**, **10d**) or electron-donating (CH_3_) groups (**8d**). Analogues substituted with electron-withdrawing group reported higher cytotoxic activity compared to the analogues having electron-donating groups (H > Cl > CH_3_).
  - The existence of chlorine atom at the *ortho* or *meta* positions of phenyl ring at C-4: Analogues with unsubstituted phenyl (**6a, 10a**) were almost equipotent to those with electron-withdrawing substituent (Cl) (**6c, 10b**) and more potent than analogues substituted with electron-donating groups (CH_3_) (**6b**) (H ∼ Cl > CH_3_) (**6a**∼**6c** ≫ **6b**), (**10a**∼**10b**).
2. Concerning the aryl groups at position-4:
  - Upon incorporation of *ortho*-substituted aryl at position 4 (target compounds **6a–i),** an electron-withdrawing group, especially halogens at the *ortho* position of the C4 aryl enhanced anticancer activity whereas analogue bearing halogen of medium size, such as (Cl) was more potent than those having halogen of large size like (Br) (R^1^; Cl > Br) (**6a** > **6d**) in comparison with electron-donating groups (R^1^ = OCH_3_) (**6c** > **6f**) (**6i** is an exception).
  - Upon integration of *para*-substituted aryl at position 4 (analogues **8a–m),** the electron-withdrawing groups at *para* position potentiate anticancer activity (R^1^; Cl ⋙ F) in comparison with an electron-donating group (R^1^ = OCH_3_) (**8c** > **8a**).
  - Regarding disubstituted aryl at position 4 (compounds **10a–d**): Disubstitution with EWG at *ortho* and *para* positions increases cytotoxic activity than *ortho* and *meta* positions (**10c,d** ≫ **10a,b**). In contrast, pyridine analogues (**12a–f),** substituted with a heteroaromatic ring at the *para* position, do not affect biological activity. The SAR is summarised in Figure 5.

#### In vitro anticancer screening at five doses of full NCI 60 cancer cell panels

The second stage was accomplished *via* screening of the most active compound (**10c**) referred to the results of the one-dose analysis of the NCI **10c** (NSC 838215) against the 60 cancer cell lines at five different concentrations (0.01, 0.1, 1, 10, and 100 µM). Cell viability was assessed using the published experimental techniques using the sulforhodamine-B (SRB) protein assay spectrophotometrically *versus* untreated with a test compound^60^. At the end of the 48 h incubation period, the five-dose assay findings were presented for each cell line tested regarding the response parameters, GI_50_ (needed molar concentration to inhibit 50% of cancer cell line growth)^61^. Compound **10c** displayed magnificent anticancer activity against nearly all the tested cell lines, with GI_50_ ranging from 1.04 to 9.56 µM. The best cytotoxic effect was observed against the T-47D breast cancer cell line (GI_50_ = 1.04 µM), while the least cytotoxic effect was observed against the MOLT-4 leukaemia cell line (GI_50_ = 9.56 µM). The most sensitive cancer cell lines were those having GI_50_ > 2 µM. SR was the most sensitive leukaemia cell line with GI_50_ = 1.47 µM. A549 and NCI-H460 were the most sensitive NSC lung cancer cell lines with GI_50_ = 1.88 and 1.39 µM, respectively. HCT-15 and HT-29 were the most sensitive colon cancer cell lines with GI_50_ = 1.52 and 1.98 µM, respectively. Regarding melanoma, SK-MEL-2, SK-MEL-5, and UACC-62 were the most sensitive cell lines with GI_50_ = 1.70, 1.24, and 1.26 µM, respectively. Renal cancer was also one of the most affected cancers, specifically ACHN, RXF-393, and UO-31 cell lines with GI_50_ =1.83, 1.38, and 1.15 µM, respectively. It revealed excellent cytotoxic activity against breast cancer, MCF7 with GI_50_ =1.77 µM. Table 1 summarises the calculated GI_50_ values for each of the sixty cancer cell lines for compound **10c** across the nine cancer types.

#### Enzyme inhibition assay of EGFR^WT^, EGFR^T790M^, and EGFR^L858R^

In the T790M mutation, methionine replaces threonine at amino acid position 790 of exon 20 of the EGFR gene^62^. This mutation alters the crystal structure of the adenosine triphosphate (ATP) binding pocket and prevents binding to the ATP binding site; hence structurally blocking first- and second-generation EGFR-TKIs and results in EGFR-TKIs resistance^63^. The “gatekeeper” hypothesis, which holds that there is a steric clash between the larger methionine moiety (compared to threonine) on the gatekeeper side chain of EGFR^T790M^ and the aniline moiety of first-generation EGFR TKIs, is one of the more biochemical mechanisms of EGFR^T790M^-associated resistance^64^. Concerning the L858R mutation, arginine replaces leucine at the 858th amino acid of the 21st exon of the EGFR^65^. It’s the most common EGFR mutation, accounting for 35% of all mutations^66^. The first-generation EGFR-TKIs include erlotinib and gefitinib^67^. The second-generation EGFR-TKIs (such as afatinib^68^, pelitinib^69^, neratinib^70^, and dacomitinib which is an irreversible inhibitor with pan-HER TKI action^71^ created to combat resistance to first-generation EGFR-TKIs, although acquired resistance to these drugs always appears. Studies demonstrated that the EGFR gene’s T790M mutation was a typical resistance mechanism to second-generation EGFR-TKIs^72^. As a result, third-generation, EGFR-TKIs were created to overcome resistance to first- and second-generation EGFR-TKIs, and they allowed the irreversible binding to Cys797 residue in the ATP-binding site (e.g. osimertinib^73^, rociletinib^74^, and olmutinib^75^.

**Figure 6.**
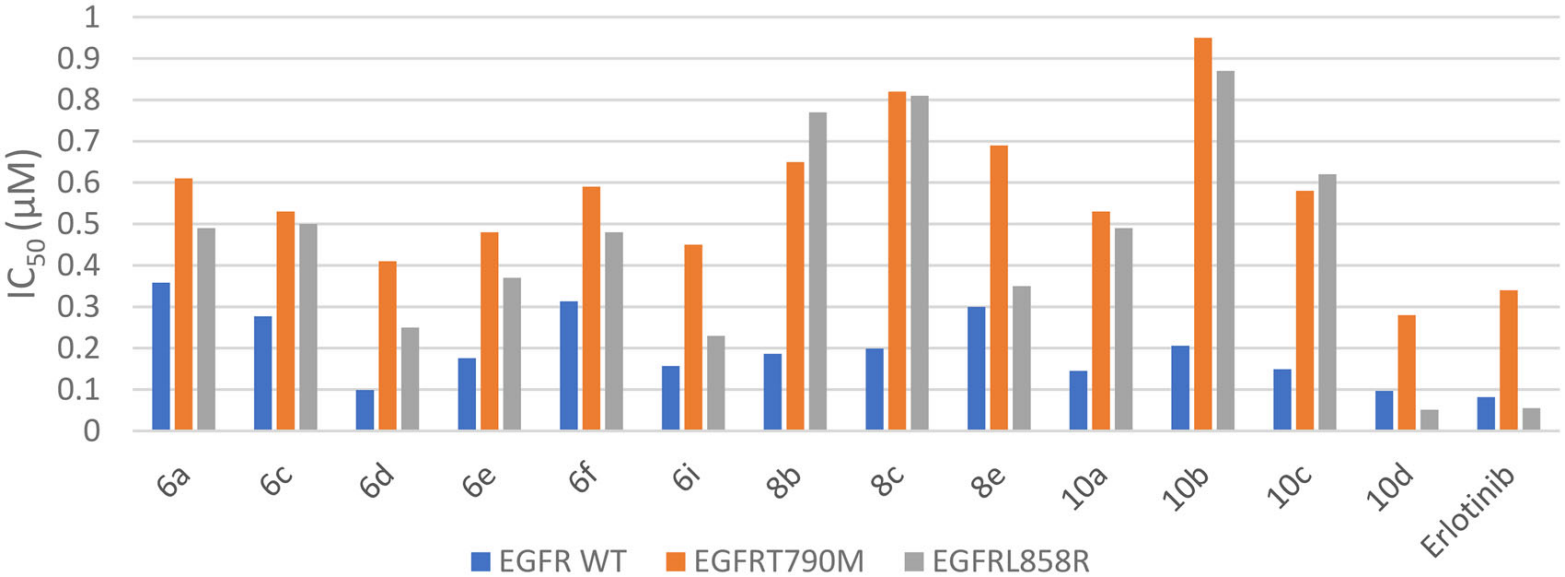
Inhibitory activity outcomes (IC_50_ µM) for the most potent 13 compounds **6a**, **6c–f**, **6i**, **8b**,**c**, **8e** and **10a–d** against EGFR^WT^, EGFR^T790M^, and EGFR^L858R^.

The target compounds were designed as inhibitors of EGFR. The most potent 13 compounds against the NCI cell lines panel **6a**, **6c–f**, **6i**, **8b**,**c**, **8e**, and **10a–d** were screened for their enzymatic inhibitory activity against wild-type (EGFR^WT^), L858R mutant (EGFR^L858R^), and T790M mutant (EGFR^T790M^) receptors using erlotinib as a reference drug. Concerning EGFR^WT^ results, the tested compounds showed antitumor activity ranging from 0.097 to 0.358 µM, where compound **10d** reported the highest cytotoxic activity (IC_50_ = 0.097 µM) which was very close to erlotinib (IC_50_ = 0.082 µM). In comparison, compound **6a** displayed the lowest cytotoxic activity (IC_50_ = 0.358 µM) (Table 2). Regarding EGFR^T790M^ results, IC_50_ values were ranged from 0.280 to 0.954 µM, with compound **10d** recording the best cytotoxic activity (IC_50_ = 0.280 µM), which was higher than erlotinib (IC_50_ = 0.342 µM). On the other hand, compound **10b** disclosed the lowest cytotoxic activity (IC_50_ = 0.954 µM) (Table 2). For EGFR^L858R^ outcomes, IC_50_ values were in the range of 0.051–0.878 µM. Compound **10d** revealed the highest cytotoxic activity (IC_50_ = 0.051 µM) with a privilege over erlotinib (IC_50_ = 0.055 µM). In contrast, compound **10b** showed the lowest cytotoxic activity (IC_50_ = 0.878 µM) (Table 2). The graph of the IC_50_ values clearly illustrated these findings (Figure 6). Accordingly, compound **10d** is the most active compound with remarkable enzymatic inhibitory activities against the wild and mutant EGFRs.

#### Cytotoxic effects in vitro on IMR-90 cells (normal lung cells)

The human normal lung fibroblast (IMR-90) cell line^76^^,^^77^ was used to evaluate the target compounds **10c** and **10d** as cytotoxic agents to assess their safety profile through their selective cytotoxicity towards cancer cells compared to normal cells using erlotinib as a reference anticancer drug. In contrast to erlotinib (IC_50_ = 39.55 ± 1.15 μM), compounds **10c** and **10d** displayed less cytotoxicity towards the human normal cells, IMR-90, with IC_50_ values of 62.17 ± 3.14 and 55.46 ± 2.57 μM, respectively. Therefore, compared to the standard anticancer treatment, erlotinib, compounds **10c** and **10d** are far safer and have fewer possible negative effects on normal cells.

#### Annexin V-FITC apoptosis assay

**Figure 7.**
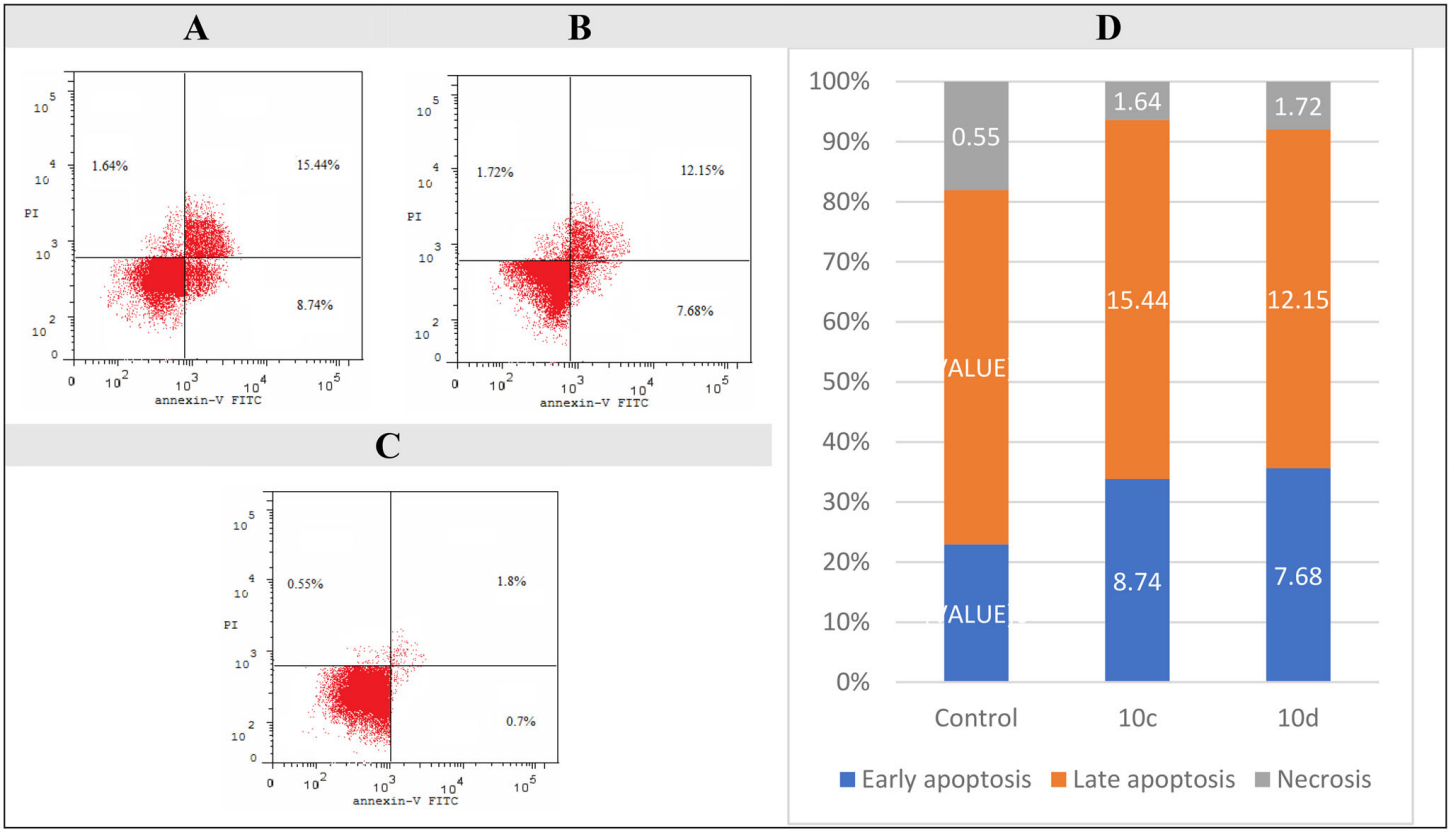
Apoptosis assay in flow cytometry, the effect of **10c** (A), compounds **10d** (B), control (C), and bar chart presentation of control, compounds **10c** and **10d** (D) on the percentage of Annexin V-FITC-positive staining in NSC lung cancer HOP-92 cells. The four quadrants were identified as LL: viable; LR: early apoptotic; UR: late apoptotic; UL: necrotic.

The most important mechanism chemotherapeutics eradicate cancer cells is apoptosis induction^78^^,^^79^. Cellular alterations brought on by apoptosis include the translocation of phosphatidylserine (PS) from the inside to the outside via the plasma membrane^80^^,^^81^. PS can be detected outside the plasma membrane using a sensitive probe called Annexin V, which can bind to PS^82^^,^^83^. We used a FITC/AV/PI dual-staining assay with the BD FACSCalibur to perform cytometric analysis to separate the apoptosis from the necrosis mode of lung cancer HOP-92 cells (the most sensitive NSC lung cancer cell line to target compounds) death caused by the two most active compounds, **10c** and **10d** (BD Bio-sciences, San Jose, CA). HOP-92 cells were also overexpressed in NSC lung cancer^84^. Therefore, they were selected to be subjected to apoptosis assay. HOP-92 cells were stained with AV/PI for 24 h at a mixed molar concentration of 10 µM with compounds **10c** and **10d** (Figure 7(A,B)). We have established that the late apoptosis ratio (upper-right quadrant of the cytogram) increased to 15.44 and 12.15% (Figure 7(A,B)) after it was 1.80% (DMSO) (Figure 7(C)). The early apoptosis ratio (lower-right quadrant of the cytogram) increased to 8.74 and 7.68% (Figure 7(A,B)) after it was 0.70% in the control sample (DMSO) (Figure 7(C)) for compounds **10c** and **10d,** respectively. These findings supported that the cytotoxic effects of compounds **10c** and **10d** were due to the apoptotic mechanism rather than the necrotic pathway. The results of compounds **10c** and **10d** compared to the control sample are presented as a bar chart (Figure 7(D)).

#### Cellular mechanistic analysis

**Figure 8.**
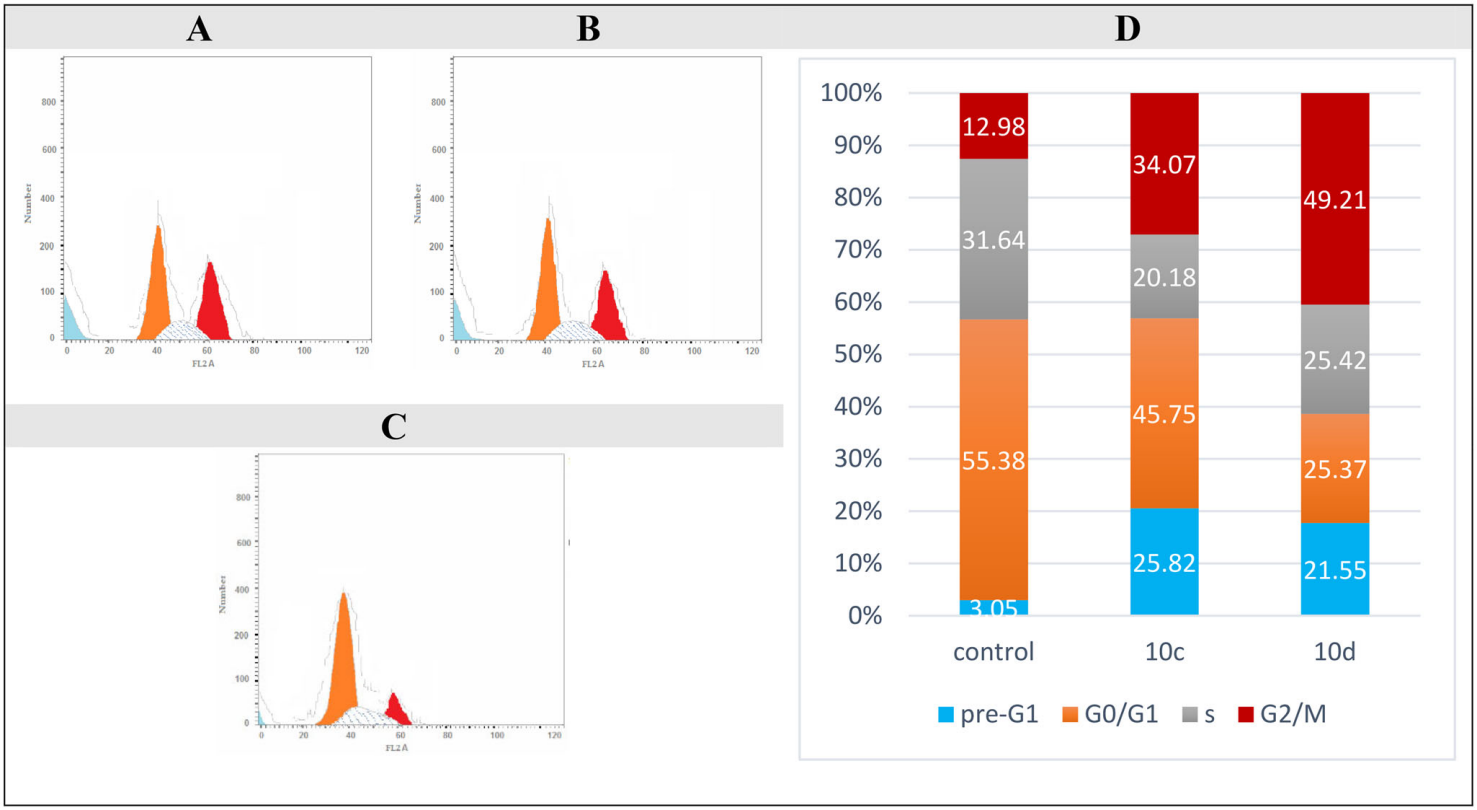
Cell distribution in pre-G1, G0/G1, S, and G2/M phases for NSC lung cancer, HOP-92, cells after treatment with **10c** (A), compounds **10d** (B), and control (C). Bar chart presentation of cell distribution for control, compounds **10c** and **10d** (D).

Antitumor drugs can cause cell cycle arrest and death via activating signalling pathways^85^^,^^86^. Cell growth in various cell cycle phases is measured by flow cytometry^87^^,^^88^. The most potent compounds, **10c** and **10d**, were picked for additional investigation to discover how they could affect cell cycle progression in the NSC lung cancer cells, HOP-92 (Figure 8(A,D)). We have treated HOP-92 cells with 10 µM of compounds **10c** and **10d** to incubate for 24 h. We have established a remarkable increase in the percentage of cells at the pre-G1 phase 25.82% Figure 8(A) and 21.55% Figure 8(B) after being 3.05% in the control sample Figure 8(C), as well as an increase in cells at G2/M to be 34.07% Figure 8(A) and 49.21% Figure 8(B) after it was 12.98% in the control sample Figure 8(C) for compounds **10c** and **10d**, respectively. On the other hand, a drop in the percentage of cells in the G0/G1 to 45.75% Figure 8(A) and 25.37% Figure 8(B) instead of 55.38% (control) Figure 8(C) and a significant reduction in the proportion of cells in the S phase to become 20.18% Figure 8(A) and 25.42% Figure 8(B) rather than 31.46% (control) Figure 8(C) were detected for compounds **10c** and **10d**, respectively. According to these observations, Compounds **10c** and **10d** induce apoptosis in NSC lung cancer, HOP-92, cells via cell cycle arrest at the pre-G1 phase as well as the G2/M phase (Figure 8(D)).

### In silico pharmacokinetic prediction

**Figure 9.**
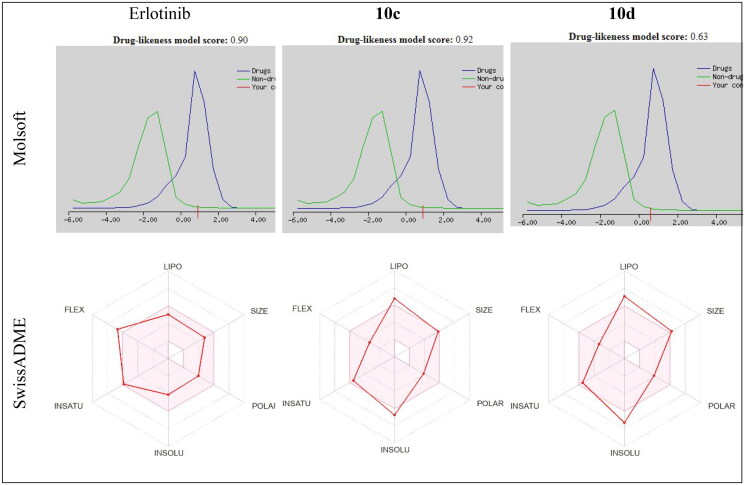
Drug-likeness model score and radar charts results of erlotinib, compounds **10c** and **10d**.

Regarding Molsoft software^89^, the results revealed that drug-likeness scores are 0.90, 0.92, and 0.63 for erlotinib and compounds **10c** and **10d,** respectively. On the other hand, by looking at the SwissADME web tool radar chart^90^, the compounds **10c** and **10d** gave better flexibility than erlotinib with a slight deviation in lipophilicity and insolubility. From these, we can conclude that compounds **10c** and **10d** are suggested to be drugs (Figure 9).

### Molecular docking analysis

A molecular docking study was achieved to establish a deep insight into the binding mode of the *R*- and *S*-enantiomers for the most potent antitumor compounds, **10c** and **10d**, within the ATP-binding pocket of the crystal structures for each enzyme: EGFR^WT^ (PDB code: 1M17)^91^, EGFR^T790M^ (PDB code: 2JIV)^92^ and EGFR^L858R^ (PDB code: 4LQM) by using “molecular operating environment (MOE) version 2019.0102”^93^. Visualisation of interactions between ligands and binding sites was accomplished via discovery studio visualiser (BIOVIA-2021.DS2021Client)^94^^,^^95^. The active site of the EGFR enzyme contains essential amino acids; Lys721, Met742, Met766, Gly767, Met769, Leu694, and Asp831 which are chiefly targeted by EGFR inhibitors^96^. Another essential amino acid is Cys797, the third-generation EGFR-TKIs conquer the T790M and L858R mutations resistance through covalent binding with Cys797^97^. The docking results revealed that our target compounds demonstrated different types of bonds with these amino acids and illustrated the results of the biological evaluation. Looking at the literature, we find that erlotinib binds with the key amino acids in the binding site through H-bonding with Met769 and hydrophobic interactions with other amino acids, including the key amino acid Lys721 and Leu694^98^^,^^99^. Considering the docking studies, compounds **10c** and **10d** showed a binding affinity for the active site of EGFR comparable to that found for erlotinib.

**Figure 10.**
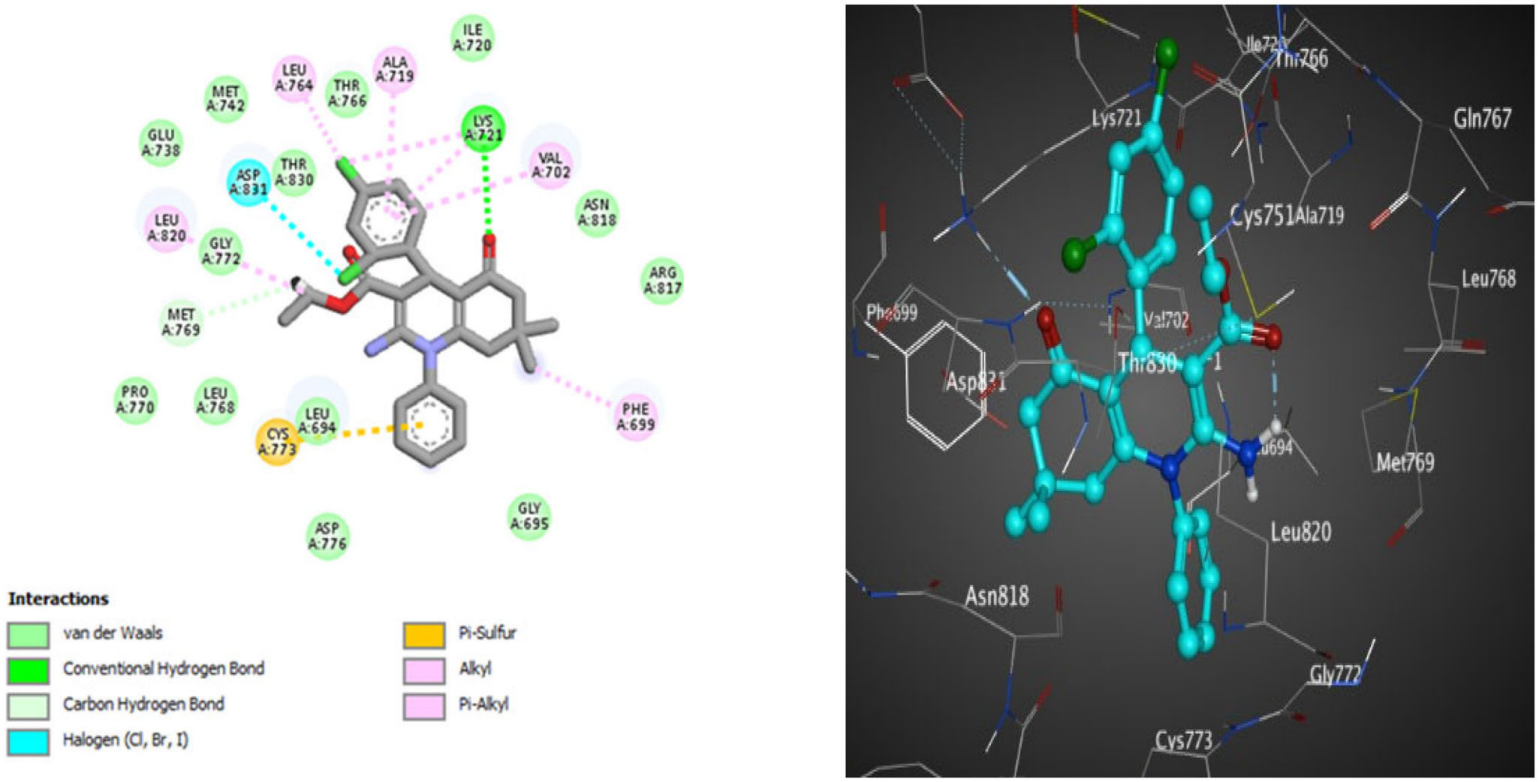
The 2D (left) and 3D (right) poses for docking interactions of the *S*-isomer of compound **10c** within the active site of EGFR (PDB code: 1M17).

**Figure 11.**
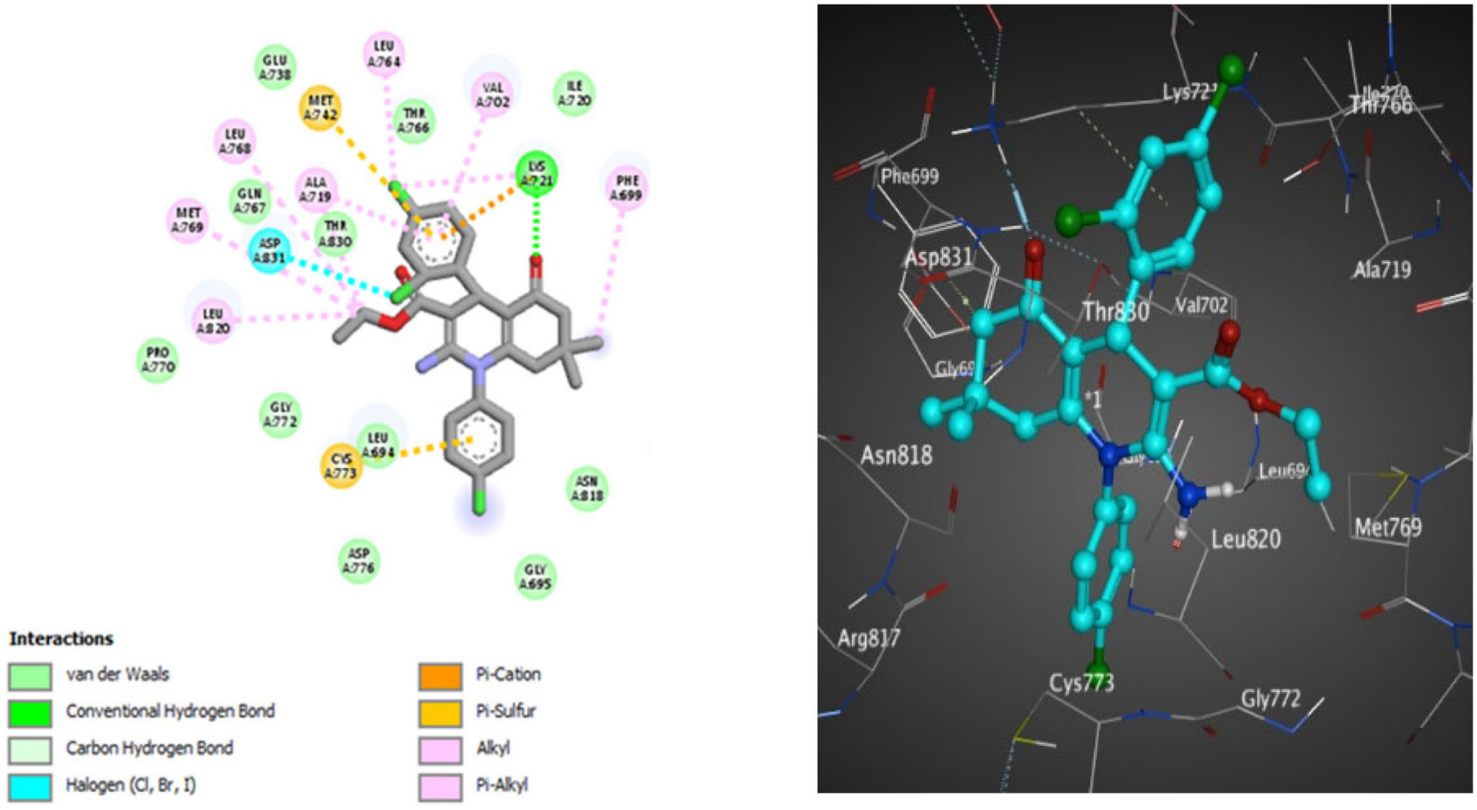
The 2D (left) and 3D (right) poses for docking interactions of the *S*-isomer of compound **10d** within the active site of EGFR (PDB code: 1M17).

**Figure 12.**
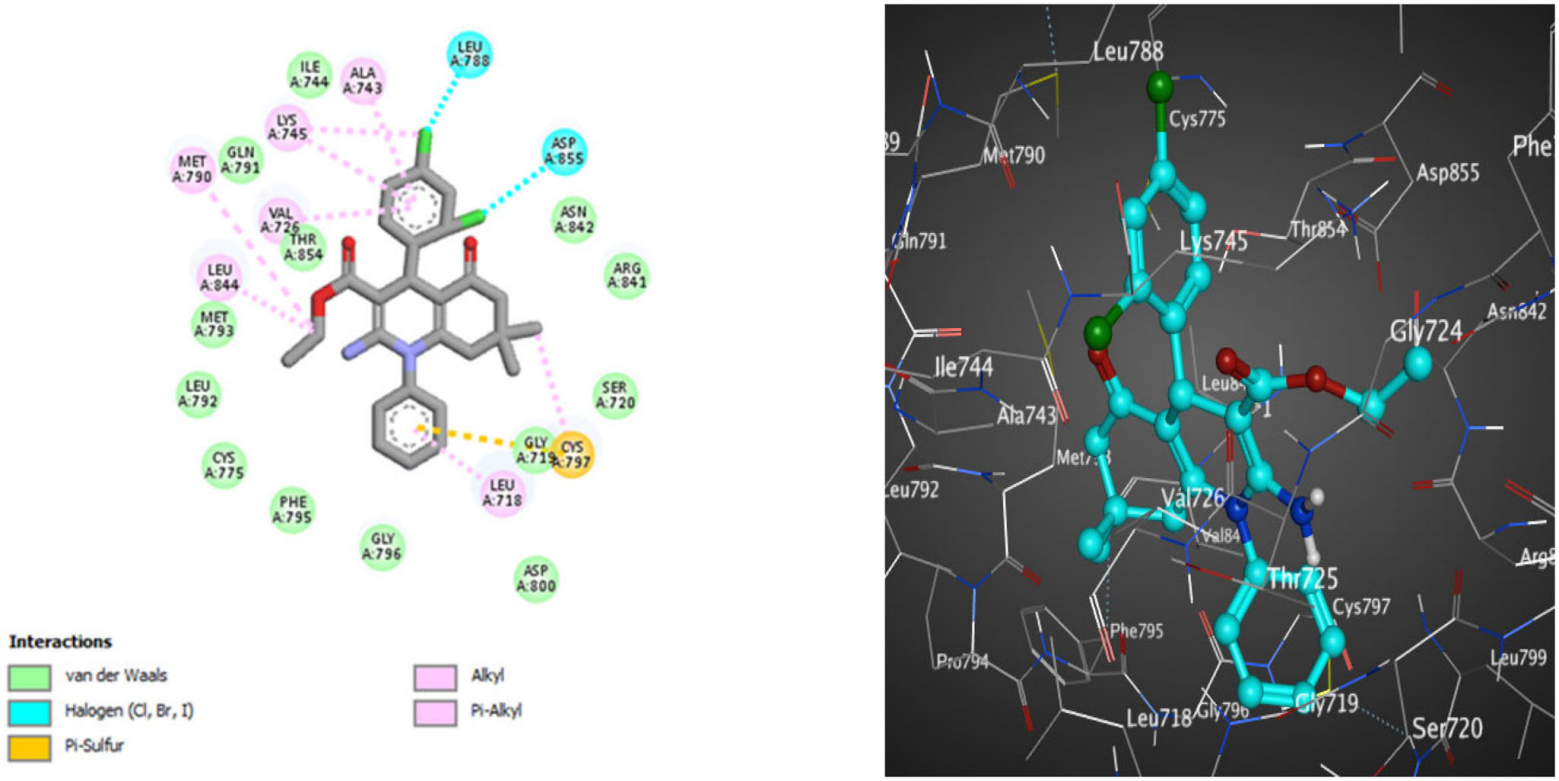
The 2D (left) and 3D (right) poses for docking interactions of the *S*-isomer of compound **10c** within the active site of EGFR (PDB code: 2JIV).

**Figure 13.**
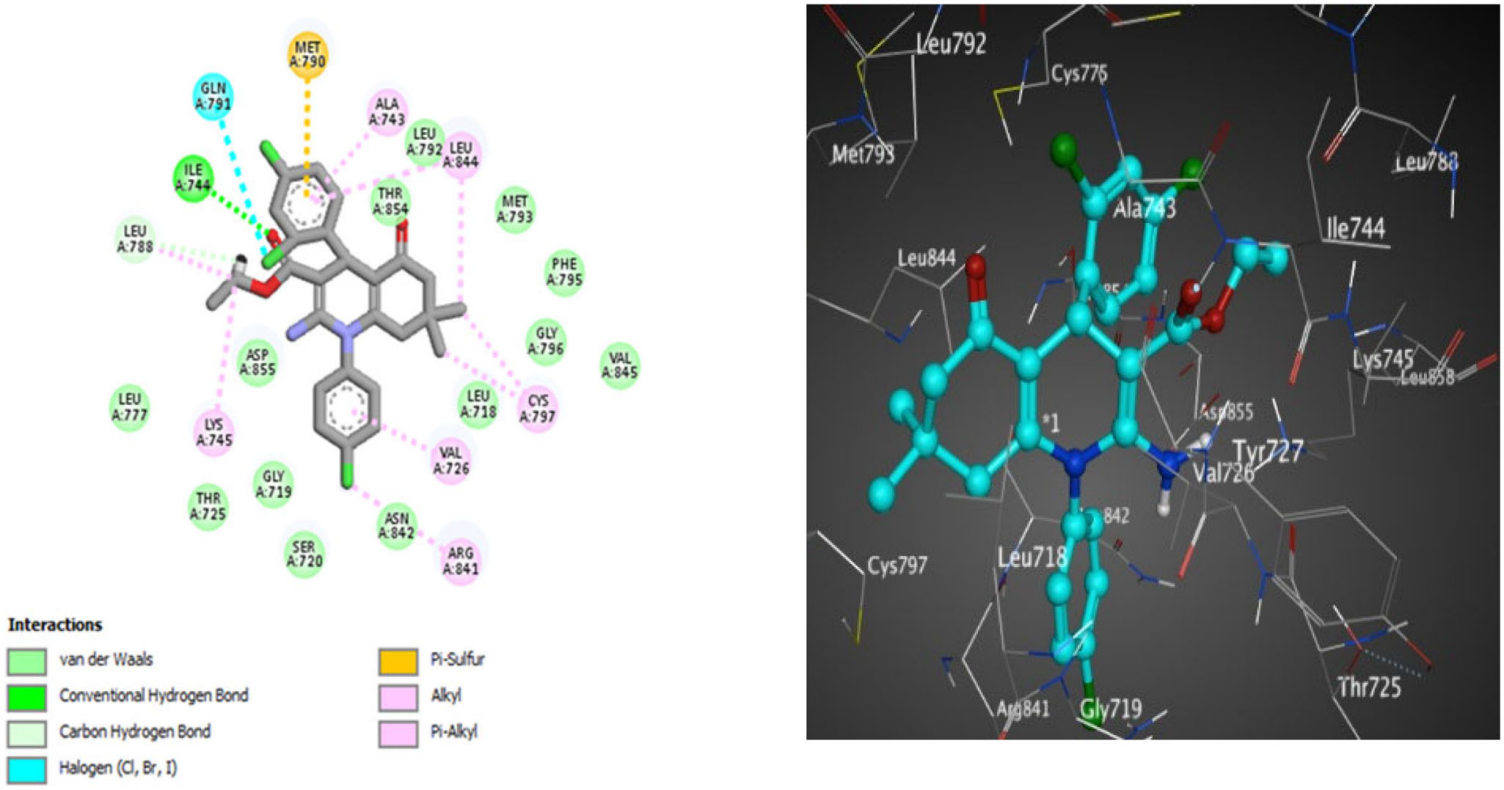
The 2D (left) and 3D (right) poses for docking interactions of the *S*-isomer of compound **10d** within the active site of EGFR (PDB code: 2JIV).

**Figure 14.**
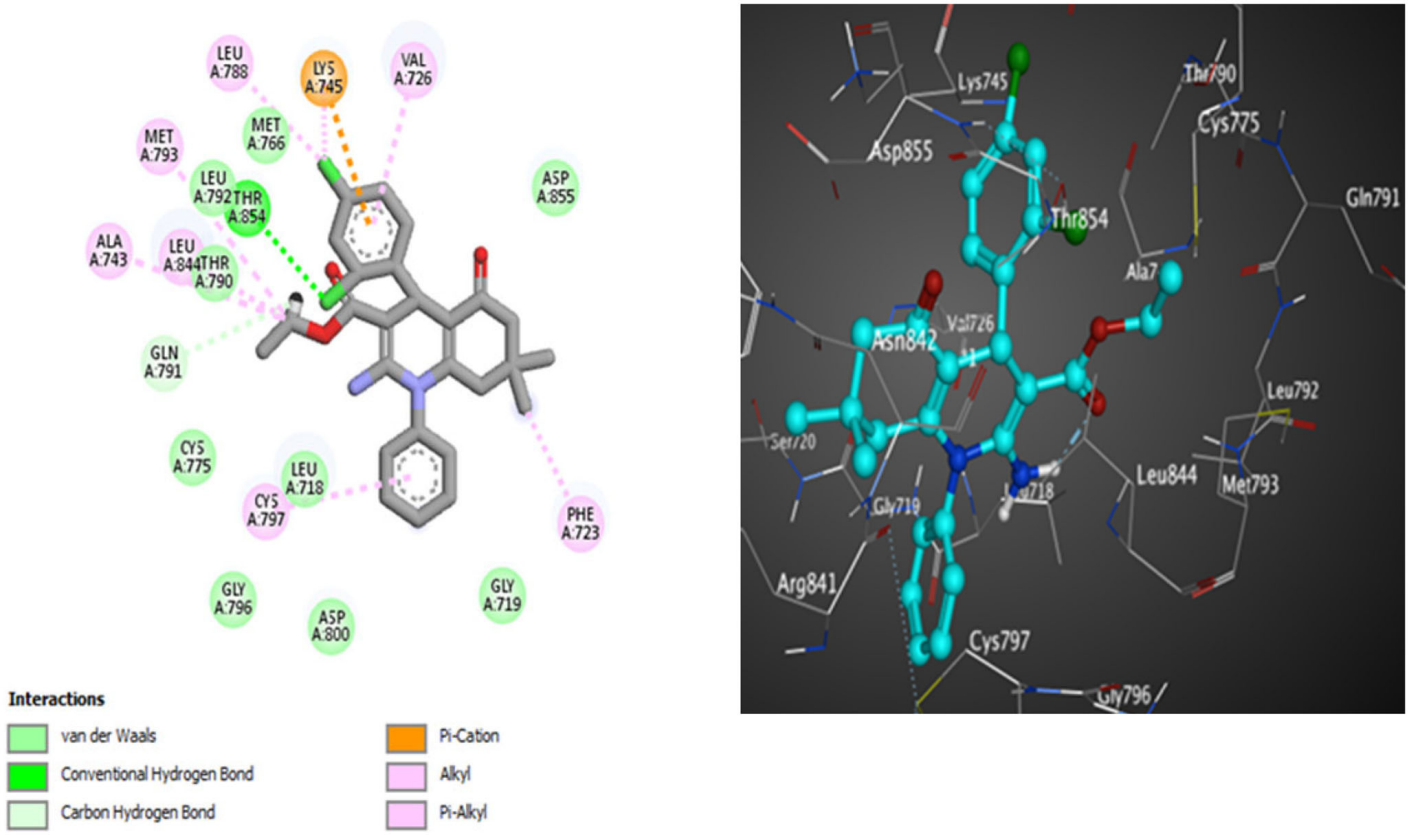
The 2D (left) and 3D (right) poses for docking interactions of the *S*-isomer of compound **10c** within the active site of EGFR (PDB code: 4LQM).

**Figure 15.**
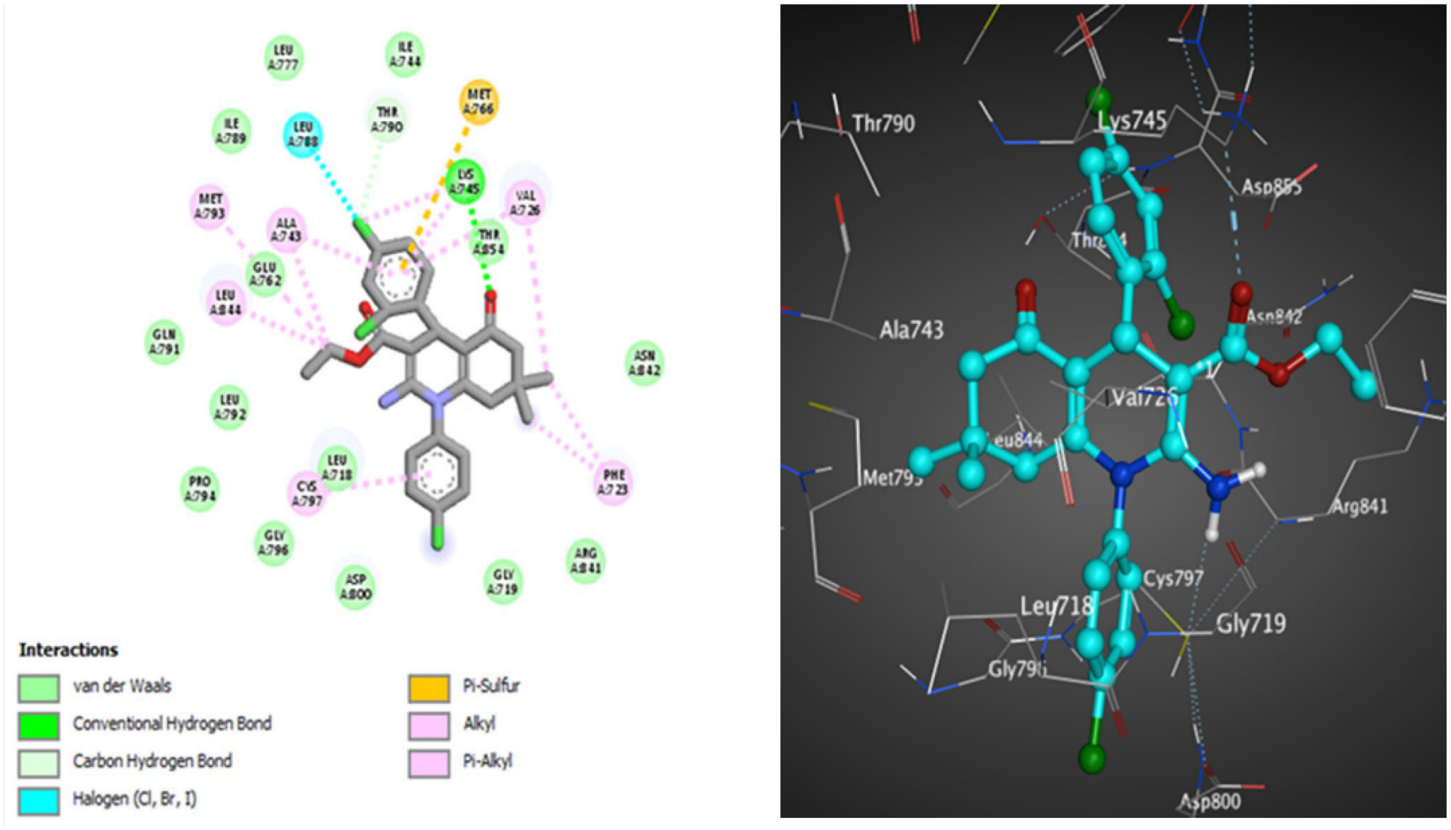
The 2D (left) and 3D (right) poses for docking interactions of the *S*-isomer of compound **10d** within the active site of EGFR (PDB code: 4LQM).

First, the original co-crystallised ligands, erlotinib, HKI, and PD-168393, were re-docked into the active sites of EGFR^WT^, EGFR^T790M^, and EGFR^L858R^ to validate the docking techniques, and the results were reported in (Table S3). Then our reference erlotinib and analogues **10c** and **10d** were docked into the active sites of EGFR^WT^, EGFR^T790M^, and EGFR^L858R^ enzymes. The docking results of erlotinib are provided in (Table S4). Fallouts of docking of the *R*- and *S*-enantiomers for analogues **10c** and **10d**, into the ATP-binding pocket of EGFR illustrated that *S*-enantiomer showed higher binding affinity than the *R*-enantiomer. This finding was also found for many other nuclei, such as tetrahydroisoquinoline^100^, thienopyrimidine^101^, pyrrolo[2,3-*d*]pyrimidine^102^, and dihydropyrimidine^103^. The alignment of *S*-enantiomers for compounds **10c** and **10d** with the co-crystallised ligands is displayed in (Figures S135–140). The *R*-enantiomer’s docking results for compounds **10c** and **10d** are detailed in (Tables S5 and S6) and (Figures S141–S146). Interactions of *S*-enantiomers for target compounds **10c** and **10d** are detailed in Figures 10–15 and Table 3.

**Table 3.** Fallouts of docking for *S*-isomers of compounds **10c** and **10d** within variant EGFRs.

Compound	PDB code (EGFR type)	Score (Kcal/mol)	Amino acids bind with		
			Hydrophobic head	HHQ core	Hydrophobic tail
**10c**	1M17 (wild type)	−7.67	Val702, Ala719, Leu764, Asp831	Phe699, Lys721*, Met769, Leu820	Cys773
**10d**		−7.73	Val702, Ala719, Met742 Leu764, Asp831	Phe699, Lys721*, Met769, Leu820	Cys773
**10c**	2JIV (T790M)	−6.65	Val726, Ala743, Lys745, Leu788, Asp855	Met790, Leu844, Cys797	Leu718, Val726
**10d**		−7.58	Ala743, Met790, Gln791	Ile744*, Lys745, Leu788, Cys797, Leu844	Arg841
**10c**	4LQM (L858R)	−6.90	Val726, Lys745, Leu788, Thr854*	Phe723, Ala743, Gln791, Met793, Leu844	Cys797
**10d**		−7.11	Val726, Ala743, Lys745, Met766, Leu788, Met790	Phe723, Lys745*, Met793, Leu844	Cys797

All amino acids make hydrophobic interactions except for those mentioned above (*) make hydrogen bonds.

For EGFR^WT^, the *S*-isomer of compound **10c** interacts through hydrogen bonding between the oxygen atom of C = O of the ester group and Lys721. It forms hydrophobic interactions with other amino acids in the active site, which are important for activity, such as Met769. Halogen bonding interactions are also reported between 2,4-dichlorine atoms and Asp831 and Leu764, respectively (Figure 10). Compound **10d** discloses hydrogen bonding interaction between the oxygen atom of C = O of the cyclohexanone ring and Lys721, in addition to forming many hydrophobic interactions with other amino acids in the active site, which are significant for activity such as Met769 and Met742. Halogen bonding interaction is also observed between the *ortho* chlorine atom and Asp831 (Figure 11).

Regarding EGFR^T790M^, docking results of compound **10c** exposed many hydrophobic attraction forces, but none of them with the amino acids that are important for activity, while compound **10d** formed a hydrogen bond with Ile744 and extra substantial hydrophobic interactions. Van der Waals attraction force is detected between one methyl group on the HHQ ring of compound **10c** and Cys797 (Figure 12), while for compound **10d,** the Van der Waals attraction force is observed between the two methyl groups on the HHQ ring and Cys797. Moreover, analogue **10d** demonstrates halogen bonding interaction between *ortho* chlorine atom on C4 phenyl and Gln791, giving a privilege for **10d** over **10c** (Figure 13).

Docking of compound **10c** into EGFR^L858R^ revealed that a hydrogen bond is reported between the *ortho* chlorine atom and Thr854. Van der Waals interaction between *N*-phenyl and Cys797 is detected. Compound **10d** forms a halogen bonding interaction between the *para* chlorine atom and Leu788. Van der Waals interaction between *N*-phenyl and Cys797 is identified (Figure 14). Besides, compound **10d** discloses additional hydrophobic interactions between the two methyl groups at the HHQ ring and Phe723 and Val725 (Figure 15). This docking study revealed that the number and type of interactions for compound **10d** are more effective than that for **10c**, which explains its stronger inhibitory activity towards wild and mutant EGFRs (EGFR^T790M^ and EGFR^L858R^).

As a conclusion for docking results, it is obvious that the halogenated inhibitors very efficiently displace the charged ATP ligand, mainly through halogen bonding interactions, stressing the potential role of halogen bonds in the design of new drugs and inhibitors.

## Conclusion

In this work, a series of thirty-two novel HHQ derivatives (in a racemic mixture) complying the pharmacophoric features for EGFR inhibitors were designed and synthesised. X-ray crystallography was performed to confirm configuration of compound **6f**. Compound **10c** showed very promising anticancer activities against 60 cancer cell line subpanels of the NCI. On the other hand, compound **10d** exhibited the most promising enzymatic inhibitory activity with IC_50_ values of 0.097 µM, 0.280 µM, and 0.051 µM, respectively, against the three variants of the enzyme, EGFR^WT^, EGFR^T790M^, and EGFR^L858R^. Furthermore, compounds **10c** and **10d** exhibited a total apoptosis percentage of 24.18 and 19.83%, respectively, more than that of the control (2.5%) by approximately 10- and 8-fold, respectively, towards HOP-92 lung cancer cells. Moreover, compounds **10c** and **10d** prompted cell cycle arrest in the pre-G1 phase in values of 25.82 and 21.55% and in the G2/M phase in values of 34.07 and 49.21%, respectively. It is worth mentioning that the biological data greatly matched the corresponding docking scores of the synthesised compounds. On the other hand, *in silico* studies were carried out and found that compound **10c** is more promising compound to be a drug than **10d** and has a privilege over erlotinib. Remarkably, compounds **10c** and **10d** could be considered promising lead compounds as EGFR inhibitors for further optimisation.

## Experimental

### Chemistry

Open-glass capillaries were used for melting point determination in a Stuart SMP30 apparatus and were uncorrected. The Sigma-Aldrich Company, and Merck company are the suppliers from which all organic reagents and solvents were purchased and were used without further purification. The progress of the reactions and the purity chick of the products was made using the developing system: ethyl acetate: *n*-hexane (5:2) as eluent and was visualised by exposure to UV lamp at *λ* 254 nm. ^1^H NMR and ^13^C NMR spectra were carried out using the Bruker instrument, having a frequency of 400 MHz for ^1^H NMR and 100 MHz for the ^13^C NMR spectrophotometer. Chemical shifts were recorded in ppm on the *δ* scale using CDCl_3_ or DMSO-d_6_ as solvents. Coupling constant (*J*) values were calculated in Hertz (Hz). Using a Thermo Scientific, ISQ Single Quadruple MS, the electron impact (EI) mass spectra were recorded. Microanalysis was performed for three elements, C, H, and N, to determine their percentages on PerkinElmer 2400 and was within ± 0.4% of theoretical values. All compounds were obtained as a racemic mixture (±) which was confirmed through x-ray crystallography and the optical rotations (α) of all synthesised compounds using a Polax-2L Polarimeter (ATAGO Co., Ltd., Saitama, Japan) where they failed to demonstrate any rotation. Several unsuccessful attempts for resolution of racemic mixtures of final products, such as crystallisation and HPLC chiral separation have been made.

#### General procedure for the synthesis of intermediates (3a–c)

A mixture of aniline derivatives (**1a–c**) (1.1 mmol) and dimedone (**2**) (1.0 mmol) was heated under reflux in DCM (20 ml) for 8 h using glacial acetic acid as catalyst (3 drops). After completing the reaction (checked by TLC), the solid powder was filtered off and washed with dist. water to afford yellow powders, **3a–c**. Intermediate **3a** reported m.p. = 180–182 °C (reported 181–183 °C^46^) intermediate **3b**, m.p. = 200–202 °C (reported 202–204 °C^47^), and intermediate **3c**, m.p. = 212–214 °C (reported 209–210 °C^104^).

#### General procedure for synthesis of the substituted 2-amino-1-phenyl-1,4,6,7,8-hexahydroquinolin-3-carboxylates (6a–i), (8a–m), (10a–d), and (12a–f)

A 50 ml round bottom flask, fitted with a reflux condenser, was charged with a mixture of **3a–c** (0.9 mmol), ethyl cyanoacetate **4** (1.2 mmol), appropriate aryl aldehyde; *o*-substituted benzaldehydes **5a–c**, *p*-substituted benzaldehydes **7a–e**, disubstituted benzaldehydes **9a,b,** or *o-* or *m*-pyridine carboxaldehydes **11a,b** (1.0 mmol) and a catalytic amount of piperidine (5 drops) in absolute ethanol (30 ml). The mixture was heated under reflux for 24 h. After completion of the reaction, the reaction mixture was cooled to room temperature, filtered off, and recrystallised from methanol to yield racemic mixture of products (**6a–i**), (**8a–m**), (**10a–d**), and (**12a–f**).

##### (±)-*Ethyl 2-amino-4–(2-chlorophenyl)-7,7-dimethyl-5-oxo-1-phenyl -1,4,5,6,7,8- hexahydroquinoline-3-carboxylate (6a)*

Off-white powder, yield: 34%; m.p. 168–170 °C. ^1^H NMR (400 MHz, DMSO-d_6_) *δ* (ppm): 0.70 (s, 3H, CH_3_-dimedone), 0.86 (s, 3H, CH_3_-dimedone), 1.08 (t, 3H, OCH_2_**CH_3_**, *J* = 8.0 Hz), 1.64 (d, 1H, COCH_2_, *J* = 16.0 Hz), 1.92 (d, 1H, COCH_2_, *J* = 16.0 Hz), 2.16 (d, 1H, **CH_2_**C(CH_3_)_2_, *J* = 16.0 Hz), 2.18 (d, 1H, **CH_2_**C(CH_3_)_2_, *J* = 16.0 Hz), 3.91–3.93 (m, 2H, O**CH_2_**CH_3_), 5.22 (s, 1H, HHQ, 4-H), 6.82 (*br*. s, 2H, NH_2_), 7.11 (t, 1H, Ar-H, *J* = 8.0 Hz), 7.24–7.27 (m, 2H, Ar-H), 7.46–7.50 (m, 3H, Ar-H), 7.61–7.67 (m, 3H, Ar-H), ^13^C NMR (100 MHz, CDCl_3_) *δ* (ppm): 14.38, 26.85, 28.35, 29.55, 32.23, 35.29, 42.09, 50.06, 59.24, 79.43, 113.09, 123.84, 125.80, 126.91, 129.39, 129.85, 130.04, 130.33, 133.16, 133.43, 136.45, 143.97, 149.81, 152.11, 170.19, 195.79; (+)ESI-MS (*m/z*): [M + H]^+^: 451.20, Anal Calcd. for C_26_H_27_ClN_2_O_3_: C; 69.25, H; 6.04, N; 6.21. Found: C; 69.01, H; 6.21, N; 6.05.

##### (±)-Ethyl 2-amino-4–(2-chlorophenyl)-7,7-dimethyl-1–(4-methylphenyl)-5-oxo-1,4,5,6,7,8-hexahydroquinoline-3-carboxylate (6b)

Off-White crystals, yield: 67%; m.p. 240–242 °C. ^1^H NMR (400 MHz, CDCl_3_) *δ* (ppm): 0.84 (s, 3H, CH_3_-dimedone), 0.95 (s, 3H, CH_3_-dimedone), 1.18 (t, 3H, OCH_2_**CH_3_**, *J* = 8.0 Hz), 1.81 (d, 1H, COCH_2_, *J* = 16.0 Hz), 2.02 (d, 1H, COCH_2_, *J* = 16.0 Hz), 2.09 (d, 1H, **CH_2_**C(CH_3_)_2_, *J* = 16.0 Hz), 2.18 (d, 1H, **CH_2_**C(CH_3_)_2_, *J* = 16.0 Hz), 2.49 (s, 3H, CH_3_), 3.96–4.10 (m, 2H, O**CH_2_**CH_3_), 5.39 (s, 1H, HHQ, 4-H), 6.31 (*br*. s, 2H, NH_2_), 7.06 (t, 1H, Ar-H, *J* = 8.0 Hz), 7.16–7.29 (m, 4H, Ar-H), 7.39 (d, 2H, Ar-H, *J* = 8.0 Hz), 7.56 (d, 1H, Ar-H, *J* = 8.0 Hz), ^13^C NMR (100 MHz, CDCl_3_) *δ* (ppm): 14.40, 21.31, 26.85, 29.59, 32.23, 35.22, 42.04, 50.06, 59.22, 79.31, 113.02, 125.81, 126.88, 129.83, 129.94, 131.02, 133.12, 133.42, 133.60, 140.31, 144.08, 150.14, 152.31, 170.21, 195.84; (+)ESI-MS (*m/z*): [M + H]^+^: 465.20, Anal Calcd. for C_27_H_29_ClN_2_O_3_: C; 69.74, H; 6.29, N; 6.02. Found: C; 69.98, H; 6.52, N; 6.30.

##### (±)-Ethyl 2-amino-4–(2-chlorophenyl)-1–(4-chlorophenyl)-7,7-dimethyl-5-oxo-1,4,5,6,7,8-hexahydroquinoline-3-carboxylate (6c)

Off-white powder, yield: 87%; m.p. 179–181 °C. ^1^H NMR (400 MHz, CDCl_3_) *δ* (ppm): 0.84 (s, 3H, CH_3_-dimedone), 0.96 (s, 3H, CH_3_-dimedone), 1.17 (t, 3H, OCH_2_**CH_3_**, *J* = 8.0 Hz), 1.78 (d, 1H, COCH_2_, *J* = 16.0 Hz), 2.01 (d, 1H, COCH_2_, *J* = 16.0 Hz), 2.10 (d, 1H, **CH_2_**C(CH_3_)_2_, *J* = 16.0 Hz), 2.18 (d, 1H, **CH_2_**C(CH_3_)_2_, *J* = 16.0 Hz), 3.98–4.10 (m, 2H, O**CH_2_**CH_3_), 5.37 (s, 1H, HHQ, 4-H), 6.25 (*br*. s, 2H, NH_2_), 7.06 (*t*, 1H, Ar-H, *J* = 8.0 Hz), 7.18 (t, 1H, Ar-H, *J* = 8.0 Hz), 7.25–7.28 (m, 1H, Ar-H), 7.33 (d, 2H, Ar-H, *J* = 8.0 Hz), 7.54–7.61 (m, 3H, Ar-H), ^13^C NMR (100 MHz, CDCl_3_) δ (ppm): 14.37, 26.85, 29.62, 32.29, 35.38, 42.15, 49.99, 59.35, 79.63, 113.21, 125.84, 127.04, 129.92, 130.74, 131.69, 133.29, 133.41, 134.91, 136.24, 143.63, 149.41, 151.80, 170.13, 195.79; (+)ESI-MS (*m/z*): [M + H]^+^: 484.81, Anal Calcd. for C_26_H_26_Cl_2_N_2_O_3_: C; 64.33, H; 5.40, N; 5.77. Found: C; 64.51, H; 5.61, N; 6.00.

##### (±)-Ethyl 2-amino-4–(2-bromophenyl)-7,7-dimethyl-5-oxo-1-phenyl-1,4,5,6,7,8-hexahydroquinoline-3-carboxylate (6d)

Yellow powder, yield: 53%; m.p. 220–222 °C. ^1^H NMR (400 MHz, DMSO-d_6_) *δ* (ppm): 0.70 (s, 3H, CH_3_-dimedone), 0.85 (s, 3H, CH_3_-dimedone), 1.07 (t, 3H, OCH_2_**CH_3_**, *J* = 8.0 Hz), 1.64 (d, 1H, COCH_2_, *J* = 16.0 Hz), 1.91 (d, 1H, COCH_2_, *J* = 16.0 Hz), 2.15 (d, 1H, **CH_2_**C(CH_3_)_2_, *J* = 16.0 Hz), 2.17 (d, 1H, **CH_2_**C(CH_3_)_2_, *J* = 16.0 Hz), 3.93–4.01 (m, 2H, O**CH_2_**CH_3_), 5.19 (s, 1H, HHQ, 4-H), 6.80 (*br*. s, 2H, NH_2_), 7.01 (t, 1H, Ar-H, *J* = 8.0 Hz), 7.30 (t, 1H, Ar-H, *J* = 8.0 Hz), 7.43 (d, 1H, Ar-H, *J* = 8.0 Hz), 7.49 (d, 3H, Ar-H, *J* = 8.0 Hz), 7.64 (d, 3H, Ar-H, *J* = 8.0 Hz), ^13^C NMR (100 MHz, CDCl_3_) *δ* (ppm): 14.57, 27.01, 29.49, 32.27, 36.73, 42.13, 50.07, 59.24, 80.07, 113.74, 123.46, 126.56, 126.96, 127.12, 130.11, 130.31, 132.80, 133.17, 136.40, 146.03, 149.62, 152.03, 170.24, 195.85; (+)ESI-MS (*m/z*): [M + H]^+^: 495.30, Anal Calcd. for C_26_H_27_BrN_2_O_3_: C; 63.03, H; 5.49, N; 5.65. Found: C; 63.22, H; 5.34, N; 5.48.

##### (±)-Ethyl 2-amino-4–(2-bromophenyl)-7,7-dimethyl-1–(4-methylphenyl)-5-oxo-1,4,5,6,7,8-hexahydroquinoline-3-carboxylate (6e)

White powder, yield: 56%; m.p. 236–238 °C. ^1^H NMR (400 MHz, CDCl_3_) *δ* (ppm): 0.83 (s, 3H, CH_3_-dimedone), 0.94 (s, 3H, CH_3_-dimedone), 1.17 (t, 3H, OCH_2_**CH_3_**, *J* = 8.0 Hz), 1.82 (d, 1H, COCH_2_, *J* = 16.0 Hz), 2.01 (d, 1H, COCH_2_, *J* = 16.0 Hz), 2.09 (d, 1H, **CH_2_**C(CH_3_)_2_, *J* = 16.0 Hz), 2.17 (d, 1H, **CH_2_**C(CH_3_)_2_, *J* = 16.0 Hz), 2.49 (s, 3H, CH_3_), 4.00–4.13 (m, 2H, O**CH_2_**CH_3_), 5.39 (s, 1H, HHQ, 4-H), 6.32 (*br*. s, 2H, NH_2_), 6.96 (t, 1H, Ar-H, *J* = 8.0 Hz), 7.20–7.23 (m, 3H, Ar-H), 7.39 (d, 2H, Ar-H, *J* = 8.0 Hz), 7.47 (d, 1H, Ar-H, *J* = 8.0 Hz), 7.53 (d, 1H, Ar-H, *J* = 8.0 Hz), ^13^C NMR (100 MHz, CDCl_3_) δ (ppm): 14.58, 21.32, 26.99, 29.50, 32.23, 36.66, 42.08, 50.07, 59.18, 79.97, 113.67, 123.45, 126.56, 127.06, 127.69, 129.89, 132.72, 133.11, 133.54, 140.36, 146.17, 149.97, 152.22, 170.22, 195.87; (+)ESI-MS (*m/z*): [M + H]^+^: 509.10, Anal Calcd. for C_27_H_29_BrN_2_O_3_: C; 63.66, H; 5.74, N; 5.50. Found: C; 63.44, H; 5.53, N; 5.71.

##### (±)-Ethyl 2-amino-4–(2-bromophenyl)-1–(4-chlorophenyl)-7,7-dimethyl-5-oxo-1,4,5,6,7,8-hexahydroquinoline-3-carboxylate (6f)

Off-white crystals, yield: 92%; m.p. 210–212 °C. ^1^H NMR (400 MHz, CDCl_3_) *δ* (ppm): 0.69 (s, 3H, CH_3_-dimedone), 0.88 (s, 3H, CH_3_-dimedone), 1.14 (t, 3H, OCH_2_**CH_3_**, *J* = 8.0 Hz), 1.65 (d, 1H, COCH_2_, *J* = 16.0 Hz), 2.00 (d, 1H, COCH_2_, *J* = 16.0 Hz), 2.21 (d, 1H, **CH_2_**C(CH_3_)_2_, *J* = 16.0 Hz), 2.24 (d, 1H, **CH_2_**C(CH_3_)_2_, *J* = 16.0 Hz), 3.94–4.02 (m, 2H, O**CH_2_**CH_3_), 4.91 (s, 1H, HHQ, 4-H), 6.80 (*br*. s, 2H, NH_2_), 7.18–7.20 (m, 1H, Ar-H), 7.25–7.34 (m, 3H, Ar-H), 7.42 (d, 2H, Ar-H, *J* = 8.0 Hz), 7.59–7.67 (m, 2H, Ar-H), ^13^C NMR (100 MHz, CDCl_3_) *δ* (ppm): 14.52, 27.02, 28.33, 29.52, 32.29, 36.81, 42.21, 50.02, 59.33, 80.33, 113.89, 125.06, 126.55, 127.22, 129.47, 131.66, 132.91, 133.22, 134.89, 136.29, 145.69, 149.20, 151.70, 170.15, 195.80; (+)ESI-MS *(m/z*): [M + H]^+^: 529.00, Anal Calcd. for C_26_H_26_BrClN_2_O_3_: C; 58.94, H; 4.95, N; 5.29. Found: C; 59.10, H; 5.21, N; 5.12.

##### (±)-Ethyl 2-amino-4–(2-methoxyphenyl)-7,7-dimethyl-5-oxo-1-phenyl-1,4,5,6,7,8-hexahydroquinoline-3-carboxylate (6g)

Yellow powder, yield: 33%; m.p. 243–245 °C. ^1^H NMR (400 MHz, CDCl_3_) *δ* (ppm): 0.80 (s, 3H, CH_3_-dimedone), 0.93 (s, 3H, CH_3_-dimedone), 1.20 (t, 3H, OCH_2_**CH_3_**, *J* = 8.0 Hz), 1.75 (d, 1H, COCH_2_, *J* = 16.0 Hz), 2.02 (d, 1H, COCH_2_, *J* = 16.0 Hz), 2.08 (d, 1H, **CH_2_**C(CH_3_)_2_, *J* = 16.0 Hz), 2.13 (d, 1H, **CH_2_**C(CH_3_)_2_, *J* = 16.0 Hz), 3.95 (s, 3H, OCH_3_), 4.03–4.06 (m, 2H, O**CH_2_**CH_3_), 5.28 (s, 1H, HHQ, 4-H), 6.21 (*br*. s, 2H, NH_2_), 6.83–6.93 (m, 3H, Ar-H), 7.09–7.13 (m, 3H, Ar-H), 7.39–7.47 (m, 3H, Ar-H), ^13^C NMR (100 MHz, CDCl_3_) *δ* (ppm): 14.36, 26.68, 29.72, 32.36, 33.21, 41.20, 42.24, 50.16, 55.91, 59.11, 79.94, 111.55, 113.79, 119.91, 120.27, 126.89, 129.82, 131.37, 132.07, 134.73, 136.91, 149.31, 151.96, 170.51, 195.83; (+)ESI-MS (*m/z*): [M + H]^+^: 447.50, Anal Calcd. for C_27_H_30_N_2_O_4_: C; 72.62, H; 6.77, N; 6.27. Found: C; 72.48, H; 7.00, N; 6.08.

##### (±)-Ethyl 2-amino-4–(2-methoxyphenyl)-7,7-dimethyl-1–(4-methylphenyl)-5-oxo-1,4,5,6,7,8-hexahydroquinoline-3-carboxylate (6h)

Yellow powder, yield: 63%; m.p. 181–183 °C. ^1^H NMR (400 MHz, CDCl_3_) *δ* (ppm): 0.78 (s, 3H, CH_3_-dimedone), 0.96 (s, 3H, CH_3_-dimedone), 1.19 (t, 3H, OCH_2_**CH_3_**, *J* = 8.0 Hz), 1.80 (d, 1H, COCH_2_, *J* = 16.0 Hz), 2.07 (d, 1H, COCH_2_, *J* = 16.0 Hz), 2.11 (d, 1H, **CH_2_**C(CH_3_)_2_, *J* = 16.0 Hz), 2.21 (d, 1H, **CH_2_**C(CH_3_)_2_, *J* = 16.0 Hz), 2.48 (s, 3H, CH_3_), 3.88 (s, 3H, OCH_3_), 4.02–4.07 (m, 2H, O**CH_2_**CH_3_), 5.11 (s, 1H, HHQ, 4-H), 6.31 (*br*. s, 2H, NH_2_), 7.20–7.23 (m, 3H, Ar-H), 7.39 (d, 2H, Ar-H, *J* = 8.0 Hz), 7.81 (d, 1H, Ar-H, *J* = 8.0 Hz), 8.36–8.38 (m, 1H, Ar-H), 8.65 (s, 1H, Ar-H),^13^C NMR (100 MHz, CDCl_3_) *δ* (ppm): 14.44, 21.32, 27.00, 29.39, 32.46, 33.30, 41.83, 49.95, 59.45, 79.40, 113.76, 123.44, 129.64, 131.36, 133.00, 138.56, 140.69, 144.14, 144.98, 146.85, 150.34, 152.11, 169.37, 195.74; (+)ESI-MS (*m/z*): [M + Na]^+^: 483.20, Anal Calcd. for C_28_H_32_N_2_O_4_: C; 73.02, H; 7.00, N; 6.08. Found: C; 73.22, H; 7.25, N; 6.24.

##### (±)-Ethyl 2-amino-1–(4-chlorophenyl)-4–(2-methoxyphenyl)-7,7-dimethyl-5-oxo-1,4,5,6,7,8-hexahydroquinoline-3-carboxylate (6i)

Yellow powder, yield: 46%; m.p. 228–230 °C. ^1^H NMR (400 MHz, CDCl_3_) *δ* (ppm): 0.79 (s, 3H, CH_3_-dimedone), 0.97 (s, 3H, CH_3_-dimedone), 1.22 (t, 3H, OCH_2_**CH_3_**, *J* = 8.0 Hz), 1.74 (d, 1H, COCH_2_, *J* = 16.0 Hz), 2.05 (d, 1H, COCH_2_, *J* = 16.0 Hz), 2.12 (d, 1H, **CH_2_**C(CH_3_)_2_, *J* = 16.0 Hz), 2.17 (d, 1H, **CH_2_**C(CH_3_)_2_, *J* = 16.0 Hz), 3.78 (s, 3H, OCH_3_), 4.05–4.09 (m, 2H, O**CH_2_**CH_3_), 5.09 (s, 1H, HHQ, 4-H), 6.17 (*br*. s, 2H, NH_2_), 6.80 (d, 2H, Ar-H, *J* = 8.0 Hz), 7.30 (t, 4H, Ar-H, *J* = 8.0 Hz), 7.57 (d, 2H, Ar-H, *J* = 8.0 Hz), ^13^C NMR (100 MHz, CDCl_3_) *δ* (ppm): 14.44, 26.73, 29.71, 32.46, 33.29, 41.90, 50.12, 55.16, 59.38, 81.68, 113.22, 116.03, 128.64, 130.75, 131.43, 135.09, 136.15, 140.03, 148.38, 151.32, 157.56, 170.03, 195.93; (+)ESI-MS (*m/z*): [M + H]^+^: 481.52, Anal Calcd. for C_27_H_29_ClN_2_O_4_: C; 67.42, H; 6.08, N; 5.82. Found: C; 67.20, H; 6.30, N; 6.09.

##### (±)-Ethyl 2-amino-4–(4-fluorophenyl)-7,7-dimethyl-5-oxo-1-phenyl -1,4,5,6,7,8-hexahydroquinoline -3- carboxylate (8a)

Faint orange powder, yield: 94%; m.p. 215–217 °C. ^1^H NMR (400 MHz, CDCl_3_) *δ* (ppm): 0.78 (s, 3H, CH_3_-dimedone), 0.95 (s, 3H, CH_3_-dimedone), 1.21 (t, 3H, OCH_2_**CH_3_**, *J* = 8.0 Hz), 1.74 (d, 1H, COCH_2_, *J* = 16.0 Hz), 2.06 (d, 1H, COCH_2_, *J* = 8.0 Hz), 2.11 (d, 1H, **CH_2_**C(CH_3_)_2_, *J* = 16.0 Hz), 2.20 (d, 1H, **CH_2_**C(CH_3_)_2_, *J* = 16.0 Hz), 4.06 (q, 2H, O**CH_2_**CH_3_, *J* = 8.0 Hz), 5.13 (s, 1H, HHQ, 4-H), 6.24 (*br*. s, 2H, NH_2_), 7.20–7.23 (m, 2H, Ar-H), 7.33–7.37 (m, 4H, Ar-H), 7.60 (s, 3H, Ar-H), ^13^C NMR (100 MHz, CDCl_3_) *δ* (ppm): 14.42, 23.82, 25.44, 26.61, 26.77, 29.65, 32.39, 33.34, 33.68, 41.84, 50.14, 59.28, 81.26, 114.34, 114.55, 128.47, 129.19, 130.04, 130.51, 136.58, 143.71, 149.07, 151.67, 169.93, 170.05, 195.87; (+)ESI-MS (*m/z*): [M + H]^+^: 435.20, Anal Calcd. for C_26_H_27_FN_2_O_3_: C; 71.87, H; 6.26, N; 6.45. Found: C; 71.56, H; 6.02, N; 6.12.

##### (±)-Ethyl 2-amino-1–(4-chlorophenyl)-4–(4-fluoroyphenyl)-7,7-dimethyl-5-oxo-1,4,5,6,7,8-hexahydroquinoline-3-carboxylate (8b)

Shiny yellow powder, Yield: 72%; m.p. 256–258 °C. ^1^H NMR (400 MHz, CDCl_3_) *δ* (ppm): 0.78 (s, 3H, CH_3_-dimedone), 0.97 (s, 3H, CH_3_-dimedone), 1.20 (t, 3H, OCH_2_**CH_3_**, *J* = 8.0 Hz), 1.74 (d, 1H, COCH_2,_ *J* = 16.0 Hz), 2.06 (d, 1H, COCH_2_, *J* = 16.0 Hz), 2.14 (d, 1H, **CH_2_**C(CH_3_)_2_, *J* = 16.0 Hz), 2.22 (d, 1H, **CH_2_**C(CH_3_)_2_, *J* = 16.0 Hz), 4.04–4.11 (m, 2H, O**CH_2_**CH_3_), 5.12 (s, 1H, HHQ, 4-H), 6.21 (*br*. s, 2H, NH_2_), 6.94 (t, 2H, Ar-H, *J* = 8.0 Hz), 7.29 (t, 2H, Ar-H, *J* = 8.0 Hz), 7.33–7.37 (m, 2H, Ar-H), 7.59 (d, 2H, Ar-H, *J* = 8.0 Hz),^13^C NMR (100 MHz, CDCl_3_) *δ* (ppm): 14.40, 26.63, 29.69, 32.45, 33.64, 41.93, 50.07, 59.41, 81.34, 114.41, 114.62, 115.71, 129.10, 129.17, 130.82, 131.38, 134.92, 136.29, 143.43, 143.46, 148.56, 151.39, 159.90, 162.32, 169.89, 195.84; (+)ESI-MS (*m/z*): [M + Na] ^+^: 491.30, Anal Calcd for C_26_H_26_ClFN_2_O_3_: C; 66.59, H; 5.59, N; 5.97, Found: C; 66.71, H; 5.91, N; 6.10.

##### (±)-Ethyl 2-amino-4–(4-chlorophenyl)-7,7-dimethyl-5-oxo-1-phenyl -1,4,5,6,7,8-hexahydroquinoline-3-carboxylate (8c)

Yellow powder, yield: 76%; m.p. 215–217 °C. ^1^H NMR (400 MHz, CDCl_3_) *δ* (ppm): 0.77 (s, 3H, CH_3_-dimedone), 0.95 (s, 3H, CH_3_-dimedone), 1.20 (t, 3H, OCH_2_**CH_3_**, *J* = 8.0 Hz), 1.74 (s, 1H, COCH_2_), 2.06 (d, 1H, COCH_2_, *J* = 16.0 Hz), 2.13 (d, 1H, **CH_2_**C(CH_3_)_2_, *J* = 16.0 Hz), 2.21 (d, 1H, **CH_2_**C(CH_3_)_2_, *J* = 16.0 Hz), 4.02–4.10 (m, 2H, O**CH_2_**CH_3_), 5.13 (s, 1H, HHQ, 4-H), 6.24 (*br*. s, 2H, NH_2_), 7.22 (d, 2H, Ar-H, *J* = 8.0 Hz), 7.31–7.37 (m, 4H, Ar-H), 7.60 (d, 3H, Ar-H, *J* = 8.0 Hz), ^13^C NMR (100 MHz, CDCl_3_) *δ* (ppm): 14.43, 26.84, 29.22, 29.66, 32.58, 33.16, 33.88, 41.77, 50.11, 59.35, 63.18, 80.62, 115.14, 127.51, 128.12, 129.20, 130.00, 133.96, 134.93, 136.34, 146.40, 149.23, 151.79, 166.50, 169.88, 195.85; (+)ESI-MS (*m/z*): [M + H]^+^: 451.00, Anal Calcd. for C_26_H_27_ClN_2_O_3_: C; 69.25, H; 6.03, N; 6.21. Found: C; 69.51, H; 6.03, N; 6.02.

##### (±)-Ethyl 2-amino-4–(4-chlorophenyl)-7,7-dimethyl-1–(4-methylphenyl)-5-oxo-1,4,5,6,7,8-hexahydroquinoline-3-carboxylate (8d)

Yellow powder, yield: 87%; m.p. 205–207 °C. ^1^H NMR (400 MHz, CDCl_3_) *δ* (ppm): 0.77 (s, 3H, CH_3_-dimedone), 0.96 (s, 3H, CH_3_-dimedone), 1.21 (t, 3H, OCH_2_**CH_3_**, *J* = 8.0 Hz), 1.77 (d, 1H, COCH_2_, *J* = 16.0 Hz), 2.06 (d, 1H, COCH_2_, *J* = 16.0 Hz), 2.13 (d, 1H, **CH_2_**C(CH_3_)_2_, *J* = 16.0 Hz), 2.21 (d, 1H, **CH_2_**C(CH_3_)_2_, *J* = 16.0 Hz), 2.49 (s, 3H, CH_3_), 4.04–4.08 (m, 2H, O**CH_2_**CH_3_), 5.12 (s, 1H, HHQ, 4-H), 6.28 (*br*. s, 2H, NH_2_), 7.18–7.25 (m, 5H, Ar-H), 7.34–7.40 (m, 3H, Ar-H,), ^13^C NMR (100 MHz, CDCl_3_) *δ* (ppm): 14.44, 21.31, 26.64, 29.68, 32.38, 33.90, 41.84, 50.12, 59.30, 80.51, 115.02, 127.89, 128.94, 129.22, 129.62, 131.11, 133.51, 140.41, 146.50, 149.55, 151.98, 169.90, 195.87; (+)ESI-MS (*m/z*): [M + H]^+^: 465.30, Anal Calcd. for C_27_H_29_ClN_2_O_3_: C; 69.74, H; 6.29, N; 6.02. Found: C; 70.00, H; 6.44, N; 6.31.

##### (±)-Ethyl 2-amino-1,4-bis (4-chlorophenyl)-7,7-dimethyl-5-oxo-1,4,5,6,7,8-hexahydroquinoline-3-carboxylate (8e)

Off-white powder, yield: 66%; m.p. 220–222 °C. ^1^H NMR (400 MHz, CDCl_3_) *δ* (ppm): 0.78 (s, 3H, CH_3_-dimedone), 0.97 (s, 3H, CH_3_-dimedone), 1.22 (t, 3H, OCH_2_**CH_3_**, *J* = 8.0 Hz), 1.74 (d, 1H, COCH_2_, *J* = 16.0 Hz), 2.07 (d, 1H, COCH_2_, *J* = 16.0 Hz), 2.14 (d, 1H, **CH_2_**C(CH_3_)_2_, *J* = 16.0 Hz), 2.22 (d, 1H, **CH_2_**C(CH_3_)_2_, *J* = 16.0 Hz), 4.00–4.10 (m, 2H, O**CH_2_**CH_3_), 5.11 (s, 1H, HHQ, 4-H), 6.21 (*br*. s, 2H, NH_2_), 7.22 (d, 2H, Ar-H, *J* = 8.0 Hz), 7.27–7.29 (m, 2H, Ar-H), 7.33 (d, 2H, Ar-H, *J* = 8.0 Hz), 7.59 (d, 2H, Ar-H, *J* = 8.0 Hz), ^13^C NMR (100 MHz, CDCl_3_) *δ* (ppm): 14.41, 26.65, 29.70, 32.45, 33.85, 41.94, 50.05, 58.48, 59.46, 80.99, 115.44, 127.97, 129.62, 131.27, 131.94, 134.84, 136.34, 146.16, 148.74, 151.46, 169.83, 195.81; (+)ESI-MS (*m/z*): [M + Na]^+^: 507.50, Anal Calcd. for C_26_H_26_Cl_2_N_2_O_3_: C; 64.33, H; 5.40, N; 5.77. Found: C; 64.57, H; 5.10, N; 6.04.

##### (±)-Ethyl2-amino-4–(4-methoxyphenyl)-7,7-dimethyl-5-oxo-1-phenyl-1,4,5,6,7,8-hexahydroquinoline-3-carboxylate (8f)

Shiny yellow powder, Yield: 43%; m.p. 169–171 °C. ^1^H NMR (400 MHz, CDCl_3_) *δ* (ppm): 0.54 (s, 3H, CH_3_-dimedone), 0.70 (s, 3H, CH_3_-dimedone), 0.98 (t, 3H, OCH_2_**CH_3_**, *J* = 8.0 Hz,), 1.50 (d, 1H, COCH_2_, *J* = 16.0 Hz), 1.82 (d, 1H, COCH_2_, *J* = 16.0 Hz), 1.88 (d, 1H, **CH_2_**C(CH_3_)_2_, *J* = 16.0 Hz), 1.97 (d, 1H, **CH_2_**C(CH_3_)_2_, *J* = 16.0 Hz), 3.53 (s, 3H, OCH_3_), 3.78–3.87 (m, 2H, O**CH_2_**CH_3_), 4.86 (s, 1H, HHQ, 4-H), 5.97 (br. s, 2H, NH_2_), 6.56 (d, 2H, Ar-H, *J* = 8.0 Hz); 7.10 (d, 4H, Ar-H, *J* = 8.0 Hz), 7.32–7.37 (m, 3H, Ar-H), ^13^C NMR (100 MHz, CDCl_3_) *δ* (ppm): 14.48, 26.70, 29.68, 32.39, 33.34, 35.12, 41.83, 50.18, 55.15, 59.23, 81.27, 113.20, 115.72, 128.68, 130.01, 129.82, 131.32, 136.54, 140.32, 148.94, 151.70, 157.52, 170.06, 195.52; (+)ESI-MS (*m/z*): [M + H] ^+^: 447.20, Anal Calcd for C_27_H_30_N_2_O_4_: C; 72.62, H; 6.77, N; 6.27; Found: C; 72.88, H; 6.95, N; 6.15.

##### (±)-Ethyl 2-amino-4–(4-methoxyphenyl)-7,7-dimethyl-1–(4-methylphenyl)-5-oxo-1,4,5,6,7,8-hexahydroquinoline-3-carboxylate (8g)

Yellow powder, yield: 63%; m.p. 181–183 °C. ^1^H NMR (400 MHz, CDCl_3_) *δ* (ppm): 0.53 (s, 3H, CH_3_-dimedone), 0.70 (s, 3H, CH_3_-dimedone), 0.97 (t, 3H, OCH_2_**CH_3_**, *J* = 8.0 Hz), 1.52 (d, 1H, COCH_2_, *J* = 16.0 Hz), 1.81 (d, 1H, COCH_2_, *J* = 16.0 Hz), 1.87 (d, 1H, **CH_2_**C(CH_3_)_2_, *J* = 16.0 Hz), 1.95 (d, 1H, **CH_2_**C(CH_3_)_2_, *J* = 16.0 Hz), 2.22 (s, 3H, CH_3_), 3.53 (s, 3H, OCH_3_), 3.76–3.85 (m, 2H, O**CH_2_**CH_3_), 4.84 (s, 1H, HHQ, 4-H), 5.98 (*br*. s, 2H, NH_2_), 6.55 (d, 2H, Ar-H, *J* = 8.0 Hz), 6.95 (d, 2H, Ar-H, *J* = 8.0 Hz), 7.08 (d, 2H, Ar-H, *J* = 8.0 Hz), 7.12 (d, 2H, Ar-H, *J* = 8.0 Hz), ^13^C NMR (100 MHz, CDCl_3_) *δ* (ppm): 14.48, 21.30, 26.71, 29.69, 32.38, 33.34, 41.81, 50.19, 55.27, 59.19, 81.15, 113.17, 115.61, 128.68, 129.67, 131.08, 133.73, 140.23, 140.42, 149.20, 151.88, 157.49, 170.07, 195.65; (+)ESI-MS (*m/z*): [M + Na]^+^: 483.20, Anal Calcd. for C_28_H_32_N_2_O_4_: C; 73.02, H; 7.00, N; 6.08. Found: C; 73.22, H; 7.25, N; 6.24.

##### (±)-Ethyl 2-amino-1–(4-chlorophenyl)-4–(4-methoxyphenyl)-7,7-dimethyl-5-oxo-1,4,5,6,7,8-hexahydroquinoline-3-carboxylate (8h)

Shiny yellow powder, Yield: 85%; m.p. 255–257 °C. ^1^H NMR (400 MHz, CDCl_3_) *δ* (ppm): 0.79 (s, 3H, CH_3_-dimedone), 0.96 (s, 3H, CH_3_-dimedone), 1.22 (t, 3H OCH_2_**CH_3_**, *J* = 8.0 Hz), 1.74 (d, 1H, COCH_2_, *J* = 16.0 Hz), 2.05 (d, 1H, COCH_2,_ *J* = 16.0 Hz), 2.13 (d, 1H, **CH_2_**C(CH_3_)_2_, *J* = 16.0 Hz), 2.21 (d, 1H, **CH_2_**C(CH_3_)_2_, *J* = 16.0 Hz), 3.77 (s, 3H, OCH_3_), 4.00–4.09 (m, 2H, O**CH_2_**CH_3_), 5.08 (s, 1H, HHQ, 4-H), 6.17 (*br*. s, 2H, NH_2_), 6.80 (d, 2H, Ar-H, *J* = 8.0 Hz), 7.30 (t, 4H, Ar-H, *J* = 8.0 Hz), 7.57 (d, 2H, Ar-H, *J* = 8.0 Hz),^13^C NMR (100 MHz, CDCl_3_) *δ* (ppm): 14.44, 26.74, 29.70, 32.45, 33.29, 41.91, 50.13, 55.37, 59.61, 81.71, 113.24, 116.07, 128.64, 130.74, 131.43, 135.12, 136.15, 140.04, 148.34, 151.32, 157.58, 170.04, 195.89; (+)ESI-MS (*m/z*): [M + H] ^+^: 481.20, Anal Calcd for C_27_H_29_ClN_2_O_4_: C; 67.42, H; 6.08, N; 5.82, Found: C; 67.56, H; 6.30, N; 5.90.

##### (±)-Ethyl 2-amino-4–(4-nitrophenyl)-7,7-dimethyl-5-oxo-1-phenyl -1,4,5,6,7,8-hexahydroquinoline-3-carboxylate (8i)

Yellow powder, yield: 33%; m.p. 164–166 °C. ^1^H NMR (400 MHz, CDCl_3_) *δ* (ppm): 0.76 (s, 3H, CH_3_-dimedone), 0.96 (s, 3H, CH_3_-dimedone), 1.19 (t, 3H, OCH_2_**CH_3_**, *J* = 8.0 Hz), 1.77 (d, 1H, COCH_2_, *J* = 16.0 Hz), 2.09 (d, 1H, COCH_2_, *J* = 16.0 Hz), 2.13 (d, 1H, **CH_2_**C(CH_3_)_2_, *J* = 16.0 Hz), 2.23 (d, 1H, **CH_2_**C(CH_3_)_2_, *J* = 16.0 Hz), 4.03–4.10 (m, 2H, O**CH_2_**CH_3_), 5.24 (s, 1H, HHQ, 4-H), 6.34 (*br*. s, 2H, NH_2_), 7.35 (d, 2H, Ar-H, *J* = 8.0 Hz), 7.58–7.63 (m, 5H, Ar-H), 8.14 (d, 2H, Ar-H, *J* = 8.0 Hz),^13^C NMR (100 MHz, CDCl_3_) *δ* (ppm): 14.55, 27.02, 29.46, 32.25, 36.78, 42.15, 50.10, 59.21, 80.13, 113.77, 123.47, 126.53, 127.09, 130.07, 130.31, 130.44, 132.81, 133.16, 136.46, 146.04, 149.56, 152.02, 170.21, 195.76; (+)ESI-MS (*m/z*): [M + Na]^+^: 484.30, Anal Calcd. for C_26_H_27_N_3_O_5_: C; 67.66, H; 5.90, N; 9.10. Found: C; 67.49, H; 6.12, N; 9.24.

##### (±)-Ethyl 2-amino-7,7-dimethyl-1–(4-methylphenyl)-4–(4-nitrophenyl)-5-oxo-1,4,5,6,7,8-hexahydroquinoline-3-carboxylate (8j)

Brown powder, yield: 65%; m.p. 270–272 °C. ^1^H NMR (400 MHz, CDCl_3_) *δ* (ppm): 0.77 (s, 3H, CH_3_-dimedone), 0.95 (s, 3H, CH_3_-dimedone), 1.20 (t, 3H, OCH_2_**CH_3_**, *J* = 8.0 Hz), 1.77 (d, 1H, COCH_2_, *J* = 16.0 Hz), 2.05 (d, 1H, COCH_2,_ *J* = 16.0 Hz), 2.12 (d, 1H, **CH_2_**C(CH_3_)_2_, *J* = 16.0 Hz), 2.21 (d, 1H, **CH_2_**C(CH_3_)_2_, *J* = 16.0 Hz), 2.49 (s, 3H, CH_3_), 4.04–4.08 (m, 2H, O**CH_2_**CH_3_), 5.12 (s, 1H, HHQ, 4-H), 6.25 (*br*. s, 2H, NH_2_), 7.18–7.22 (m, 4H, Ar-H), 7.34–7.39 (m, 4H, Ar-H), ^13^C NMR (100 MHz, CDCl_3_) *δ* (ppm): 14.42, 21.33 26.61, 29.64, 32.41, 35.00, 41.90, 50.02, 59.44, 79.71, 114.13, 123.30, 128.72, 129.57, 131.31, 133.20, 140.68, 146.68, 146.04, 150.11, 152.13, 155.54, 169.61, 195.72; (+)ESI-MS (*m/z*): [M + H_2_O]^+^: 491.30, Anal Calcd. for C_27_H_29_N_3_O_5_: C; 68.19, H; 6.15, N; 8.84. Found: C; 68.48, H; 6.33, N; 9.09.

##### (±)-Ethyl2-amino-1–(4-chlorophenyl)-7,7-dimethyl-4–(4-nitrophenyl)-5-oxo-1,4,5,6,7,8-hexahydroquinoline-3-carboxylate (8k)

Faint brown powder, yield: 65%; m.p. 198–200 °C. ^1^H NMR (400 MHz, CDCl_3_) *δ* (ppm): 0.77 (s, 3H, CH_3_-dimedone), 0.96 (s, 3H, CH_3_-dimedone), 1.26 (t, 3H, OCH_2_**CH_3_**, *J* = 8.0 Hz), 1.73 (d, 1H, COCH_2_, *J* = 16.0 Hz), 2.05 (d, 1H, COCH_2_, *J* = 16.0 Hz), 2.18 (d, 1H, **CH_2_**C(CH_3_)_2_, *J* = 16.0 Hz), 2.22 (d, 1H, **CH_2_**C(CH_3_)_2_, *J* = 16.0 Hz), 4.04–4.09 (m, 2H, O**CH_2_**CH_3_), 5.11 (s, 1H, HHQ, 4-H), 6.20 (*br*. s, 2H, NH_2_), 7.21 (d, 2H, Ar-H, *J* = 8.0 Hz), 7.27 (d, 2H, Ar-H, *J* = 8.0 Hz), 7.32 (d, 2H, Ar-H, *J* = 8.0 Hz), 7.57 (d, 2H, Ar-H, *J* = 8.0 Hz), ^13^C NMR (100 MHz, CDCl_3_) *δ* (ppm): 14.40, 26.66, 29.68, 32.44, 33.85, 41.94, 50.07, 59.45, 81.00, 115.46, 128.13, 129.16, 130.84, 131.37, 134.87, 136.33, 146.16, 148.71, 151.47, 169.81, 195.76; (+)ESI-MS (*m/z*): [M + Na]^+^: 518.60, Anal Calcd. for C_26_H_26_ClN_3_O_5_: C; 62.97, H; 5.28, N; 8.47. Found: C; 63.10, H; 5.56, N; 8.29.

##### (±)-Ethyl 2-amino-7,7-dimethyl-4-[4-(morpholin-4-yl) phenyl]- 5-oxo-1-phenyl -1,4,5,6,7,8-hexahydroquinoline-3-carboxylate (8l)

Orange powder, yield: 44%; m.p. 193–195 °C. ^1^H NMR (400 MHz, DMSO-d_6_) *δ* (ppm): 0.71 (s, 3H, CH_3_-dimedone), 0.88 (s, 3H, CH_3_-dimedone), 1.15 (t, 3H, OCH_2_**CH_3_**, *J* = 8.0 Hz), 1.64 (d, 1H, COCH_2_, *J* = 16.0 Hz), 1.98 (d, 1H, COCH_2_, *J* = 16.0 Hz), 2.10 (d, 1H, **CH_2_**C(CH_3_)_2_, *J* = 16.0 Hz), 2.18 (d, 1H, **CH_2_**C(CH_3_)_2_, *J* = 16.0 Hz), 3.05 (s, 4H, morpholine), 3.71 (s, 4H, morpholine), 3.97 (m, 2H, O**CH_2_**CH_3_), 4.86 (s, 1H, HHQ, 4-H), 6.65 (*br*. s, 2H, NH_2_), 6.84 (d, 2H, Ar-H, *J* = 8.0 Hz), 7.17 (d, 2H, Ar-H, *J* = 8.0 Hz), 7.40 (d, 2H, Ar-H, *J* = 8.0 Hz), 7.59–7.65 (m, 3H, Ar-H, *J* = 8.0 Hz), ^13^C NMR (100 MHz, CDCl_3_) *δ* (ppm): 14.44, 26.82, 29.68, 32.45, 33.17, 41.92, 46.98, 49.67, 50.17, 59.35, 62.15, 66.43, 67.04, 81.80, 113.54, 115.41, 116.13, 128.39, 130.71, 131.42, 133.74, 135.22, 136.13, 148.27, 149.19, 151.30, 154.23, 170.06, 195.84; (+)ESI-MS (*m/z*): [M + Na]^+^: 524.20, Anal Calcd. for C_30_H_35_N_3_O_4_: C; 71.83, H; 7.03, N; 8.38. Found: C; 72.05, H; 7.29, N; 8.60.

##### (±)-Ethyl 2-amino-1–(4-chlorophenyl)-7,7-dimethyl-4-[4-(morpholin-4-yl) phenyl]-5-oxo-1,4,5,6,7,8-hexahydroquinoline-3-carboxylate (8m)

Orange powder, yield: 71%; m.p. 175–177 °C. ^1^H NMR (400 MHz, CDCl_3_) *δ* (ppm): 0.80 (s, 3H, CH_3_-dimedone), 0.97 (s, 3H, CH_3_-dimedone), 1.23 (t, 3H, OCH_2_**CH_3_**, *J* = 8.0 Hz), 1.75 (d, 1H, COCH_2_, *J* = 16.0 Hz), 2.06 (d, 1H, COCH_2_, *J* = 16.0 Hz), 2.14 (d, 1H, **CH_2_**C(CH_3_)_2_, *J* = 16.0 Hz), 2.22 (d, 1H, **CH_2_**C(CH_3_)_2_, *J* = 16.0 Hz), 3.13–3.18 (m, 4H, morpholine), 3.86–3.92 (m, 4H, morpholine), 4.02–4.14 (m, 2H, O**CH_2_**CH_3_), 5.08 (s, 1H, HHQ, 4-H), 6.17 (*br*. s, 2H, NH_2_), 6.92 (t, 2H, Ar-H, *J* = 8.0 Hz), 7.29–7.34 (m, 4H, Ar-H), 7.58 (d, 1H, Ar-H, *J* = 8.0 Hz), 7.98 (d, 1H, Ar-H, *J* = 8.0 Hz),^13^C NMR (100 MHz, CDCl_3_) *δ* ppm):14.46, 26.81, 29,69, 32.46, 33.26, 41.91, 46.94, 50.07, 59.39, 62.18, 66.43, 66.80, 81.65, 113.53, 115.96, 121.75, 128.50, 130.75, 131.41, 133.76, 135.10, 136.17, 148.51, 151.32, 154.10, 154.28, 170.03, 195.99; (+)ESI-MS (*m/z*): [M + H]^+^: 536.10, Anal Calcd. for C_30_H_34_ClN_3_O_4_: C; 67.22, H; 6.39, N; 7.84. Found: C; 67.50, H; 6.61, N; 7.52.

##### (±)-Ethyl 2-amino-4–(2,3-dichlorophenyl)-7,7-dimethyl-5-oxo-1-phenyl-1,4,5,6,7,8-hexahydroquinoline-3-carboxylate (10a)

Yellow powder, yield: 58%; m.p. 235–237 °C. ^1^H NMR (400 MHz, CDCl_3_) *δ* (ppm): 0.84 (s, 3H, CH_3_-dimedone), 0.95 (s, 3H, CH_3_-dimedone), 1.17 (t, 3H, OCH_2_**CH_3_**, *J* = 8.0 Hz), 1.80 (d, 1H, COCH_2,_ *J* = 16.0 Hz), 2.01 (d, 2H, COCH_2,_ *J* = 16.0 Hz), 2.11 (d, 1H, **CH_2_**C(CH_3_)_2_, *J* = 16.0 Hz), 2.18 (d, 1H, **CH_2_**C(CH_3_)_2_, *J* = 16.0 Hz), 3.96–4.05 (m, 2H, O**CH_2_**CH_3_), 5.44 (s, 1H, HHQ, 4-H), 6.31 (*br*. s, 2H, NH_2_), 7.12 (t, 1H, Ar-H, *J* = 8.0 Hz), 7.25 (d, 2H, Ar-H, *J* = 8.0 Hz), 7.50 (d, 1H, Ar-H, *J* = 8.0 Hz), 7.61 (m, 3H, Ar-H), ^13^C NMR (100 MHz, CDCl_3_) *δ* (ppm); 14.39, 27.02, 29.45, 32.26, 36.57, 42.09, 50.05, 59.30, 78.92, 112.58, 126.04, 127.85, 130.17, 130.31, 131.71, 131.85, 133.06, 136.27, 146.25, 150.23, 152.28, 170.05, 195.86; ESI-MS (*m/z*): [M]^+^: 484.13, Anal Calcd. for C_26_H_26_Cl_2_N_2_O_3_: C; 64.33, H; 5.40, N; 5.77. Found: C; 64.12, H; 5.46, N; 5.69.

##### (±)-Ethyl 2-amino-1–(4-chorophenyl)-4–(2,3-dichlorophenyl)-7,7-dimethyl-5-oxo-1-phenyl-1,4,5,6,7,8-hexahydroquinoline-3-carboxylate (10b)

Yellow powder, yield: 63%; m.p. 250–252 °C. ^1^H NMR (400 MHz, CDCl_3_) *δ* (ppm): 0.84 (s, 3H, CH_3_-dimedone), 0.96 (s, 3H, CH_3_-dimedone), 1.15 (t, 3H, OCH_2_CH_3_, *J* = 8.0 Hz), 1.78 (d, 1H, COCH_2,_ *J* = 16.0 Hz), 2.00 (d, 1H, COCH_2_, *J* = 16.0 Hz), 2.10 (d, 1H, **CH_2_**C(CH_3_)_2_, *J* = 16.0 Hz), 2.17 (d, 1H, **CH_2_**C(CH_3_)_2_, *J* = 16.0 Hz), 3.97–4.06 (m, 2H, O**CH_2_**CH_3_), 5.41 (s, 1H, HHQ, 4-H), 6.28 (*br*. s, 2H, NH_2_), 7.11 (t, 1H, Ar-H, *J* = 8.0 Hz), 7.26–7.33 (m, 4H, Ar-H), 7.48 (d, 1H, Ar-H, *J* = 8.0 Hz), 7.58 (d, 1H, Ar-H, *J* = 8.0 Hz), ^13^C NMR (100 MHz, CDCl_3_) *δ* (ppm): 14.36, 27.01, 29.48, 32.29, 36.59, 42.14, 49.99, 59.37, 79.19, 112.78, 126.07, 127.94, 130.79, 131.66, 131.76, 131.81, 133.09, 134.75, 136.36, 146.00, 149.75, 151.97, 169.94, 195.74.; ESI-MS *(m/z)*: [M]^+^: 518.69, Anal Calcd. for C_26_H_25_Cl_3_N_2_O_3_: C; 60.07, H; 4.85, N; 5.39. Found: C; 59.90, H; 4.79, N; 5.49.

##### (±)-Ethyl 2-amino-4–(2,4-dichlorophenyl)-7,7-dimethyl-5-oxo-1-phenyl-1,4,5,6,7,8-hexahydroquinoline-3-carboxylate (10c)

Yellow powder, yield: 46%; m.p. 226–228 °C. IR (KBr disc): ῡ (cm^−1^): 3472 (NH_2_ str.), 3059 (aromatic C-H str.), 2948 (aliphatic C-H str.), 1662 (C = O str.), 1495 (aliphatic C = C str.), 1266 (aliphatic C-N str.), 1209 (aliphatic C-O str.), ^1^H NMR (400 MHz, CDCl_3_) *δ* (ppm): 0.83 (s, 3H, CH_3_-dimedone), 0.94 (s, 3H, CH_3_-dimedone), 1.19 (t, 3H, OCH_2_**CH_3_**, *J* = 8.0 Hz), 1.77 (d, 1H, COCH_2,_ *J* = 8.0 Hz), 2.01 (d, 1H, COCH_2_, *J* = 16.0 Hz), 2.09 (d, 1H, **CH_2_**C(CH_3_)_2_, *J* = 16.0 Hz), 2.18 (d, 1H, **CH_2_**C(CH_3_)_2_, *J* = 16.0 Hz), 4.00–4.08 (m, 2H, O**CH_2_**CH_3_), 5.34 (s, 1H, HHQ, 4-H), 6.31 (*br*. s, 2H, NH_2_), 7.14–7.17 (m, 1H, Ar-H), 7.36 (d, 3H, Ar-H, *J* = 8.0 Hz), 7.48–7.51 (m, 1H, Ar-H), 7.56–7.61 (m, 3H, Ar-H), ^13^C NMR (100 MHz, CDCl_3_) *δ* (ppm); 14.42, 26.84, 29.56, 32.24, 35.22, 42.07, 50.02, 59.31, 78.87, 112.57, 126.09, 129.47, 130.16, 130.27, 130.50, 131.68, 134.05, 134.09, 136.25, 142.65, 150.10, 152.19, 169.99, 195.82.; ESI-MS (*m/z*): [M]^+^: 484.67, Anal Calcd. for C_26_H_26_Cl_2_N_2_O_3_: C; 64.33, H; 5.40, N; 5.77. Found: C; 64.21, H; 5.41, N; 5.84.

##### (±)-Ethyl 2-amino-1–(4-chorophenyl)-4–(2,4-dichlorophenyl)-7,7-dimethyl-5-oxo-1-phenyl-1,4,5,6,7,8-hexahydroquinoline-3-carboxylate (10d)

Yellow powder, yield: 50%; m.p. 217–219 °C. IR (KBr disc): ῡ (cm^−1^): 3408 (NH_2_ str), 3061 (aromatic C-H str), 2960 (aliphatic C-H str), 1640 (C = O str), 1497 (aliphatic C = C str), 1210 (aliphatic C-N str), 1175 (aliphatic C-O str), ^1^H NMR (400 MHz, CDCl_3_) *δ* (ppm): 0.85 (s, 3H, CH_3_-dimedone), 0.96 (s, 3H, CH_3_-dimedone), 1.18 (t, 3H, OCH_2_**CH_3_**, *J* = 8.0 Hz), 1.77 (d, 1H, COCH_2_, *J* = 16.0 Hz), 2.00 (d, 1H, COCH_2_, *J* = 16.0 Hz), 2.10 (d, 1H, **CH_2_**C(CH_3_)_2_, *J* = 16.0 Hz), 2.18 (d, 1H, **CH_2_**C(CH_3_)_2_, *J* = 16.0 Hz), 3.98–4.04 (m, 2H, O**CH_2_**CH_3_), 5.32 (s, 1H, HHQ, 4-H), 6.27 (br. s, 2H, NH_2_), 7.16 (d, 1H, Ar-H, *J* = 8.0 Hz), 7.31 (s, 3H, Ar-H), 7.48 (d, 1H, Ar-H, *J* = 8.0 Hz), 7.59 (d, 2H, Ar-H, *J* = 8.0 Hz), ^13^C NMR (100 MHz, CDCl_3_) *δ* (ppm): 14.38, 26.86, 29.59, 32.29, 35.34, 42.15, 49.97, 59.42, 79.13, 112.74, 126.10, 129.55, 130.79, 131,64, 131.82, 134.03, 134.23, 134.76, 136.38, 142.28, 149.59, 151.86, 169.93, 195.73ESI-MS (*m/z*): [M]^+^: 518.18, Anal Calcd. for C_26_H_25_Cl_3_N_2_O_3_: C; 60.07, H; 4.85, N; 5.39. Found: C; 60.22, H; 4.93, N; 5.35.

##### (±)-Ethyl 2-amino-7,7-dimethyl-5-oxo-1-phenyl-4-(pyridin-2-yl)-1,4,5,6,7,8- hexahydroquinoline-3-carboxylate (12a)

Brown powder, yield: 93%; m.p. 158–160 °C. ^1^H NMR (400 MHz, DMSO-d_6_) *δ* (ppm): 0.78 (s, 3H, CH_3_-dimedone), 0.95 (s, 3H, CH_3_-dimedone), 1.19 (t, 3H, OCH_2_**CH_3_**, *J* = 8.0 Hz), 1.77 (d, 1H, COCH_2_, *J* = 16.0 Hz), 2.07 (d, 1H, COCH_2_, *J* = 16.0 Hz), 2.12 (d, 1H, **CH_2_**C(CH_3_)_2_, *J* = 16.0 Hz), 2.16 (d, 1H, **CH_2_**C(CH_3_)_2_, *J* = 16.0 Hz), 4.06 (m, 2H, O**CH_2_**CH_3,_ *J* = 8.0 Hz), 5.12 (s, 1H, HHQ, 4-H), 6.27 (*br*. s, 2H, NH_2_), 7.16–7.19 (m, 1H, Ar-H), 7.35 (d, 2H, Ar-H, *J* = 8.0 Hz), 7.60 (d, 3H, Ar-H, *J* = 8.0 Hz), 7.75 (d, 1H, Ar-H, *J* = 8.0 Hz), 8.36 (d, 1H, Ar-H, *J* = 8.0 Hz), 8.66 (s, 1H, Ar-H), ^13^C NMR (100 MHz, CDCl_3_) *δ* (ppm): 14.41, 26.78, 29.56, 32.43, 32.58, 32.80, 41.87, 50.04, 59.36, 80.22, 114.53, 122.80, 130.03, 130.19, 130.59, 135.70, 136.18, 143.21, 146.94, 149.75, 151.85, 169.65, 195.66; (+)ESI-MS (*m/z*): [M + H]^+^: 418.20, Anal Calcd. for C_25_H_27_N_3_O_3_: C; 71.92, H; 6.52, N; 10.06. Found: C; 72.10, H; 6.73, N; 10.21.

##### (±)-Ethyl 2-amino-7,7-dimethyl-1–(4-methylphenyl)-5-oxo-1-phenyl-4-(pyrdin-2-yl)-1,4,5,6,7,8-hexahydroquinoline-3-carboxylate (12b)

Brown powder, yield: 49%; m.p. 196–198 °C. ^1^H NMR (400 MHz, CDCl_3_) *δ* (ppm): 0.81 (s, 3H, CH_3_-dimedone), 0.96 (s, 3H, CH_3_-dimedone), 1.22 (t, 3H, OCH_2_**CH_3_**, *J* = 8.0 Hz), 2.06 (d, 1H, COCH_2_, *J* = 16.0 Hz), 2.09 (d, 1H, COCH_2_), 2.16 (d, 1H, **CH_2_**C(CH_3_)_2_, *J* = 16.0 Hz), 2.21 (d, 1H, **CH_2_**C(CH_3_)_2_, *J* = 16.0 Hz), 2.47 (s, 3H, CH_3_), 4.08 (q, 2H, O**CH_2_**CH_3_), 5.25 (s, 1H, HHQ, 4-H), 6.36 (*br*. s, 2H, NH_2_), 7.29 (s, 1H, Ar-H), 7.37 (d, 3H, Ar-H, *J* = 8.0 Hz), 7.56 (d, 3H, Ar-H, *J* = 8.0 Hz), 8.54 (s, 1H, Ar-H), ^13^C NMR (100 MHz, CDCl_3_) *δ* (ppm): 14.53, 21.33 26.80, 29.48, 32.54, 36.82, 41.74, 50.18, 59.13, 61.29, 77.28 (masked by solvent peaks), 121.02, 122.22, 124.94, 125.49, 129.88, 134.26, 136.58, 138.74, 152.78, 159.87, 169.78, 196.07; (+)ESI-MS (*m/z*): [M + Na]^+^: 454.10, Anal Calcd.for C_26_H_29_N_3_O_3_: C; 72.37, H; 6.77, N; 9.74. Found: C; 72.55, H; 7.04, N; 10.03.

##### (±)-Ethyl 2-amino-1–(4-chlorophenyl)-7,7-dimethyl-5-oxo-4-(pyridine-2-yl)-1,4,5,6,7,8-hexahydroquinoline-3-carboxylate (12c)

Brown powder, Yield: 60%; m.p. 256–258 °C. ^1^H NMR (400 MHz, CDCl_3_) *δ* (ppm): 0.78 (s, 3H, CH_3_-dimedone), 0.96 (s, 3H, CH_3_-dimedone), 1.19 (t, 3H, OCH_2_**CH_3_**, *J* = 8.0 Hz,), 2.08 (d, 1H, COCH_2_, *J* = 16.0 Hz), 2.11 (d, 1H, COCH_2,_ *J* = 16.0 Hz *J* = 16.0 Hz), 2.15 (d, 1H, CH_2_C(CH_3_)_2_, *J* = 16.0 Hz), 2.21 (d, 1H, CH_2_C(CH_3_)_2_, *J* = 16.0 Hz), 4.03–4.07 (m, 2H, O**CH_2_**CH_3,_ *J* = 8.0 Hz), 5.09 (s, 1H, HHQ, 4-H), 6.29 (*br*. s, 2H, NH_2_), 7.16 (m, 1H, Ar-H), 7.29 (d, 2H, Ar-H, *J* = 8.0 Hz), 7.55 (d, 2H, Ar-H, *J* = 8.0 Hz), 7.73 (d, 1H, Ar-H, *J* = 8.0 Hz), 8.33 (m, 1H, Ar-H, *J* = 8.0 Hz), 8.60 (s, 1H, Ar-H), ^13^C NMR (100 MHz, CDCl_3_) *δ* (ppm): 14.49, 27.03, 28.32, 29.30, 32.58, 32.82, 36.85, 41.78, 43.48, 50.08, 50.34, 59.30, 99.09, 121.38, 124.95, 129.41, 130.59, 131.72, 135.59, 135.99, 136.96, 152.48, 159.73, 169.53, 196.06; (+)ESI-MS (*m/z*): [M + H]^+^: 452.20, Anal Calcd for C_25_H_26_ClN_3_O_3_: C; 66.44, H; 5.80, N; 9.30, Found: C; 66.22, H; 6.09, N; 9.52.

##### (±)-Ethyl 2-amino-7,7-dimethyl-5-oxo-4-(pyridin-3-yl)-1,4,5,6,7,8- hexahydroquinoline-3-carboxylate (12d)

Yellow powder, Yield: 57%; m.p. 215–217 °C. ^1^H NMR (400 MHz, CDCl_3_) *δ* (ppm): 0.77 (s, 3H, CH_3_-dimedone), 0.95 (s, 3H, CH_3_-dimedone), 1.19 (t, 3H, OCH_2_**CH_3_**, *J* = 8.0 Hz), 1.78 (d, 1H, COCH_2_, *J* = 16.0 Hz), 2.06 (d, 1H, COCH_2_, *J* = 16.0 Hz), 2.11 (d, 1H, **CH_2_**C(CH_3_)_2_, *J* = 16.0 Hz), 2.21 (d, 1H, **CH_2_**C(CH_3_)_2_, *J* = 16.0 Hz), 4.04–4.07 (m, 2H, O**CH_2_**CH_3_), 5.12 (s, 1H, HHQ, 4-H), 6.30 (br. s, 2H, NH_2_), 7.21–7.24 (m, 1H, Ar-H), 7.36 (d, 2H, Ar-H, *J* = 8.0 Hz), 7.58–7.63 (m, 2H, Ar-H), 7.81–7.84 (m, 2H, Ar-H), 8.36–8.37 (m, 1H, Ar-H), 8.65–8.66 (d, 1H, Ar-H, *J* = 8.0 Hz), ^13^C NMR (100 MHz, CDCl_3_) *δ* (ppm): 14.41, 26.84, 29.48, 32.43, 32.99, 41.84, 49.98, 59.39, 79.90, 114.23, 123.07, 130.26, 130.63, 136.02, 136.82, 143.89, 145.80, 148.45, 149.79, 151.90, 169.50, 195.68; (+)ESI-MS (*m/z*): [M + H]^+^: 418.50; Anal Calcd for C_25_H_27_N_3_O_3_, C; 71.92, H; 6.52, N; 10.06, Found: C; 72.00, H; 6.71, N; 10.22.

##### (±)-Ethyl 2-amino-7,7-dimethyl-1–(4-methylphenyl)-5-oxo-1-phenyl-4-(pyrdin-3-yl)-1,4,5,6,7,8-hexahydroquinoline-3-carboxylate (12e)

Yellow powder, yield: 84%; m.p. 261–263 °C. ^1^H NMR (400 MHz, CDCl_3_) *δ* (ppm): 0.79 (s, 3H, CH_3_-dimedone), 0.93 (s, 3H, CH_3_-dimedone), 1.19 (t, 3H, OCH_2_**CH_3_**, *J* = 8.0 Hz), 1.78 (d, 1H, COCH_2_, *J* = 16.0 Hz), 2.02 (d, 1H, COCH_2_, *J* = 16.0 Hz), 2.07 (d, 1H, **CH_2_**C(CH_3_)_2_, *J* = 16.0 Hz), 2.16 (d, 1H, **CH_2_**C(CH_3_)_2_, *J* = 16.0 Hz), 2.49 (s, 3H, CH_3_), 3.99–4.03 (m, 2H, O**CH_2_**CH_3_), 5.27 (s, 1H, HHQ, 4-H), 6.23 (*br*. s, 2H, NH_2_), 6.82–6.89 (m, 2H, Ar-H), 7.08–7.12 (m, 1H, Ar-H), 7.25–7.28 (m, 2H, Ar-H), 7.37–7.45 (m, 3H, Ar-H), ^13^C NMR (100 MHz, CDCl_3_) *δ* (ppm): 14.36, 21.31, 26.46, 29.73, 32.34, 32.55, 33.19, 42.20, 50.17, 55,90, 59.06, 79.82, 111.34, 113.71, 119.89, 120.21, 126.83, 129.85, 130.96, 132.20, 140.01, 149.60, 152.16, 157.89, 170.52, 195.81; (+)ESI-MS (*m/z*): [M + H]^+^: 432.50, Anal Calcd. for C_26_H_29_N_3_O_3_: C; 72.37, H; 6.77, N; 9.74. Found: C; 72.51, H; 7.01, N; 10.02.

##### (±)-Ethyl 2-amino-1–(4-chlorophenyl)-7,7-dimethyl-5-oxo-4-(pyridin-3-yl)-1,4,5,6,7,8- hexahydroquinoline-3-carboxylate (12f)

Shiny white powder, Yield: 87%; m.p. 261–263 °C. ^1^H NMR (400 MHz, CDCl_3_) *δ* (ppm): 0.79 (s, 3H, CH_3_-dimedone), 0.98 (s, 3H, CH_3_-dimedone), 1.21 (t, 3H, OCH_2_**CH_3_**, *J* = 8.0 Hz), 1.77 (d, 1H, COCH_2_, *J* = 16.0 Hz), 2.06 (d, 1H, COCH_2_, *J* = 16.0 Hz), 2.13 (d, 1H, **CH_2_**C(CH_3_)_2_, *J* = 16.0 Hz), 2.22 (d, 1H, **CH_2_**C(CH_3_)_2_, *J* = 16.0 Hz), 4.03–4.09 (m, 2H, O**CH_2_**CH_3_), 5.10 (s, 1H, HHQ, 4-H), 6.26 (*br*. s, 2H, NH_2_), 7.18–7.21 (m, 1H, Ar-H); 7.32 (d, 2H, Ar-H, *J* = 8.0 Hz); 7.59 (d, 2H, Ar-H, *J* = 8.0 Hz); 7.75 (d, 1H, Ar-H, *J* = 8.0 Hz); 8.36 (d, 1H, Ar-H, *J* = 8.0 Hz), 8.62 (d, 1H, Ar-H, *J* = 8.0 Hz), ^13^C NMR (100 MHz, CDCl_3_) *δ* (ppm): 14.41, 27.07, 29.33, 32.54, 33.43, 41.92, 49.87, 59.61, 79.65, 113.97, 123.69, 131.41, 134.22, 136.66, 139.51, 143.32, 145.27, 145.93, 149.68, 151.66, 169.18, 195.65; (+)ESI-MS (*m/z*): [M + H]^+^: 452.50, Anal Calcd for C_25_H_26_ClN_3_O_3_, C; 66.44, H; 5.80, N; 9.30, Found: C; 66.62, H; 6.03, N; 9.52.

#### X-ray crystallography

A colourless prism crystal of compound **6f** (accession number 2163585) was picked up as a representative sample to study the enantiomerism of our target compounds^52^^,^^53^. The detailed procedures of the x-ray crystallography technique are discussed in the Supplementary file.

### Biology

The comprehensive procedures of biological assays of the target HHQ analogues (**6a–i**, **8a–m**, **10a–d**, and **12a–f** are presented in the Supplementary file, including; preliminary *in vitro* anticancer screening^105^, EGFR kinase inhibitory assay^106^, IMR-90 normal lung cell line for safety^76^^,^^77^, Annexin V-FITC apoptosis assay^107^, and cellular mechanistic analysis^108^.

### In silico studies

*In silico* studies of the representative target HHQ analogues **10c** and **10d** were presented in the Supplementary file. These studies include molecular docking analysis and predicting targeted compounds’ physicochemical properties and pharmacokinetics using Molsoft software and the SwissADME web tool^91^^,^^92^^,^^109^^,^^110^. The procedures of these studies are presented in detail in the Supplementary file.

## Supplementary Material

Supplemental Material

## References

[1] Kothayer H, Rezq S, Abdelkhalek AS, Romero DG, Elbaramawi SS. Triple targeting of mutant EGFRL858R/T790M, COX-2, and 15-LOX: design and synthesis of novel quinazolinone tethered phenyl urea derivatives for anti-inflammatory and anticancer evaluation. J Enzyme Inhib Med Chem. 2023;38(1):2199166. 37038884 10.1080/14756366.2023.2199166 PMC10114980

[2] Engle K, Kumar G. Cancer multidrug-resistance reversal by ABCB1 inhibition: a recent update. Eur J Med Chem. 2022;239:114542. 35751979 10.1016/j.ejmech.2022.114542

[3] Chen S, Zhao Y, Liu S, Zhang J, Assaraf YG, Cui W, Wang L. Epigenetic enzyme mutations as mediators of anti-cancer drug resistance. Drug Resist Updates. 2022;61:100821. 10.1016/j.drup.2022.100821 35219075

[4] Wang Z, Cai J, Cheng J, Yang W, Zhu Y, Li H, Lu T, Chen Y, Lu S. FLT3 inhibitors in acute myeloid leukemia: challenges and recent developments in overcoming resistance. J Med Chem. 2021;64(6):2878–2900. 33719439 10.1021/acs.jmedchem.0c01851

[5] Sung H, Ferlay J, Siegel RL, Laversanne M, Soerjomataram I, Jemal A, Bray F. Global cancer statistics 2020: GLOBOCAN estimates of incidence and mortality worldwide for 36 cancers in 185 countries. CA Cancer J Clin. 2021;71:209–249. 33538338 10.3322/caac.21660

[6] Wu M, Zhang P. EGFR-mediated autophagy in tumourigenesis and therapeutic resistance. Cancer Lett. 2020;469:207–216. 31639425 10.1016/j.canlet.2019.10.030

[7] El-Haggar R, Hammad SF, Alsantali RI, Alrooqi MM, El Hassab MA, Masurier N, Ahmed MF. 3-Substituted-2,3-dihydrothiazole as a promising scaffold to design EGFR inhibitors. Bioorg Chem. 2022;129:106172. 36182865 10.1016/j.bioorg.2022.106172

[8] Ahmed SA, Kamel MS, Aboelez MO, Ma X, Al-Karmalawy AA, Mousa SAS, Shokr EK, Abdel-Ghany H, Belal A, El Hamd MA, et al. Thieno [2, 3-b] thiophene derivatives as potential EGFRWT and EGFRT790M inhibitors with antioxidant activities: microwave-assisted synthesis and quantitative *in vitro* and *in silico* studies. ACS Omega. 2022;7(49):45535–45544. 36530244 10.1021/acsomega.2c06219 PMC9753534

[9] Hong SY, Kao YR, Lee TC, Wu CW. Upregulation of E3 ubiquitin ligase CBLC enhances EGFR dysregulation and signaling in lung adenocarcinomaCBLC dysregulates EGFR signaling. Cancer Res. 2018;78(17):4984–4996. 29945960 10.1158/0008-5472.CAN-17-3858

[10] Jänne PA, Baik C, Su W-C, Johnson ML, Hayashi H, Nishio M, Kim D-W, Koczywas M, Gold KA, Steuer CE, et al. Efficacy and safety of patritumab deruxtecan (HER3-DXd) in EGFR inhibitor–resistant, EGFR-mutated non–small cell lung cancer. Cancer Discov. 2022;12(1):74–89. 34548309 10.1158/2159-8290.CD-21-0715 PMC9401524

[11] Lieser RM, Li Q, Chen W, Sullivan MO. Incorporation of endosomolytic peptides with varying disruption mechanisms into EGFR-Targeted protein conjugates: the effect on intracellular protein delivery and EGFR specificity in breast cancer cells. Mol Pharm. 2022;19(2):661–673. 35040326 10.1021/acs.molpharmaceut.1c00788

[12] Farag AK, Ahn BS, Yoo JS, Karam R, Roh EJ. Design, synthesis, and biological evaluation of pseudo-bicyclic pyrimidine-based compounds as potential EGFR inhibitors. Bioorg Chem. 2022;126:105918. 35696765 10.1016/j.bioorg.2022.105918

[13] El-Naggar AM, Hassan A, Elkaeed EB, Alesawy MS, Al‐Karmalawy AA. Design, synthesis, and SAR studies of novel 4-methoxyphenyl pyrazole and pyrimidine derivatives as potential dual tyrosine kinase inhibitors targeting both EGFR and VEGFR-2. Bioorg Chem. 2022;123:105770. 35395446 10.1016/j.bioorg.2022.105770

[14] de Lima PO, Joseph S, Panizza B, Simpson F. Epidermal growth factor receptor’s function in cutaneous squamous cell carcinoma and its role as a therapeutic target in the age of immunotherapies. Curr Treat Options Oncol. 2020;21(1):9. 32016630 10.1007/s11864-019-0697-3

[15] Cooper AJ, Sequist LV, Lin JJ. Third-generation EGFR and ALK inhibitors: mechanisms of resistance and management. Nat Rev Clin Oncol. 2022;19(8):499–514. 35534623 10.1038/s41571-022-00639-9 PMC9621058

[16] Gelatti AC, Drilon A, Santini FC. Optimizing the sequencing of tyrosine kinase inhibitors (TKIs) in epidermal growth factor receptor (EGFR) mutation-positive non-small cell lung cancer (NSCLC. Lung Cancer. 2019;137:113–122. 31568888 10.1016/j.lungcan.2019.09.017 PMC7478849

[17] Liang H, Pan Z, Wang W, Guo C, Chen D, Zhang J, Zhang Y, Tang S, He J, Liang W. The alteration of T790M between 19 del and L858R in NSCLC in the course of EGFR-TKIs therapy: a literature-based pooled analysis. J Thorac Dis. 2018;10(4):2311–2320. 29850136 10.21037/jtd.2018.03.150 PMC5949462

[18] Qin X, Liu P, Li Y, Hu L, Liao Y, Cao T, Yang L. Design, synthesis and biological evaluation of novel 3, 4-dihydro-2H-[1, 4] oxazino [2, 3-f] quinazolin derivatives as EGFR-TKIs. Bioorg Med Chem Lett. 2023;80:129104. 36509365 10.1016/j.bmcl.2022.129104

[19] Dong RF, Zhu ML, Liu MM, Xu YT, Yuan LL, Bian J, Xia YZ, Kong LY. EGFR mutation mediates resistance to EGFR tyrosine kinase inhibitors in NSCLC: from molecular mechanisms to clinical research. Pharmacol Res. 2021;167:105583. 33775864 10.1016/j.phrs.2021.105583

[20] Beyett TS, To C, Heppner DE, Rana JK, Schmoker AM, Jang J, De Clercq DJH, Gomez G, Scott DA, Gray NS, et al. Molecular basis for cooperative binding and synergy of ATP-site and allosteric EGFR inhibitors. Nat Commun. 2022;13(1):2530–2543. 35534503 10.1038/s41467-022-30258-y PMC9085736

[21] Saad MH, El-Moselhy TF, Nabaweya E-DS, Mehany AB, Belal A, Abourehab MA, Tawfik HO, El-Hamamsy MH. Discovery of new symmetrical and asymmetrical nitrile-containing 1, 4-dihydropyridine derivatives as dual kinases and P-glycoprotein inhibitors: synthesis, in vitro assays, and *in silico* studies. J Enzyme Inhib Med Chem. 2022;37(1):2489–2511. 36093880 10.1080/14756366.2022.2120478 PMC9481151

[22] Eldehna WM, El Hassab MA, Elsayed ZM, Al-Warhi T, Elkady H, Abo-Ashour MF, Abourehab MA, Eissa IH, Abdel-Aziz HA. Design, synthesis, *in vitro* biological assessment and molecular modeling insights for novel 3-(naphthalen-1-yl)-4, 5-dihydropyrazoles as anticancer agents with potential EGFR inhibitory activity. Sci Rep. 2022;12(1):12821. 35896557 10.1038/s41598-022-15050-8 PMC9329325

[23] Nafie MS, Kishk SM, Mahgoub S, Amer AM. Quinoline‐based thiazolidinone derivatives as potent cytotoxic and apoptosis‐inducing agents through EGFR inhibition. Chem Biol Drug Des. 2022;99(4):547–560. 34873844 10.1111/cbdd.13997

[24] Eissa IH, Yousef RG, Elkady H, Alsfouk AA, Alsfouk BA, Husein DZ, Ibrahim IM, Elkaeed EB, Metwaly AM. A new anticancer semisynthetic theobromine derivative targeting EGFR protein: CADDD study. Life. 2023;13(1):191. 36676140 10.3390/life13010191 PMC9867533

[25] Kumari L, Mazumder A, Pandey D, Yar MS, Kumar R, Mazumder R, Sarafroz M, Ahsan MJ, Kumar V, Gupta S, et al. Synthesis and biological potentials of quinoline analogues: a review of literature. Mini-Rev Organ Chem. 2019;16(7):653–688.

[26] Faidallah HM, Rostom SA, Asiri AM, Khan KA, Radwan MF, Asfour HZ. 3-Cyano-8-methyl-2-oxo-1, 4-disubstituted-1, 2, 5, 6, 7, 8-hexahydroquinolines: synthesis and biological evaluation as antimicrobial and cytotoxic agents. J Enzyme Inhib Med Chem. 2013;28(1):123–130. 22136527 10.3109/14756366.2011.637201

[27] Al-Said MS, Ghorab MM, Al-Dosari MS, Hamed MM. Synthesis and in vitro anticancer evaluation of some novel hexahydroquinoline derivatives having a benzenesulfonamide moiety. Eur J Med Chem. 2011;46(1):201–207. 21112675 10.1016/j.ejmech.2010.11.002

[28] Sabbagh OIE, Shabaan MA, Kadry HH, Al‐Din ES. Synthesis of new nonclassical acridines, quinolines, and quinazolines derived from dimedone for biological evaluation. Arch Pharm . 2010;343(9):519–527. 10.1002/ardp.200900296 20814944

[29] Shaheen MA, El-Emam AA, El-Gohary NS. Design, synthesis and biological evaluation of new series of hexahydroquinoline and fused quinoline derivatives as potent inhibitors of wild-type EGFR and mutant EGFR (L858R and T790M. Bioorg Chem. 2020;105:104274. 33339080 10.1016/j.bioorg.2020.104274

[30] Ranjbar S, Edraki N, Firuzi O, Khoshneviszadeh M, Miri R. 5-Oxo-hexahydroquinoline: an attractive scaffold with diverse biological activities. Mol Divers. 2019;23(2):471–508. 30390186 10.1007/s11030-018-9886-4

[31] Roskoski R. Small molecule inhibitors targeting the EGFR/ErbB family of protein-tyrosine kinases in human cancers. Pharmacol Res. 2019;139:395–411. 30500458 10.1016/j.phrs.2018.11.014

[32] Laudadio E, Mobbili G, Sorci L, Galeazzi R, Minnelli C. Mechanistic insight toward EGFR activation induced by ATP: role of mutations and water in ATP binding patterns. J Biomol Struct Dyn. 2023;41(14):6492–6501. 35968630 10.1080/07391102.2022.2108497

[33] Sabbah DA, Hajjo R, Sweidan K. Review on epidermal growth factor receptor (EGFR) structure, signaling pathways, interactions, and recent updates of EGFR inhibitors. Curr Top Med Chem. 2020;20(10):815–834. 32124699 10.2174/1568026620666200303123102

[34] To C, Beyett TS, Jang J, Feng WW, Bahcall M, Haikala HM, Shin BH, Heppner DE, Rana JK, Leeper BA, et al. An allosteric inhibitor against the therapy-resistant mutant forms of EGFR in non-small cell lung cancer. Nat Cancer. 2022;3(4):402–417. 35422503 10.1038/s43018-022-00351-8 PMC9248923

[35] Abdullah MiN, Ali Y, Abd Hamid S. Insights into the structure and drug design of benzimidazole derivatives targeting the epidermal growth factor receptor (EGFR). Chem Biol Drug Des. 2022;100(6):921–934. 34651438 10.1111/cbdd.13974

[36] Jiao X, Zhang Q, Zhang Y, Shao J, Ding L, Tang C, Feng B. Synthesis and biological evaluation of new series of quinazoline derivatives as EGFR/HER2 dual-target inhibitors. Bioorg Med Chem Lett. 2022;67:128703. 35364239 10.1016/j.bmcl.2022.128703

[37] Zubair T, Bandyopadhyay D. Small molecule EGFR inhibitors as anti-cancer agents: discovery, mechanisms of action, and opportunities. Int J Mol Sci. 2023;24(3):2651–2676. 36768973 10.3390/ijms24032651 PMC9916655

[38] Todsaporn D, Mahalapbutr P, Poo-Arporn RP, Choowongkomon K, Rungrotmongkol T. Structural dynamics and kinase inhibitory activity of three generations of tyrosine kinase inhibitors against wild-type, L858R/T790M, and L858R/T790M/C797S forms of EGFR. Comput Biol Med. 2022;147:105787. 35803080 10.1016/j.compbiomed.2022.105787

[39] Aly RM, Serya RA, El-Motwally AM, Esmat A, Abbas S, Abou El Ella DA. Novel quinoline-3-carboxamides (part 2): design, optimization and synthesis of quinoline based scaffold as EGFR inhibitors with potent anticancer activity. Bioorg Chem. 2017;75:368–392. 29096097 10.1016/j.bioorg.2017.10.018

[40] Gaber AA, Morsy AME, ‐ Sherbiny FF, Bayoumi AH, Gamal KME, ‐ Adl KE, ‐ Al‐Karmalawy AA, Ezz Eldin RR, Saleh MA, Abulkhair HS. Pharmacophore‐linked pyrazolo [3, 4‐d] pyrimidines as EGFR‐TK inhibitors: synthesis, anticancer evaluation, pharmacokinetics, and *in silico* mechanistic studies. Arch Pharm. 2021;354:e2100258. 10.1002/ardp.202100258 34467546

[41] Shaikh GM, Murahari M, Thakur S, Kumar MS, Yc M. Studies on ligand-based pharmacophore modeling approach in identifying potent future EGFR inhibitors. J Mol Graph Model. 2022;112:108114. 34979367 10.1016/j.jmgm.2021.108114

[42] Sangani CB, Makawana JA, Zhang X, Teraiya SB, Lin L, Zhu H-L. Design, synthesis and molecular modeling of pyrazole–quinoline–pyridine hybrids as a new class of antimicrobial and anticancer agents. Eur J Med Chem. 2014;76:549–557. 24607998 10.1016/j.ejmech.2014.01.018

[43] Rao K, Chai Z, Zhou P, Liu D, Sun Y, Yu F. Transition-metal-free approach to quinolines via direct oxidative cyclocondensation reaction of N, N-dimethyl enaminones with o-aminobenzyl alcohols. Front Chem. 2022;10:1008568. 36212061 10.3389/fchem.2022.1008568 PMC9532769

[44] Amorzesh H, Bayat M, Nasri S. Catalyst-free synthesis of highly functionalized triazole hexahydroquinoline carbohydrazide scaffolds via four-component cyclocondensation reaction. Mol Diversity. 2022;26:1–10. 10.1007/s11030-022-10592-5 36585569

[45] Kalhor S, Yarie M, Rezaeivala M, Zolfigol MA. Novel magnetic nanoparticles with morpholine tags as multirole catalyst for synthesis of hexahydroquinolines and 2-amino-4, 6-diphenylnicotinonitriles through vinylogous anomeric-based oxidation. Res Chem Intermed. 2019;45(6):3453–3480.

[46] Modha SG, Pöthig A, Dreuw A, Bach T. 6π] Photocyclization to cis-hexahydrocarbazol-4-ones: substrate modification, mechanism, and scope. J Org Chem. 2019;84(3):1139–1153. 30648858 10.1021/acs.joc.8b03144 PMC6362434

[47] Mousavi SR, Sereshti H, Rashidi Nodeh H, Foroumadi A. A novel and reusable magnetic nanocatalyst developed based on graphene oxide incorporated strontium nanoparticles for the facial synthesis of β‐enamino ketones under solvent‐free conditions. Appl Organometal Chem. 2019;33(1):e4644.

[48] Nonsuwan P, Matsugami A, Hayashi F, Hyon S-H, Matsumura K. Controlling the degradation of an oxidized dextran-based hydrogel independent of the mechanical properties. Carbohydr Polym. 2019;204:131–141. 30366524 10.1016/j.carbpol.2018.09.081

[49] Kumar AR, Selvaraj S, Jayaprakash K, Gunasekaran S, Kumaresan S, Devanathan J, Selvam K, Ramadass L, Mani M, Rajkumar P. Multi-spectroscopic (FT-IR, FT-Raman, 1H NMR and 13C NMR) investigations on syringaldehyde. J Mol Struct. 2021;1229:129490.

[50] Hussen AS, Pandey AP, Sharma A. Mechanochemical-(hand-grinding-) assisted domino synthesis of fused pyran-spirooxindoles under solvent‐and catalyst‐free condition. ChemistrySelect. 2018;3(41):11505–11509.

[51] Hansen PE, Vakili M, Kamounah FS, Spanget-Larsen J. NH stretching frequencies of intramolecularly hydrogen-bonded systems: an experimental and theoretical study. Molecules. 2021;26:1–19. 10.3390/molecules26247651 PMC8706864 34946735

[52] Shaldam M, Tawfik H, Elmansi H, Belal F, Yamaguchi K, Sugiura M, Magdy G. Synthesis, crystallographic, DNA binding, and molecular docking/dynamic studies of a privileged chalcone-sulfonamide hybrid scaffold as a promising anticancer agent. J Biomol Struct Dyn. 2022;40:1–15. 36310097 10.1080/07391102.2022.2138551

[53] Babar A, Saeed A, Fatima S, Bolte M, Arshad N, Parveen U, Hökelek T, El-Seedi HR. Synthesis, X-ray, DFT, Hirshfeld surface analysis, molecular docking, urease inhibition, antioxidant, cytotoxicity, DNA protection, and DNA binding properties of 5-(tert-butyl)-N-(2, 4-dichlorophenyl)-1 H-1, 2, 4-triazol-3-amine. Struct Chem. 2023;34:1–17.

[54] Mishnev A, Bisenieks E, Mandrika I, Petrovska R, Kalme Z, Bruvere I, Duburs G. Crystal structure and metabolic activity of 4-(thien-2-yl)-2-methyl-5-oxo-1, 4, 5, 6, 7, 8-hexahydroquinoline-3-carboxylic acid ethoxycarbonylphenylmethylester. Acta Crystallogr E Crystallogr Commun. 2018;74(Pt 11):1577–1579. 30443384 10.1107/S2056989018014251 PMC6218899

[55] Gündüz MG, Armaković SJ, Dengiz C, Tahir MN, Armaković S. Crystal structure determination and computational studies of 1, 4-dihydropyridine derivatives as selective T-type calcium channel blockers. J Mol Struct. 2021;1230:129898.

[56] Tawfik HO, Petreni A, Supuran CT, El-Hamamsy MH. Discovery of new carbonic anhydrase IX inhibitors as anticancer agents by toning the hydrophobic and hydrophilic rims of the active site to encounter the dual-tail approach. Eur J Med Chem. 2022;232:114190. 35182815 10.1016/j.ejmech.2022.114190

[57] Mar’yasov M, Sheverdov V, Davydova V, Nasakin O. Antiproliferative activity of cyano-substituted pyrans and 1, 2, 5, 6, 7, 8-hexahydroquinoline-3, 3, 4, 4-tetracarbonitriles. Pharm Chem J. 2017;50(12):798–799.

[58] Kim E, Chung Y. Feasibility study of deep learning based radiosensitivity prediction model of National Cancer Institute-60 cell lines using gene expression. Nuclear Eng Technol. 2022;54(4):1439–1448.

[59] Ciftci H, Sever B, Ocak F, Bayrak N, Yıldız M, Yıldırım H, DeMirci H, Tateishi H, Otsuka M, Fujita M. In vitro and in silico study of analogs of plant product plastoquinone to be effective in colorectal cancer treatment. Molecules. 2022;27:693–711. 35163957 10.3390/molecules27030693 PMC8839215

[60] Sonousi A, Hassan RA, Osman EO, Abdou AM, Emam SH. Design and synthesis of novel quinazolinone-based derivatives as EGFR inhibitors with antitumor activity. J Enzyme Inhib Med Chem. 2022;37(1):2644–2659. 36146940 10.1080/14756366.2022.2118735 PMC9518264

[61] Aboukhatwa SM, Sidhom PA, Angeli A, Supuran CT, Tawfik HO. Terminators or guardians? Design, synthesis, and cytotoxicity profiling of chalcone-sulfonamide hybrids. ACS Omega. 2023;8(8):7666–7683. 36872984 10.1021/acsomega.2c07285 PMC9979347

[62] Zhou Y, Xiang S, Yang F, Lu X. Targeting gatekeeper mutations for kinase drug discovery. J Med Chem. 2022;65(23):15540–15558. 36395392 10.1021/acs.jmedchem.2c01361

[63] Wang J, Wu L. An evaluation of aumolertinib for the treatment of EGFR T790M mutation-positive non-small cell lung cancer. Expert Opin Pharmacother. 2022;23(6):647–652. 35272542 10.1080/14656566.2022.2050213

[64] Gad MM, Abdelwaly A, Helal MA. Structural basis for the selectivity of 3rd generation EGFR inhibitors: a molecular dynamics study. J Biomol Struct Dyn. 2023;41(13):6134–6144. 35903965 10.1080/07391102.2022.2103028

[65] You L, Zheng X, Deng D, Pan H, Han W. The benefit of anti-angiogenic therapy in EGFR exon 21 L858R mutant non-small cell lung cancer patients: a retrospective study. Sci Rep. 2022;12(1):14624. 36028744 10.1038/s41598-022-18889-z PMC9418331

[66] Yun CH, Boggon TJ, Li Y, Woo MS, Greulich H, Meyerson M, Eck MJ. Structures of lung cancer-derived EGFR mutants and inhibitor complexes: mechanism of activation and insights into differential inhibitor sensitivity. Cancer Cell. 2007;11(3):217–227. 17349580 10.1016/j.ccr.2006.12.017 PMC1939942

[67] Kawashima Y, Fukuhara T, Saito H, Furuya N, Watanabe K, Sugawara S, Iwasawa S, Tsunezuka Y, Yamaguchi O, Okada M, et al. Bevacizumab plus erlotinib versus erlotinib alone in Japanese patients with advanced, metastatic, EGFR-mutant non-small-cell lung cancer (NEJ026): overall survival analysis of an open-label, randomised, multicentre, phase 3 trial. Lancet Respir Med. 2022;10(1):72–82. 34454653 10.1016/S2213-2600(21)00166-1

[68] Huang CH, Ju JS, Chiu TH, Huang ACC, Tung PH, Wang CC, Liu CY, Chung FT, Fang YF, Guo YK, et al. Afatinib treatment in a large real‐world cohort of nonsmall cell lung cancer patients with common and uncommon epidermal growth factor receptor mutation. Int J Cancer. 2022;150(4):626–635. 34558665 10.1002/ijc.33821

[69] Kenessey I, Kramer Z, István L, Cserepes MT, Garay T, Hegedűs B, Dobos J, Tímár J, Tóvári J. Inhibition of epidermal growth factor receptor improves antitumor efficacy of vemurafenib in BRAF-mutant human melanoma in preclinical model. Melanoma Res. 2018;28(6):536–546. 30124539 10.1097/CMR.0000000000000488

[70] Xuhong JC, Qi XW, Zhang Y, Jiang J. Mechanism, safety and efficacy of three tyrosine kinase inhibitors lapatinib, neratinib and pyrotinib in HER2-positive breast cancer. Am J Cancer Res. 2019;9:2103–2119. 31720077 PMC6834479

[71] Necchi A, Lo Vullo S, Perrone F, Raggi D, Giannatempo P, Calareso G, Nicolai N, Piva L, Biasoni D, Catanzaro M, et al. First‐line therapy with dacomitinib, an orally available pan‐HER tyrosine kinase inhibitor, for locally advanced or metastatic penile squamous cell carcinoma: results of an open‐label, single‐arm, single‐centre, phase 2 study. BJU Int. 2018;121(3):348–356. 28921872 10.1111/bju.14013

[72] Takeda M, Nakagawa K. First-and second-generation EGFR-TKIs are all replaced to osimertinib in chemo-naive EGFR mutation-positive non-small cell lung cancer? Int J Mol Sci. 2019;20:146–164. 30609789 10.3390/ijms20010146 PMC6337322

[73] Lazzari C, Gregorc V, Karachaliou N, Rosell R, Santarpia M. Mechanisms of resistance to osimertinib. J Thorac Dis. 2020;12(5):2851–2858. 32642198 10.21037/jtd.2019.08.30 PMC7330330

[74] Murtuza A, Bulbul A, Shen JP, Keshavarzian P, Woodward BD, Lopez-Diaz FJ, Lippman SM, Husain H. Novel third-generation EGFR tyrosine kinase inhibitors and strategies to overcome therapeutic resistance in lung cancer. Cancer Res. 2019;79(4):689–698. 30718357 10.1158/0008-5472.CAN-18-1281

[75] Zhang W, Fan YF, Cai CY, Wang JQ, Teng QX, Lei ZN, Zeng L, Gupta P, Chen ZS. Olmutinib (BI1482694/HM61713), a novel epidermal growth factor receptor tyrosine kinase inhibitor, reverses ABCG2-mediated multidrug resistance in cancer cells. Front Pharmacol. 2018;9:1097. 1–13. 30356705 10.3389/fphar.2018.01097 PMC6189370

[76] Zhao S, Liu J, Lv Z, Zhang G, Xu Z. Recent updates on 1,2,3-triazole-containing hybrids with in vivo therapeutic potential against cancers: a mini-review. Eur J Med Chem. 2023;251:115254. 36893627 10.1016/j.ejmech.2023.115254

[77] Kesavan MP, Ravi L, Balachandran C, Thangadurai TD, Aoki S, Webster TJ, Rajesh J. Promising anticancer activity with high selectivity of DNA/plasma protein targeting new phthalazin-1(2H)-one heterocyclic scaffolds. J Mol Struct. 2023;1274:134423. 1–12.

[78] El-Malah A, Taher ES, Angeli A, Elbaramawi SS, Mahmoud Z, Moustafa N, Supuran CT, Ibrahim TS. Schiff bases as linker in the development of quinoline-sulfonamide hybrids as selective cancer-associated carbonic anhydrase isoforms IX/XII inhibitors: a new regioisomerism tactic. Bioorg Chem. 2023;131:106309. 36502567 10.1016/j.bioorg.2022.106309

[79] He X, Chen J, Wei L, Kandawa-Shultz M, Shao G, Wang Y. Antitumor activity of iridium/ruthenium complexes containing nitro-substituted quinoline ligands *in vivo* and *in vitro*. Dyes Pigm. 2023;213:111146. 10.1039/d2dt03317h 36942609

[80] Cao TQ, An HX, Ma RJ, Dai KY, Ji HY, Liu AJ, Zhou JP. Structural characteristics of a low molecular weight velvet antler protein and the anti-tumor activity on S180 tumor-bearing mice. Bioorg Chem. 2023;131:106304. 36463590 10.1016/j.bioorg.2022.106304

[81] Hammouda MM, Elmaaty AA, Nafie MS, Abdel-Motaal M, Mohamed NS, Tantawy MA, Belal A, Alnajjar R, Eldehna WM, Al‐Karmalawy AA. Design and synthesis of novel benzoazoninone derivatives as potential CBSIs and apoptotic inducers: in vitro, in vivo, molecular docking, molecular dynamics, and SAR studies. Bioorg Chem. 2022;127:105995. 35792315 10.1016/j.bioorg.2022.105995

[82] Amin MM, Abuo-Rahma GEDA, Shaykoon MSA, Marzouk AA, Abourehab MA, Saraya RE, Badr M, Sayed AM, Beshr EA. Design, synthesis, cytotoxic activities, and molecular docking of chalcone hybrids bearing 8-hydroxyquinoline moiety with dual tubulin/EGFR kinase inhibition. Bioorg Chem. 2023;134:106444. 36893547 10.1016/j.bioorg.2023.106444

[83] Hegedüs L, Okumus Ö, Mairinger F, Ploenes T, Reuter S, Schuler M, Welt A, Vega-Rubin-de-Celis S, Theegarten D, Bankfalvi A, et al. TROP2 expression and SN38 antitumor activity in malignant pleural mesothelioma cells provide a rationale for antibody-drug conjugate therapy. Lung Cancer. 2023;178:237–246. 36907051 10.1016/j.lungcan.2023.03.003

[84] Hinterleitner C, Strähle J, Malenke E, Hinterleitner M, Henning M, Seehawer M, Bilich T, Heitmann J, Lutz M, Mattern S, et al. Platelet PD-L1 reflects collective intratumoral PD-L1 expression and predicts immunotherapy response in non-small cell lung cancer. Nat Commun. 2021;12(1):7005. 34853305 10.1038/s41467-021-27303-7 PMC8636618

[85] Noser AA, Abdelmonsef AH, Salem MM. Design, synthesis and molecular docking of novel substituted azepines as inhibitors of PI3K/Akt/TSC2/mTOR signaling pathway in colorectal carcinoma. Bioorg Chem. 2023;131:106299. 36493622 10.1016/j.bioorg.2022.106299

[86] Ma K, Wang Z, Ju X, Huang J, He R. Rapeseed peptide inhibits HepG2 cell proliferation by regulating the mitochondrial and P53 signaling pathways. J Sci Food Agric. 2023;103(3):1474–1483. 36168817 10.1002/jsfa.12243

[87] Teleb WK, Tantawy MA, Xu X, Hussein AA, Abdel-Rahman MA. Cytotoxicity and molecular alterations induced by scorpion venom antimicrobial peptide Smp43 in breast cancer cell lines MDA-MB-231 and MCF-7. Int J Pept Res Ther. 2022;29(1):15.

[88] Swedan HK, Kassab AE, Gedawy EM, Elmeligie SE. Design, synthesis, and biological evaluation of novel ciprofloxacin derivatives as potential anticancer agents targeting topoisomerase II enzyme. J Enzyme Inhib Med Chem. 2023;38(1):118–137. 36305290 10.1080/14756366.2022.2136172 PMC9635472

[89] Mir SA, Muhammad A, Padhiary A, Ekka NJ, Baitharu I, Naik PK, Nayak B. Identification of potent EGFR-TKD inhibitors from NPACT database through combined computational approaches. J Biomol Struct Dyn. 2023;41:1–14. 36695102 10.1080/07391102.2023.2171133

[90] Khan S, Hussain A, Asif M, Sattar FA, Audhal FA, Qadir MI, Hamdard MH., Nasrullah In-silico studies of inhibitory compounds against protease enzymes of SARS-CoV-2. Medicine . 2023;102(6):e31318. 36820539 10.1097/MD.0000000000031318 PMC9907915

[91] Musa A, Ihmaid SK, Hughes DL, Said MA, Abulkhair HS, El-Ghorab AH, Abdelgawad MA, Shalaby K, Shaker ME, Alharbi KS, et al. The anticancer and EGFR-TK/CDK-9 dual inhibitory potentials of new synthetic pyranopyrazole and pyrazolone derivatives: x-ray crystallography, *in vitro*, and *in silico* mechanistic investigations. J Biomol Struct Dyn. 2023;41:1–15. 36661285 10.1080/07391102.2023.2167000

[92] Kardile RA, Sarkate AP, Lokwani DK, Tiwari SV, Azad R, Thopate SR. Thopate, Design, synthesis, and biological evaluation of novel quinoline derivatives as small molecule mutant EGFR inhibitors targeting resistance in NSCLC: *in vitro* screening and ADME predictions. Eur J Med Chem. 2023;245(Pt 1):114889. 36375337 10.1016/j.ejmech.2022.114889

[93] Adel M, Abouzid KA. New fluorinated diarylureas linked to pyrrolo [2, 3-d] pyrimidine scaffold as VEGFR-2 inhibitors: molecular docking and biological evaluation. Bioorg Chem. 2022;127:106006. 35820328 10.1016/j.bioorg.2022.106006

[94] Paul SK, Dutta Chowdhury K, Dey SR, Paul A, Haldar R. Exploring the possibility of drug repurposing for cancer therapy targeting human lactate dehydrogenase A: a computational approach. J Biomol Struct Dyn. 2022;40:1–10. 36576127 10.1080/07391102.2022.2158134

[95] El-Gazzar YI, Ghaiad HR, El Kerdawy AM, George RF, Georgey HH, Youssef KM, El‐Subbagh HI. New quinazolinone‐based derivatives as DHFR/EGFR‐TK inhibitors: synthesis, molecular modeling simulations, and anticancer activity. Arch Pharm . 2023;356(1):e2200417. 10.1002/ardp.202200417 36257809

[96] Songtawee N, Gleeson MP, Choowongkomon K. Computational study of EGFR inhibition: molecular dynamics studies on the active and inactive protein conformations. J Mol Model. 2013;19(2):497–509. 22955422 10.1007/s00894-012-1559-0

[97] Ahmad I, Shaikh M, Surana S, Ghosh A, Patel H. p38α MAP kinase inhibitors to overcome EGFR tertiary C797S point mutation associated with osimertinib in non-small cell lung cancer (NSCLC): emergence of fourth-generation EGFR inhibitor. J Biomol Struct Dyn. 2022;40(7):3046–3059. 33174519 10.1080/07391102.2020.1844801

[98] Al-Warhi T, El Kerdawy AM, Said MA, Albohy A, Elsayed ZM, Aljaeed N, Elkaeed EB, Eldehna WM, Abdel-Aziz HA, Abdelmoaz MA. Novel 2-(5-Aryl-4, 5-dihydropyrazol-1-yl) thiazol-4-one as EGFR inhibitors: synthesis, biological assessment and molecular docking insights. Drug Des Devel Ther. 2022;16:1457–1471. 10.2147/DDDT.S356988 PMC9123247 35607598

[99] Ghorab WM, El-Sebaey SA, Ghorab MM. Design, synthesis and molecular modeling study of certain EGFR inhibitors with a quinazolinone scaffold as anti-hepatocellular carcinoma and Radio-sensitizers. Bioorg Chem. 2023;131:106310. 36528923 10.1016/j.bioorg.2022.106310

[100] Shao J, Zhu K, Du D, Zhang Y, Tao H, Chen Z, Jiang H, Chen K, Luo C, Duan W. Discovery of 2-substituted-N-(3-(3, 4-dihydroisoquinolin-2 (1H)-yl)-2-hydroxypropyl)-1, 2, 3, 4-tetrahydroisoquinoline-6-carboxamide as potent and selective protein arginine methyltransferases 5 inhibitors: design, synthesis and biological evaluation. Eur J Med Chem. 2019;164:317–333. 30605830 10.1016/j.ejmech.2018.12.065

[101] Bugge S, Moen IU, Sylte KOK, Sundby E, Hoff BH. Truncated structures used in search for new lead compounds and in a retrospective analysis of thienopyrimidine-based EGFR inhibitors. Eur J Med Chem. 2015;94:175–194. 25768701 10.1016/j.ejmech.2015.03.004

[102] Zhang C, Pei H, He J, Zhu J, Li W, Niu T, Xiang M, Chen L. Design, synthesis and evaluation of novel 7H-pyrrolo [2, 3-d] pyrimidin-4-amine derivatives as potent, selective and reversible Bruton’s tyrosine kinase (BTK) inhibitors for the treatment of rheumatoid arthritis. Eur J Med Chem. 2019;169:121–143. 30875504 10.1016/j.ejmech.2019.02.077

[103] Tawfik HO, El-Moselhy TF, El-Din NS, El-Hamamsy MH. Design, synthesis, and bioactivity of dihydropyrimidine derivatives as kinesin spindle protein inhibitors. Bioorg Med Chem. 2019;27(23):115126. 31648875 10.1016/j.bmc.2019.115126

[104] Chinen AB, Guan CM, Ferrer JR, Barnaby SN, Merkel TJ, Mirkin CA. Nanoparticle probes for the detection of cancer biomarkers, cells, and tissues by fluorescence. Chem Rev. 2015;115(19):10530–10574. 26313138 10.1021/acs.chemrev.5b00321 PMC5457709

[105] Sordon S, Popłoński J, Milczarek M, Stachowicz M, Tronina T, Kucharska AZ, Wietrzyk J, Huszcza E. Structure–antioxidant–antiproliferative activity relationships of natural C7 and C7–C8 hydroxylated flavones and flavanones. Antioxidants. 2019;8(7):210. 31284642 10.3390/antiox8070210 PMC6680932

[106] Nasser AA, Eissa IH, Oun MR, El-Zahabi MA, Taghour MS, Belal A, Saleh AM, Mehany ABM, Luesch H, Mostafa AE, et al. Discovery of new pyrimidine-5-carbonitrile derivatives as anticancer agents targeting EGFR WT and EGFR T790M. Org Biomol Chem. 2020;18(38):7608–7634. 32959865 10.1039/d0ob01557a

[107] Mghwary AE-S, Gedawy EM, Kamal AM, Abuel-Maaty SM. Novel thienopyrimidine derivatives as dual EGFR and VEGFR-2 inhibitors: design, synthesis, anticancer activity and effect on cell cycle profile. J Enzyme Inhib Med Chem. 2019;34(1):838–852. 30919701 10.1080/14756366.2019.1593160 PMC6442109

[108] Behzadi M, Eghtedardoost M, Bagheri M. Endocytosis involved d-oligopeptide of tryptophan and arginine displays ordered nanostructures and cancer cell stereoselective toxicity by autophagy. ACS Appl Mater Interfaces. 2022;14(13):14928–14943. 35319877 10.1021/acsami.1c23846

[109] Mir SA, Dash GC, Meher RK, Mohanta PP, Chopdar KS, Mohapatra PK, Baitharu I, Behera AK, Raval MK, Nayak B. *In silico* and *in vitro* evaluations of fluorophoric thiazolo-[2, 3-b] quinazolinones as anti-cancer agents targeting EGFR-TKD. Appl Biochem Biotechnol. 2022;194(10):4292–4318. 35366187 10.1007/s12010-022-03893-w

[110] Ezelarab HA, Ali TF, Abbas SH, Sayed AM, Beshr EA, Hassan HA. New antiproliferative 3-substituted oxindoles inhibiting EGFR/VEGFR-2 and tubulin polymerization. Mol Divers. 2023;27:1–18. 36790582 10.1007/s11030-023-10603-z PMC11070402

