# Supplementary material for: Recruitment of hexahydroquinoline as anticancer scaffold targeting inhibition of wild and mutants EGFR (EGFRWT, EGFRT790M, and EGFRL858R)
Source: J Enzyme Inhib Med Chem. 2023 Aug 7;38(1):2241674. doi: 10.1080/14756366.2023.2241674 (PMC10408569; doi:10.1080/14756366.2023.2241674)
Supplement: Supplemental Material [file IENZ_A_2241674_SM6031.pdf]

Supplementary file

Recruitment of hexahydroquinoline as anticancer scaffold targeting inhibition of wild and mutants EGFR ($EGFR^{WT}$, $EGFR^{T790M}$, and $EGFR^{L858R}$)

Mahmoud G. Abo Al-Hamd, Haytham O. Tawfik, Omeima Abdullah, Koki Yamaguchi, Masaharu Sugiura, Ahmed B. M. Mehany, Mervat H. El-Hamamsy, Tarek F. El-Moselhy

1. **Mahmoud G. Abo Al-Hamd**
   Instructor, Department of Pharmaceutical Chemistry,
   Faculty of Pharmacy, Tanta University, El Giesh street, Tanta, 31527, Egypt
   E-mail: mahmoud.gamal@pharm.tanta.edu.eg

2. **Haytham O. Tawfik, PhD**
   Assistant Professor, Department of Pharmaceutical Chemistry,
   Faculty of Pharmacy, Tanta University, El Giesh street, Tanta, 31527, Egypt.
   E-mail: haytham.omar.mahmoud@pharm.tanta.edu.eg

3. **Omeima Abdullah, PhD**
   Assistant Professor, Pharmaceutical Chemistry Department, College of Pharmacy, Umm Al-Qura University.
   E-mail: oaabdullah@uqu.edu.sa

4. **Koki Yamaguchi, PhD**
   Professor, Faculty of Pharmaceutical Sciences, Sojo University, Kumamoto, Japan
   E-mail: kyamag05@ph.sojo-u.ac.jp

5. **Masaharu Sugiura, PhD**
   Professor, Faculty of Pharmaceutical Sciences, Sojo University, Kumamoto, Japan
   E-mail: msugiura@ph.sojo-u.ac.jp

6. **Ahmed B. M. Mehany**
   Zoology Department, Faculty of Science, Al-Azhar University, Cairo, Egypt
   E-mail: abelal_81@yahoo.com

7. **Mervat H. El-Hamamsy, PhD**
   Associate Professor, Department of Pharmaceutical Chemistry,
   Faculty of Pharmacy, Tanta University, El Giesh street, Tanta, 31527, Egypt.
   E-mail: mhamamsy@pharm.tanta.edu.eg

8. **Tarek F. El-Moselhy, PhD**
   Professor, Department of Pharmaceutical Chemistry,
   Faculty of Pharmacy, Tanta University, El Giesh street, Tanta, 31527, Egypt.
   E-mail: tarek.faathy@pharm.tanta.edu.eg

Corresponding authors:

Mahmoud G. Abo Al-Hamd: E-mail: mahmoud.gamal@pharm.tanta.edu.eg

ORCID: 0000-0001-7736-6949

Haytham O. Tawfik: E-mail: haytham.omar.mahmoud@pharm.tanta.edu.eg

ORCID: 0000-0001-6455-5716

Table of Contents

Title	Page
^{1}H NMR, ^{13}C NMR, Mass spectral data of the target compounds (**6a-i**), (**8a-m**), (**10a-d**) and (**12a-f**) and IR spectral data of compounds (**10c-d**)	
**Figure S1.** ^{1}H NMR (400 MHz, DMSO-d_6) spectrum of compound **6a**	**S6**
**Figure S2**. ^{13}C NMR (100 MHz, $CDCl_3$) spectrum of compound **6a**	**S6**
**Figure S3.** Mass spectrum (positive mode) of compound **6a**	**S7**
**Figure S4.** ^{1}H NMR (400 MHz, $CDCl_3$) spectrum of compound **6b**	**S8**
**Figure S5**. ^{13}C NMR (100 MHz, $CDCl_3$) spectrum of compound **6b**	**S8**
**Figure S6.** Mass spectrum (positive mode) of compound **6b**	**S9**
**Figure S7.** ^{1}H NMR (400 MHz, $CDCl_3$) spectrum of compound **6c**	**S10**
**Figure S8**. ^{13}C NMR (100 MHz, $CDCl_3$) spectrum of compound **6c**	**S10**
**Figure S9.** Mass spectrum (positive mode) of compound **6c**	**S11**
**Figure S10.** ^{1}H NMR (400 MHz, DMSO-d_6) spectrum of compound **6d**	**S12**
**Figure S11.** ^{13}C NMR (100 MHz, $CDCl_3$) spectrum of compound **6d**	**S12**
**Figure S12.** Mass spectrum (positive mode) of compound **6d**	**S13**
**Figure S13.** ^{1}H NMR (400 MHz, $CDCl_3$) spectrum of compound **6e**	**S14**
**Figure S14.** ^{13}C NMR (100 MHz, $CDCl_3$) spectrum of compound **6e**	**S14**
**Figure S15.** Mass spectrum (positive mode) of compound **6e**	**S15**
**Figure S16.** ^{1}H NMR (400 MHz, $CDCl_3$) spectrum of compound **6f**	**S16**
**Figure S17.** ^{13}C NMR (100 MHz, $CDCl_3$) spectrum of compound **6f**	**S16**
**Figure S18.** Mass spectrum (positive mode) of compound **6f**	**S17**
**Figure S19.** ^{1}H NMR (400 MHz, $CDCl_3$) spectrum of compound **6g**	**S18**
**Figure S20.** ^{13}C NMR (100 MHz, $CDCl_3$) spectrum of compound **6g**	**S18**
**Figure S21.** Mass spectrum (positive mode) of compound **6g**	**S19**
**Figure S22.** ^{1}H NMR (400 MHz, $CDCl_3$) spectrum of compound **6h**	**S20**
**Figure S23.** ^{13}C NMR (100 MHz, $CDCl_3$) spectrum of compound **6h**	**S20**
**Figure S24.** Mass spectrum (positive mode) of compound **6h**	**S21**
**Figure S25.** ^{1}H NMR (400 MHz, $CDCl_3$) spectrum of compound **6i**	**S22**
**Figure S26.** ^{13}C NMR (100 MHz, $CDCl_3$) spectrum of compound **6i**	**S22**
**Figure S27.** Mass spectrum (positive mode) of compound **6i**	**S23**
**Figure S28.** ^{1}H NMR (400 MHz, $CDCl_3$) spectrum of compound **8a**	**S24**
**Figure S29.** ^{13}C NMR (100 MHz, $CDCl_3$) spectrum of compound **8a**	**S24**
**Figure S30.** Mass spectrum (positive mode) of compound **8a**	**S25**
**Figure S31.** ^{1}H NMR (400 MHz, $CDCl_3$) spectrum of compound **8b**	**S26**
**Figure S32.** ^{13}C NMR (100 MHz, $CDCl_3$) spectrum of compound **8b**	**S26**
**Figure S33.** Mass spectrum (positive mode) of compound **8b**	**S27**
**Figure S34.** ^{1}H NMR (400 MHz, $CDCl_3$) spectrum of compound **8c**	**S28**
**Figure S35.** ^{13}C NMR (100 MHz, $CDCl_3$) spectrum of compound **8c**	**S28**
**Figure S36.** Mass spectrum (positive mode) of compound **8c**	**S29**
**Figure S37.** ^{1}H NMR (400 MHz, $CDCl_3$) spectrum of compound **8d**	**S30**
**Figure S38.** ^{13}C NMR (100 MHz, $CDCl_3$) spectrum of compound **8d**	**S30**
**Figure S39.** Mass spectrum (positive mode) of compound **8d**	**S31**
**Figure S40.** ^{1}H NMR (400 MHz, $CDCl_3$) spectrum of compound **8e**	**S32**
**Figure S41.** ^{13}C NMR (100 MHz, $CDCl_3$) spectrum of compound **8e**	**S32**
**Figure S42.** Mass spectrum (positive mode) of compound **8e**	**S33**
**Figure S43.** ^{1}H NMR (400 MHz, $CDCl_3$) spectrum of compound **8f**	**S34**
**Figure S44.** ^{13}C NMR (100 MHz, $CDCl_3$) spectrum of compound **8f**	**S34**
**Figure S45.** Mass spectrum (positive mode) of compound **8f**	**S35**
**Figure S46.** ^{1}H NMR (400 MHz, $CDCl_3$) spectrum of compound **8g**	**S36**
**Figure S47.** ^{13}C NMR (100 MHz, $CDCl_3$) spectrum of compound **8g**	**S36**

**Figure S48.** Mass spectrum (positive mode) of compound **8g**	**S37**
**Figure S49.** ^{1}H NMR (400 MHz, $CDCl_3$) spectrum of compound **8h**	**S38**
**Figure S50.** ^{13}C NMR (100 MHz, $CDCl_3$) spectrum of compound **8h**	**S38**
**Figure S51.** Mass spectrum (positive mode) of compound **8h**	**S39**
**Figure S52.** ^{1}H NMR (400 MHz, $CDCl_3$) spectrum of compound **8i**	**S40**
**Figure S53.** ^{13}C NMR (100 MHz, $CDCl_3$) spectrum of compound **8i**	**S40**
**Figure S54.** Mass spectrum (positive mode) of compound **8i**	**S41**
**Figure S55.** ^{1}H NMR (400 MHz, $CDCl_3$) spectrum of compound **8j**	**S42**
**Figure S56.** ^{13}C NMR (100 MHz, $CDCl_3$) spectrum of compound **8j**	**S42**
**Figure S57.** Mass spectrum (positive mode) of compound **8j**	**S43**
**Figure S58.** ^{1}H NMR (400 MHz, $CDCl_3$) spectrum of compound **8k**	**S44**
**Figure S59.** ^{13}C NMR (100 MHz, $CDCl_3$) spectrum of compound **8k**	**S44**
**Figure S60.** Mass spectrum (positive mode) of compound **8k**	**S45**
**Figure S61.** ^{1}H NMR (400 MHz, DMSO-d_6) spectrum of compound **8l**	**S46**
**Figure S62.** ^{13}C NMR (100 MHz, $CDCl_3$) spectrum of compound **8l**	**S46**
**Figure S63.** Mass spectrum (positive mode) of compound **8l**	**S47**
**Figure S64.** ^{1}H NMR (400 MHz, $CDCl_3$) spectrum of compound **8m**	**S48**
**Figure S65.** ^{13}C NMR (100 MHz, $CDCl_3$) spectrum of compound **8m**	**S48**
**Figure S66.** Mass spectrum (positive mode) of compound **8m**	**S49**
**Figure S67.** ^{1}H NMR (400 MHz, $CDCl_3$) spectrum of compound **10a**	**S50**
**Figure S68.** ^{13}C NMR (100 MHz, $CDCl_3$) spectrum of compound **10a**	**S50**
**Figure S69.** Mass spectrum of compound **10a**	**S51**
**Figure S70.** ^{1}H NMR (400 MHz, $CDCl_3$) spectrum of compound **10b**	**S52**
**Figure S71.** ^{13}C NMR (100 MHz, $CDCl_3$) spectrum of compound **10b**	**S52**
**Figure S72.** Mass spectrum of compound **10b**	**S53**
**Figure S73.** ^{1}H NMR (400 MHz, $CDCl_3$) spectrum of compound **10c**	**S54**
**Figure S74.** ^{13}C NMR (100 MHz, $CDCl_3$) spectrum of compound **10c**	**S54**
**Figure S75.** Mass spectrum of compound **10c**	**S55**
**Figure S76.** IR spectrum of compound **10c**	**S55**
**Figure S77.** ^{1}H NMR (400 MHz, $CDCl_3$) spectrum of compound **10d**	**S56**
**Figure S78.** ^{13}C NMR (100 MHz, $CDCl_3$) spectrum of compound **10d**	**S56**
**Figure S79.** Mass spectrum of compound **10d**	**S57**
**Figure S80.** IR spectrum of compound **10d**	**S57**
**Figure S81.** ^{1}H NMR (400 MHz, DMSO-d_6) spectrum of compound **12a**	**S58**
**Figure S82.** ^{13}C NMR (100 MHz, $CDCl_3$) spectrum of compound **12a**	**S58**
**Figure S83.** Mass spectrum (positive mode) of compound **12a**	**S59**
**Figure S84.** ^{1}H NMR (400 MHz, $CDCl_3$) spectrum of compound **12b**	**S60**
**Figure S85.** ^{13}C NMR (100 MHz, $CDCl_3$) spectrum of compound **12b**	**S60**
**Figure S86.** Mass spectrum (positive mode) of compound **12b**	**S61**
**Figure S87.** ^{1}H NMR (400 MHz, $CDCl_3$) spectrum of compound **12c**	**S62**
**Figure S88.** ^{13}C NMR (100 MHz, $CDCl_3$) spectrum of compound **12c**	**S62**
**Figure S89.** Mass spectrum (positive mode) of compound **12c**	**S63**
**Figure S90.** ^{1}H NMR (400 MHz, $CDCl_3$) spectrum of compound **12d**	**S64**
**Figure S91.** ^{13}C NMR (100 MHz, $CDCl_3$) spectrum of compound **12d**	**S64**
**Figure S92.** Mass spectrum (positive mode) of compound **12d**	**S65**
**Figure S93.** ^{1}H NMR (400 MHz, $CDCl_3$) spectrum of compound **12e**	**S66**
**Figure S94.** ^{13}C NMR (100 MHz, $CDCl_3$) spectrum of compound **12e**	**S66**
**Figure S95.** Mass spectrum (positive mode) of compound **12e**	**S67**
**Figure S96.** ^{1}H NMR (400 MHz, $CDCl_3$) spectrum of compound **12f**	**S68**
**Figure S97.** ^{13}C NMR (100 MHz, $CDCl_3$) spectrum of compound **12f**	**S68**
**Figure S98.** Mass spectrum (positive mode) of compound **12f**	**S69**
**Biological Data**	
**Figure S99.** One dose mean graph for compound **6a** (NSC 832911) at 10 μM	**S70**

**Figure S100.** One dose mean graph for compound **6b** (NSC 833296) at 10 μM	**S71**
**Figure S101.** One dose mean graph for compound **6c** (NSC 833299) at 10 μM	**S72**
**Figure S102.** One dose mean graph for compound **6d** (NSC 832912) at 10 μM	**S73**
**Figure S103.** One dose mean graph for compound **6e** (NSC 832917) at 10 μM	**S74**
**Figure S104.** One dose mean graph for compound **6f** (NSC 833300) at 10 μM	**S75**
**Figure S105.** One dose mean graph for compound **6g** (NSC 835925) at 10 μM	**S76**
**Figure S106.** One dose mean graph for compound **6h** (NSC 835924) at 10 μM	**S77**
**Figure S107.** One dose mean graph for compound **6i** (NSC 835584) at 10 μM	**S78**
**Figure S108.** One dose mean graph for compound **8a** (NSC 832913) at 10 μM	**S79**
**Figure S109.** One dose mean graph for compound **8b** (NSC 833301) at 10 μM	**S80**
**Figure S110.** One dose mean graph for compound **8c** (NSC 835582) at 10 μM	**S81**
**Figure S111.** One dose mean graph for compound **8d** (NSC 833297) at 10 μM	**S82**
**Figure S112.** One dose mean graph for compound **8e** (NSC 834379) at 10 μM	**S83**
**Figure S113.** One dose mean graph for compound **8f** (NSC 832914) at 10 μM	**S84**
**Figure S114.** One dose mean graph for compound **8g** (NSC 832918) at 10 μM	**S85**
**Figure S115.** One dose mean graph for compound **8h** (NSC 833302) at 10 μM	**S86**
**Figure S116.** One dose mean graph for compound **8i** (NSC 832915) at 10 μM	**S87**
**Figure S117.** One dose mean graph for compound **8j** (NSC 832919) at 10 μM	**S88**
**Figure S118.** One dose mean graph for compound **8k** (NSC 835585) at 10 μM	**S89**
**Figure S119.** One dose mean graph for compound **8l** (NSC 832916) at 10 μM	**S90**
**Figure S120.** One dose mean graph for compound **8m** (NSC 833303) at 10 μM	**S91**
**Figure S121.** One dose mean graph for compound **10a** (NSC 838214) at 10 μM	**S92**
**Figure S122.** One dose mean graph for compound **10b** (NSC 838216) at 10 μM	**593**
**Figure S123.** One dose mean graph for compound **10c** (NSC 838215) at 10 μM	**S94**
**Figure S124.** Dose response curves (percentage growth versus concentration of compound at NCI fixed protocol, μM) for tested compound **10c** (NSC 838215) against the nine different panels of NCI cancer cell lines at five concentrations (from 10^{-4} mol/L to 10^{-8} mol/L.	**S95**
**Figure S125.** Cytotoxic activity of compound **10c** (NSC 838215) against the NCI 60 human cancer cell line at five doses.	**S96**
**Figure S126.** Mean graphs of $\log_{10}$ values (Molar) of GI_{50}, TGI and LC_{50} of compound **10c** (NSC 838215) obtained from the NCI 60 cell line experiments.	**S97**
**Figure S127.** Dose response curves (percentage growth versus concentration of compound at NCI fixed protocol, μM) for tested compound **10c** (NSC 838215) against the nine different panels of NCI cancer cell lines at five concentrations (from 10^{-4} mol/L to 10^{-8} mol/L).	**S97**
**Figure S128.** One dose mean graph for compound **10d** (NSC 838217) at 10 μM	**S98**
**Figure S129.** One dose mean graph for compound **12a** (NSC 832910) at 10 μM	**S99**
**Figure S130.** One dose mean graph for compound **12b** (NSC 833295) at 10 μM	**S100**
**Figure S131.** One dose mean graph for compound **12c** (NSC 833298) at 10 μM	**S101**
**Figure S132.** One dose mean graph for compound **12d** (NSC 835583) at 10 μM	**S102**
**Figure S133.** One dose mean graph for compound **12e** (NSC 835923) at 10 μM	**S103**
**Figure S134.** One dose mean graph for compound **12f** (NSC 834380) at 10 μM	**S104**
**Table S1.** GI % mean of the target compounds (**6a-i** and **8a-m**) across the NCI-60 human cancer cell line panel	**S105**
**Table S2.** GI % mean of the target compounds (**10a-d** and **12a-f**) across the NCI-60 human cancer cell line panel.	**S106**
**Molecular docking**	
**Table S3**. Validation of docking process by re-docking of the co-crystallized ligands of the three PDB codes (1M17, 2JIV and 4LQM) in the active sites	**S107**
**Table S4**. 2D and 3D docking pose of erlotinib in the active sites of PDB codes (1M17, 2JIV and 4LQM).	**S108**

**Table S5**. Docking scores of *R*-isomer of compound **10c** in the active sites of PDB codes (1M17, 2JIV and 4LQM)	**S109**
**Table S6**. Docking scores of *R*-isomer of compound **10d** in the active sites of PDB codes (1M17, 2JIV and 4LQM)	**S109**
**Figure S135**. 3D alignment of the *S*-enantiomer of compound **10c** (cyan) with the original ligand (erlotinib) (purple) (PDB code: 1M17).	**S110**
**Figure S136**. 3D alignment of the *S*-enantiomer of compound **10c (**cyan) with the original ligand (HKI) (purple) (PDB code: 2JIV).	**S110**
**Figure S137.** 3D alignment of the *S*-enantiomer of compound **10c (**cyan) with the original ligand (PD-168393**)** (purple) (PDB code: 4LQM.	**S110**
**Figure S138**. 3D alignment of the *S*-enantiomer of compound **10d** (cyan) with the original ligand (erlotinib) (purple) (PDB code: 1M17).	**S111**
**Figure S139**. 3D alignment of the *S*-enantiomer of compound **10d** (cyan) with the original ligand (HKI) (purple) (PDB code: 2JIV).	**S111**
**Figure S140**. 3D alignment of the *S*-enantiomer of compound **10d** (cyan) with the original ligand (PD-168393**)** (purple) (PDB code: 4LQM).	**S111**
**Figure S141**. The 2D (left) and 3D (right) pose for docking interactions of *R*-isomer of compound **10c** within the active site of EGFR (PDB code: 1M17).	**S112**
**Figure S142**. The 2D (left) and 3D (right) pose for docking interactions of *R*-isomer of compound **10c** within the active site of EGFR (PDB code: 2JIV).	**S112**
**Figure S143**. The 2D (left) and 3D (right) pose for docking interactions of *R*-isomer of compound **10c** within the active site of EGFR (PDB code: 4LQM).	**S113**
**Figure S144**. The 2D (left) and 3D (right) pose for docking interactions of *R*-isomer of compound **10d** within the active site of EGFR (PDB code: 1M17).	**S113**
**Figure S145**. The 2D (left) and 3D (right) pose for docking interactions of *R*-isomer of compound **10d** within the active site of EGFR (PDB code: 2JIV).	**S114**
**Figure S146**. The 2D (left) and 3D (right) pose for docking interactions of *R*-isomer of compound **10d** within the active site of EGFR (PDB code: 4LQM).	**S114**
**Experimental section**	
**X-ray crystallography**	**S115**
**Biology**	**S115**
In vitro preliminary anticancer activity at a single dose against 60 NCI cell lines	**S115**
Enzyme inhibition assay of $EGFR^{WT}$, $EGFR^{T790M}$, and $EGFR^{L858R}$	**S115**
Annexin V-FITC apoptosis assay	**S116**
Cellular mechanistic analysis	**S116**
**Docking protocol**	**S116**
**References**	**S117**

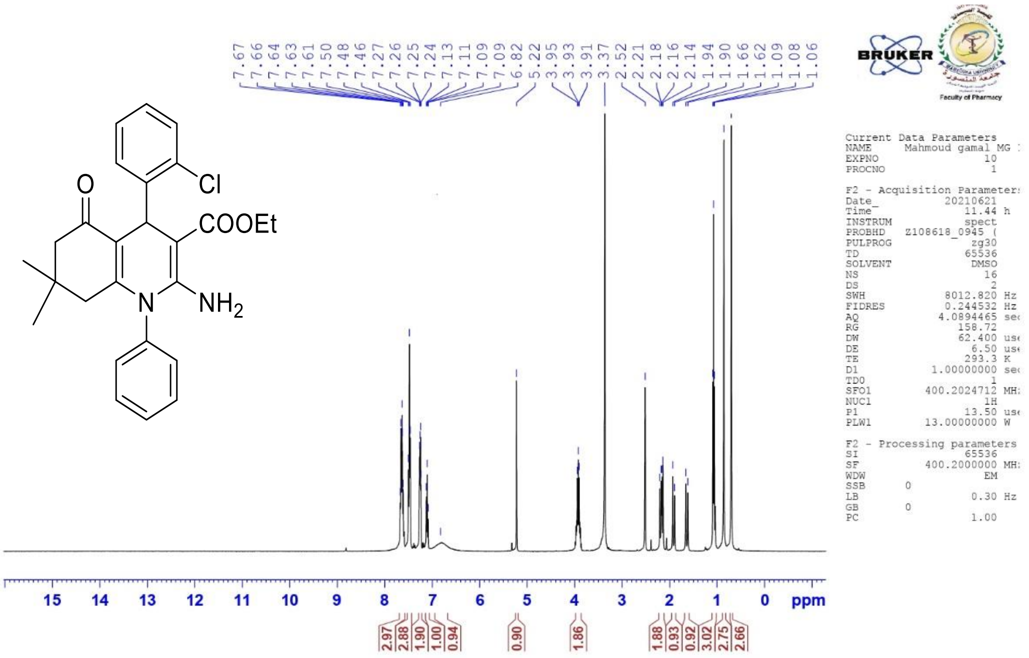

Figure S1. ^{1}H NMR (400 MHz, DMSO-d_6) spectrum of compound **6a**

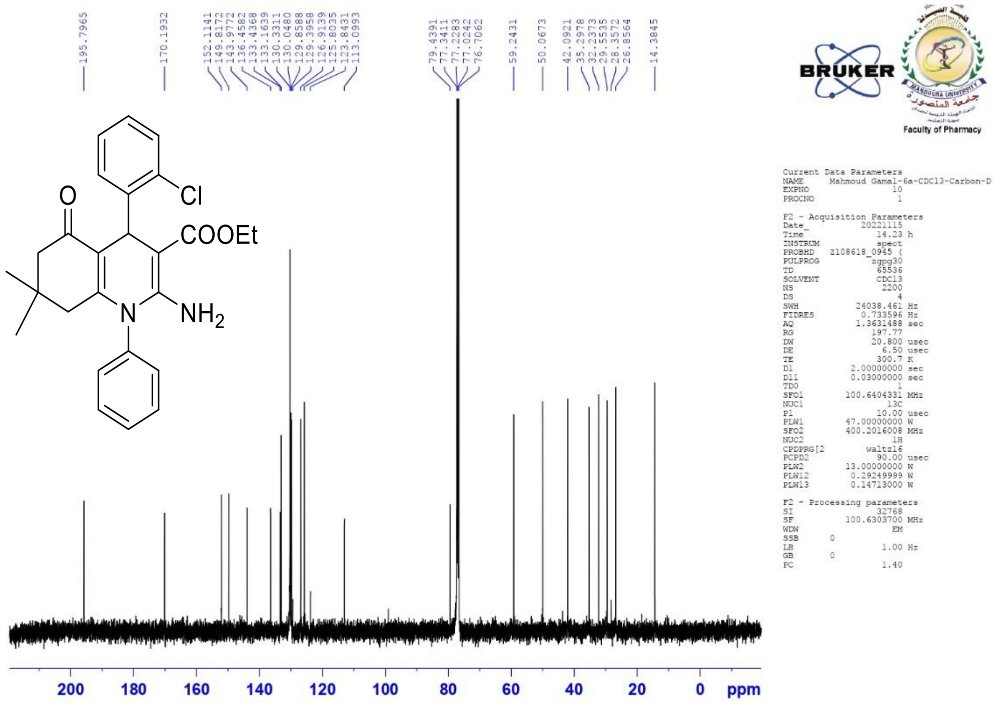

Figure S2. ^{13}C NMR (100 MHz, $CDCl_3$) spectrum of compound **6a**

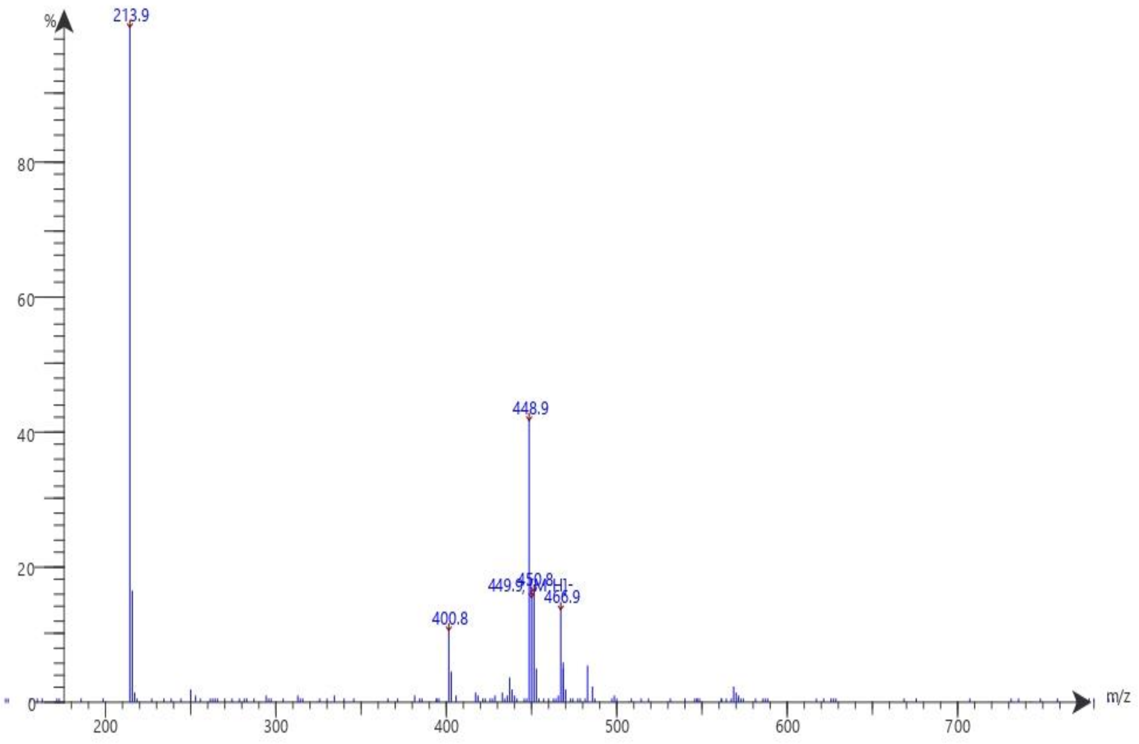

Figure S3. Mass spectrum (positive mode) of compound **6a**

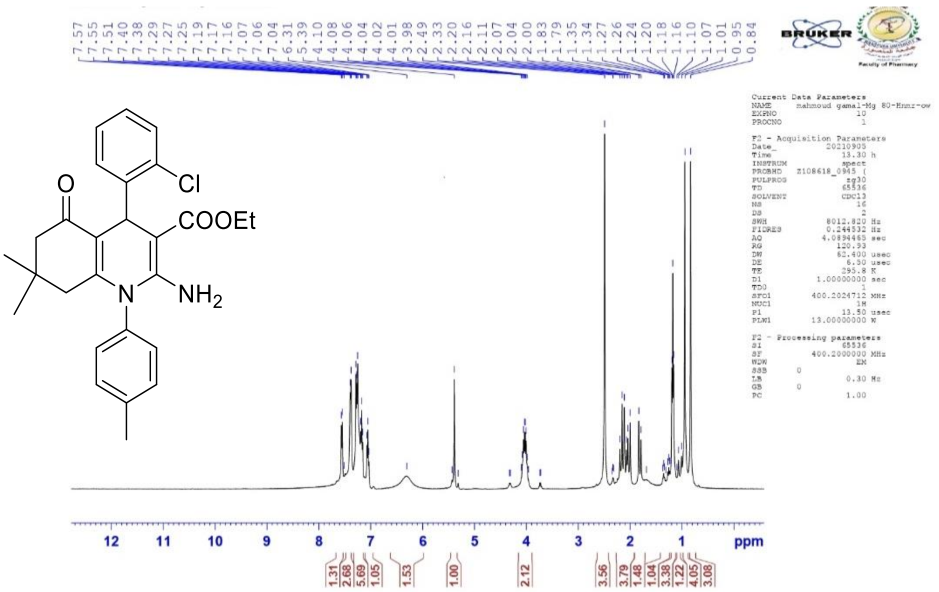

Figure S4. ^{1}H NMR (400 MHz, $CDCl_3$) spectrum of compound **6b**

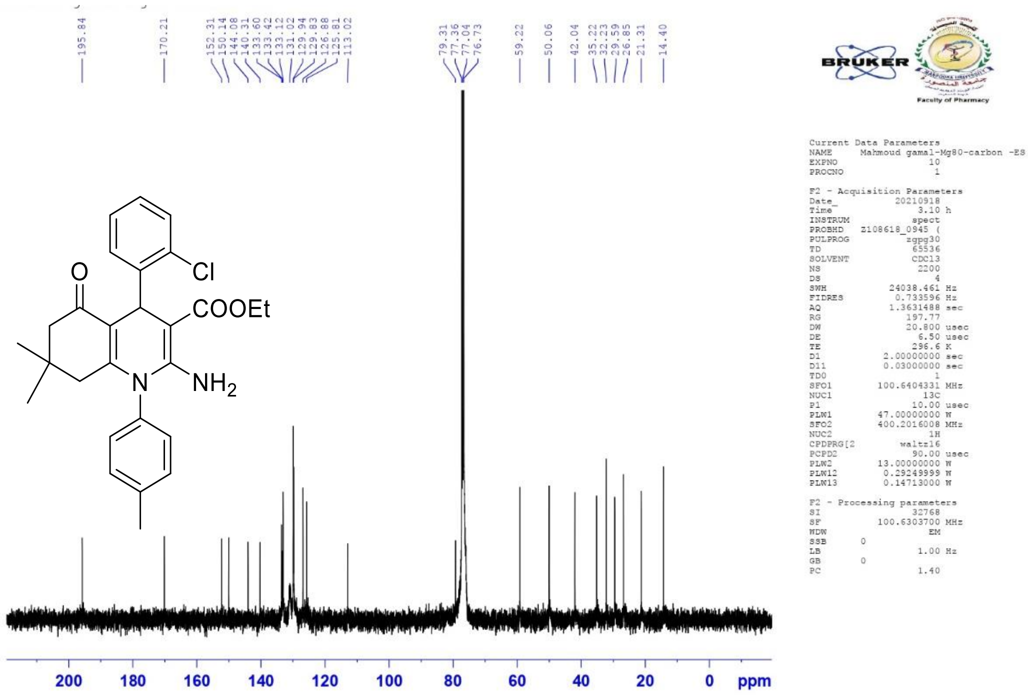

Figure S5. ^{13}C NMR (100 MHz, $CDCl_3$) spectrum of compound **6b**

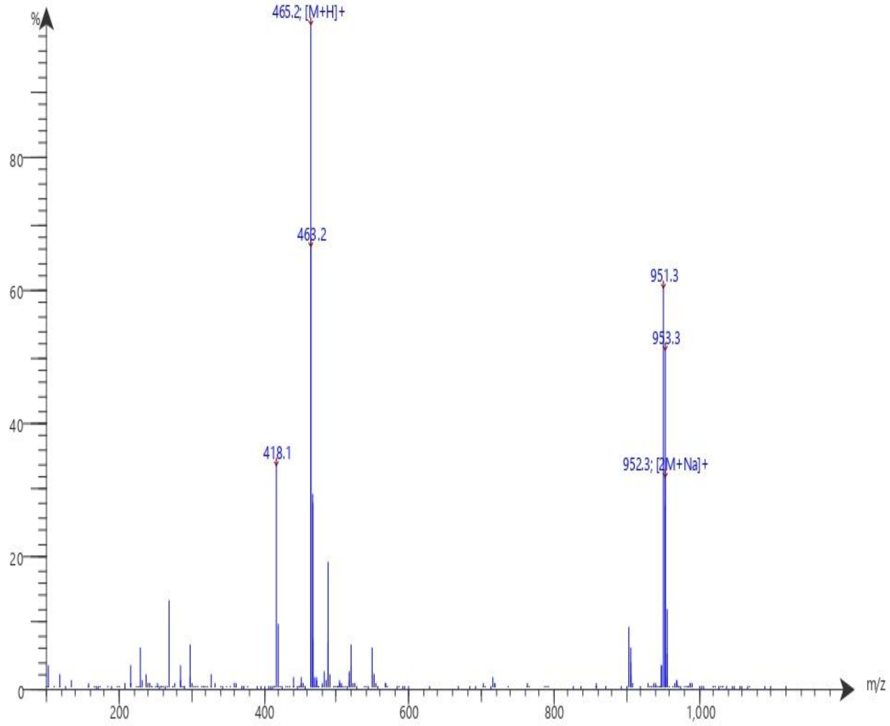

Figure S6. Mass spectrum (positive mode) of compound **6b**

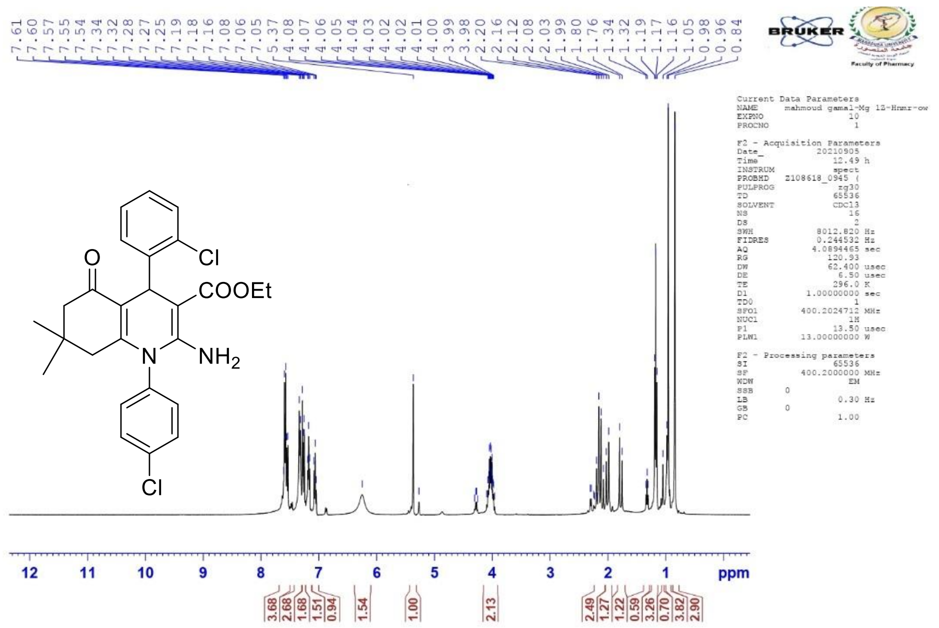

Figure S7. ^{1}H NMR (400 MHz, $CDCl_3$) spectrum of compound **6c**

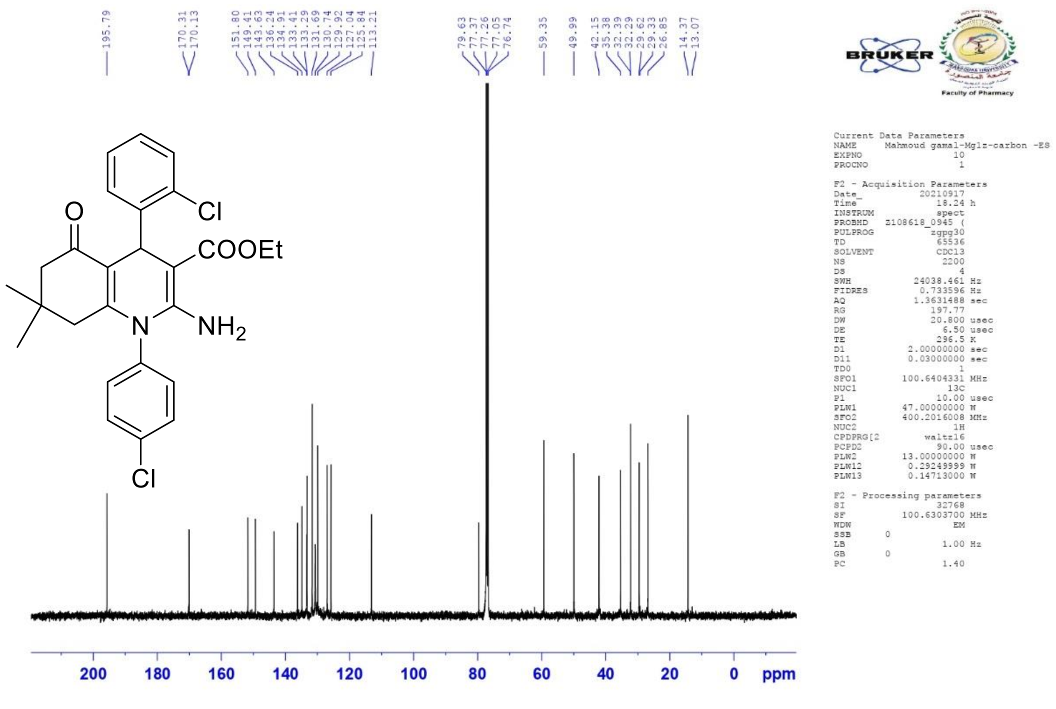

Figure S8. ^{13}C NMR (100 MHz, $CDCl_3$) spectrum of compound **6c**

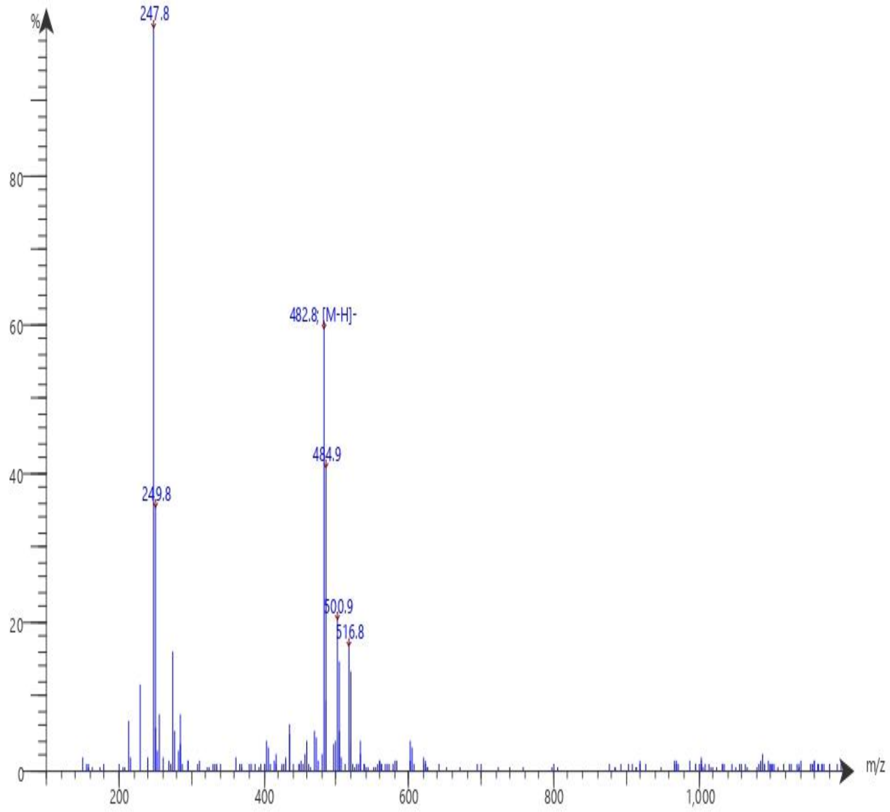

Figure S9. Mass spectrum (positive mode) of compound **6c**

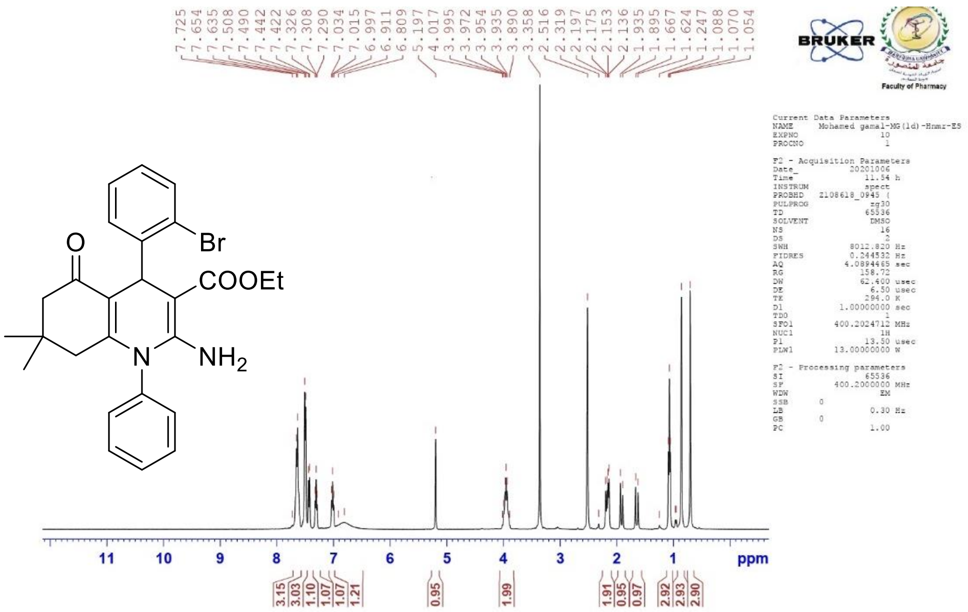

Figure S10. ^{1}H NMR (400 MHz, DMSO-d_6) spectrum of compound **6d**

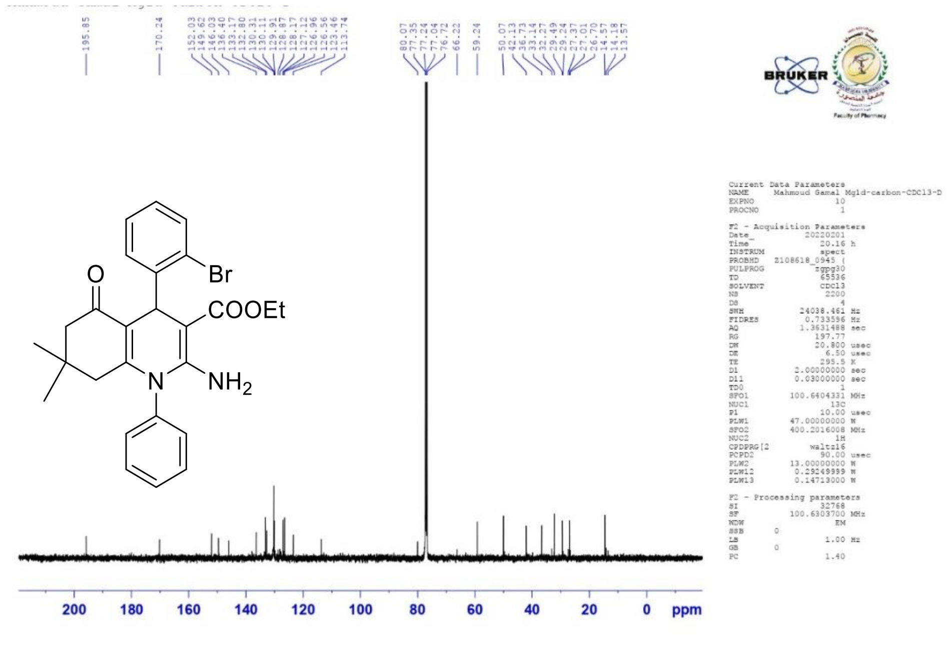

Figure S11. ^{13}C NMR (100 MHz, $CDCl_3$) spectrum of compound **6d**

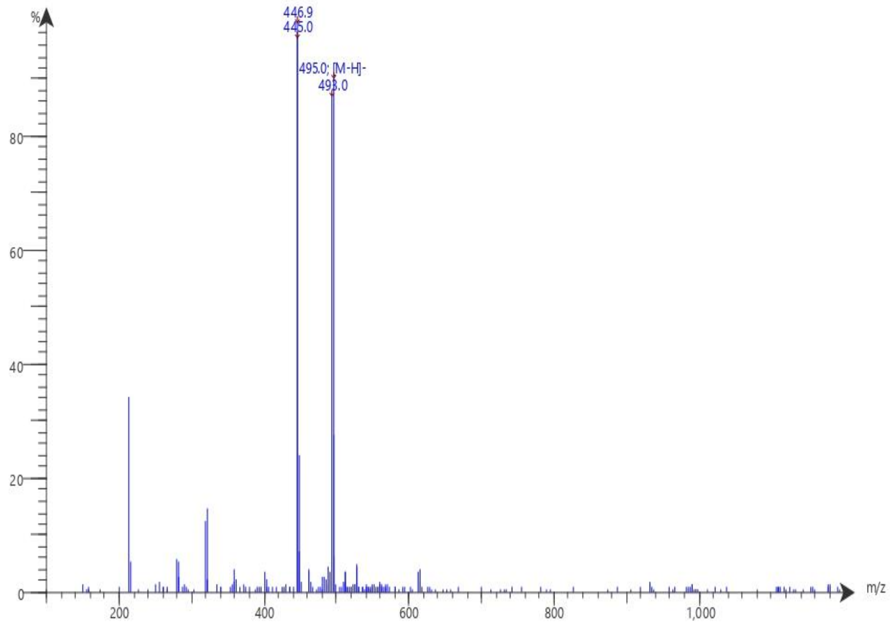

Figure S12. Mass spectrum (positive mode) of compound **6d**

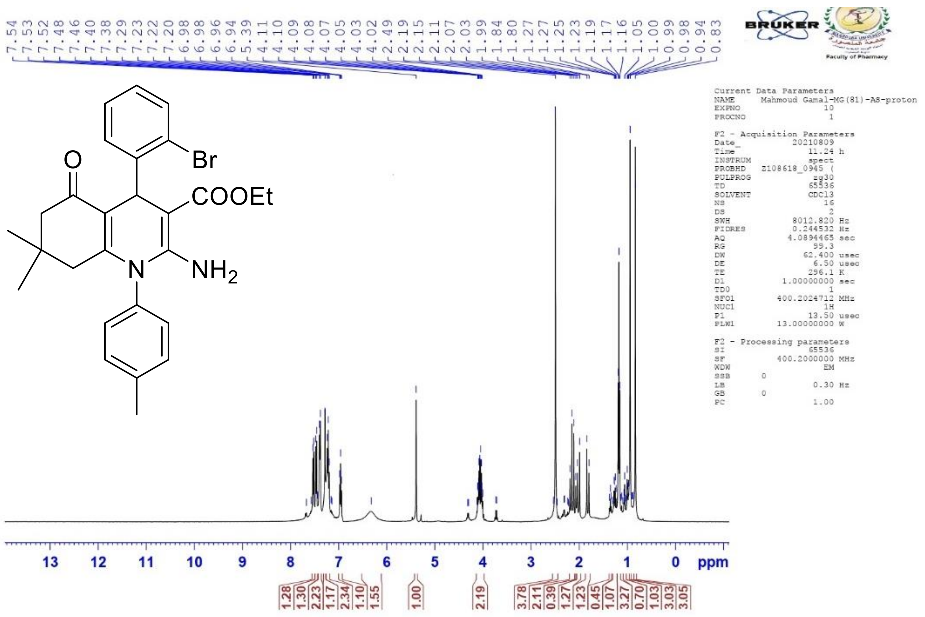

Figure S13. ^{1}H NMR (400 MHz, $CDCl_3$) spectrum of compound **6e**

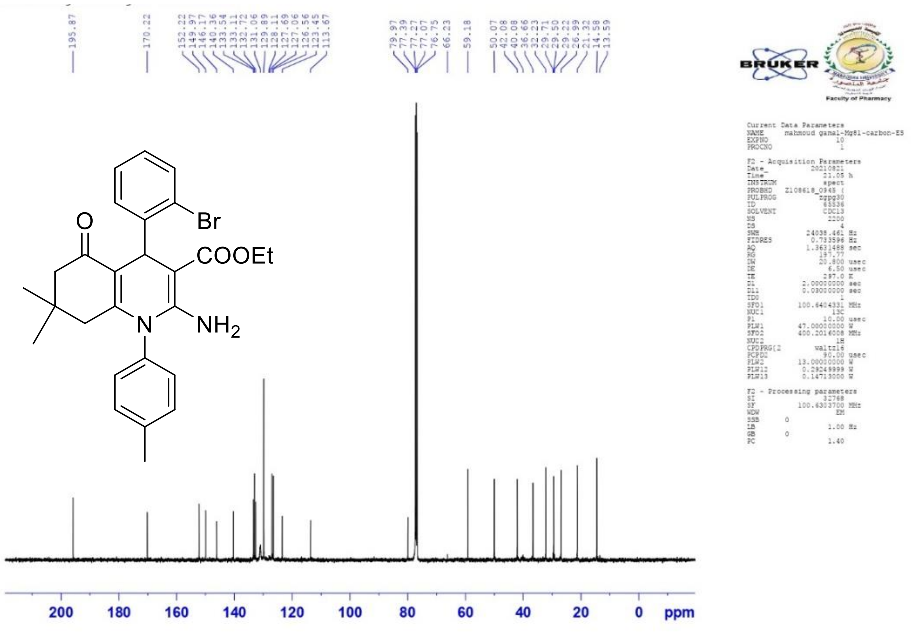

Figure S14. ^{13}C NMR (100 MHz, $CDCl_3$) spectrum of compound **6e**

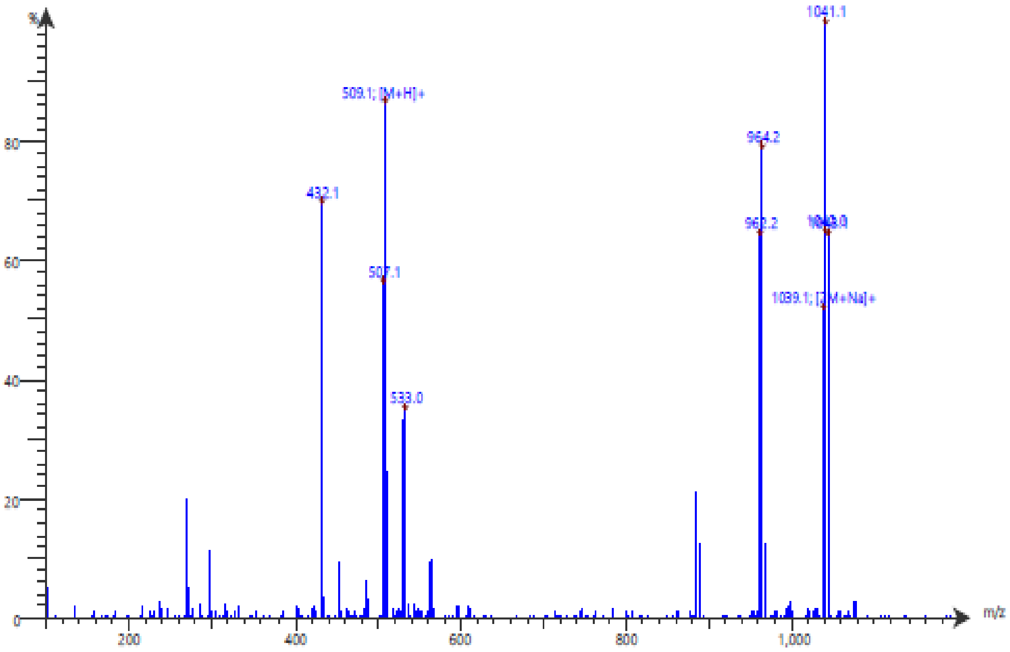

Figure S15. Mass spectrum (positive mode) of compound **6e**

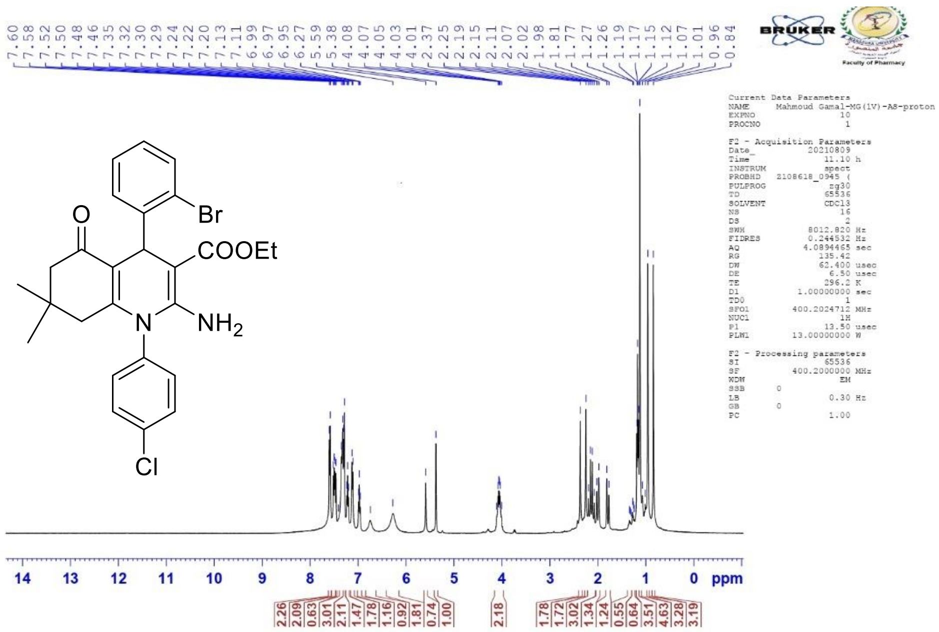

Figure S16. ^{1}H NMR (400 MHz, $CDCl_3$) spectrum of compound **6f**

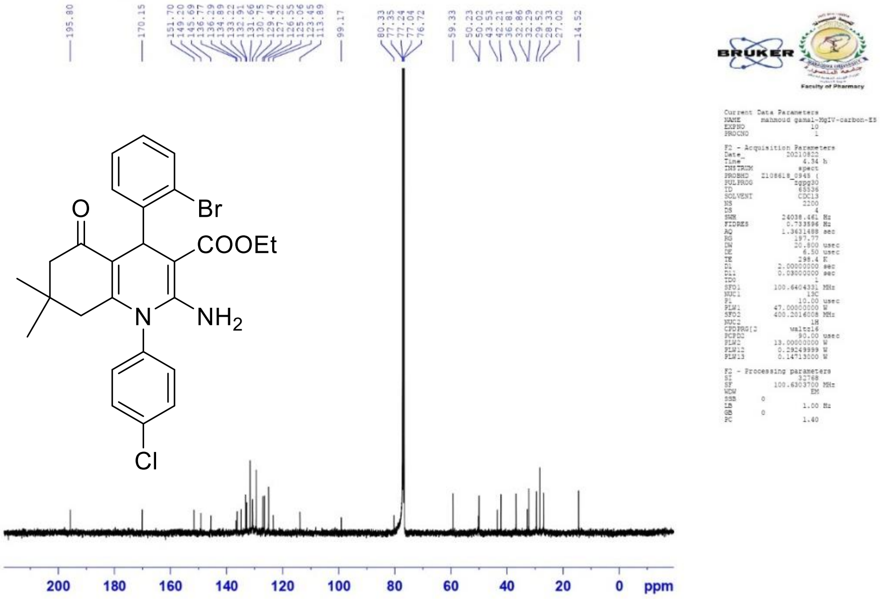

Figure S17. ^{13}C NMR (100 MHz, $CDCl_3$) spectrum of compound **6f**

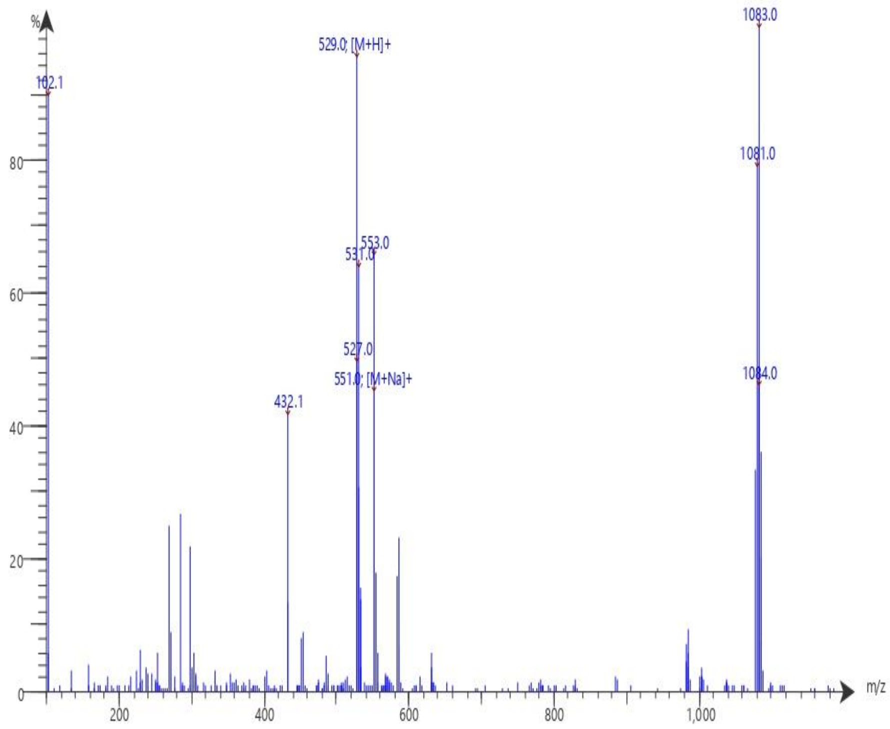

Figure S18. Mass spectrum (positive mode) of compound **6f**

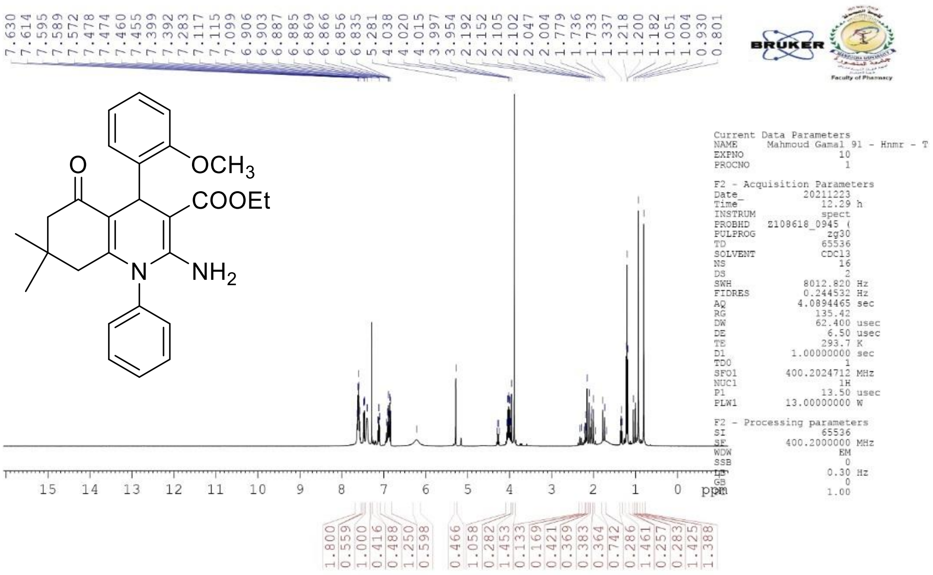

Figure S19. ^{1}H NMR (400 MHz, $CDCl_3$) spectrum of compound **6g**

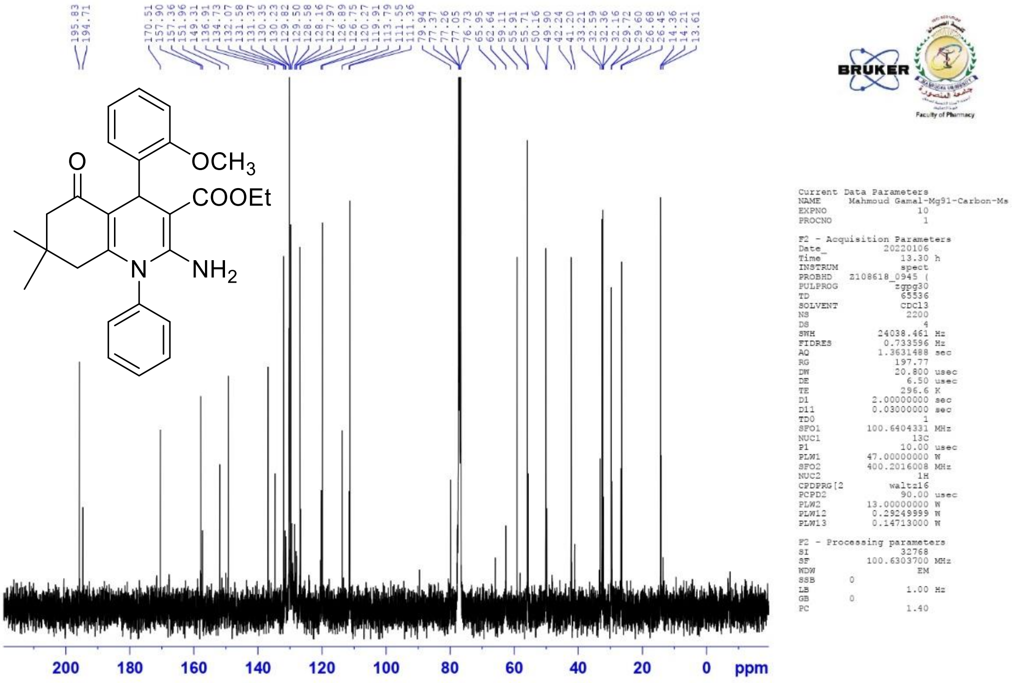

Figure S20. ^{13}C NMR (100 MHz, $CDCl_3$) spectrum of compound **6g**

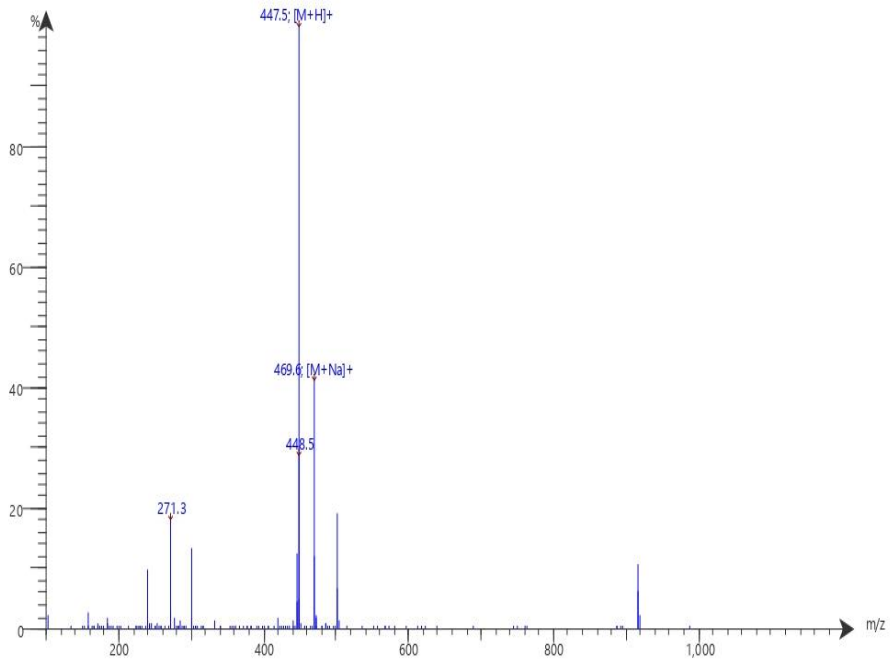

Figure S21. Mass spectrum (positive mode) of compound **6g**

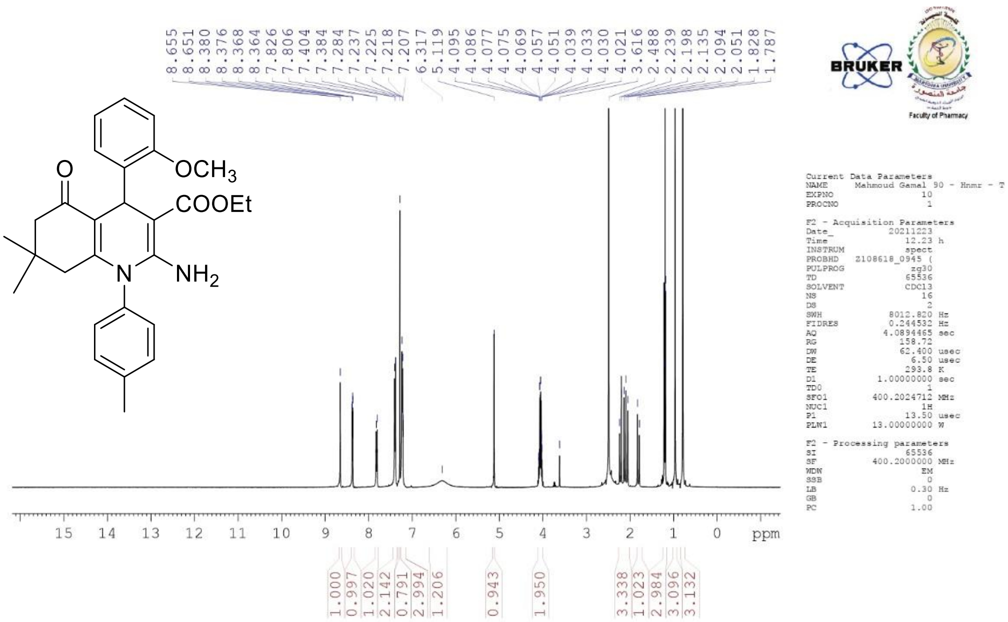

Figure S22. ^{1}H NMR (400 MHz, $CDCl_3$) spectrum of compound **6h**

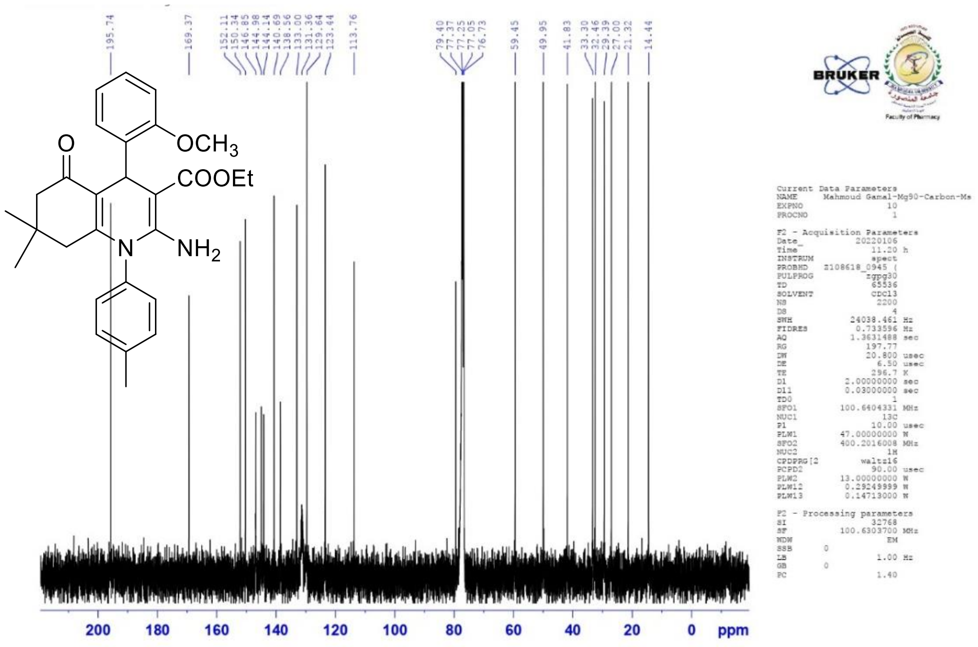

Figure S23. ^{13}C NMR (100 MHz, $CDCl_3$) spectrum of compound **6h**

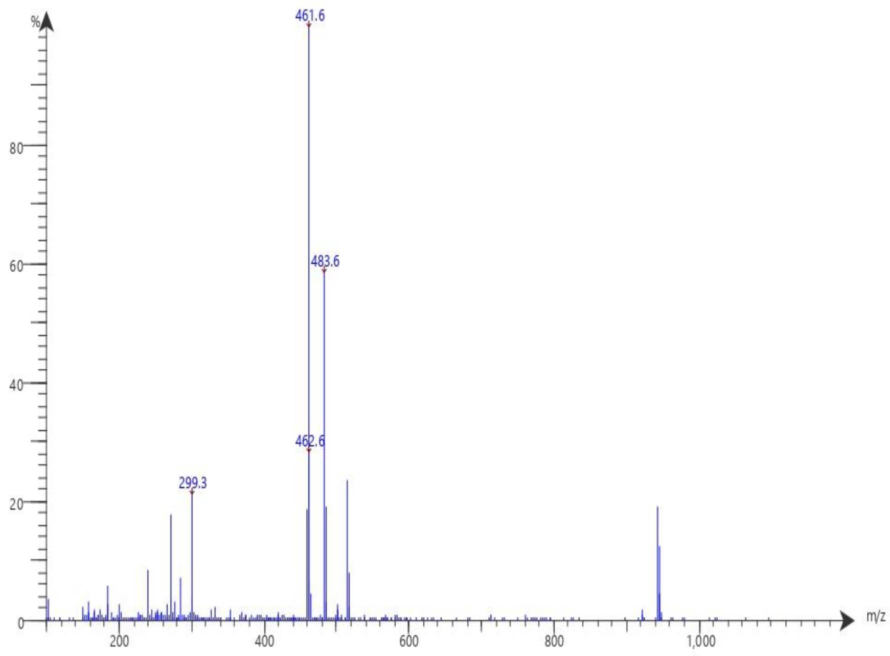

Figure S24. Mass spectrum (positive mode) of compound **6h**

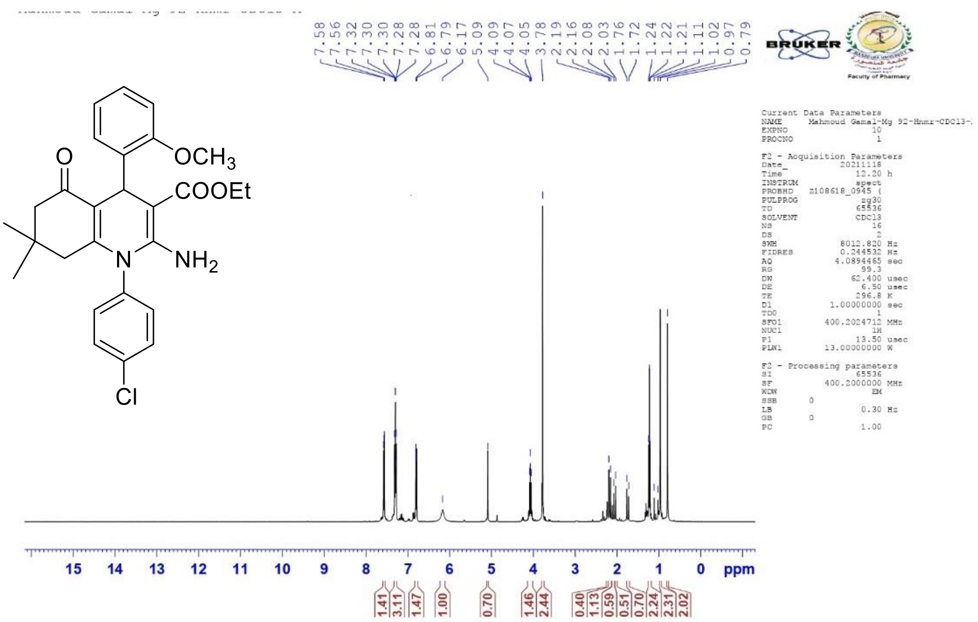

Figure S25. ^{1}H NMR (400 MHz, $CDCl_3$) spectrum of compound **6i**

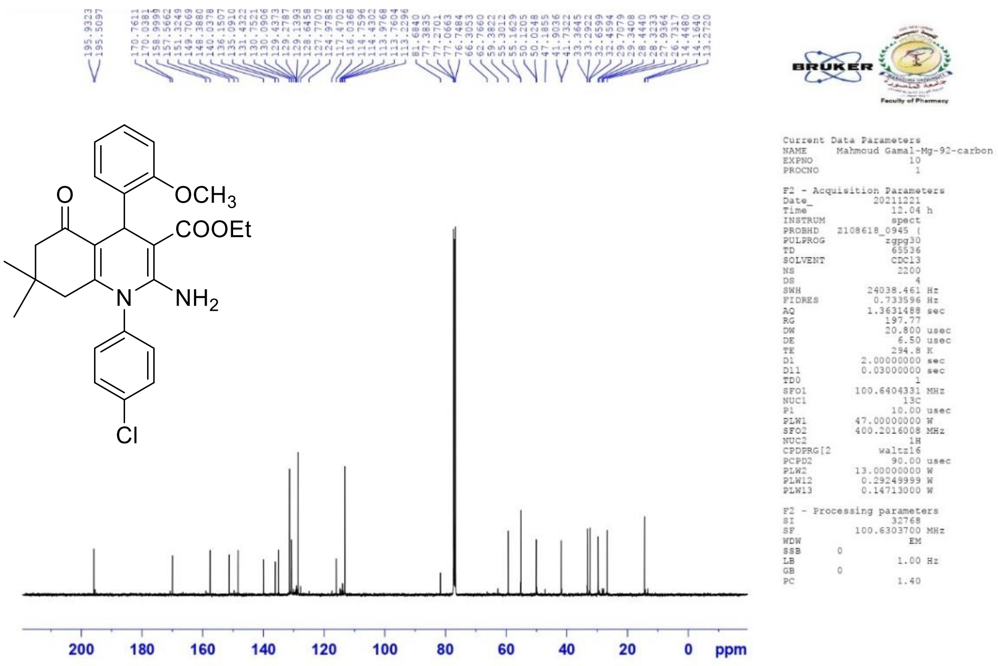

Figure S26. ^{13}C NMR (100 MHz, $CDCl_3$) spectrum of compound **6i**

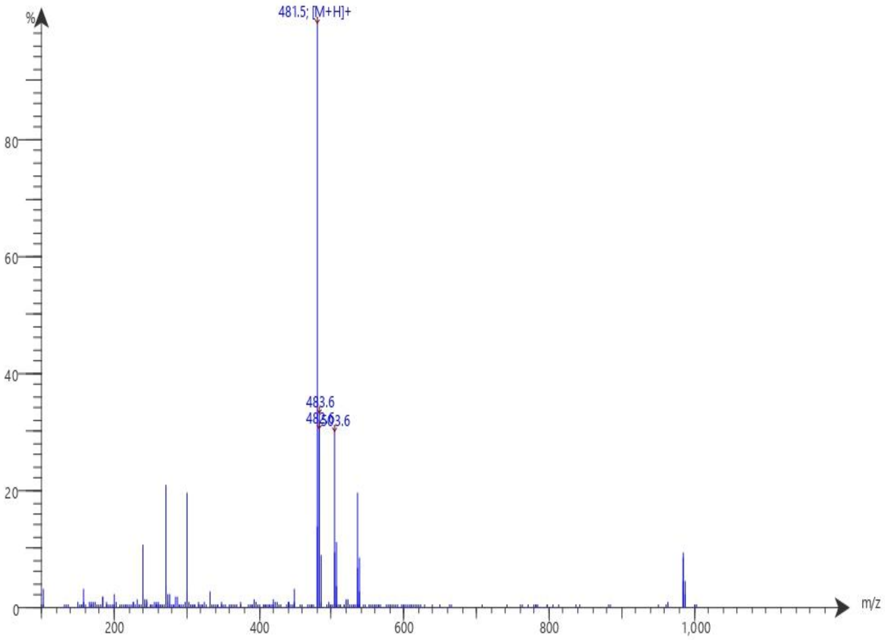

Figure S27. Mass spectrum (positive mode) of compound **6i**

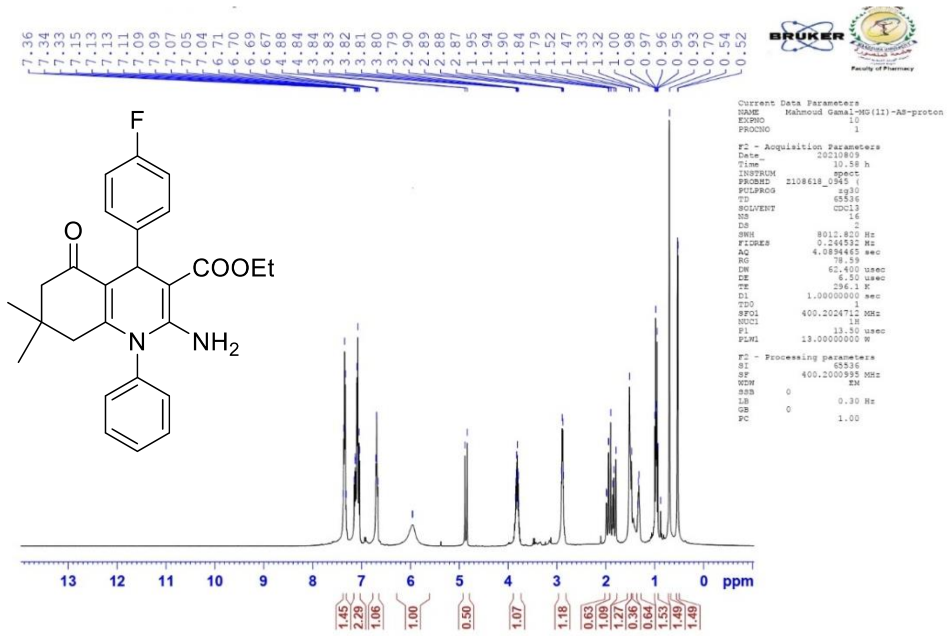

Figure S28. ^{1}H NMR (400 MHz, $CDCl_3$) spectrum of compound **8a**

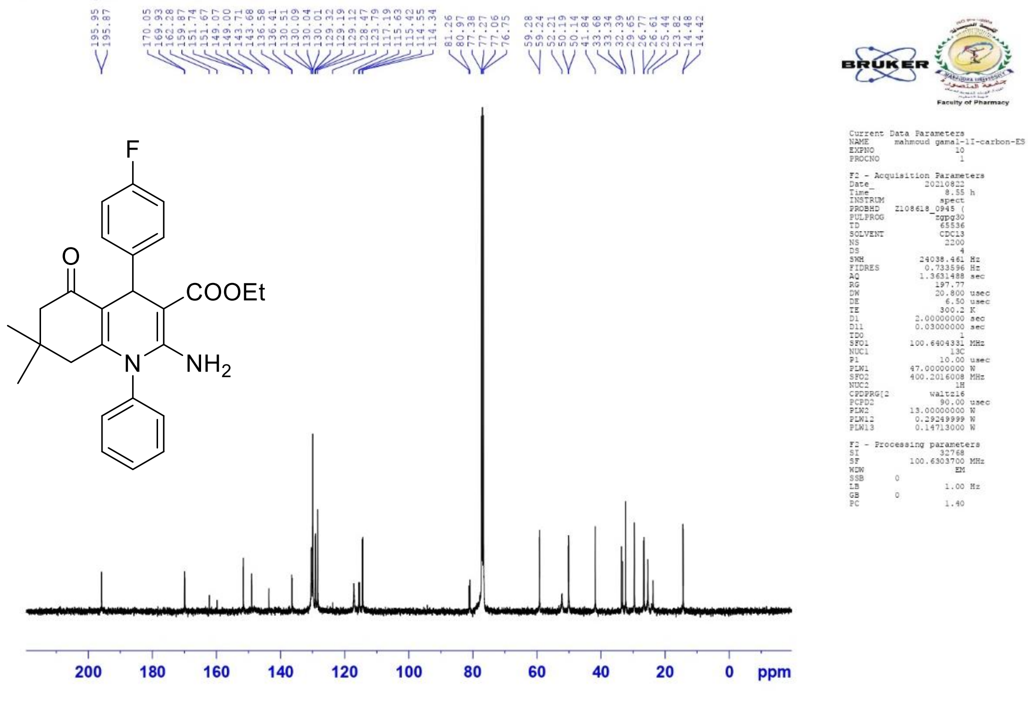

Figure S29. ^{13}C NMR (100 MHz, $CDCl_3$) spectrum of compound **8a**

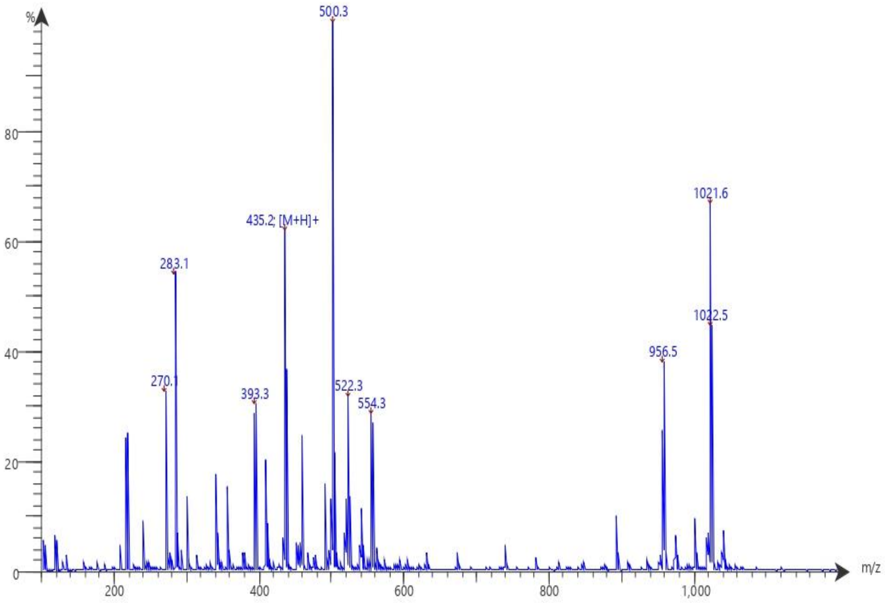

Figure S30. Mass spectrum (positive mode) of compound **8a**

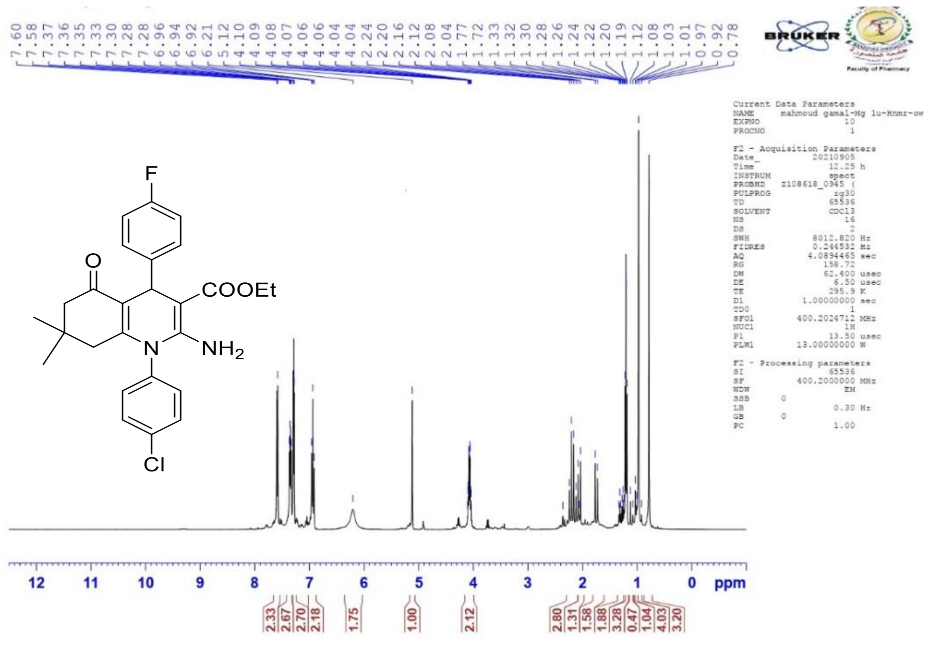

Figure S31. ^{1}H NMR (400 MHz, $CDCl_3$) spectrum of compound **8b**

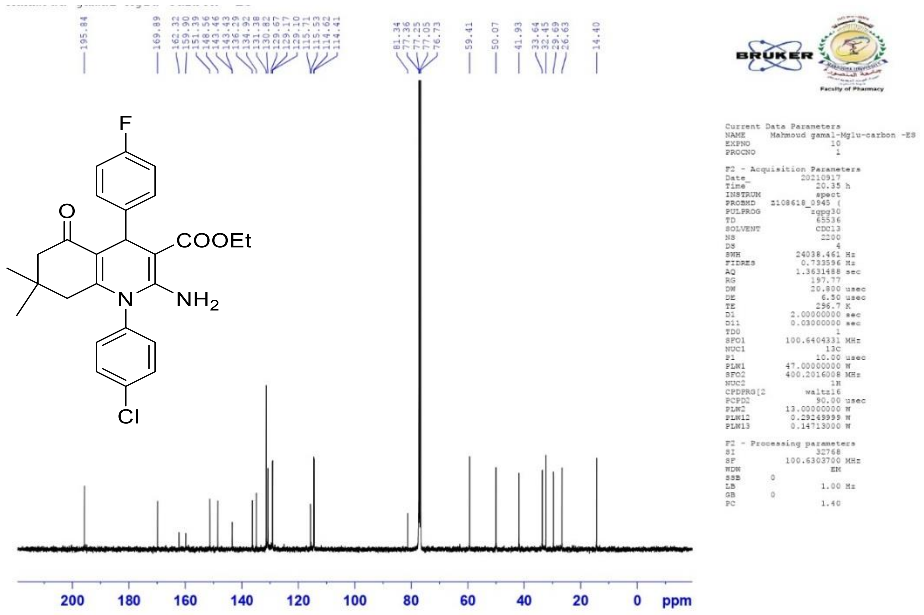

Figure S32. ^{13}C NMR (100 MHz, $CDCl_3$) spectrum of compound **8b**

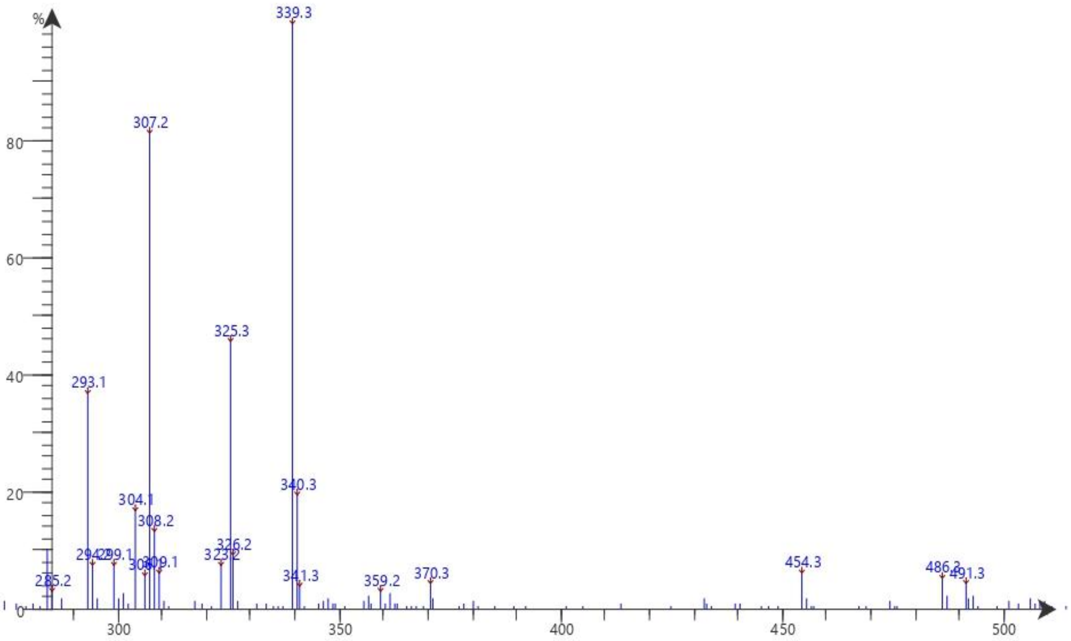

Figure S33. Mass spectrum (positive mode) of compound **8b**

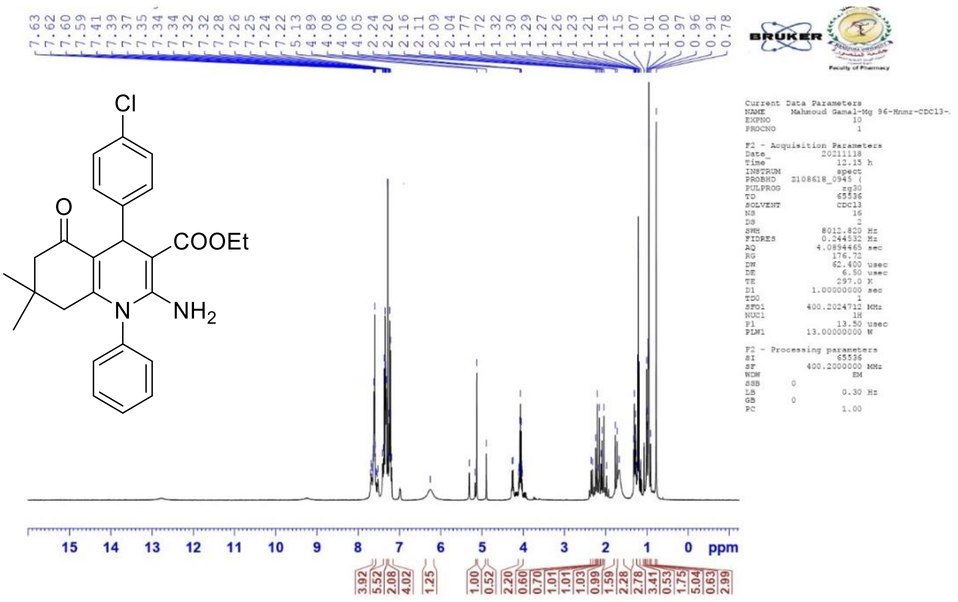

Figure S34. ^{1}H NMR (400 MHz, $CDCl_3$) spectrum of compound **8c**

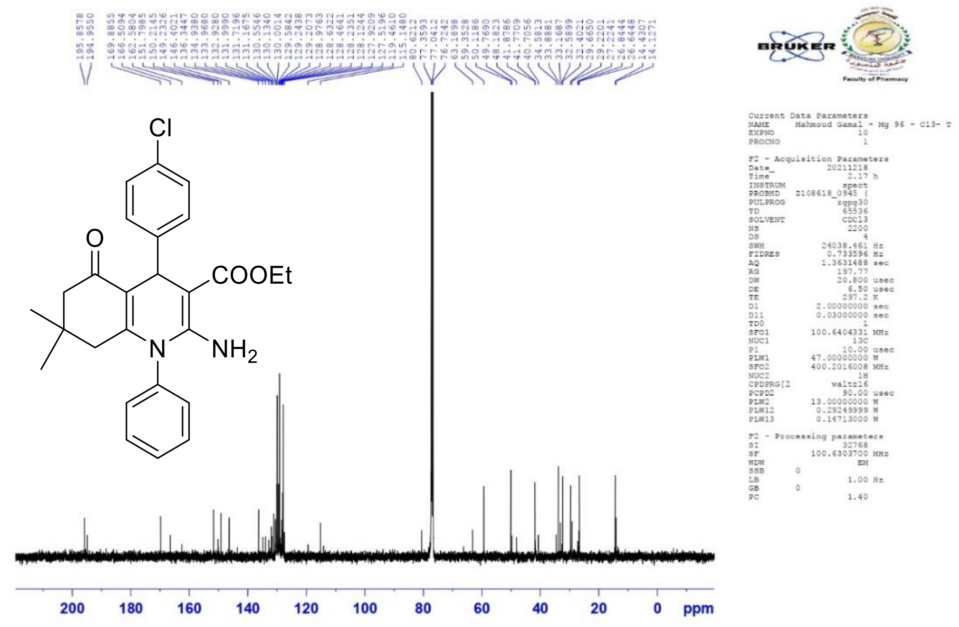

Figure S35. ^{13}C NMR (100 MHz, $CDCl_3$) spectrum of compound **8c**

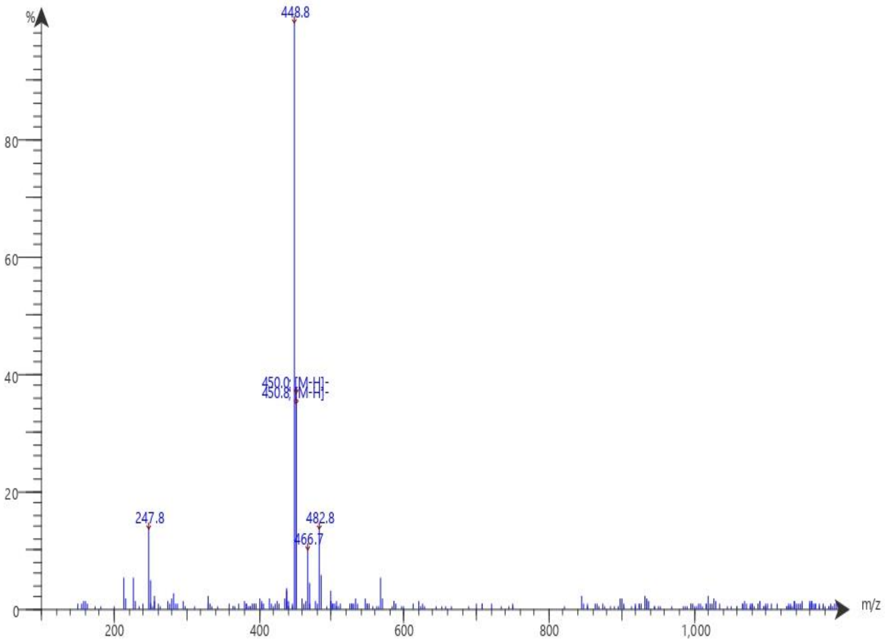

Figure S36. Mass spectrum (positive mode) of compound **8c**

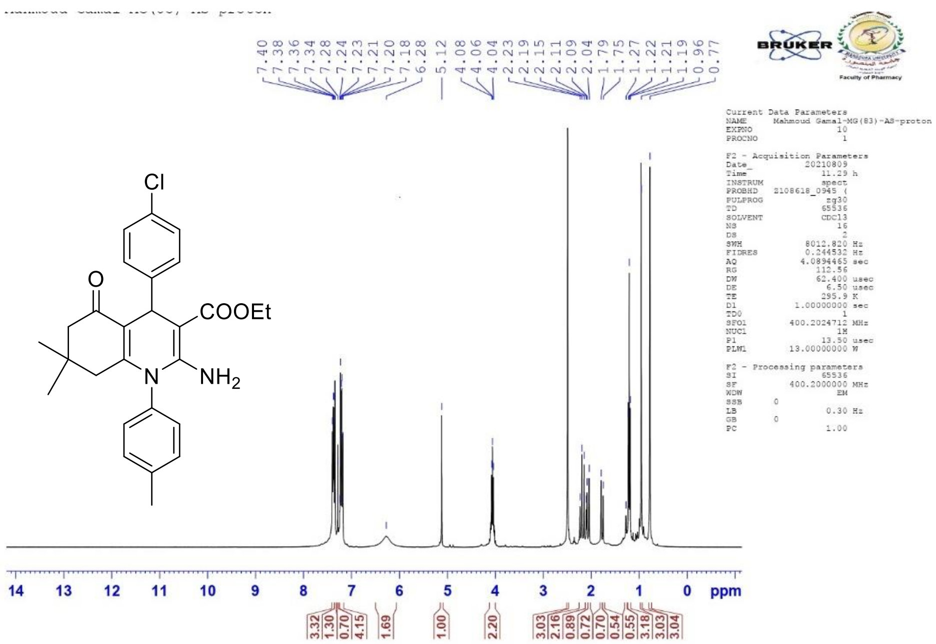

Figure S37. ^{1}H NMR (400 MHz, $CDCl_3$) spectrum of compound **8d**

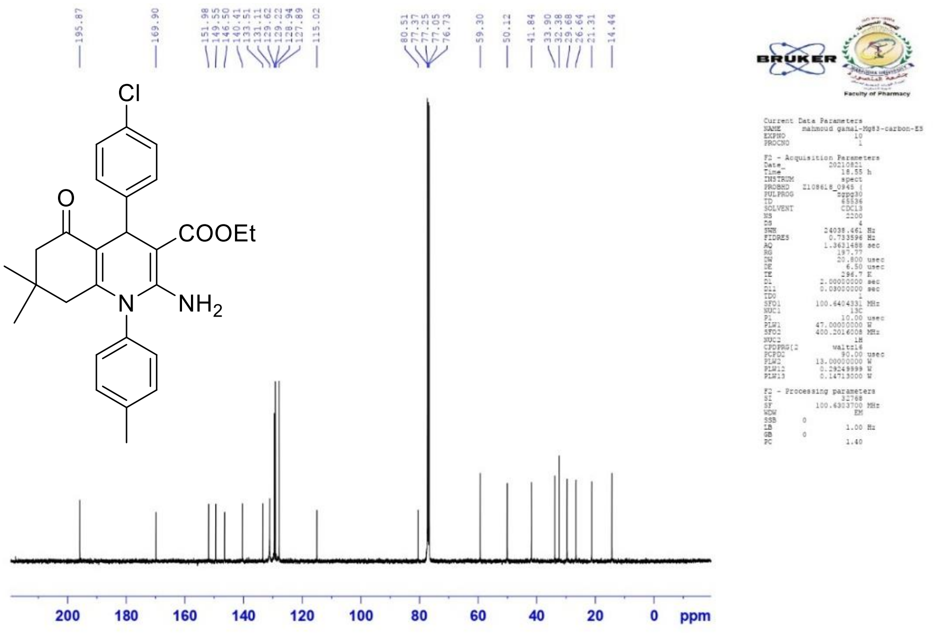

Figure S38. ^{13}C NMR (100 MHz, $CDCl_3$) spectrum of compound **8d**

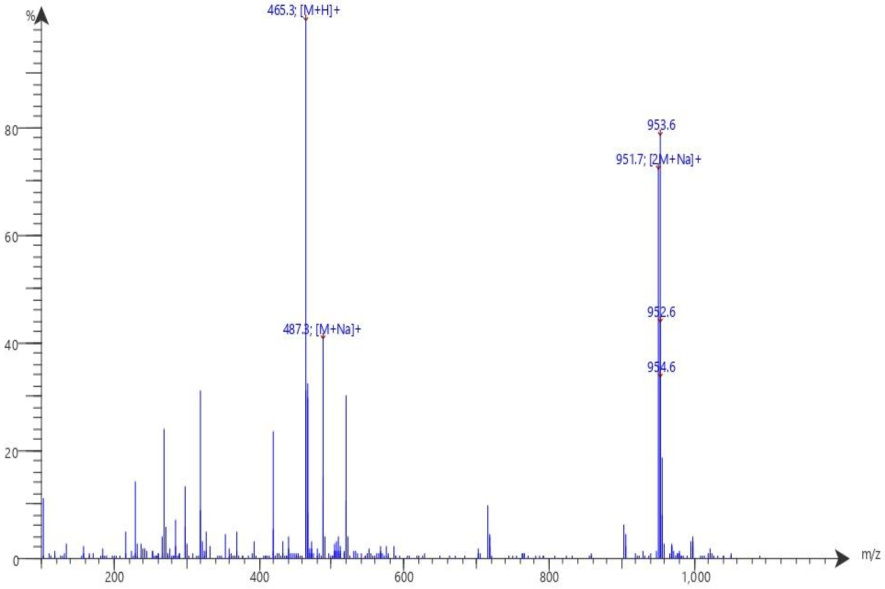

Figure S39. Mass spectrum (positive mode) of compound **8d**

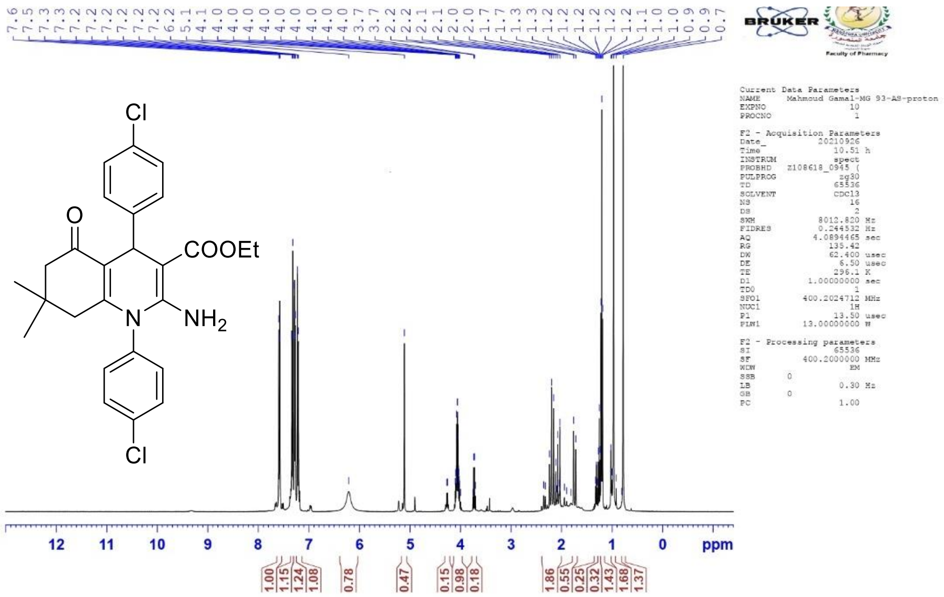

Figure S40. ^{1}H NMR (400 MHz, $CDCl_3$) spectrum of compound **8e**

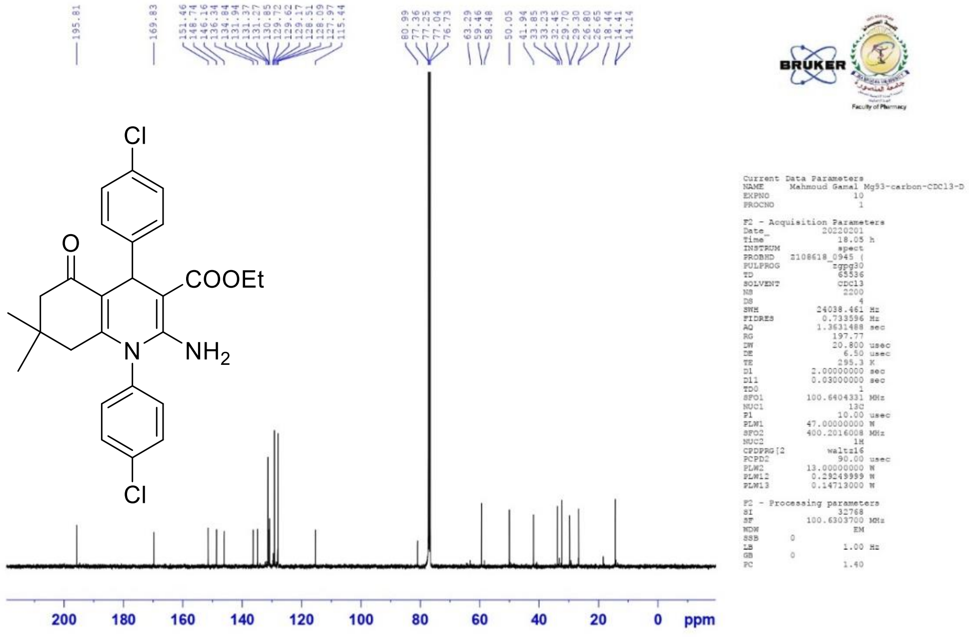

Figure S41. ^{13}C NMR (100 MHz, $CDCl_3$) spectrum of compound **8e**

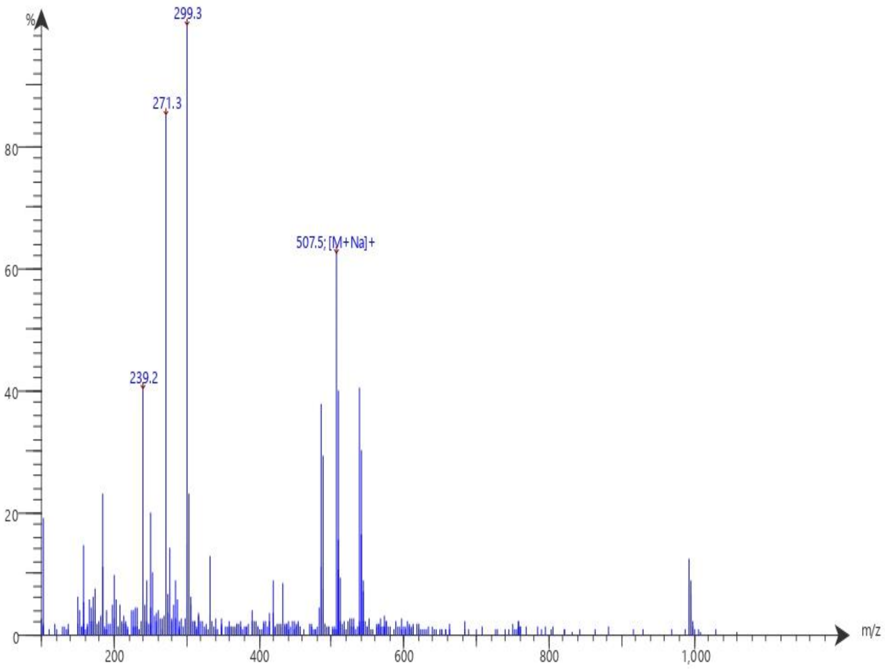

Figure S42. Mass spectrum (positive mode) of compound **8e**

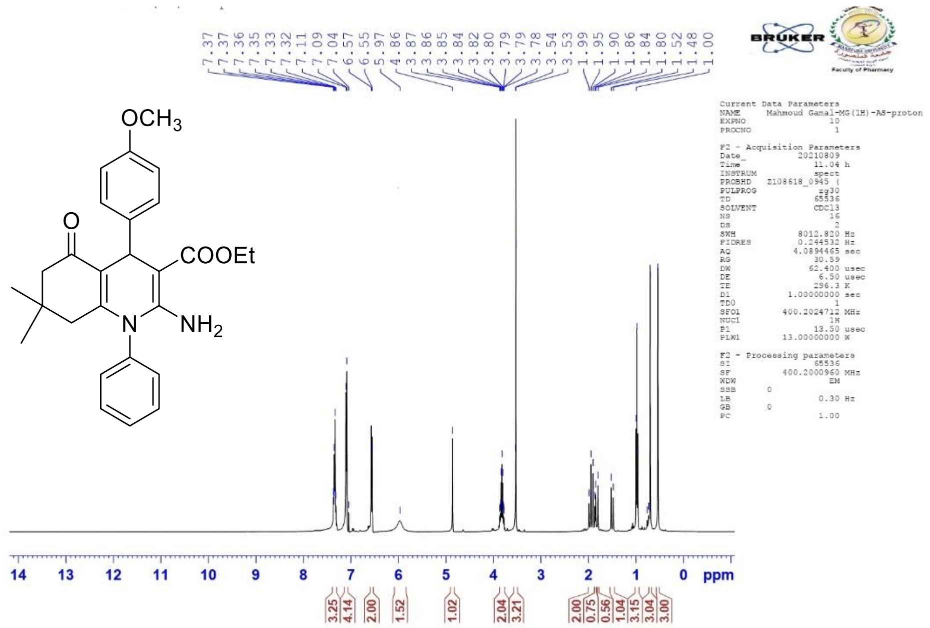

Figure S43. ^{1}H NMR (400 MHz, $CDCl_3$) spectrum of compound **8f**

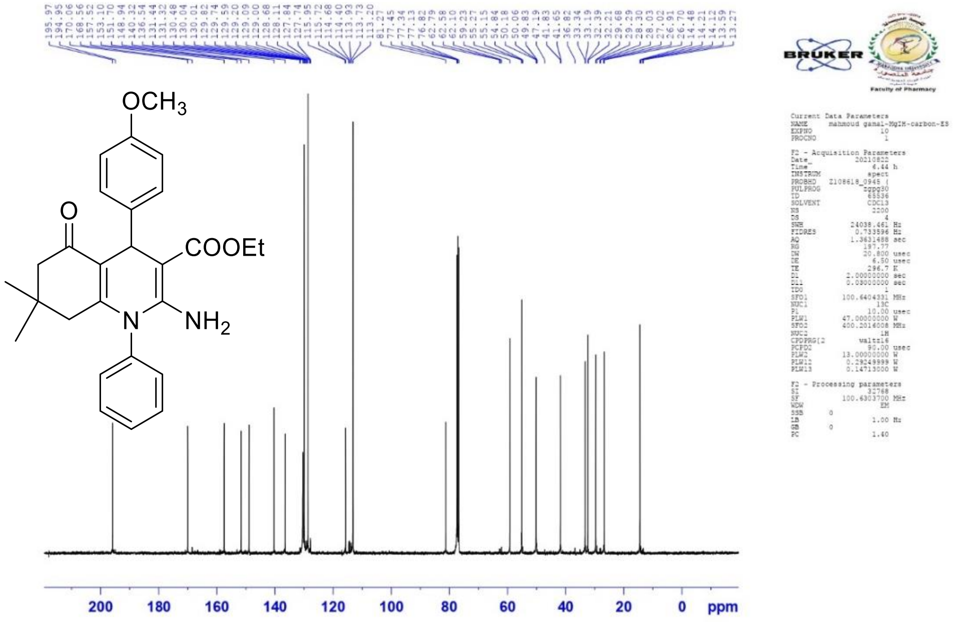

Figure S44. ^{13}C NMR (100 MHz, $CDCl_3$) spectrum of compound **8f**

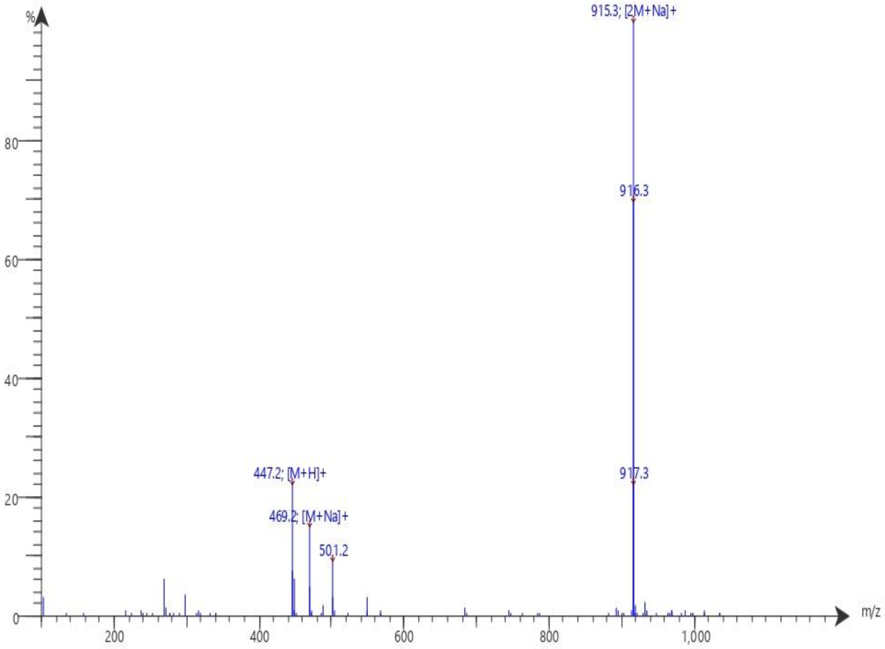

Figure S45. Mass spectrum (positive mode) of compound **8f**

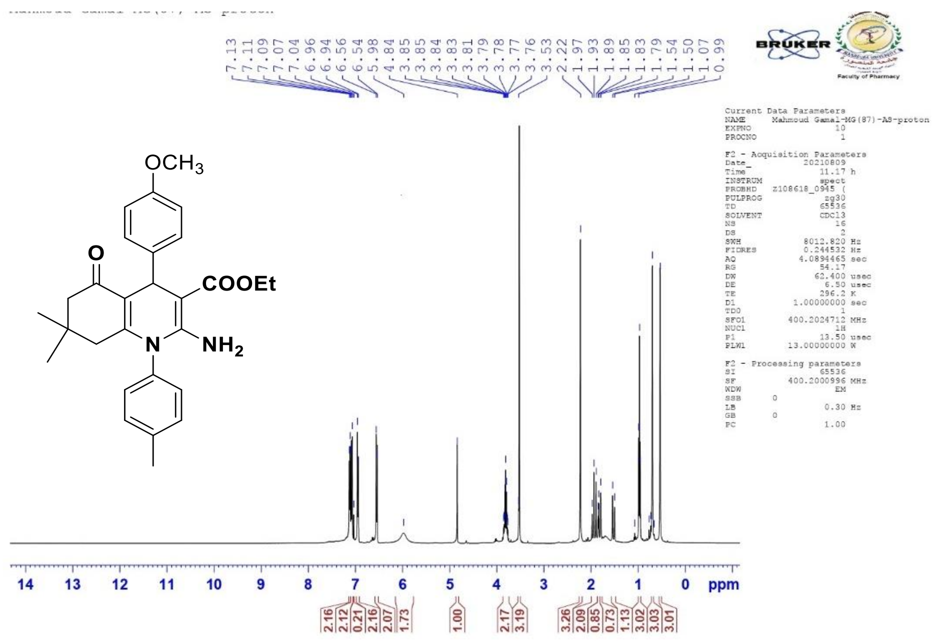

Figure S46. ^{1}H NMR (400 MHz, $CDCl_3$) spectrum of compound **8g**

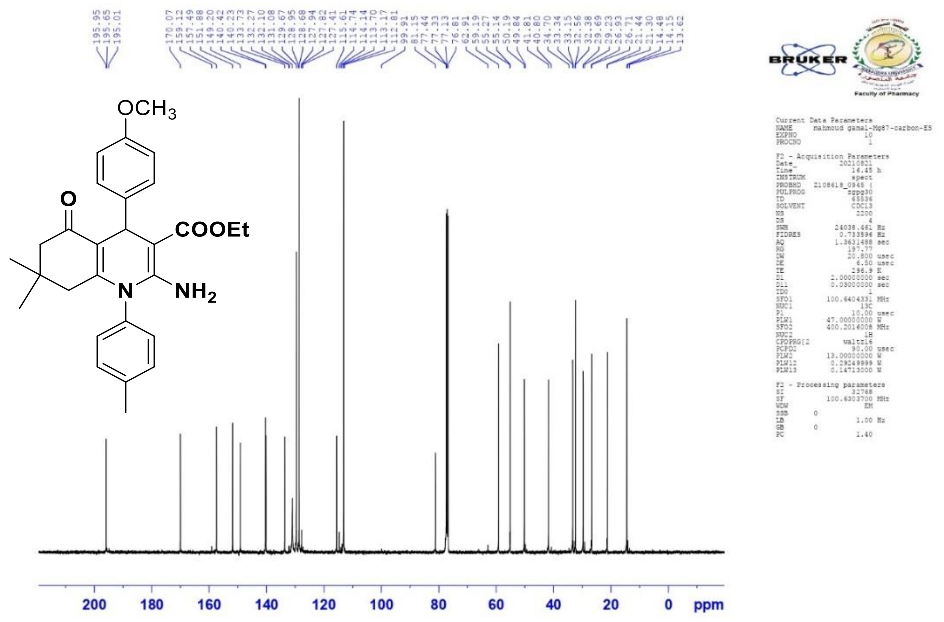

Figure S47. ^{13}C NMR (100 MHz, $CDCl_3$) spectrum of compound **8g**

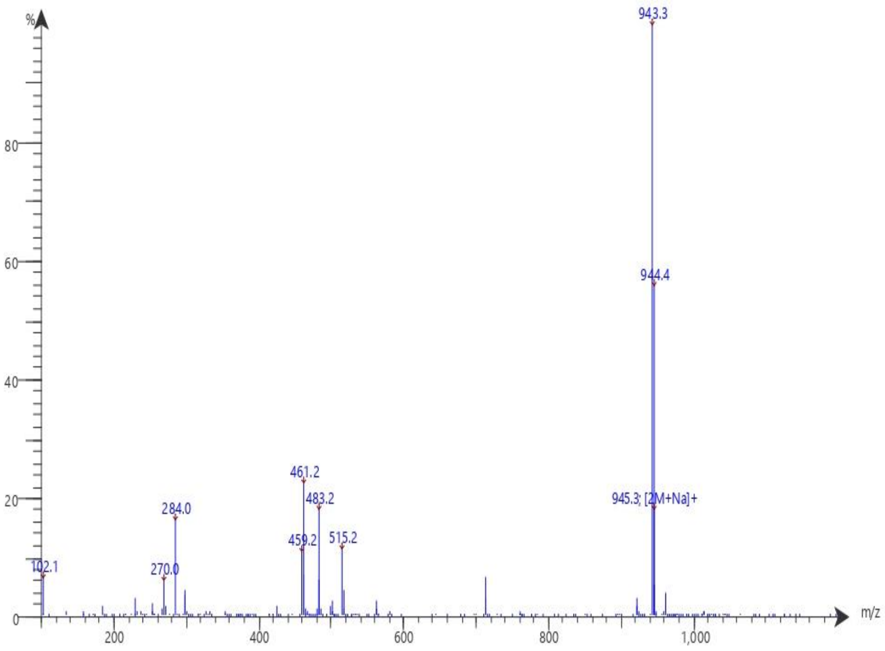

Figure S48. Mass spectrum (positive mode) of compound **8g**

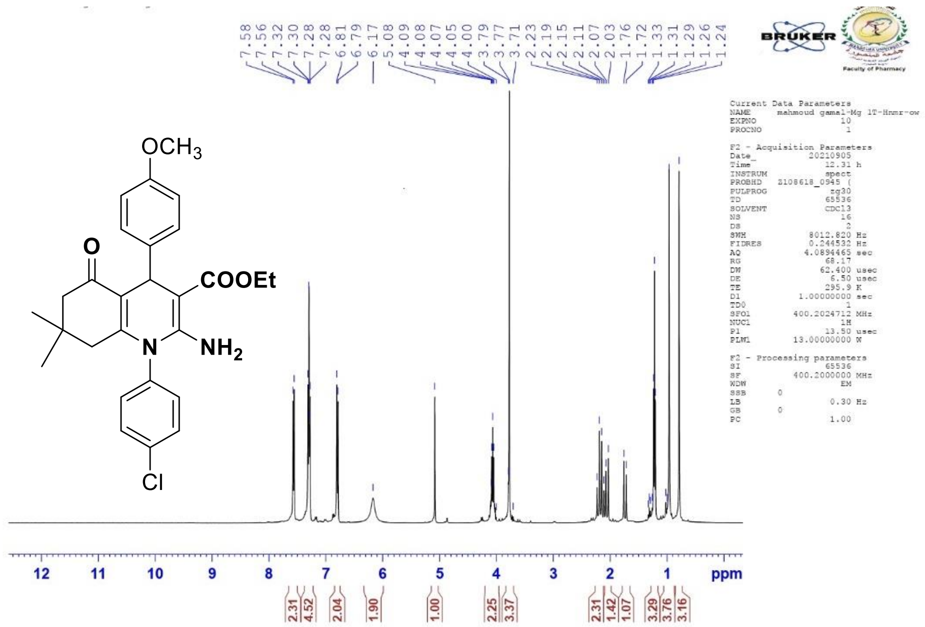

Figure S49. ^{1}H NMR (400 MHz, $CDCl_3$) spectrum of compound **8h**

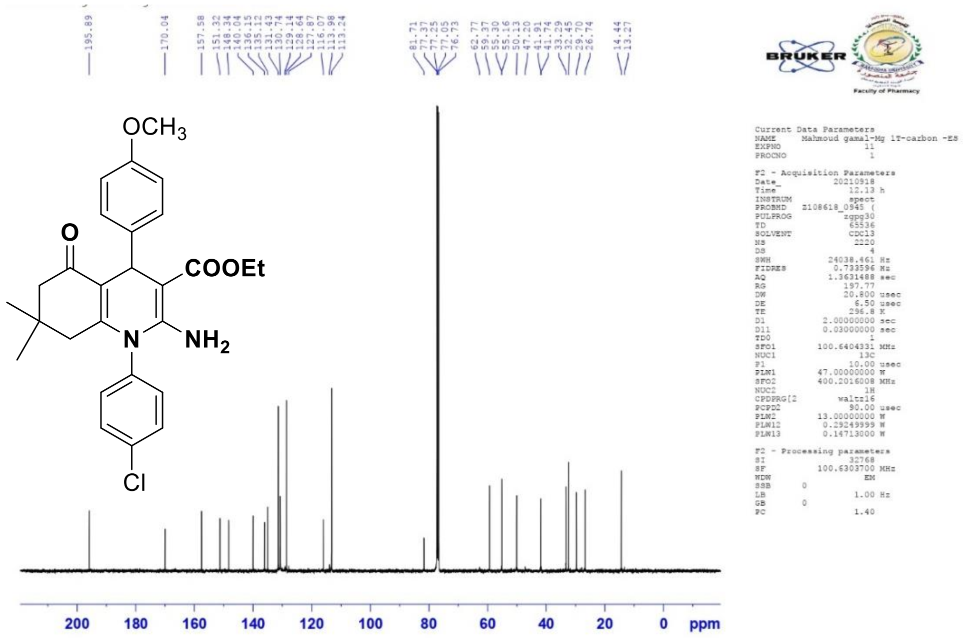

Figure S50. ^{13}C NMR (100 MHz, $CDCl_3$) spectrum of compound **8h**

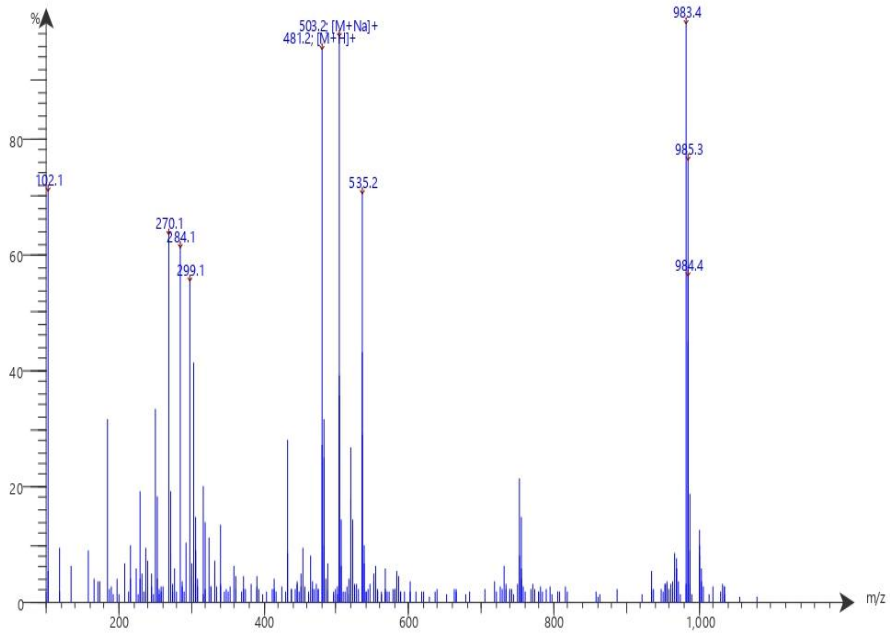

Figure S51. Mass spectrum (positive mode) of compound **8h**

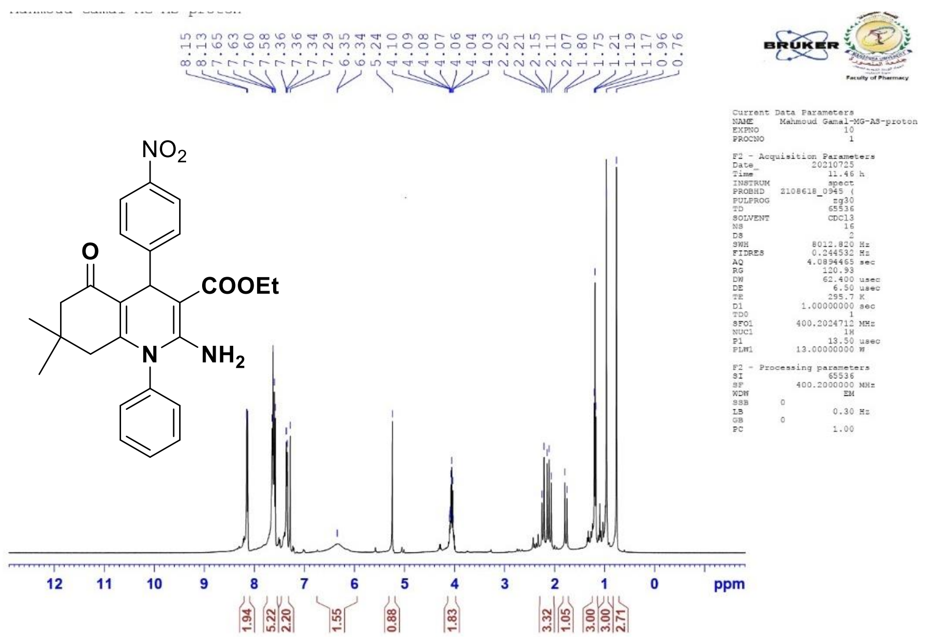

Figure S52. ^{1}H NMR (400 MHz, $CDCl_3$) spectrum of compound **8i**

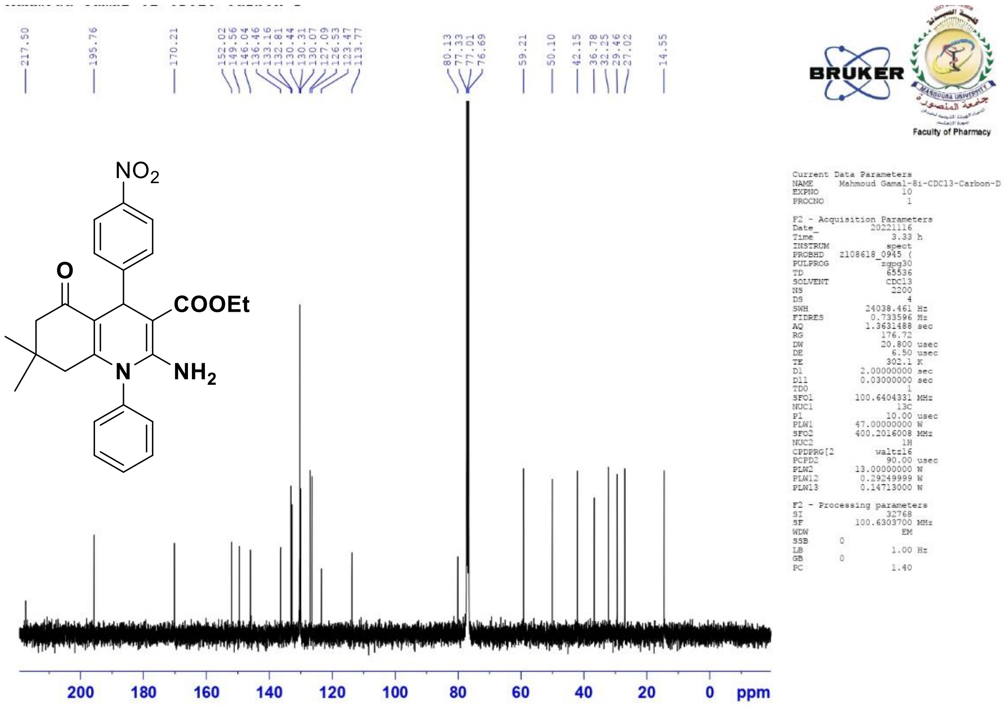

Figure S53. ^{13}C NMR (100 MHz, $CDCl_3$) spectrum of compound **8i**

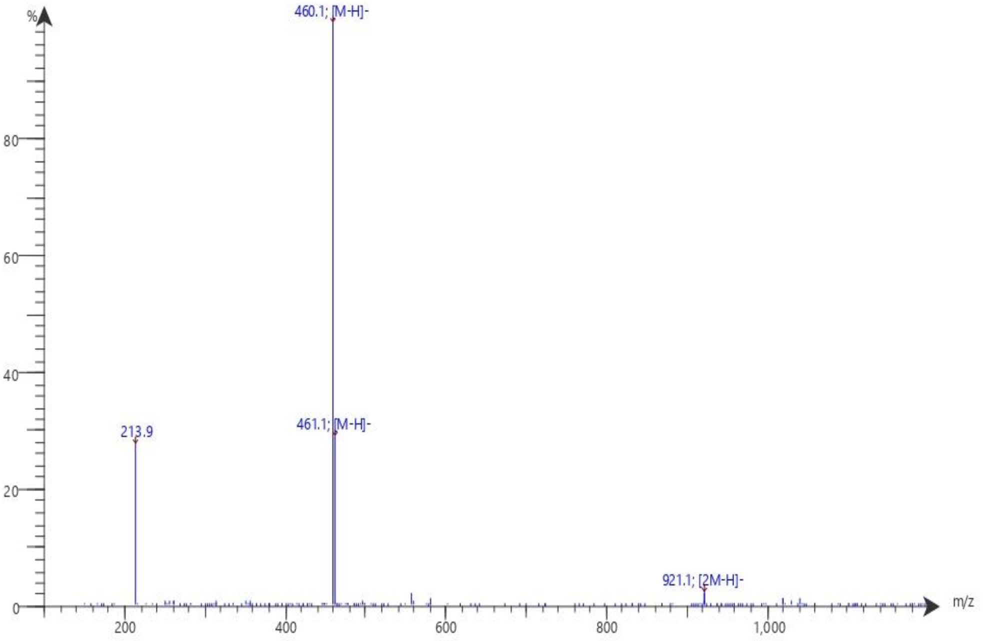

Figure S54. Mass spectrum (positive mode) of compound **8i**

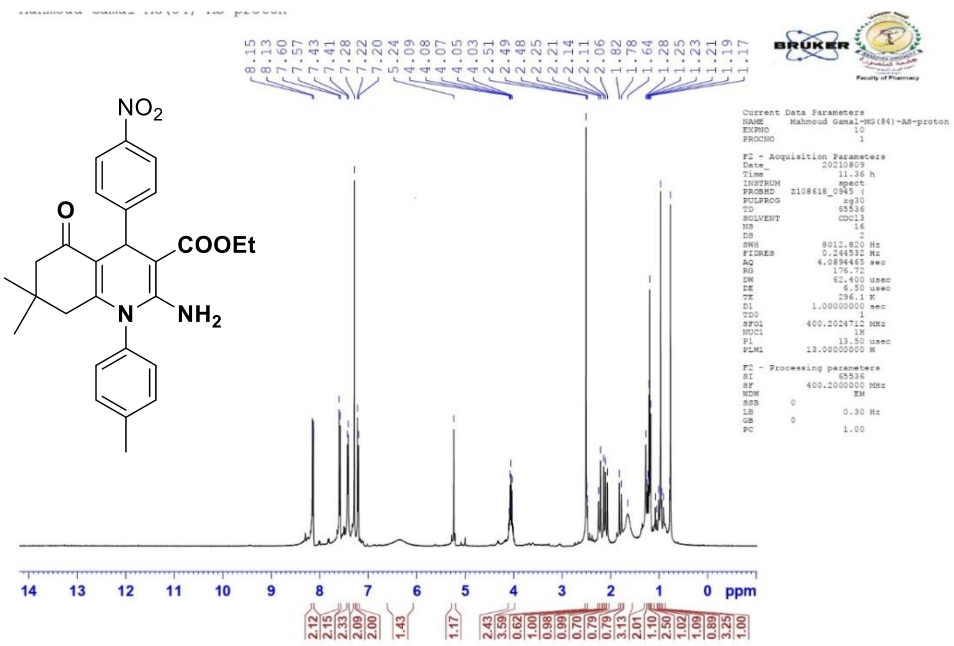

Figure S55. ^{1}H NMR (400 MHz, $CDCl_3$) spectrum of compound **8j**

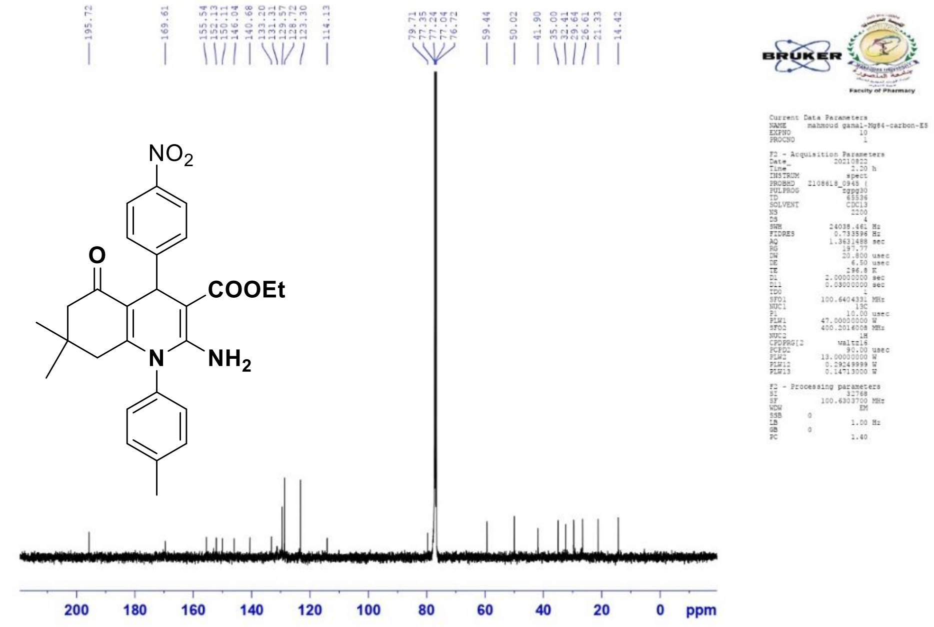

Figure S56. ^{13}C NMR (100 MHz, $CDCl_3$) spectrum of compound **8j**

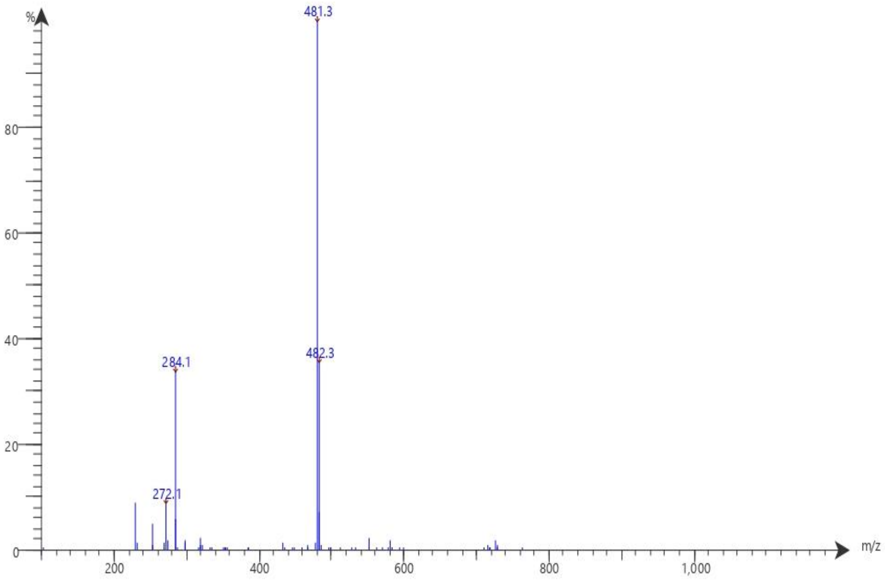

Figure S57. Mass spectrum (positive mode) of compound **8j**

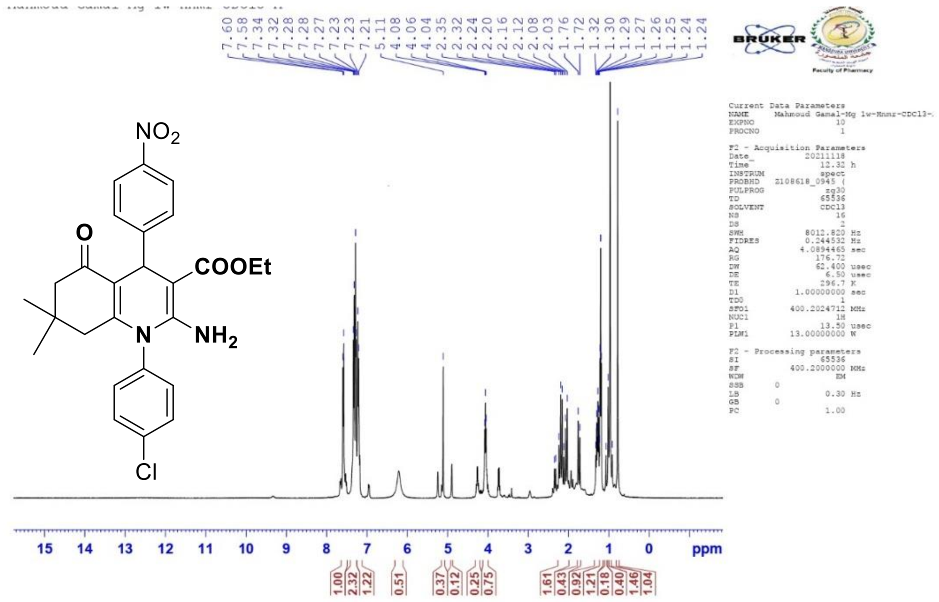

Figure S58. ^{1}H NMR (400 MHz, $CDCl_3$) spectrum of compound **8k**

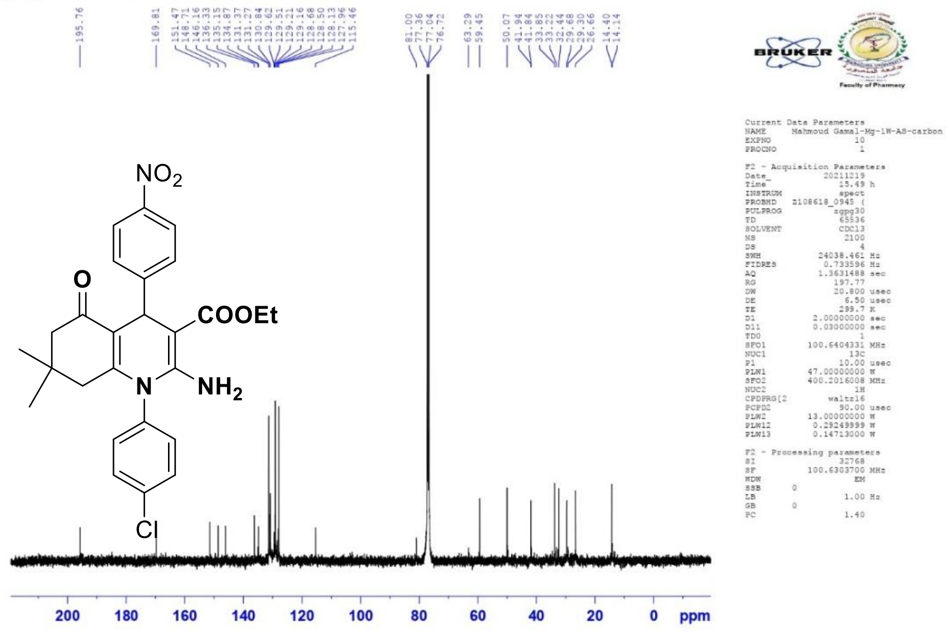

Figure S59. ^{13}C NMR (100 MHz, $CDCl_3$) spectrum of compound **8k**

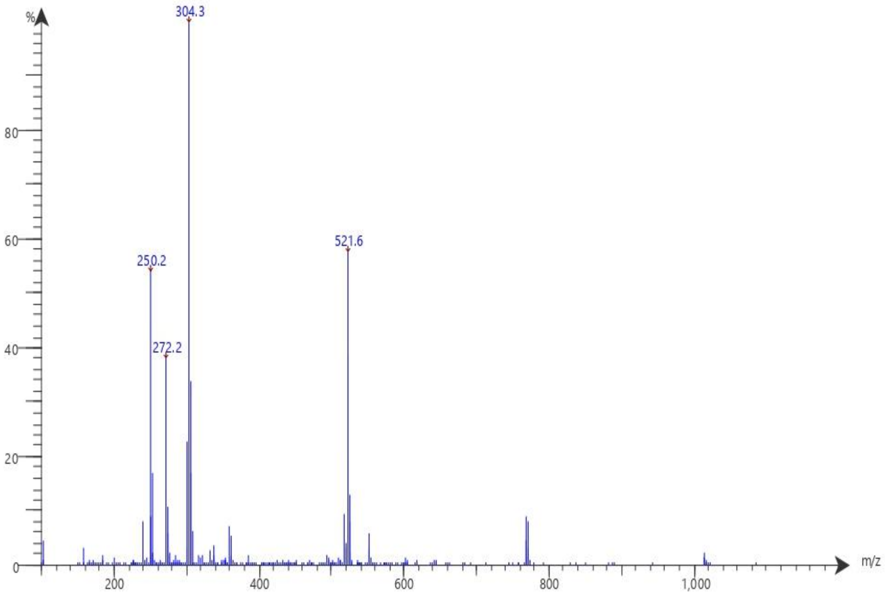

Figure S60. Mass spectrum (positive mode) of compound **8k**

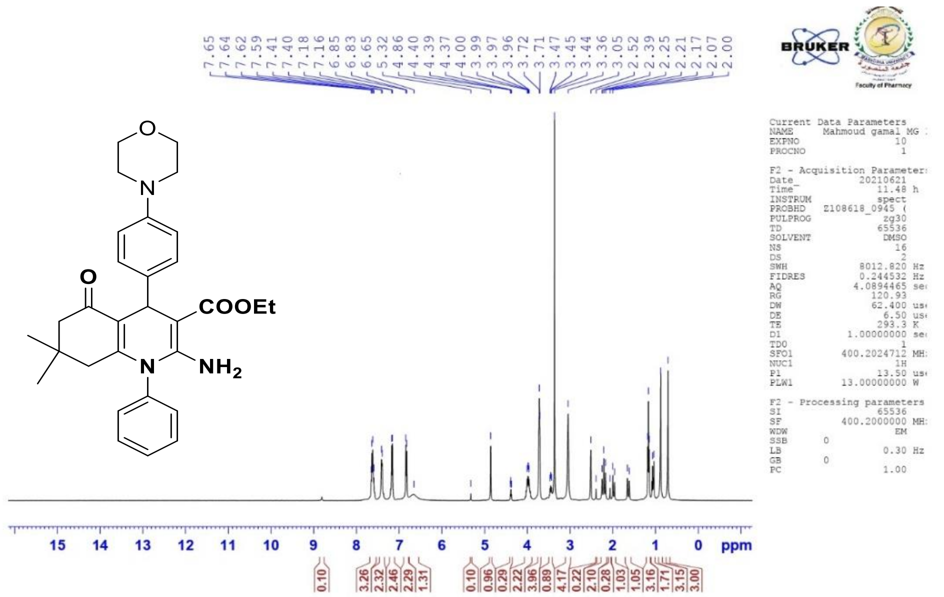

Figure S61. ^{1}H NMR (400 MHz, DMSO-d_6) spectrum of compound **8l**

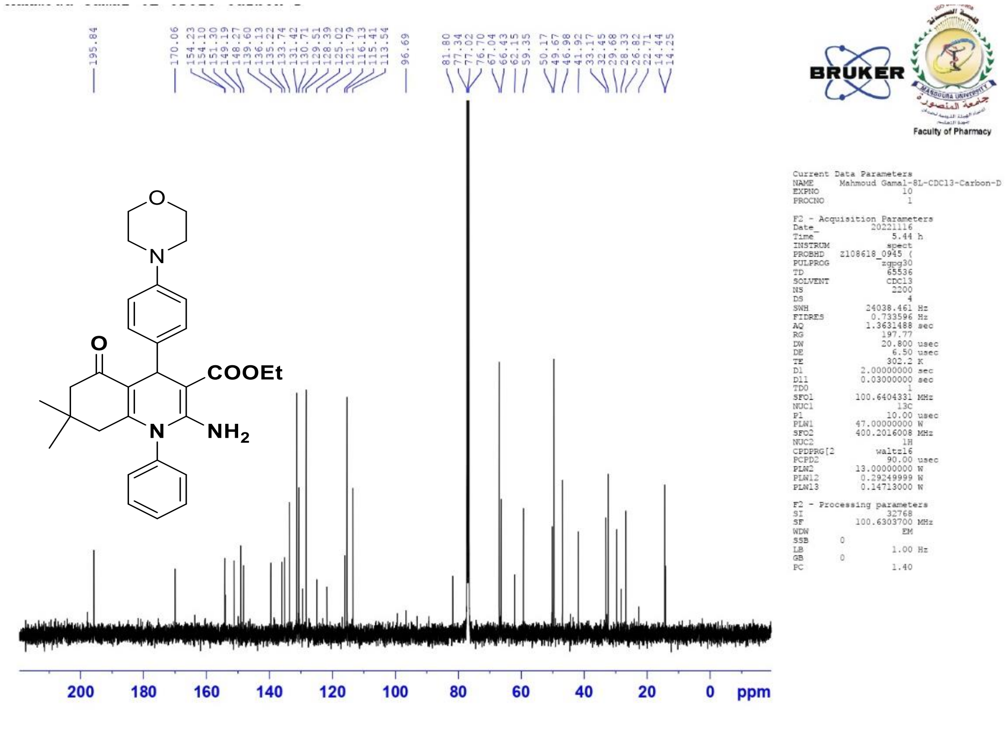

Figure S62. ^{13}C NMR (100 MHz, $CDCl_3$) spectrum of compound **8l**

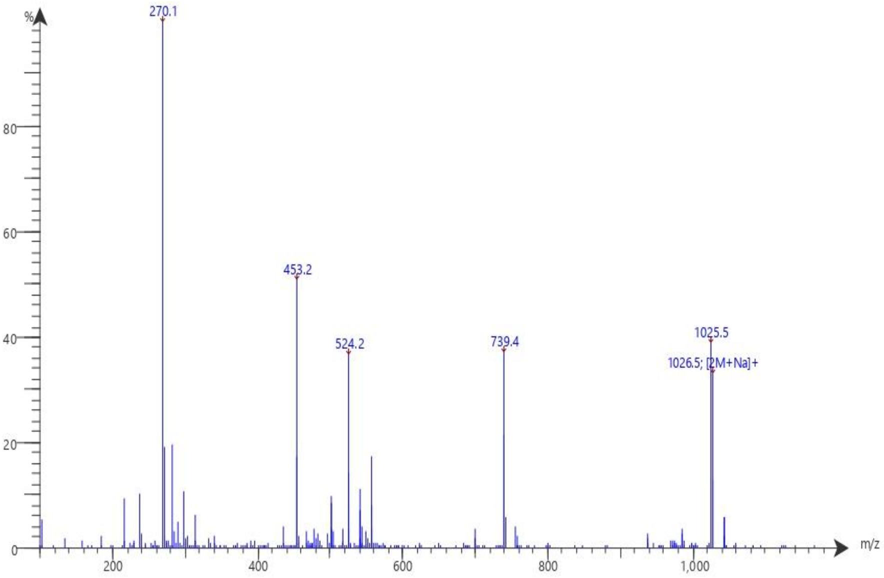

Figure S63. Mass spectrum (positive mode) of compound **8l**

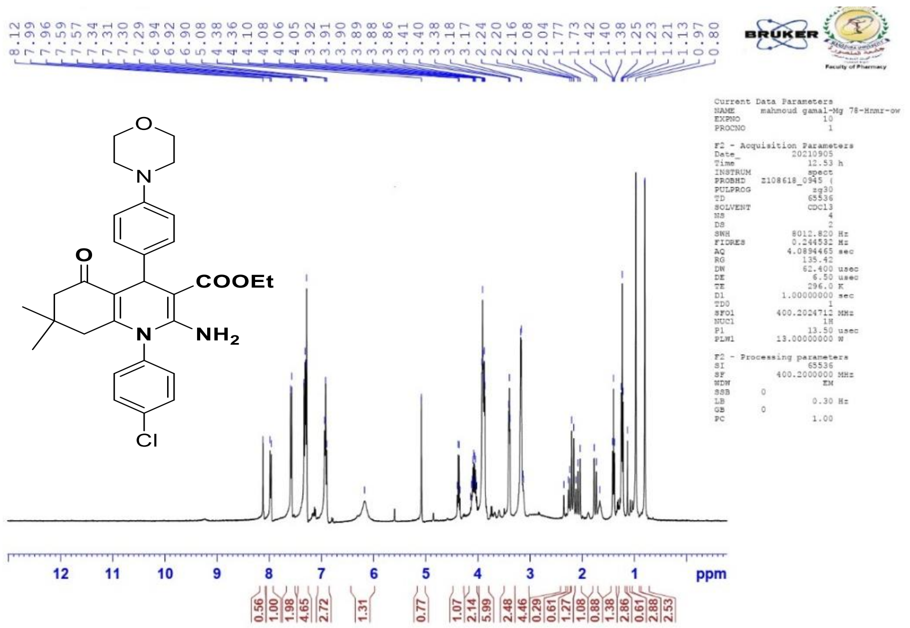

Figure S64. ^{1}H NMR (400 MHz, $CDCl_3$) spectrum of compound **8m**

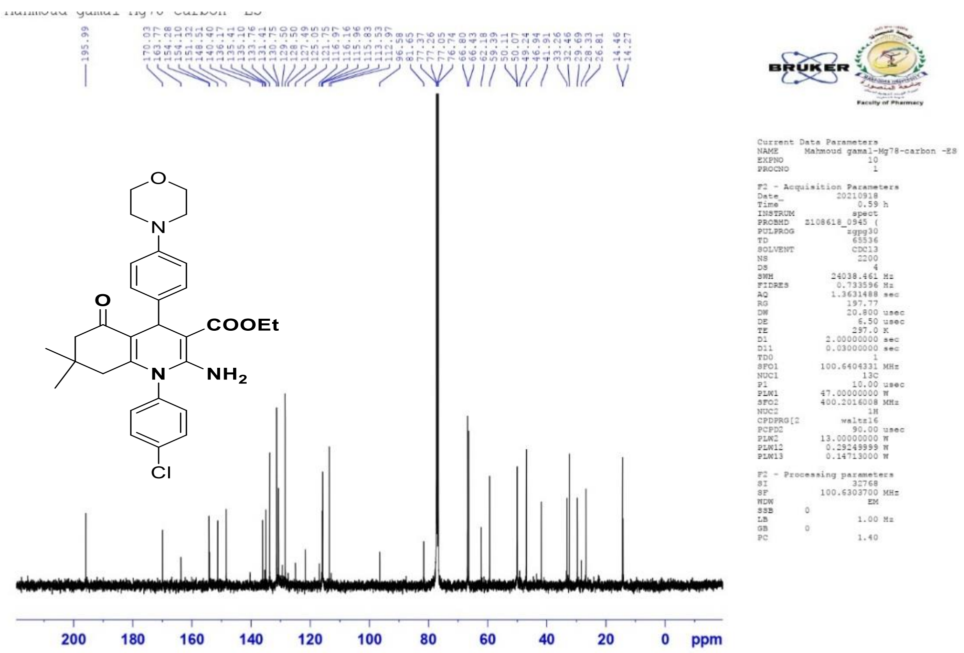

Figure S65. ^{13}C NMR (100 MHz, $CDCl_3$) spectrum of compound **8m**

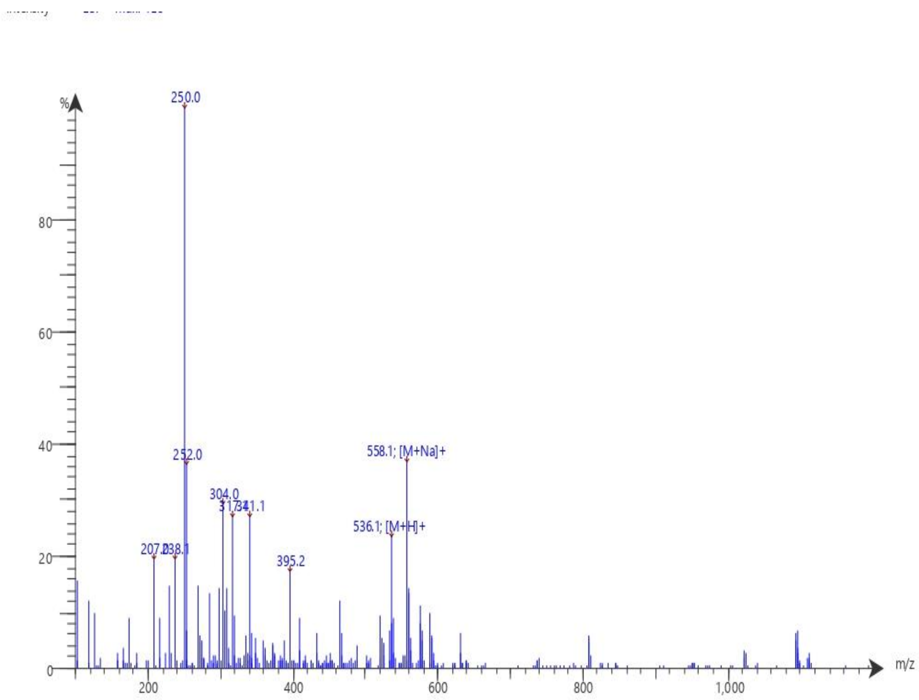

Figure S66. Mass spectrum (positive mode) of compound **8m**

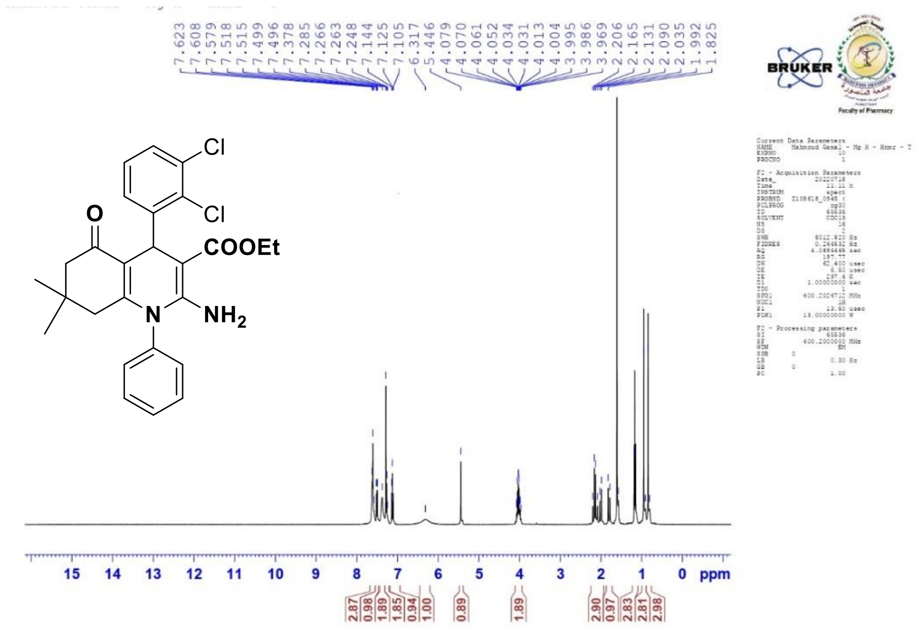

Figure S67. ^{1}H NMR (400 MHz, $CDCl_3$) spectrum of compound **10a**

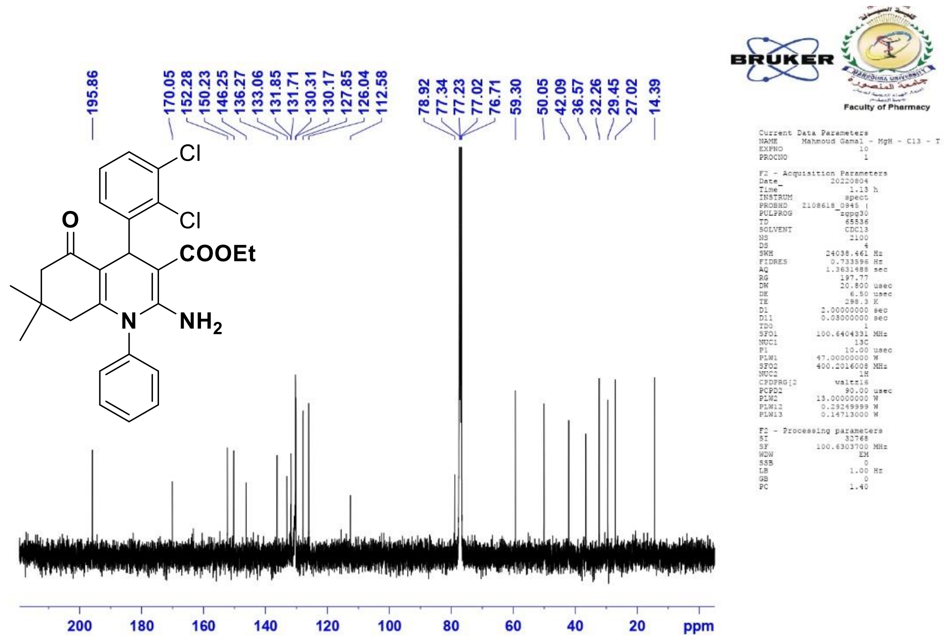

Figure S68. ^{13}C NMR (100 MHz, $CDCl_3$) spectrum of compound **10a**

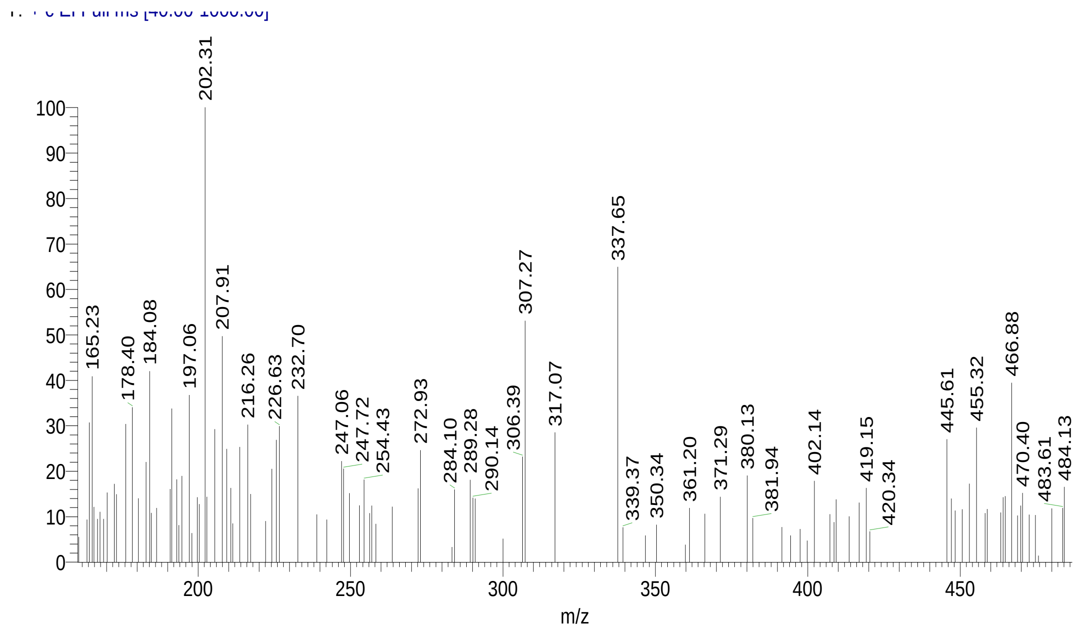

Figure S69. Mass spectrum of compound **10a**

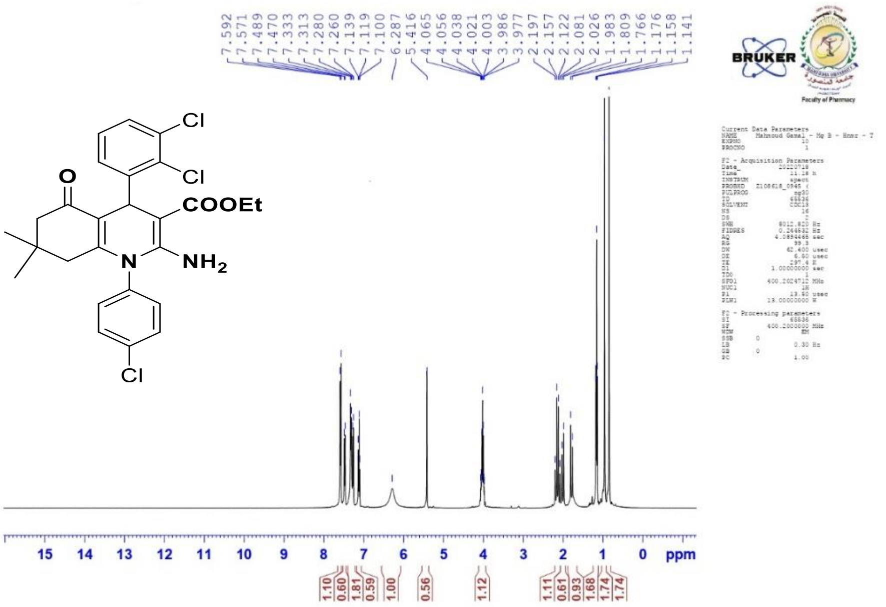

Figure S70. ^{1}H NMR (400 MHz, $CDCl_3$) spectrum of compound **10b**

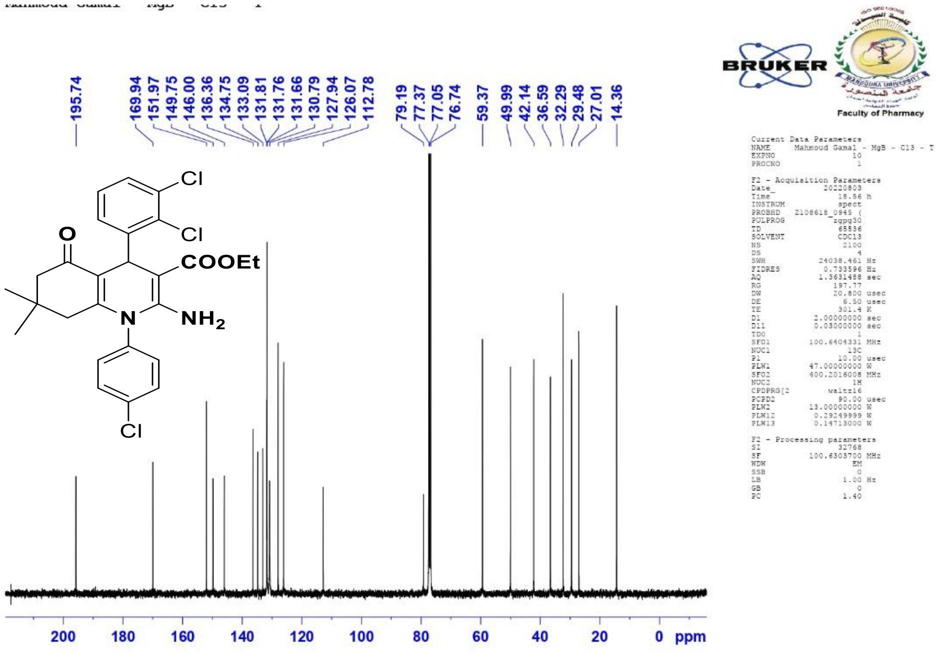

Figure S71. ^{13}C NMR (100 MHz, $CDCl_3$) spectrum of compound **10b**

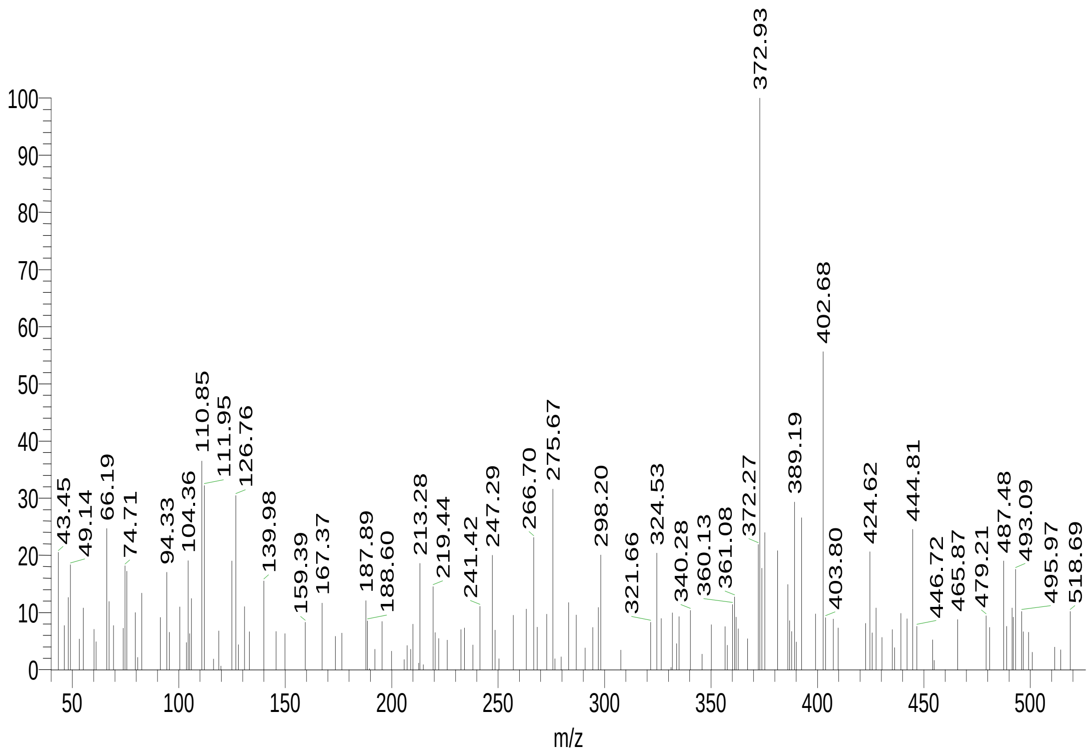

Figure S72. Mass spectrum of compound **10b**

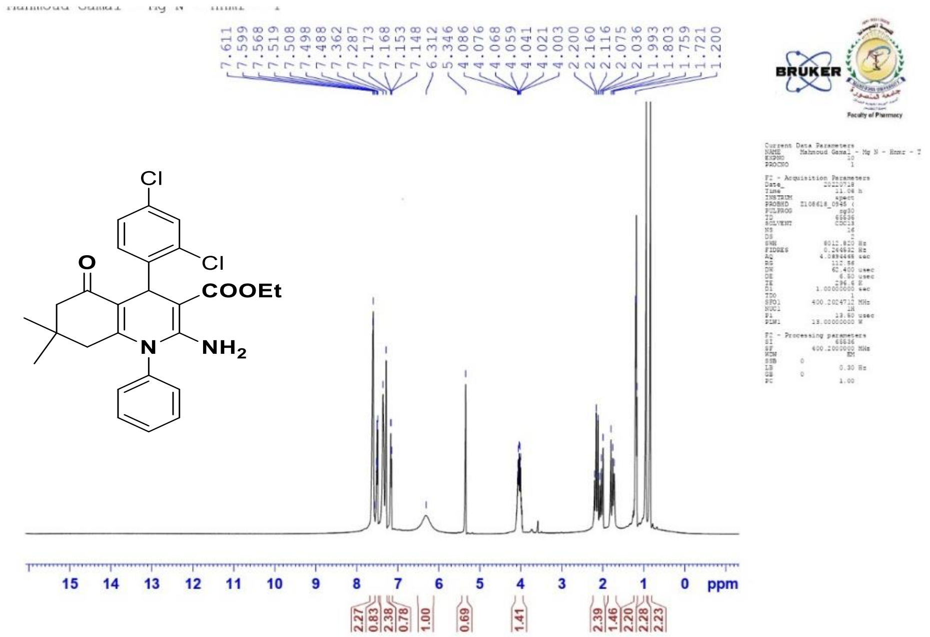

Figure S73. ^{1}H NMR (400 MHz, $CDCl_3$) spectrum of compound **10c**

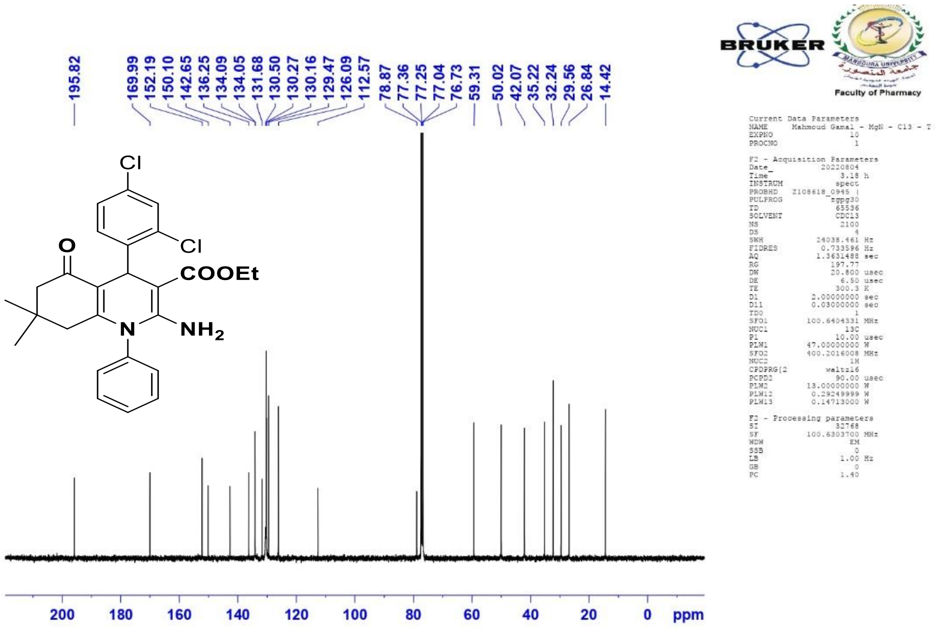

Figure S74. ^{13}C NMR (100 MHz, $CDCl_3$) spectrum of compound **10c**

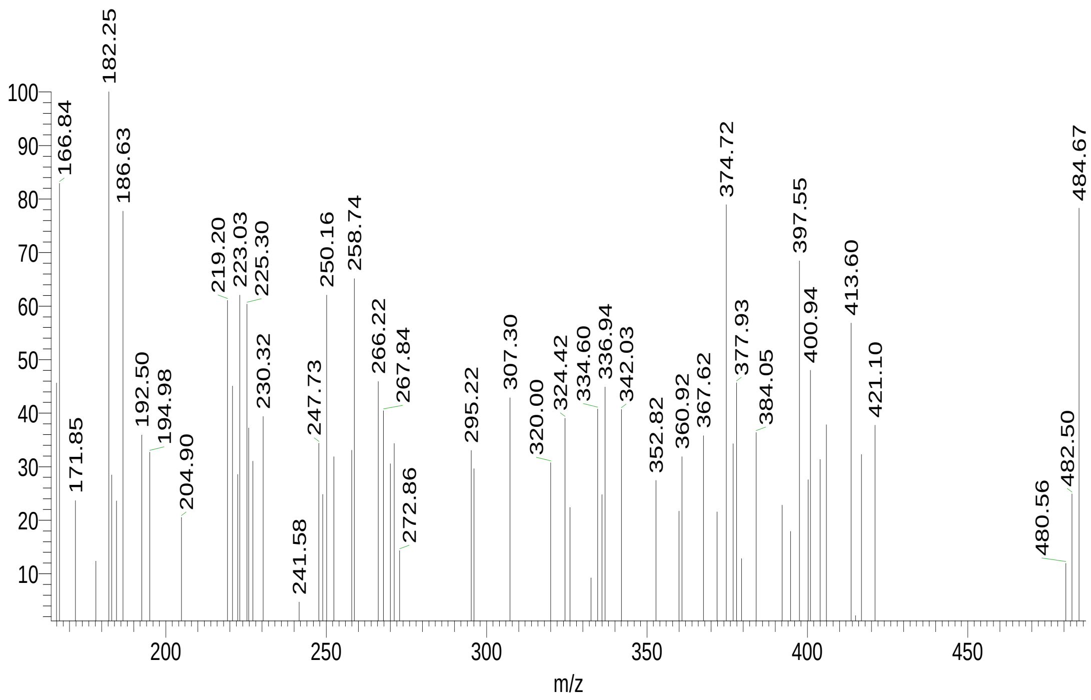

Figure S75. Mass spectrum of compound **10c**

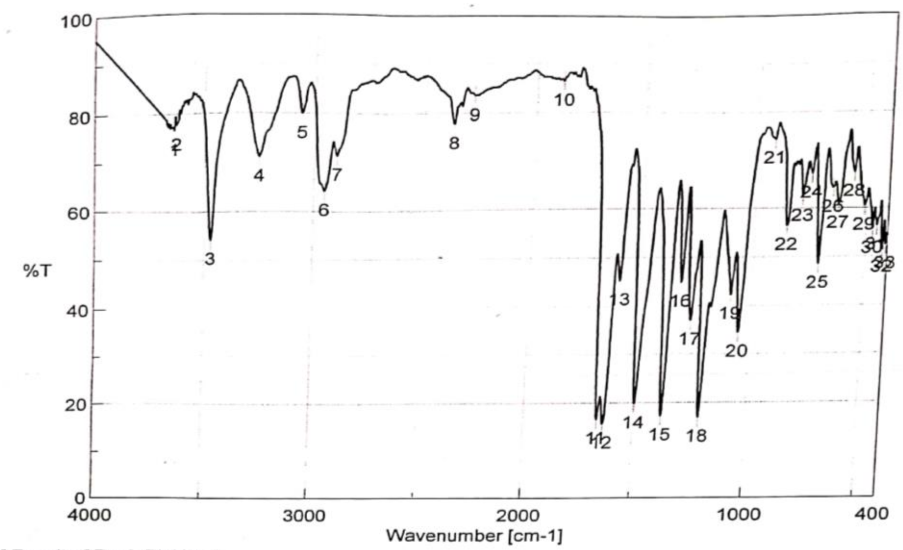

[Result of Peak Picking]

No.	Position	Intensity	No.	Position	Intensity
1	3640.95	76.6623	2	3630.34	77.7197
3	3472.2	54.2064	4	3251.4	71.1126
5	3059.51	79.9314	6	2948.63	64.244
7	2894.63	71.4272	8	2371.05	77.748
9	2283.3	83.4985	10	1881.22	86.4314
11	1662.34	16.5281	12	1634.38	15.6171
13	1589.06	45.3195	14	1495.53	19.7539
15	1374.03	17.2071	16	1315.21	44.8569
17	1266.04	36.9702	18	1209.15	16.8911
19	1093.44	42.2684	20	1051.01	34.4308
21	924.7	74.2857	22	857.204	56.4299
23	790.671	62.3789	24	754.031	67.1309
25	708.712	48.5777	26	655.679	64.1778
27	629.644	60.8856	28	561.184	67.3724
29	510.08	60.3982	30	469.582	55.6428
31	450.297	56.3551	32	422.334	51.728
33	407.871	52.4191			

Figure S76. IR spectrum of compound **10c**

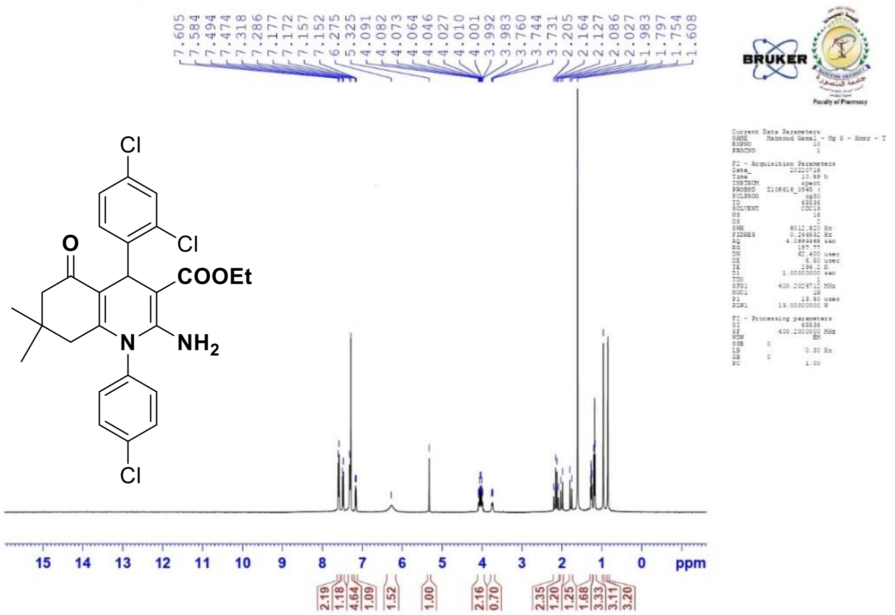

Figure S77. ^{1}H NMR (400 MHz, $CDCl_3$) spectrum of compound **10d**

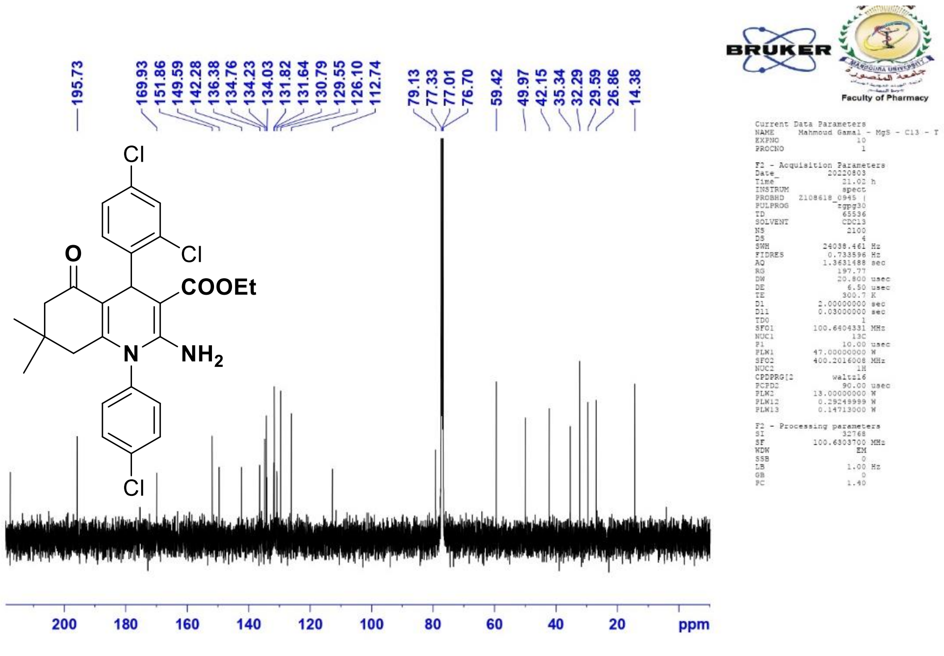

Figure S78. ^{13}C NMR (100 MHz, $CDCl_3$) spectrum of compound **10d**

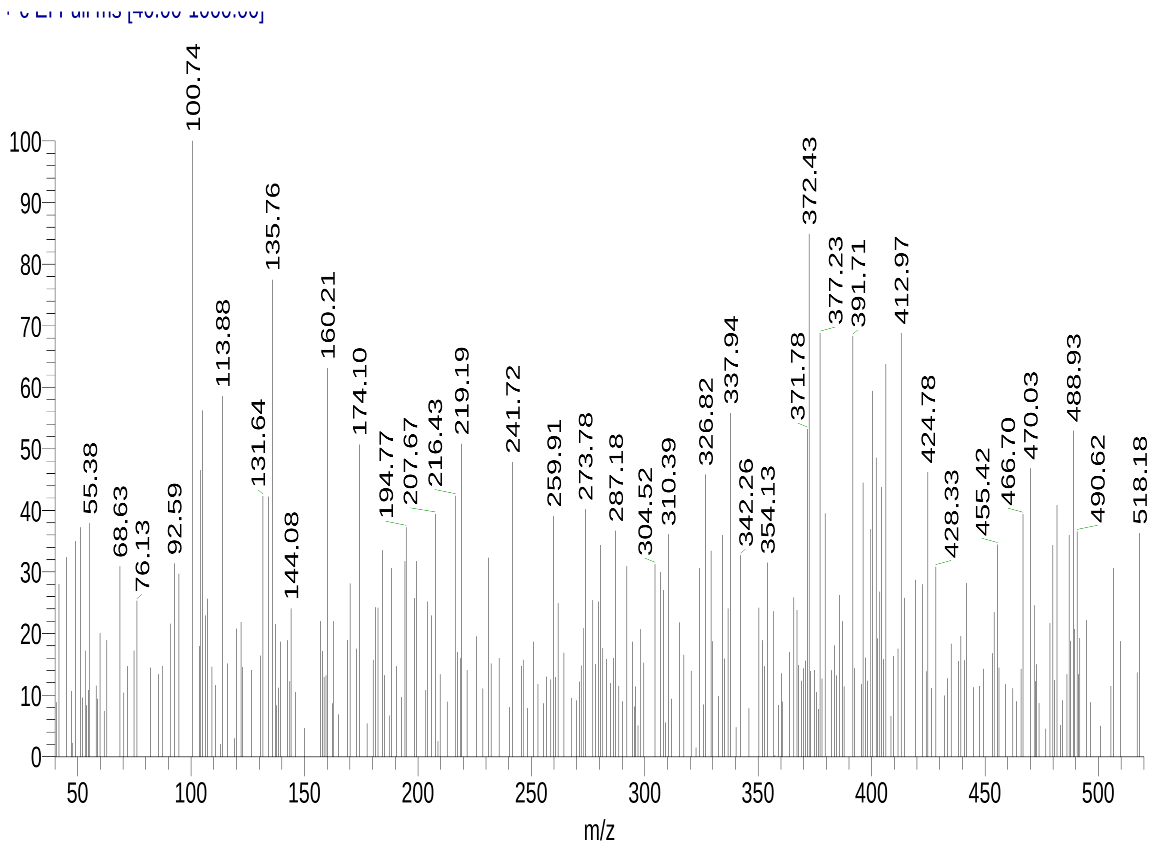

Figure S79. Mass spectrum of compound **10d**

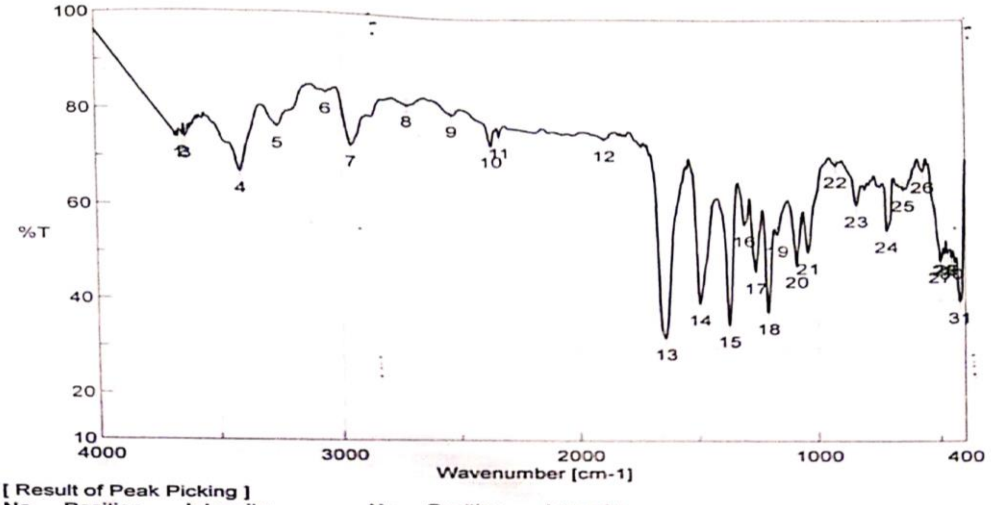

[Result of Peak Picking]

No.	Position	Intensity	No.	Position	Intensity
1	3660.23	73.7953	2	3640.95	74.1416
3	3629.37	73.8525	4	3408.57	66.6529
5	3257.18	75.9841	6	3061.44	83.4378
7	2960.2	72.4589	8	2723	81.3399
9	2534.97	79.3123	10	2371.05	72.7292
11	2336.34	74.6472	12	1900.5	73.9385
13	1640.16	31.9884	14	1497.45	39.2742
15	1373.07	34.7263	16	1313.29	55.8439
17	1264.11	45.9394	18	1210.11	37.3972
19	1175.4	53.7691	20	1092.48	47.2702
21	1046.19	50.0917	22	935.306	68.367
23	848.525	60.0454	24	724.139	54.5928
25	657.607	63.3684	26	575.647	67.3095
27	503.33	48.1559	28	486.938	49.719
29	474.403	49.7031	30	458.011	48.9527
31	422.334	39.6524			

Figure S80. IR spectrum of compound **10d**

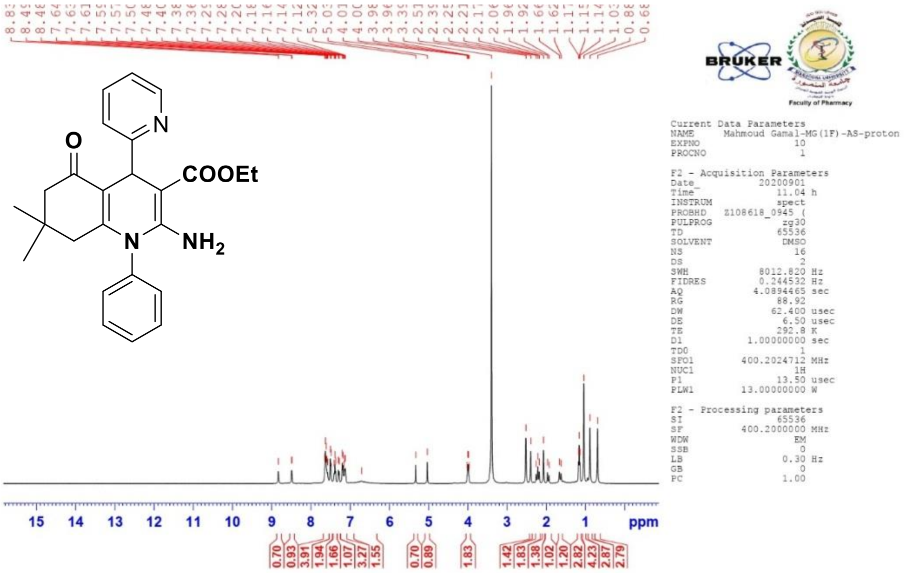

Figure S81. ^{1}H NMR (400 MHz, DMSO-d_6) spectrum of compound **12a**

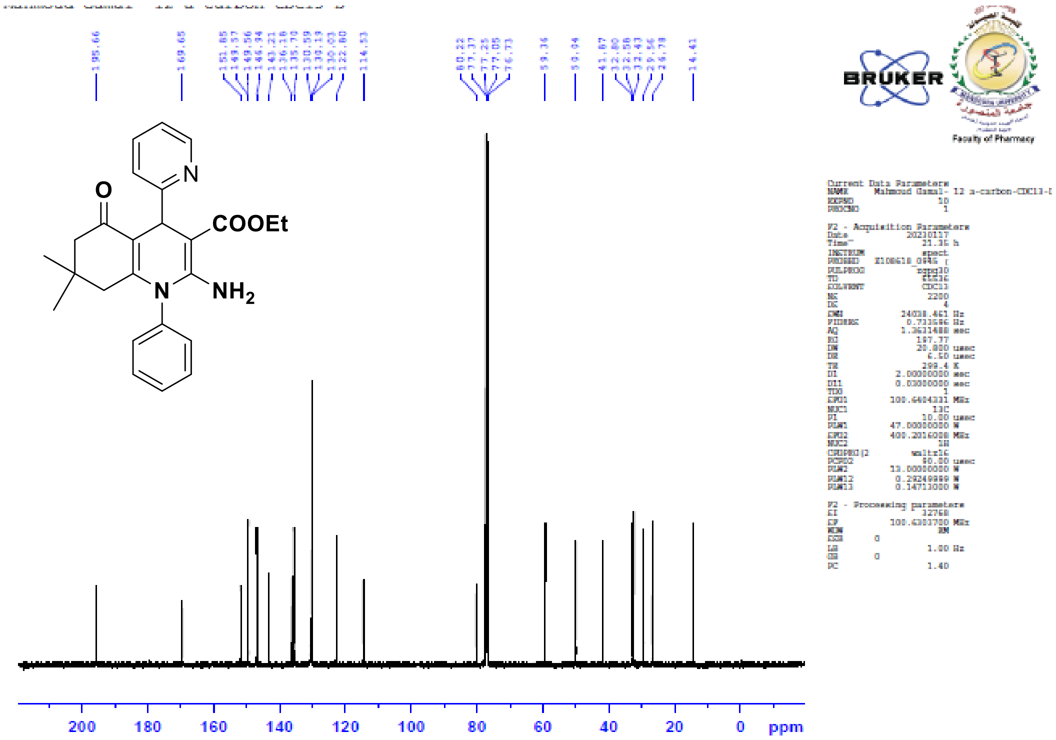

Figure S82. ^{13}C NMR (100 MHz, $CDCl_3$) spectrum of compound **12a**

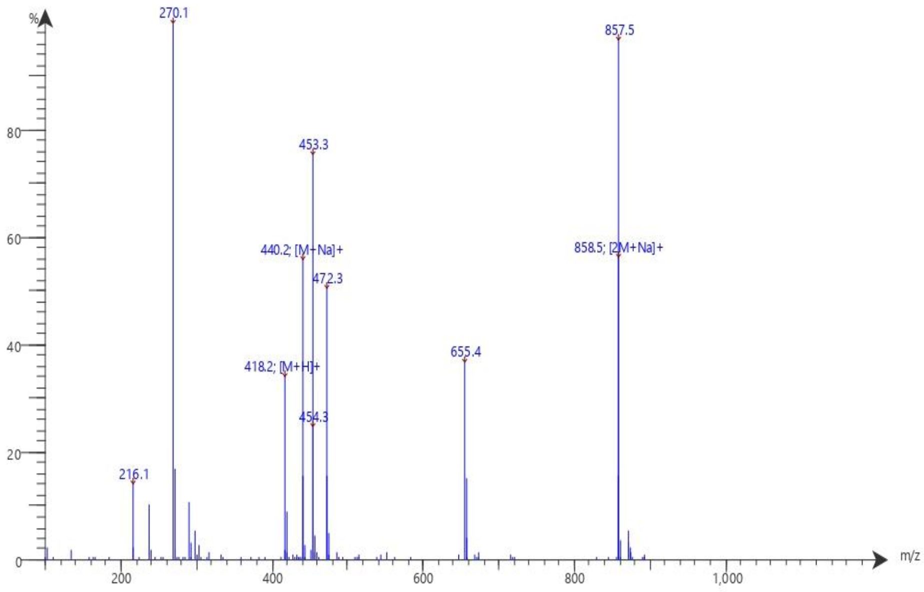

Figure S83. Mass spectrum (positive mode) of compound **12a**

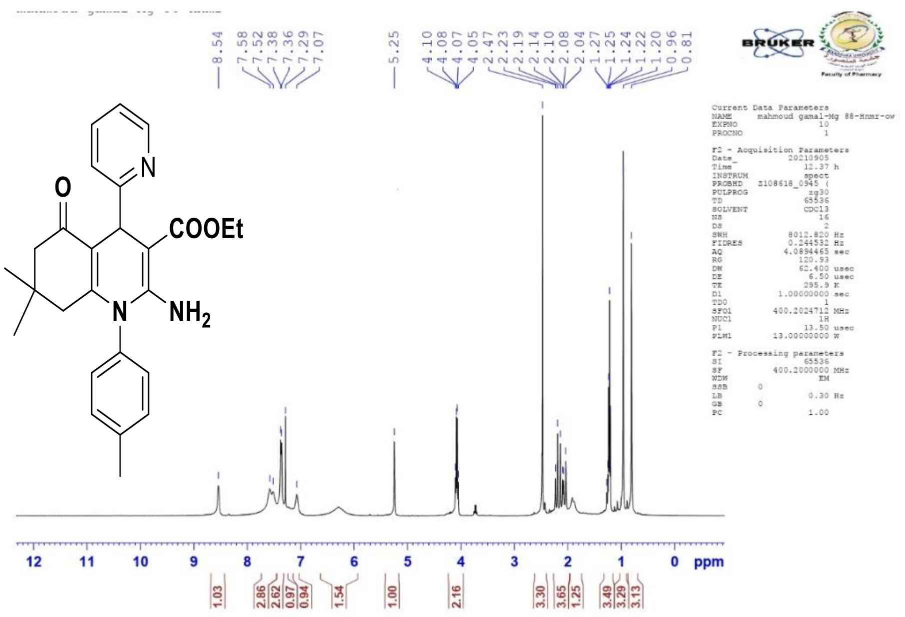

Figure S84. ^{1}H NMR (400 MHz, $CDCl_3$) spectrum of compound **12b**

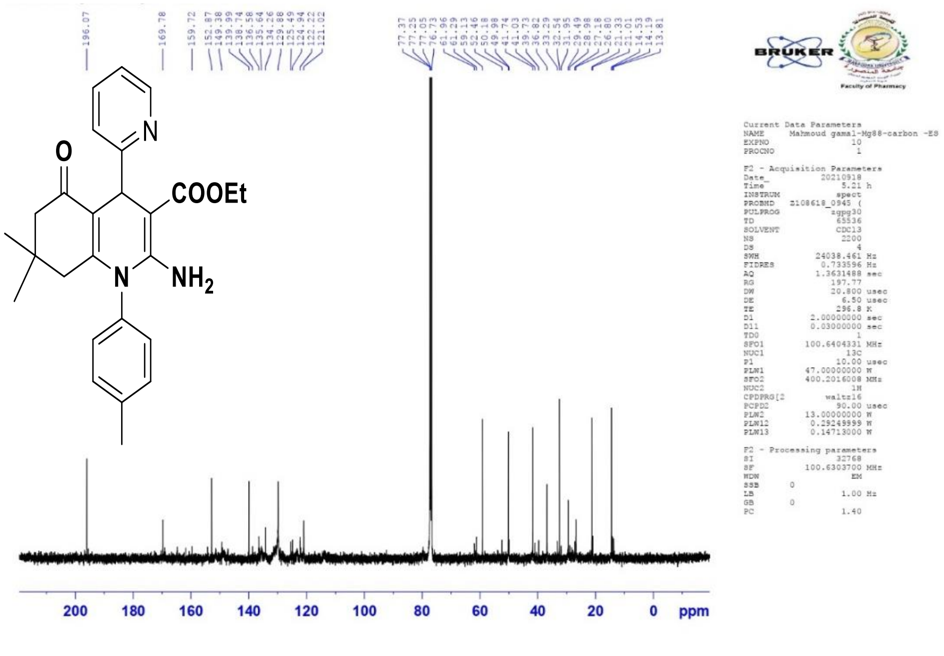

Figure S85. ^{13}C NMR (100 MHz, $CDCl_3$) spectrum of compound **12b**

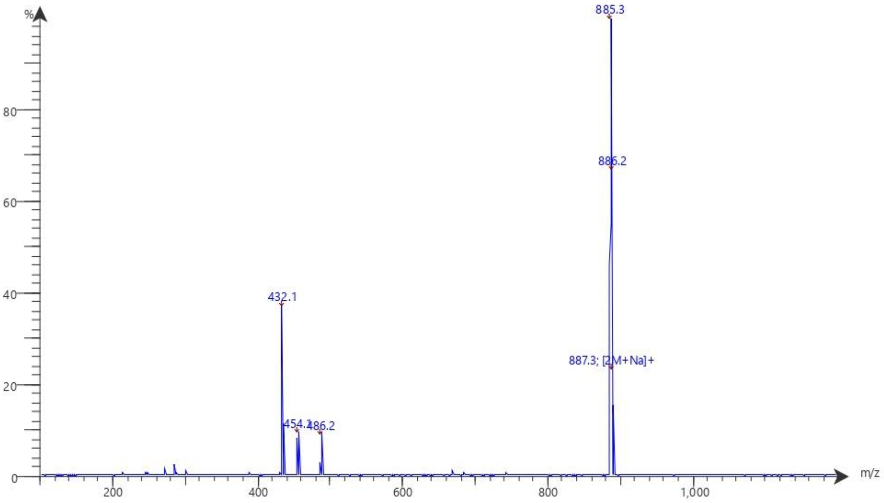

Figure S86. Mass spectrum (positive mode) of compound **12b**

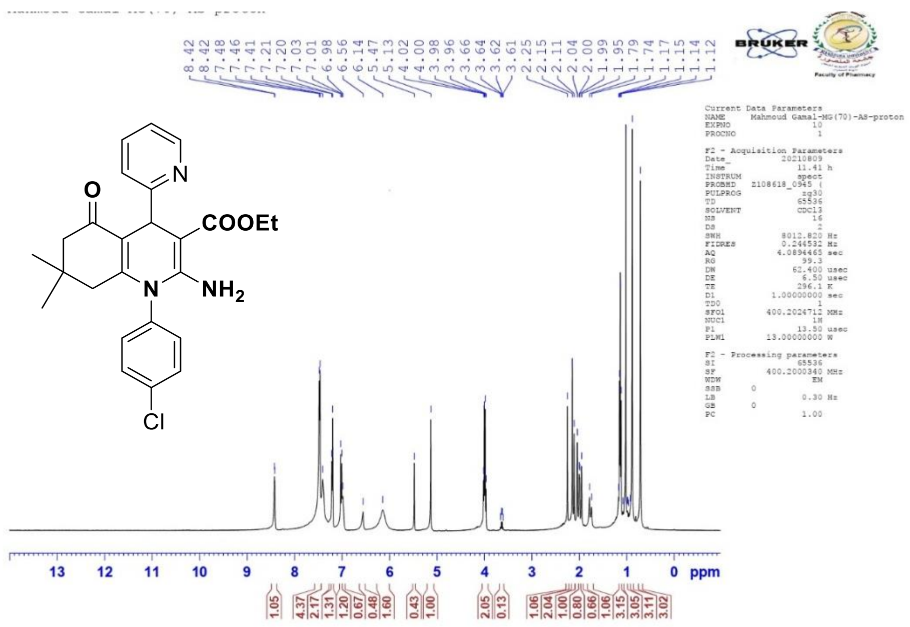

Figure S87. ^{1}H NMR (400 MHz, $CDCl_3$) spectrum of compound **12c**

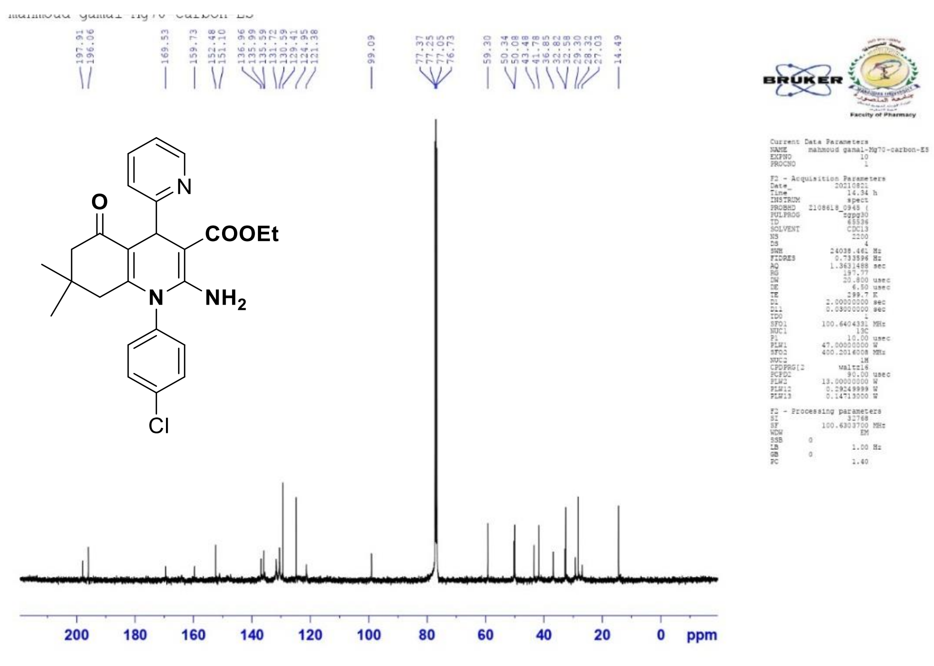

Figure S88. ^{13}C NMR (100 MHz, $CDCl_3$) spectrum of compound **12c**

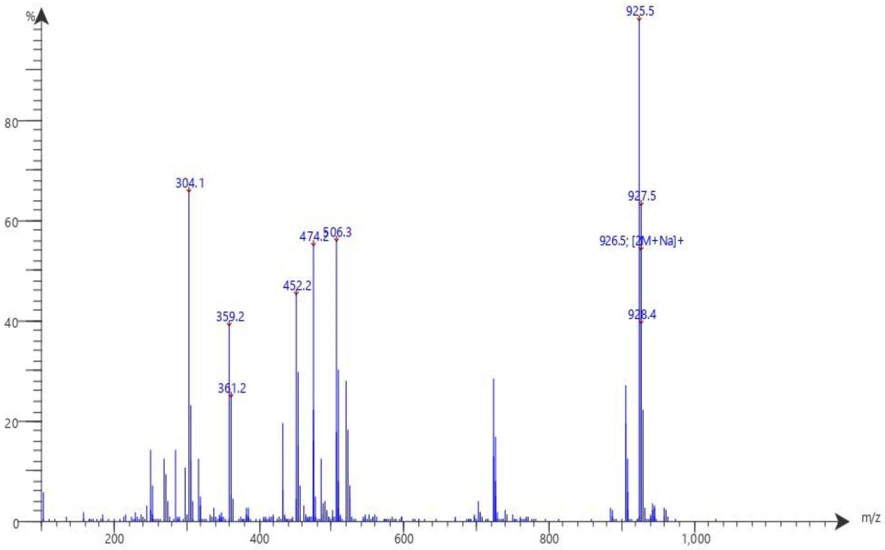

Figure S89. Mass spectrum (positive mode) of compound **12c**

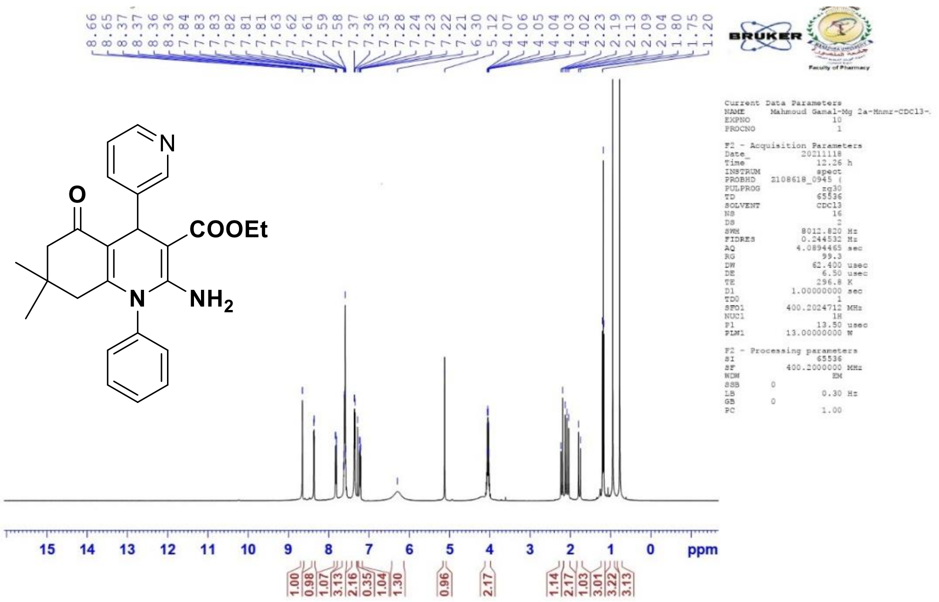

Figure S90. ^{1}H NMR (400 MHz, $CDCl_3$) spectrum of compound **12d**

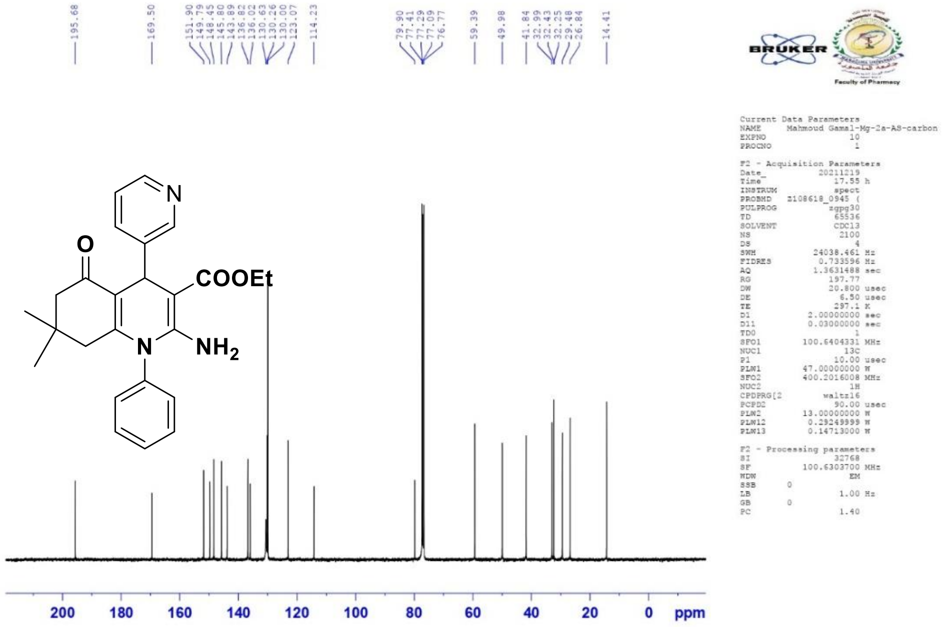

Figure S91. ^{13}C NMR (100 MHz, $CDCl_3$) spectrum of compound **12d**

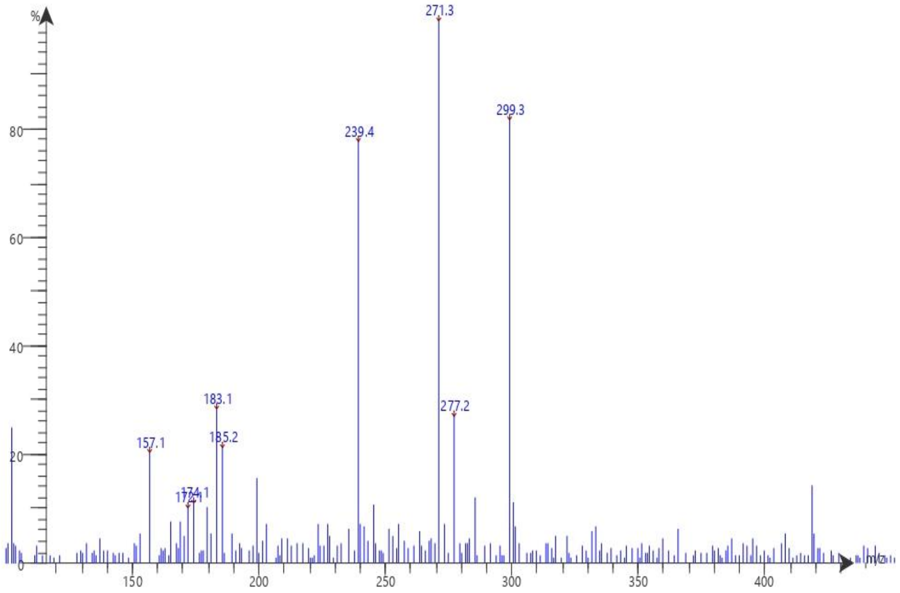

Figure S92. Mass spectrum (positive mode) of compound **12d**

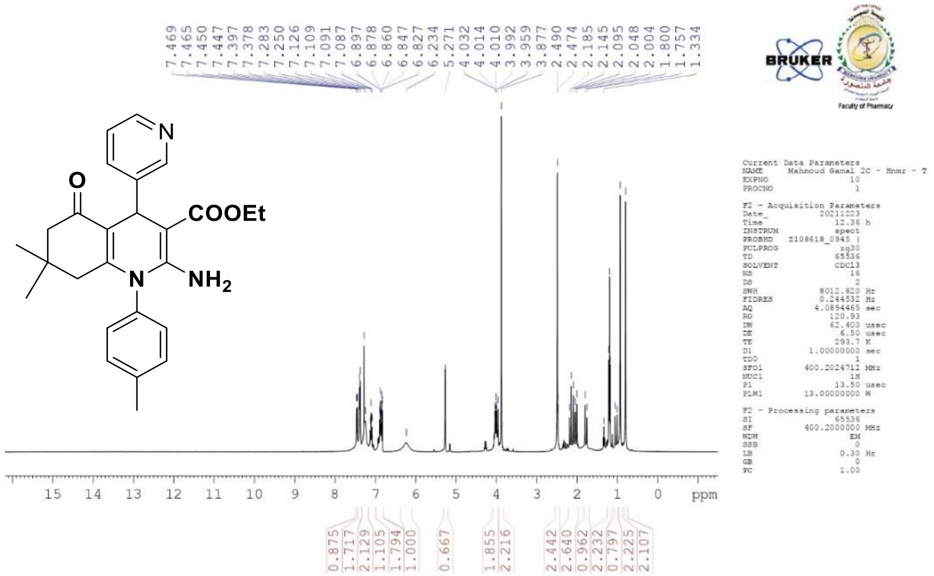

Figure S93. ^{1}H NMR (400 MHz, $CDCl_3$) spectrum of compound **12e**

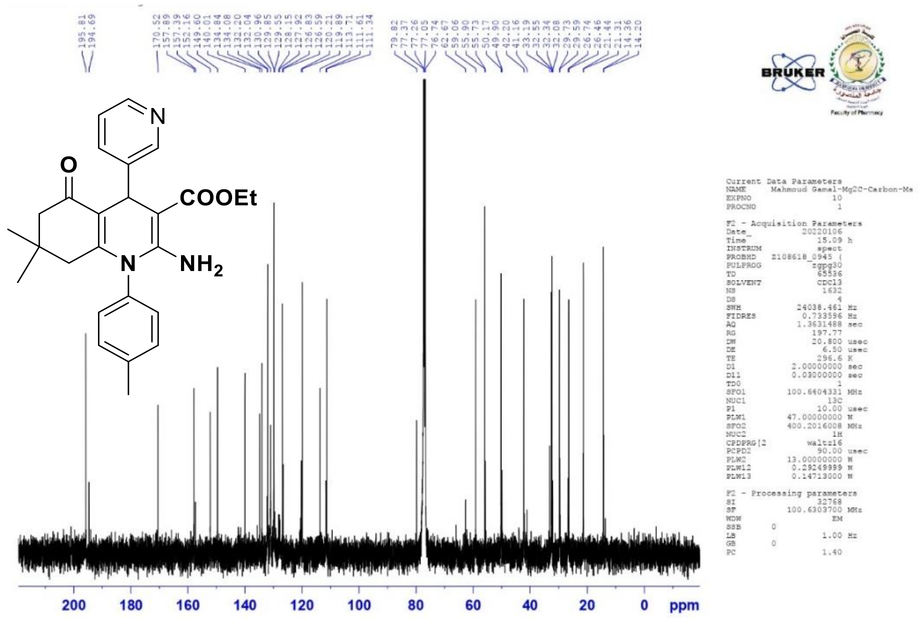

Figure S94. ^{13}C NMR (100 MHz, $CDCl_3$) spectrum of compound **12e**

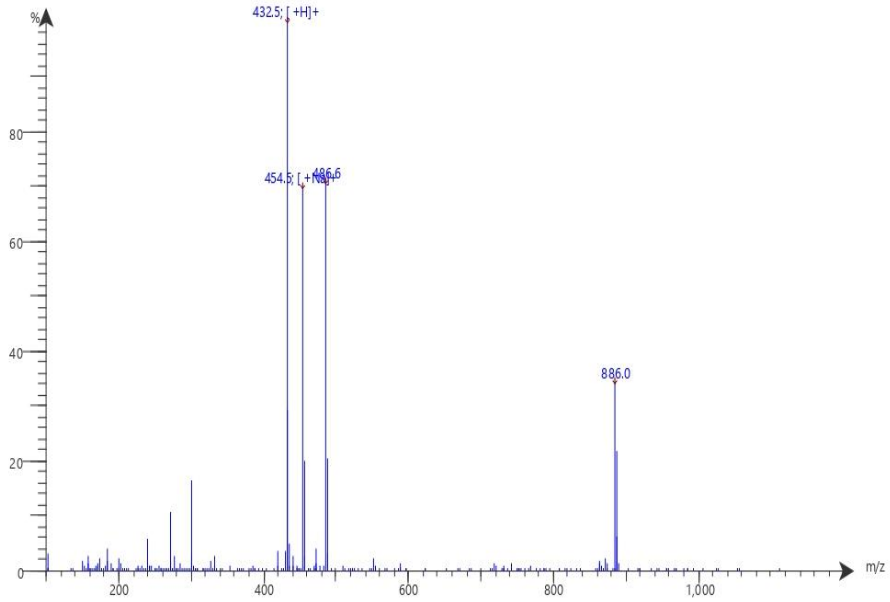

Figure S95. Mass spectrum (positive mode) of compound **12e**

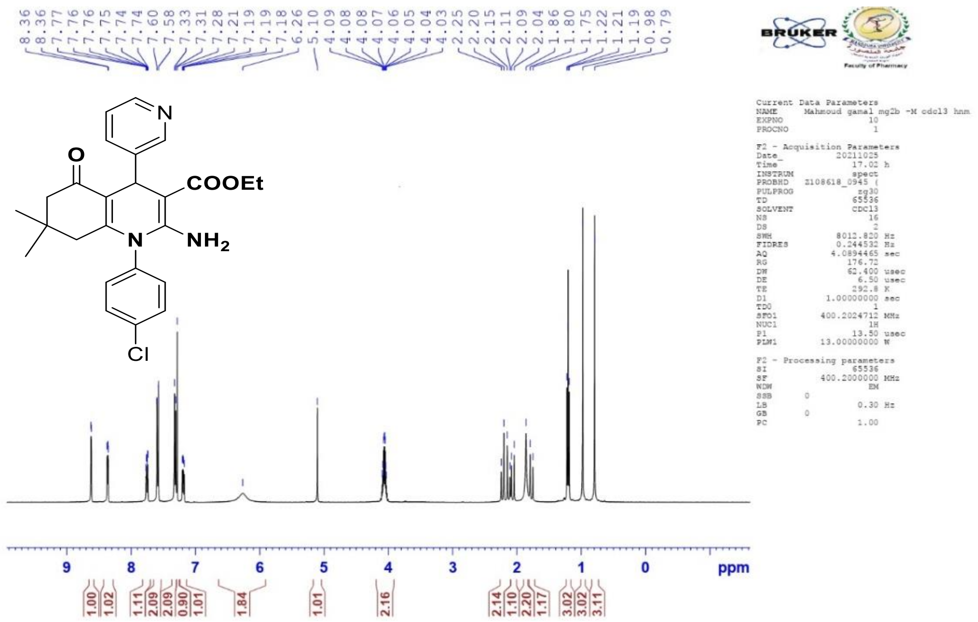

Figure S96. ^{1}H NMR (400 MHz, $CDCl_3$) spectrum of compound **12f**

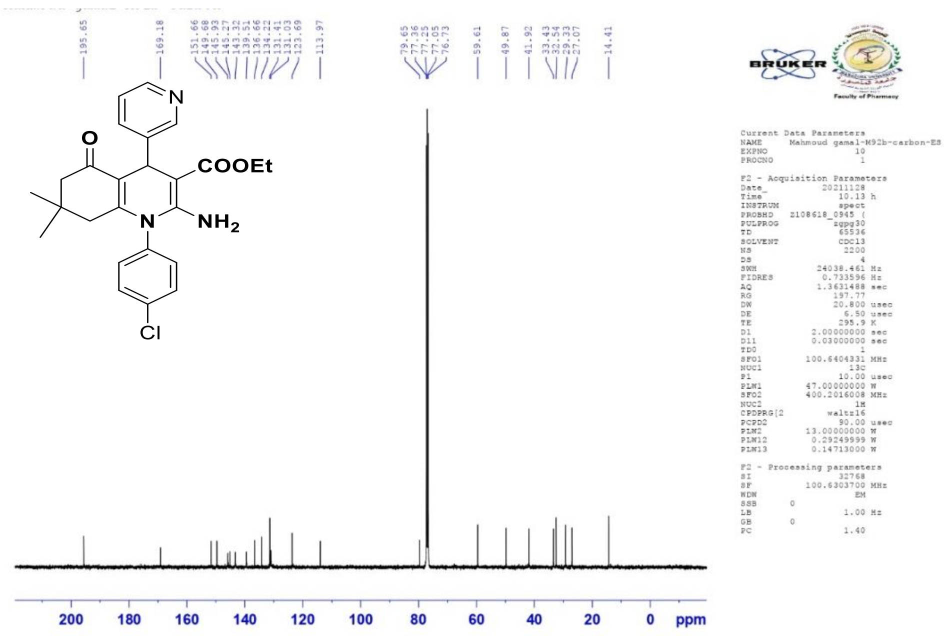

Figure S97. ^{13}C NMR (100 MHz, $CDCl_3$) spectrum of compound **12f**

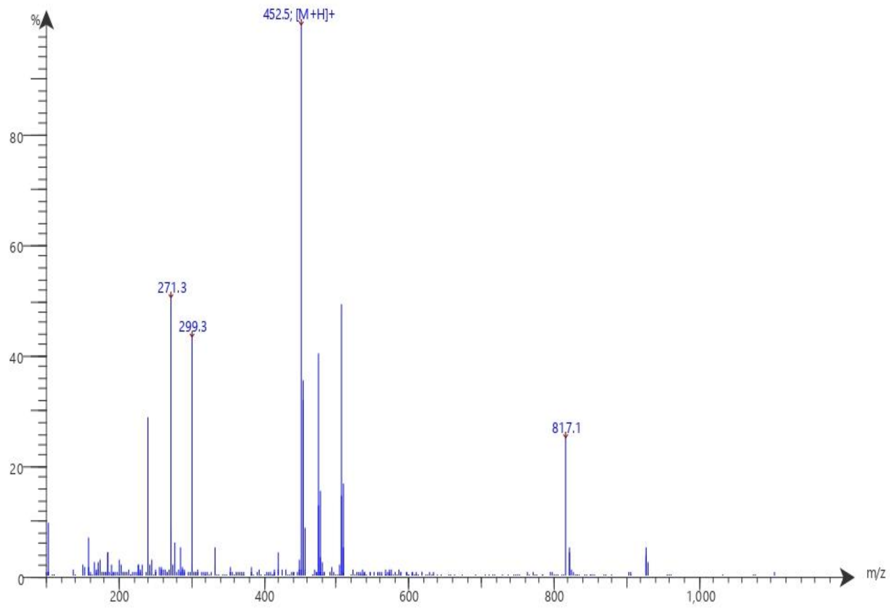

Figure S98. Mass spectrum (positive mode) of compound **12f**

Developmental Therapeutics Program One Dose Mean Graph	NSC: D-832911 / 1	Conc: 1.00E-5 Molar	Test Date: Oct 18, 2021
	Experiment ID: 2110OS01		Report Date: Apr 01, 2022

Panel/Cell Line	Growth Percent
**Leukemia**	
CCRF-CEM	48.35
HL-60(TB)	-23.03
K-562	41.53
MOLT-4	25.83
RPMI-8226	22.55
SR	32.47
**Non-Small Cell Lung Cancer**	
A549/ATCC	75.64
EKVX	74.61
HOP-62	94.09
HOP-92	68.94
NCI-H226	76.34
NCI-H322M	82.18
NCI-H460	60.21
NCI-H522	57.54
**Colon Cancer**	
COLO 205	65.34
HCC-2998	85.62
HCT-116	41.27
HCT-15	49.08
HT29	55.02
KM12	58.44
SW-620	74.61
**CNS Cancer**	
SF-268	80.00
SF-295	75.03
SF-539	100.22
SNB-19	70.45
SNB-75	66.30
U251	79.35
**Melanoma**	
LOX IMVI	85.44
MALME-3M	89.32
M14	50.80
MDA-MB-435	73.10
SK-MEL-2	57.66
SK-MEL-28	81.38
SK-MEL-5	60.49
UACC-257	72.52
UACC-62	44.05
**Ovarian Cancer**	
IGROV1	92.05
OVCAR-3	62.67
OVCAR-4	74.63
OVCAR-5	86.78
OVCAR-8	83.65
NCI/ADR-RES	73.97
SK-OV-3	90.08
**Renal Cancer**	
786-0	79.36
A498	99.35
ACHN	69.24
CAKI-1	46.71
RXF 393	78.22
SN12C	75.04
TK-10	100.40
UO-31	55.35
**Prostate Cancer**	
PC-3	29.65
DU-145	88.25
**Breast Cancer**	
MCF7	56.51
MDA-MB-231/ATCC	84.82
HS 578T	93.36
BT-549	78.60
T-47D	52.01
MDA-MB-468	50.23
Mean	67.01
Delta	90.04
Range	123.43

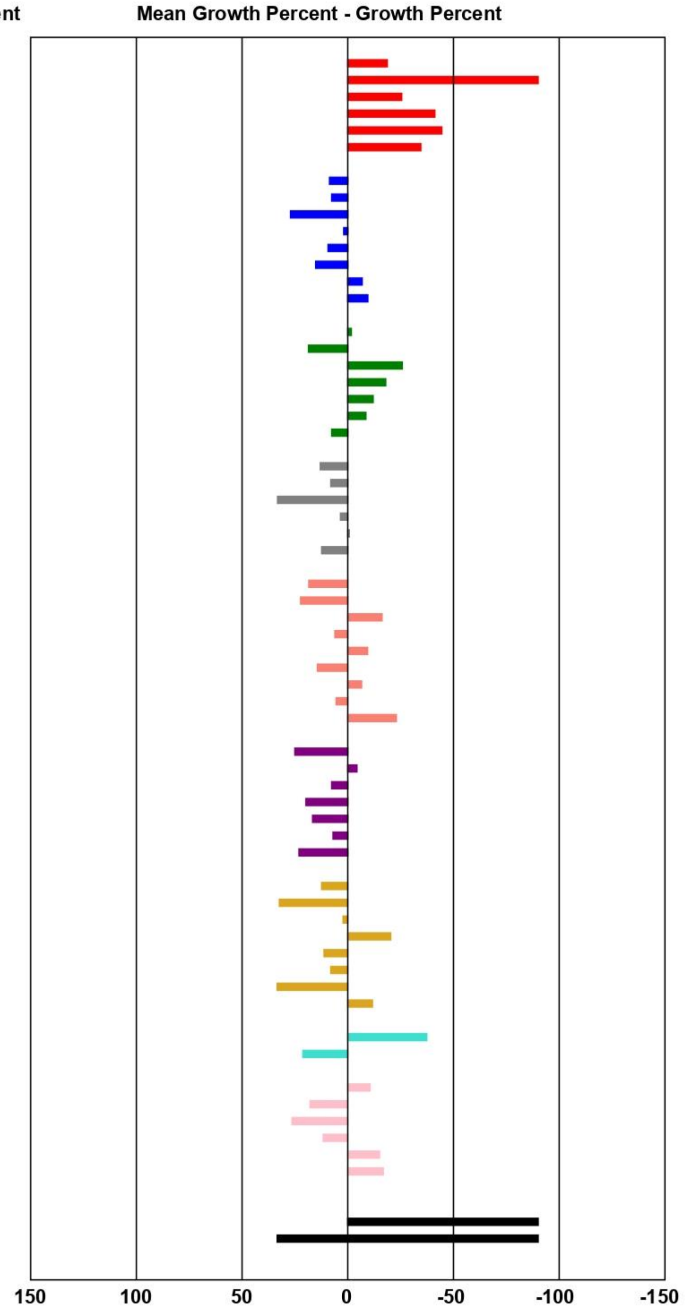

Figure S99. One dose mean graph for compound **6a** (NSC 832911) at 10 μM

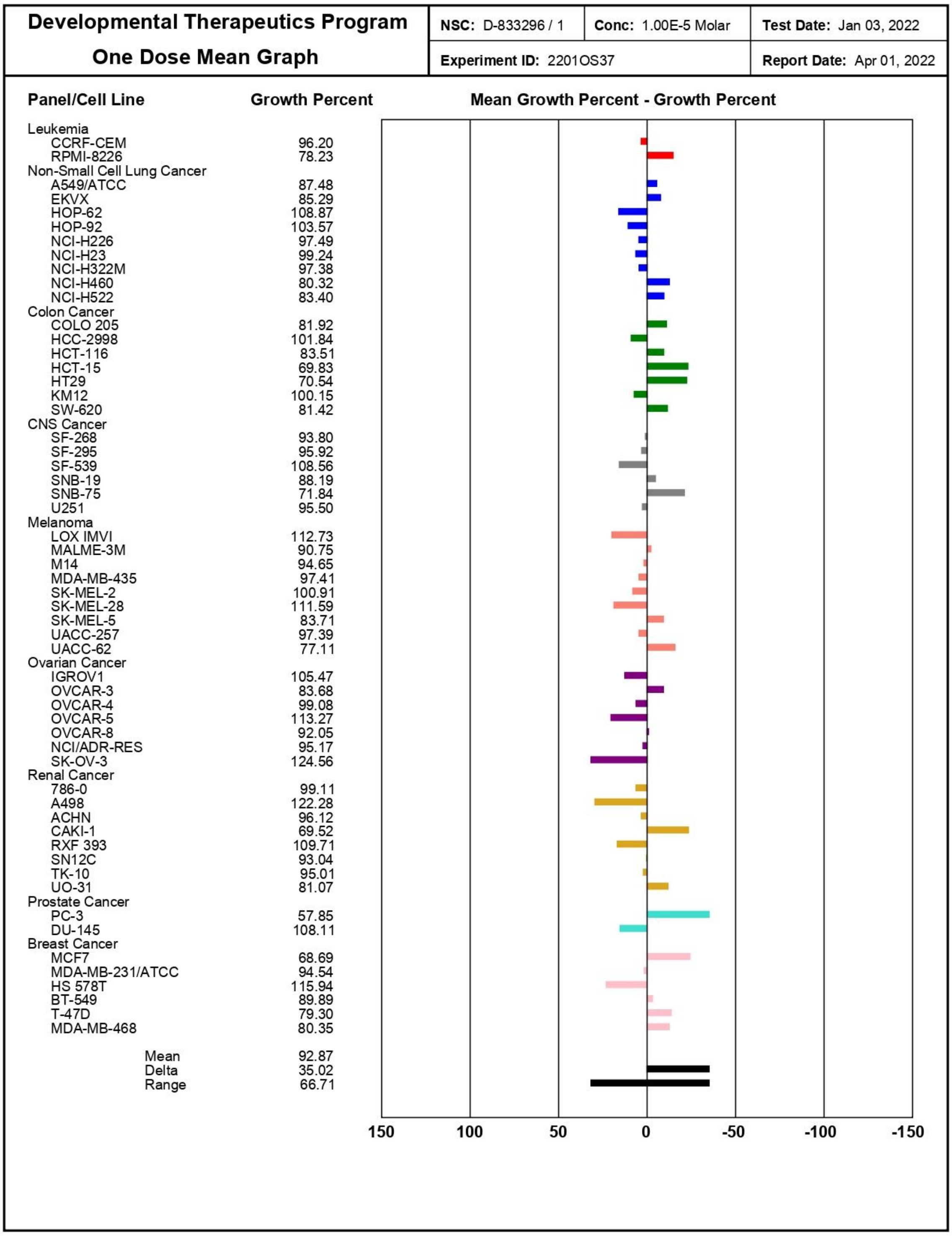

Figure S100. One dose mean graph for compound **6b** (NSC 833296) at 10 μM

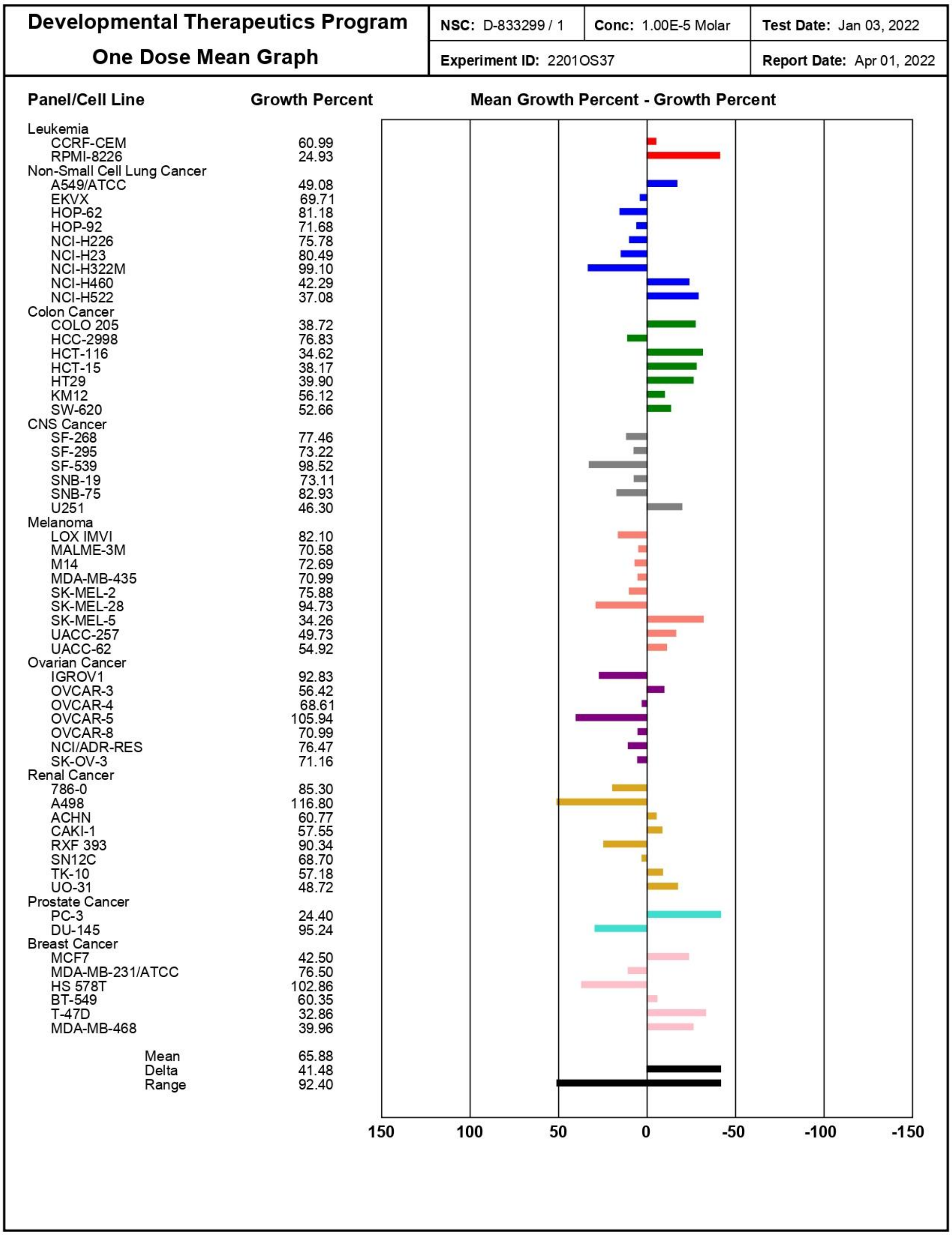

Figure S101. One dose mean graph for compound **6c** (NSC 833299) at 10 μM

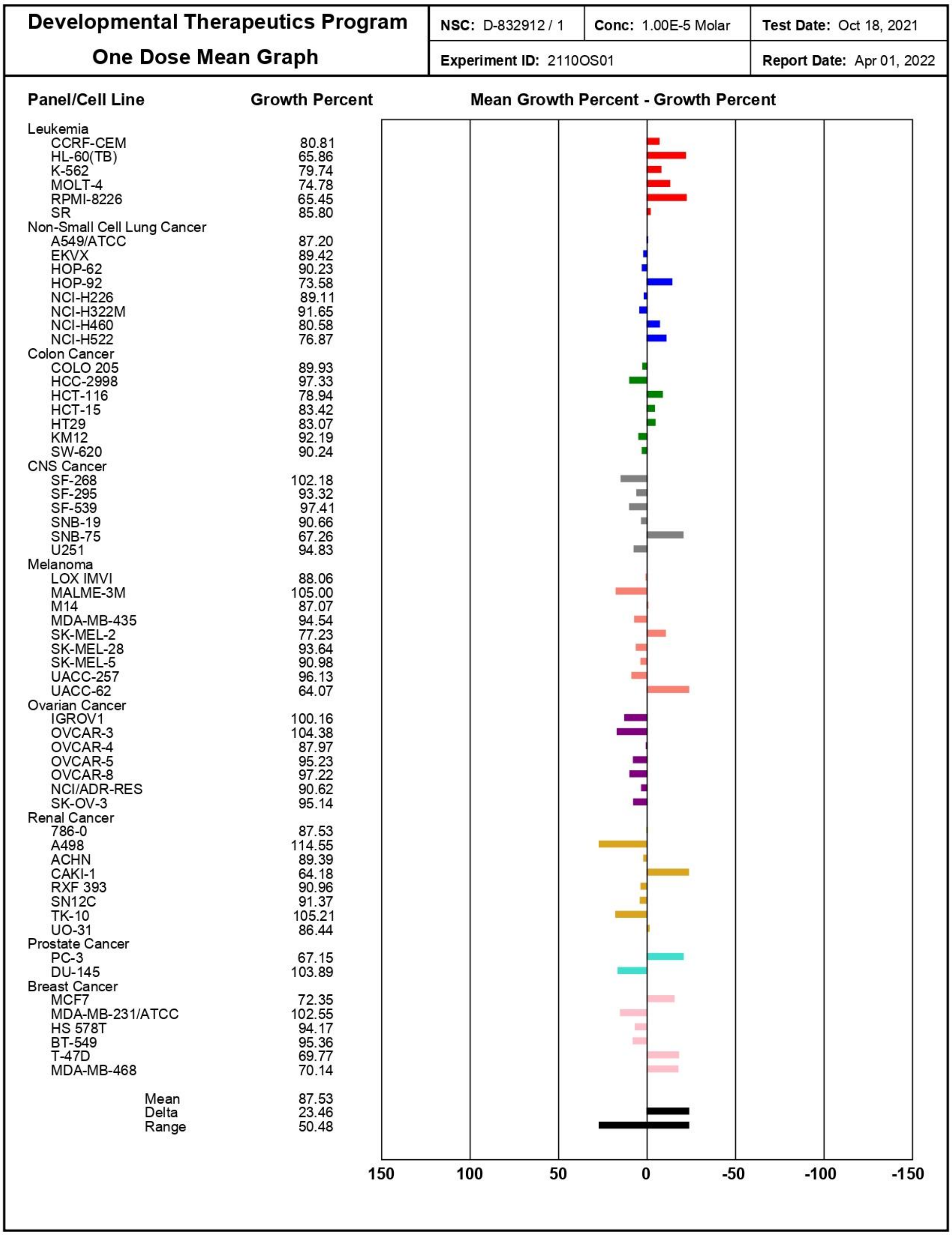

Figure S102. One dose mean graph for compound **6d** (NSC 832912) at 10 μM

Developmental Therapeutics Program	NSC: D-832917 / 1	Conc: 1.00E-5 Molar	Test Date: Oct 18, 2021
One Dose Mean Graph	Experiment ID: 2110OS01		Report Date: Apr 01, 2022

Panel/Cell Line	Growth Percent
Leukemia	
CCRF-CEM	39.53
HL-60(TB)	-11.17
K-562	50.63
MOLT-4	29.10
RPMI-8226	35.53
SR	65.74
Non-Small Cell Lung Cancer	
A549/ATCC	80.94
EKVX	91.04
HOP-62	90.81
HOP-92	71.59
NCI-H226	84.95
NCI-H322M	99.01
NCI-H460	53.50
NCI-H522	67.50
Colon Cancer	
COLO 205	62.72
HCC-2998	89.39
HCT-116	40.64
HCT-15	58.37
HT29	62.51
KM12	83.76
SW-620	46.03
CNS Cancer	
SF-268	99.32
SF-295	90.47
SF-539	103.55
SNB-19	86.67
SNB-75	87.20
U251	80.73
Melanoma	
LOX IMVI	88.78
MALME-3M	109.09
M14	82.96
MDA-MB-435	82.63
SK-MEL-2	85.04
SK-MEL-28	93.69
SK-MEL-5	77.41
UACC-257	84.12
UACC-62	71.75
Ovarian Cancer	
IGROV1	110.12
OVCAR-3	84.90
OVCAR-4	83.21
OVCAR-5	97.52
OVCAR-8	90.11
NCI/ADR-RES	95.75
SK-OV-3	91.93
Renal Cancer	
786-0	83.93
A498	108.35
ACHN	85.91
CAKI-1	69.82
RXF 393	86.53
SN12C	85.24
TK-10	110.27
UO-31	76.00
Prostate Cancer	
PC-3	32.44
DU-145	101.79
Breast Cancer	
MCF7	56.65
MDA-MB-231/ATCC	93.44
HS 578T	99.52
BT-549	93.87
T-47D	69.64
MDA-MB-468	77.05
Mean	77.96
Delta	89.13
Range	121.44

Mean Growth Percent - Growth Percent

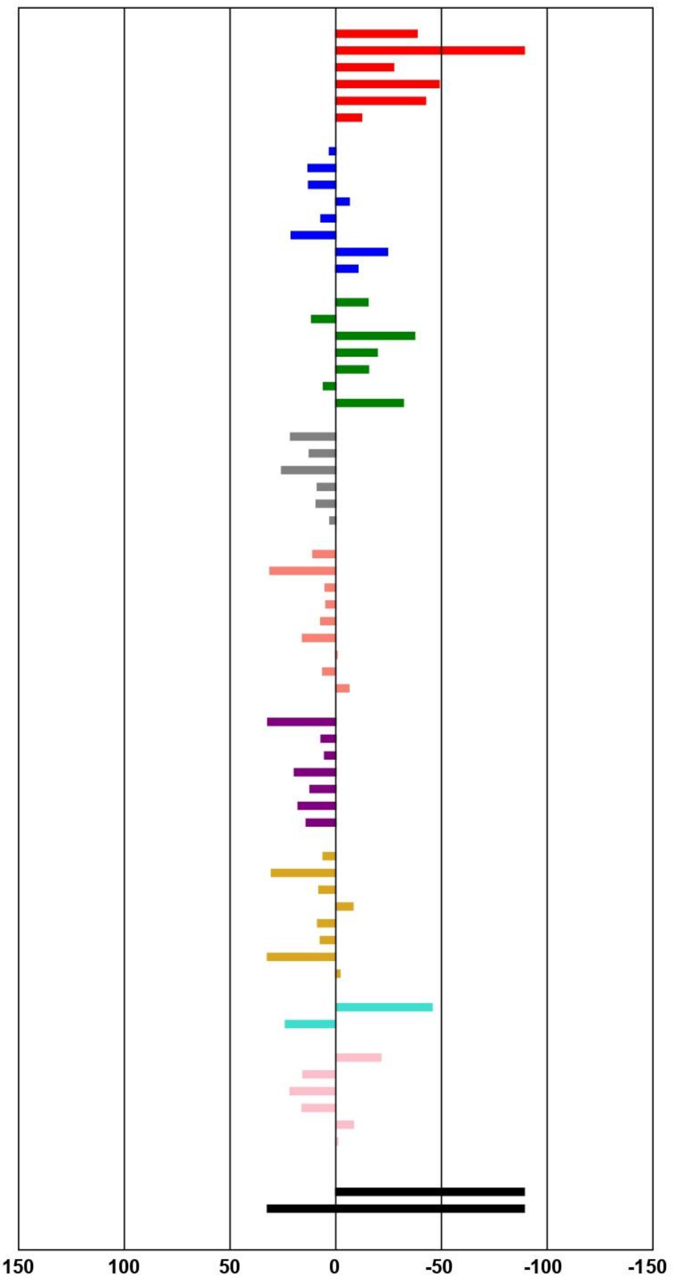

Figure S103. One dose mean graph for compound **6e** (NSC 832917) at 10 μM

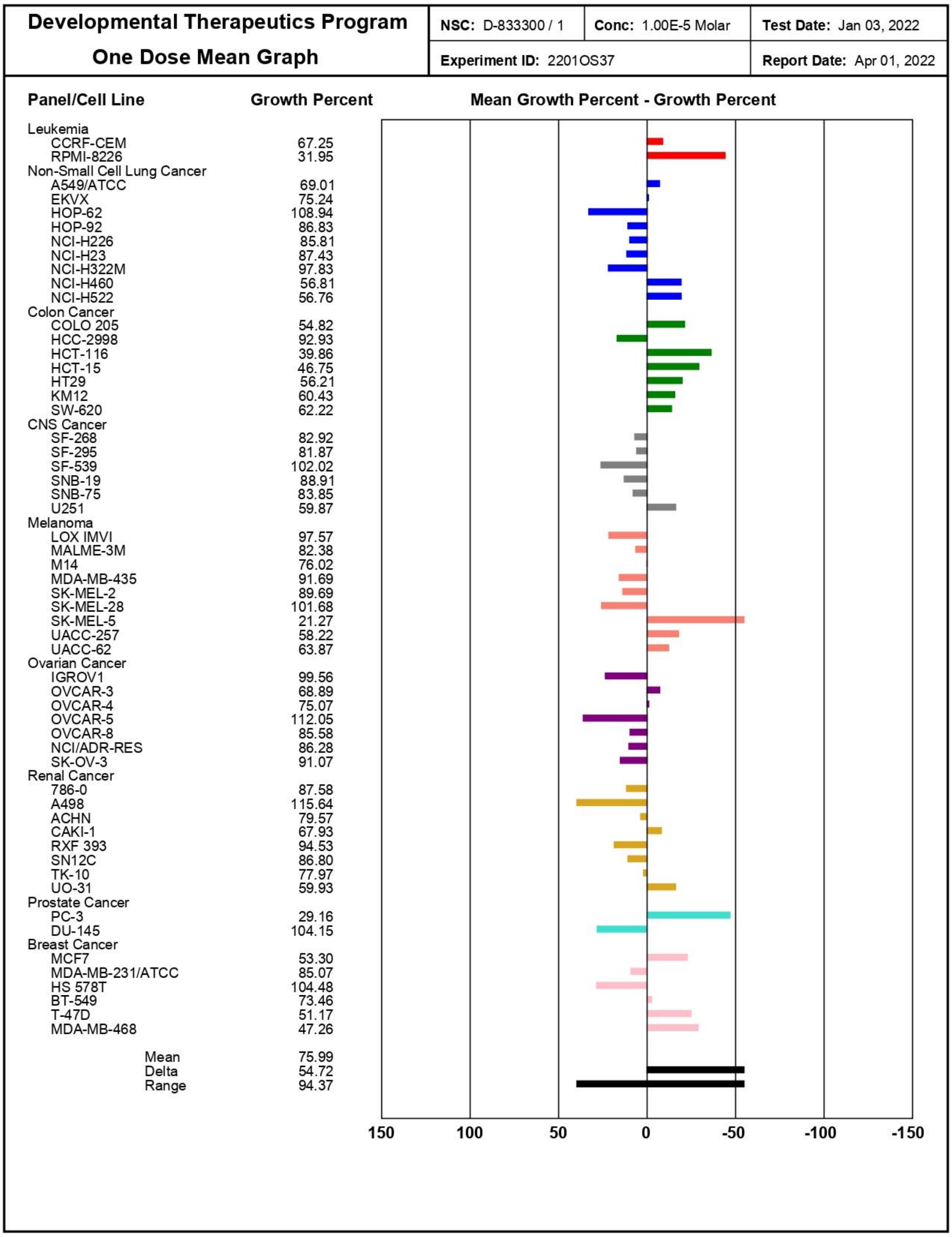

Figure S104. One dose mean graph for compound **6f** (NSC 833300) at 10 μM

Developmental Therapeutics Program	NSC: D-835925 / 1	Conc: 1.00E-5 Molar	Test Date: Mar 07, 2022
One Dose Mean Graph	Experiment ID: 2203OS82		Report Date: Apr 01, 2022

Panel/Cell Line	Growth Percent
**Leukemia**	
CCRF-CEM	100.07
HL-60(TB)	99.20
K-562	97.22
MOLT-4	104.63
RPMI-8226	100.49
SR	95.02
**Non-Small Cell Lung Cancer**	
A549/ATCC	103.28
EKVX	91.86
HOP-62	101.56
HOP-92	113.54
NCI-H226	100.79
NCI-H23	98.28
NCI-H322M	94.01
NCI-H460	115.26
NCI-H522	95.35
**Colon Cancer**	
COLO 205	127.93
HCC-2998	100.94
HCT-116	101.32
HCT-15	96.31
HT29	102.62
KM12	112.56
SW-620	107.18
**CNS Cancer**	
SF-268	113.99
SF-295	89.24
SF-539	92.59
SNB-19	92.20
SNB-75	93.98
U251	110.85
**Melanoma**	
LOX IMVI	100.52
MALME-3M	91.28
M14	110.96
MDA-MB-435	112.10
SK-MEL-2	109.28
SK-MEL-28	109.88
SK-MEL-5	97.16
UACC-257	106.67
UACC-62	80.39
**Ovarian Cancer**	
IGROV1	96.85
OVCAR-3	112.12
OVCAR-4	101.62
OVCAR-5	102.74
OVCAR-8	107.98
NCI/ADR-RES	96.55
SK-OV-3	98.15
**Renal Cancer**	
786-0	97.71
A498	98.66
ACHN	102.92
CAKI-1	97.22
SN12C	92.62
TK-10	106.32
UO-31	74.43
**Prostate Cancer**	
PC-3	89.17
DU-145	99.64
**Breast Cancer**	
MCF7	89.68
MDA-MB-231/ATCC	92.50
HS 578T	105.60
BT-549	104.94
T-47D	92.71
MDA-MB-468	88.68
Mean	100.33
Delta	25.90
Range	53.50

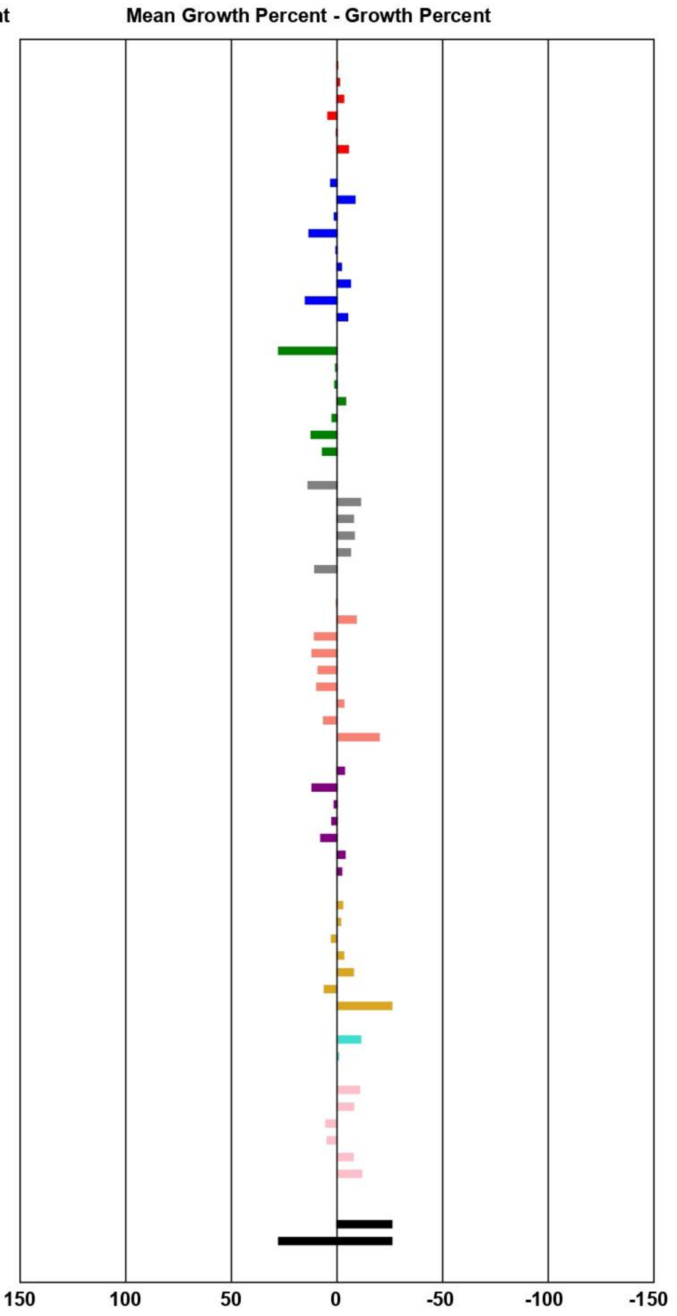

Figure S105. One dose mean graph for compound **6g** (NSC 835925) at 10 μM

Developmental Therapeutics Program<br>One Dose Mean Graph	NSC: D-835924 / 1	Conc: 1.00E-5 Molar	Test Date: Mar 07, 2022
	Experiment ID: 2203OS82		Report Date: Apr 01, 2022

Panel/Cell Line	Growth Percent
Leukemia	
CCRF-CEM	98.99
HL-60(TB)	98.90
K-562	98.38
MOLT-4	105.62
RPMI-8226	96.24
SR	92.09
Non-Small Cell Lung Cancer	
A549/ATCC	106.92
EKVX	92.24
HOP-62	106.23
HOP-92	117.40
NCI-H226	99.69
NCI-H23	108.77
NCI-H322M	93.37
NCI-H460	110.81
NCI-H522	97.57
Colon Cancer	
COLO 205	133.69
HCC-2998	106.20
HCT-116	102.53
HCT-15	98.41
HT29	115.71
KM12	108.40
SW-620	105.33
CNS Cancer	
SF-268	100.18
SF-295	90.99
SF-539	100.56
SNB-19	93.15
SNB-75	105.04
U251	104.26
Melanoma	
LOX IMVI	103.85
MALME-3M	93.43
M14	112.08
MDA-MB-435	111.14
SK-MEL-2	110.46
SK-MEL-28	104.21
SK-MEL-5	100.42
UACC-257	104.32
UACC-62	89.78
Ovarian Cancer	
IGROV1	98.63
OVCAR-3	117.97
OVCAR-4	107.98
OVCAR-5	104.24
OVCAR-8	110.43
NCI/ADR-RES	99.74
SK-OV-3	103.06
Renal Cancer	
786-0	99.65
A498	101.75
ACHN	103.06
CAKI-1	97.65
SN12C	99.62
TK-10	107.15
UO-31	78.66
Prostate Cancer	
PC-3	93.47
DU-145	106.87
Breast Cancer	
MCF7	93.53
MDA-MB-231/ATCC	95.47
HS 578T	102.21
BT-549	95.73
T-47D	97.79
MDA-MB-468	100.60
Mean	102.25
Delta	23.59
Range	55.03

Mean Growth Percent - Growth Percent

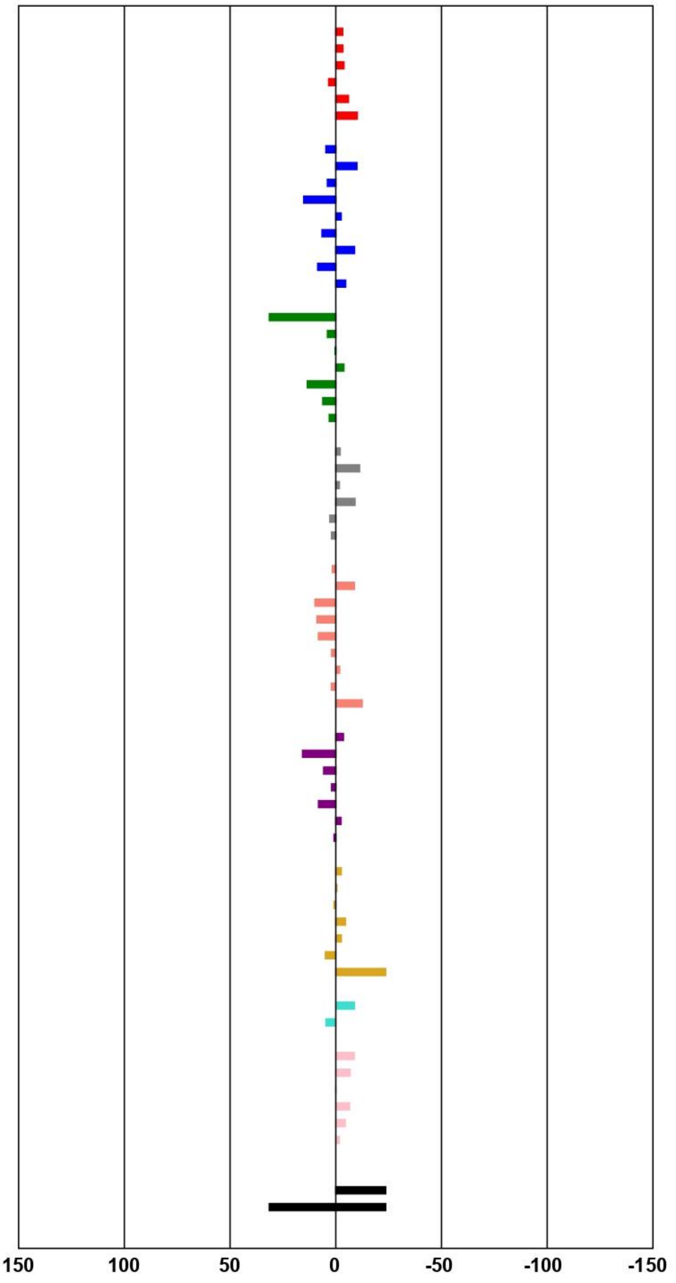

Figure S106. One dose mean graph for compound **6h** (NSC 835924) at 10 μM

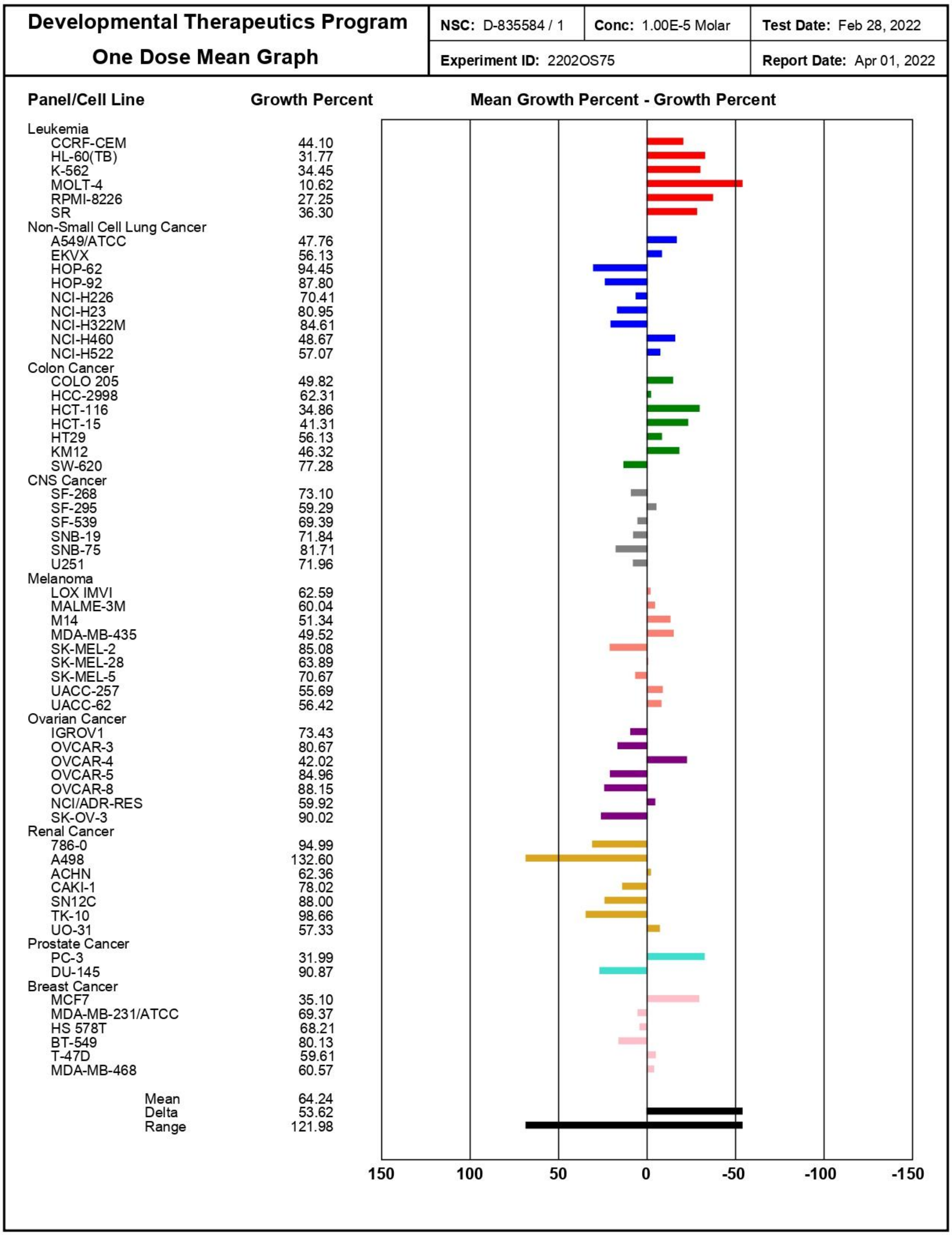

Figure S107. One dose mean graph for compound **6i** (NSC 835584) at 10 μM

Developmental Therapeutics Program	NSC: D-832913 / 1	Conc: 1.00E-5 Molar	Test Date: Oct 18, 2021
One Dose Mean Graph	Experiment ID: 2110OS01		Report Date: Apr 01, 2022

Panel/Cell Line	Growth Percent
Leukemia	
CCRF-CEM	107.67
HL-60(TB)	104.30
K-562	115.64
MOLT-4	111.99
RPMI-8226	105.58
SR	105.07
Non-Small Cell Lung Cancer	
A549/ATCC	103.87
EKVX	98.97
HOP-62	101.88
HOP-92	97.90
NCI-H226	97.34
NCI-H322M	99.72
NCI-H460	117.33
NCI-H522	97.31
Colon Cancer	
COLO 205	107.39
HCC-2998	101.69
HCT-116	103.78
HCT-15	103.21
HT29	111.71
KM12	111.42
SW-620	110.39
CNS Cancer	
SF-268	109.28
SF-295	108.38
SF-539	94.22
SNB-19	93.53
SNB-75	90.38
U251	101.58
Melanoma	
LOX IMVI	97.77
MALME-3M	105.67
M14	98.14
MDA-MB-435	101.71
SK-MEL-2	109.95
SK-MEL-28	101.79
SK-MEL-5	100.71
UACC-257	109.29
UACC-62	97.03
Ovarian Cancer	
IGROV1	107.53
OVCAR-3	122.98
OVCAR-4	104.89
OVCAR-5	98.90
OVCAR-8	110.85
NCI/ADR-RES	102.67
SK-OV-3	107.34
Renal Cancer	
786-0	97.83
A498	116.15
ACHN	104.85
CAKI-1	91.74
RXF 393	118.69
SN12C	102.07
TK-10	94.47
UO-31	91.22
Prostate Cancer	
PC-3	103.68
DU-145	116.86
Breast Cancer	
MCF7	99.13
MDA-MB-231/ATCC	108.97
HS 578T	99.86
BT-549	108.40
T-47D	98.47
MDA-MB-468	95.43
Mean	104.01
Delta	13.63
Range	32.60

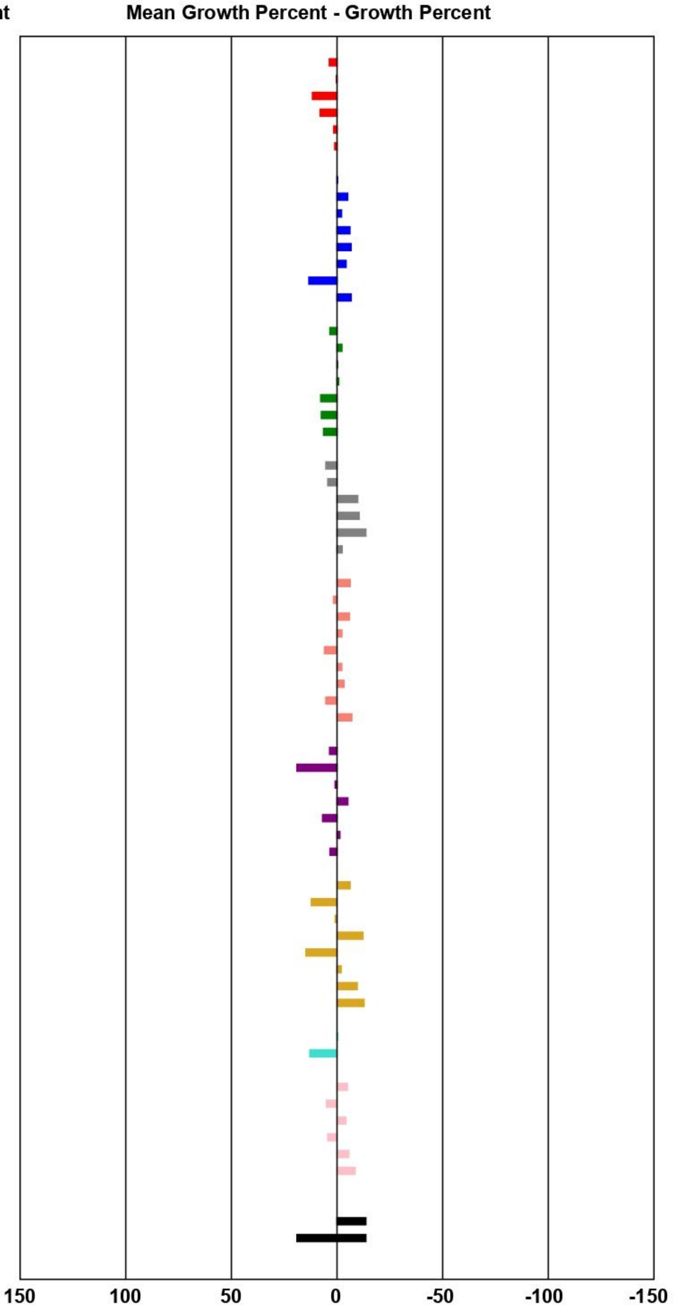

Figure S108. One dose mean graph for compound **8a** (NSC 832913) at 10 μM

Developmental Therapeutics Program One Dose Mean Graph	NSC: D-833301 / 1	Conc: 1.00E-5 Molar	Test Date: Jan 03, 2022
	Experiment ID: 2201OS37		Report Date: Apr 01, 2022

Panel/Cell Line	Growth Percent
Leukemia	
CCRF-CEM	73.57
RPMI-8226	67.57
Non-Small Cell Lung Cancer	
A549/ATCC	88.04
EKVX	74.99
HOP-62	111.51
HOP-92	79.24
NCI-H226	94.81
NCI-H23	81.75
NCI-H322M	93.97
NCI-H460	105.76
NCI-H522	59.76
Colon Cancer	
COLO 205	86.54
HCC-2998	99.34
HCT-116	66.16
HCT-15	65.65
HT29	60.50
KM12	90.91
SW-620	100.92
CNS Cancer	
SF-268	92.12
SF-295	87.82
SF-539	100.01
SNB-19	77.53
SNB-75	102.24
U251	79.13
Melanoma	
LOX IMVI	86.18
MALME-3M	98.24
M14	85.60
MDA-MB-435	88.84
SK-MEL-2	80.04
SK-MEL-28	93.52
SK-MEL-5	71.23
UACC-257	88.84
UACC-62	59.99
Ovarian Cancer	
IGROV1	90.04
OVCAR-3	106.20
OVCAR-4	83.61
OVCAR-5	97.09
OVCAR-8	87.14
NCI/ADR-RES	87.75
SK-OV-3	101.54
Renal Cancer	
786-0	79.60
A498	110.18
ACHN	94.70
CAKI-1	74.17
RXF 393	82.65
SN12C	91.44
TK-10	96.12
UO-31	70.08
Prostate Cancer	
PC-3	65.02
DU-145	101.75
Breast Cancer	
MCF7	75.46
MDA-MB-231/ATCC	81.31
HS 578T	119.48
BT-549	86.99
T-47D	72.58
MDA-MB-468	82.56
Mean	86.25
Delta	26.49
Range	59.72

Mean Growth Percent - Growth Percent

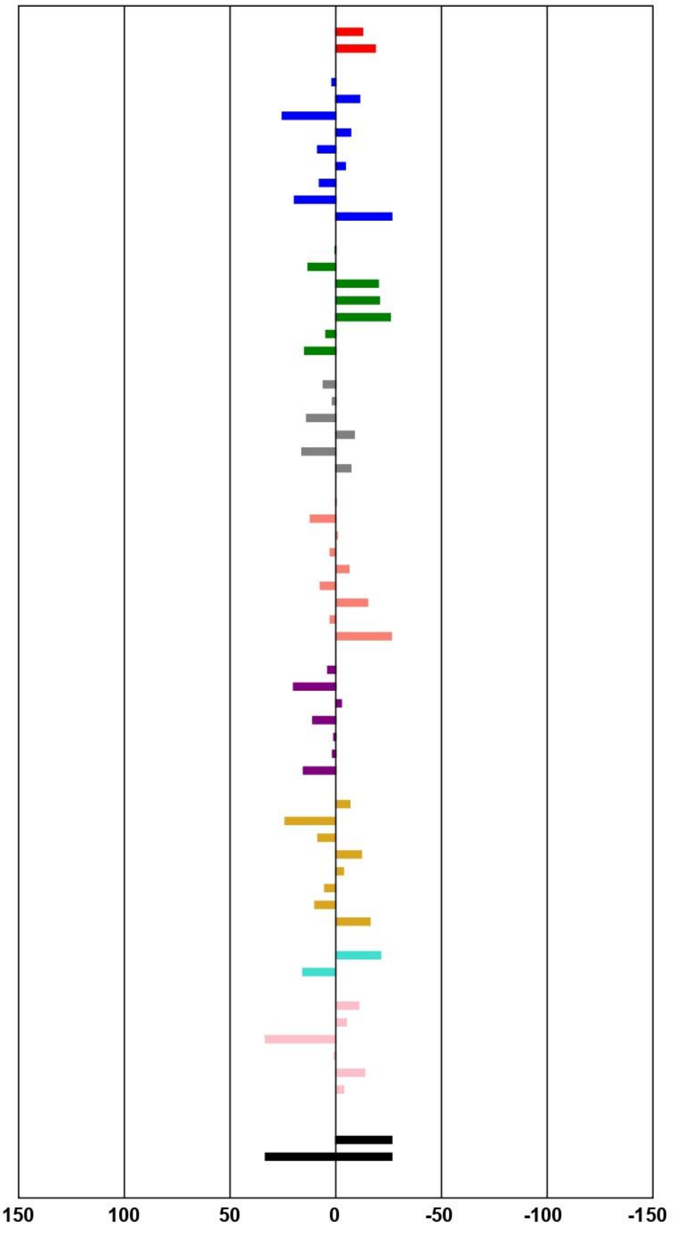

Figure S109. One dose mean graph for compound **8b** (NSC 833301) at 10 μM

Developmental Therapeutics Program	NSC: D-835582 / 1	Conc: 1.00E-5 Molar	Test Date: Feb 28, 2022
One Dose Mean Graph	Experiment ID: 2202OS75		Report Date: Apr 01, 2022

Panel/Cell Line	Growth Percent
Leukemia	
CCRF-CEM	15.39
HL-60(TB)	6.03
K-562	18.27
MOLT-4	1.54
RPMI-8226	7.68
SR	13.08
Non-Small Cell Lung Cancer	
A549/ATCC	33.86
EKVX	27.69
HOP-62	80.68
HOP-92	69.48
NCI-H226	70.38
NCI-H23	48.85
NCI-H322M	64.93
NCI-H460	12.98
NCI-H522	38.39
Colon Cancer	
COLO 205	-17.66
HCC-2998	42.92
HCT-116	18.72
HCT-15	14.46
HT29	34.80
KM12	17.94
SW-620	27.92
CNS Cancer	
SF-268	57.96
SF-295	21.28
SF-539	36.24
SNB-19	34.92
SNB-75	73.05
U251	41.97
Melanoma	
LOX IMVI	41.45
MALME-3M	15.86
M14	28.23
MDA-MB-435	21.99
SK-MEL-2	30.20
SK-MEL-28	44.46
SK-MEL-5	27.65
UACC-257	28.02
UACC-62	22.19
Ovarian Cancer	
IGROV1	59.46
OVCAR-3	51.59
OVCAR-4	29.35
OVCAR-5	57.63
OVCAR-8	61.12
NCI/ADR-RES	25.62
SK-OV-3	43.69
Renal Cancer	
786-0	67.34
A498	67.16
ACHN	22.26
CAKI-1	50.22
SN12C	64.19
TK-10	80.44
UO-31	29.04
Prostate Cancer	
PC-3	21.63
DU-145	58.28
Breast Cancer	
MCF7	17.42
MDA-MB-231/ATCC	52.98
HS 578T	71.93
BT-549	43.20
T-47D	29.51
MDA-MB-468	33.16
Mean	37.47
Delta	55.13
Range	98.34

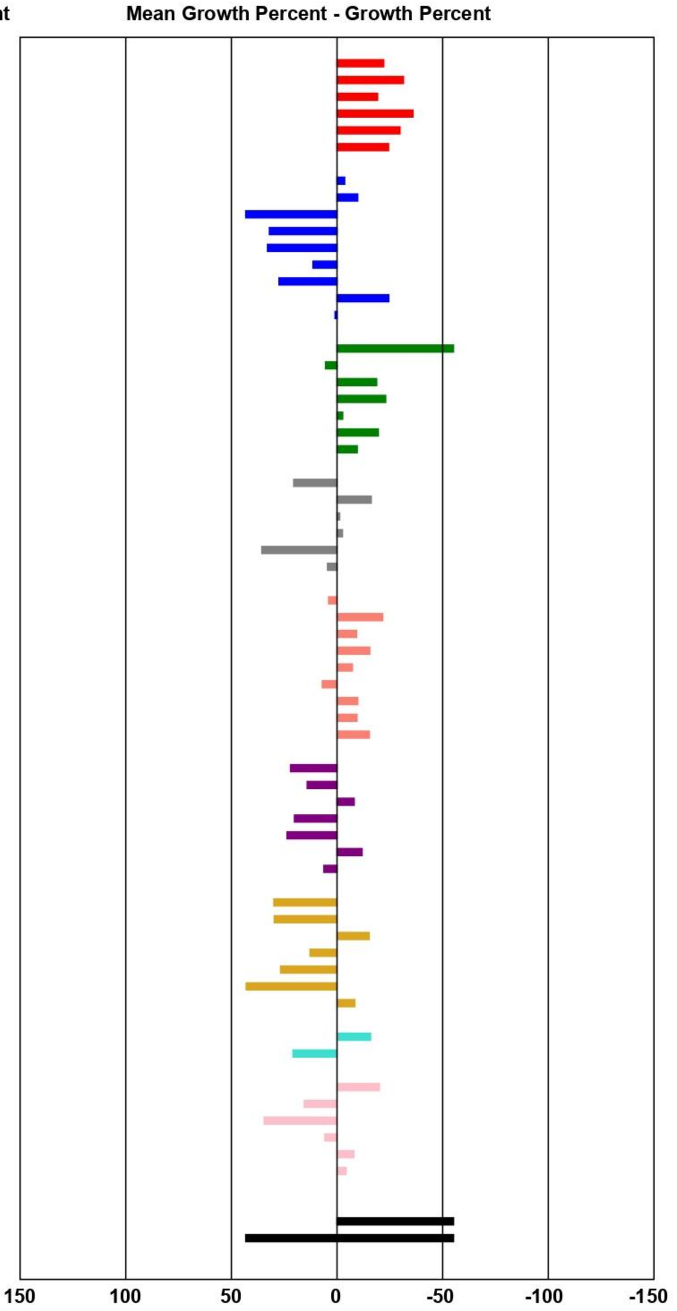

Figure S110. One dose mean graph for compound **8c** (NSC 835582) at 10 μM

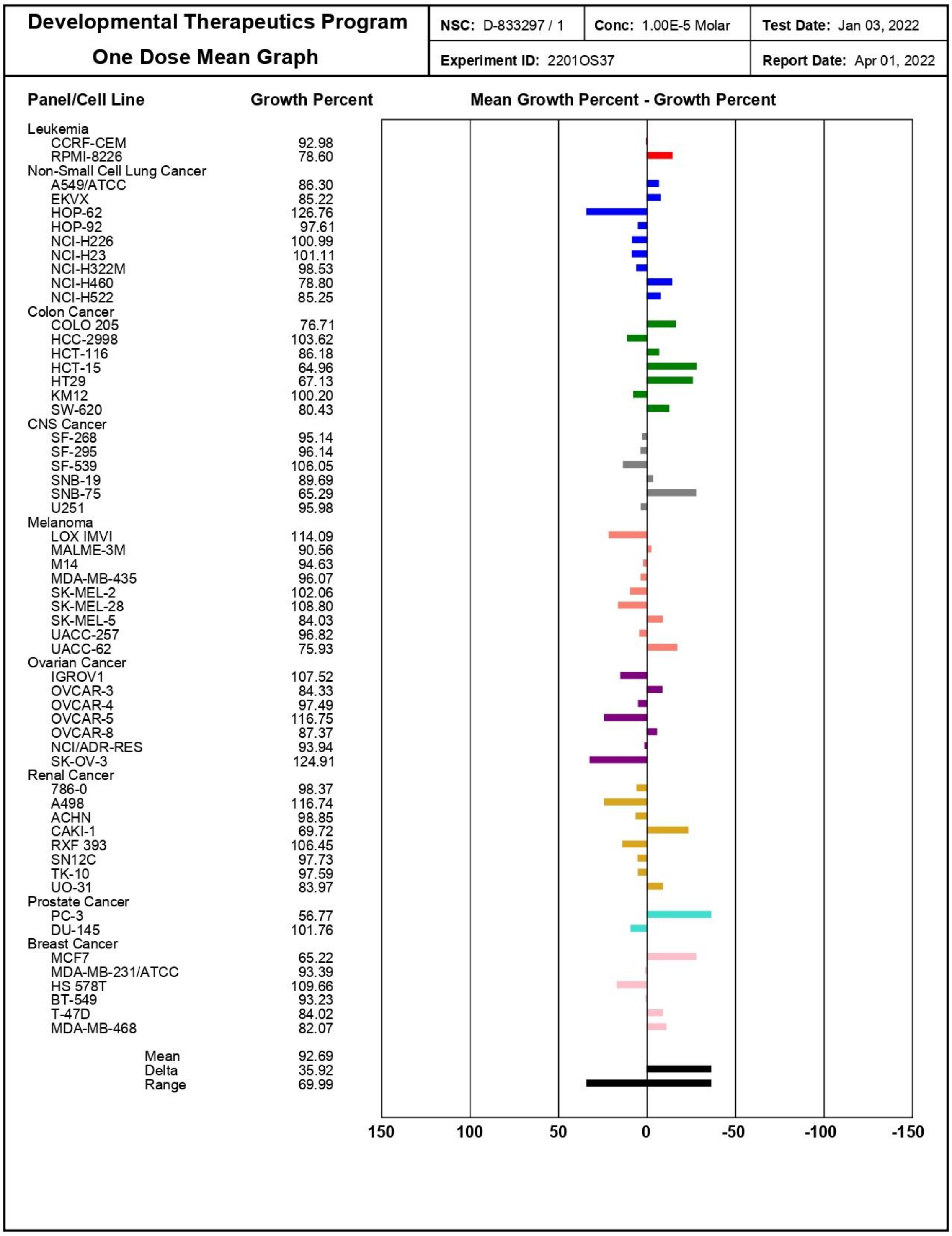

Figure S111. One dose mean graph for compound **8d** (NSC 833297) at 10 μM

Developmental Therapeutics Program	NSC: D-834379 / 1	Conc: 1.00E-5 Molar	Test Date: Feb 14, 2022
One Dose Mean Graph	Experiment ID: 2202OS68		Report Date: Apr 01, 2022

Panel/Cell Line	Growth Percent
Leukemia	
CCRF-CEM	38.98
HL-60(TB)	37.67
K-562	37.11
MOLT-4	11.08
RPMI-8226	10.10
SR	48.47
Non-Small Cell Lung Cancer	
A549/ATCC	38.08
EKVX	67.08
HOP-62	94.08
HOP-92	90.17
NCI-H226	106.53
NCI-H23	80.58
NCI-H322M	98.71
NCI-H460	40.78
NCI-H522	80.81
Colon Cancer	
COLO 205	47.12
HCC-2998	88.71
HCT-116	55.00
HCT-15	42.36
HT29	53.57
KM12	61.76
SW-620	67.15
CNS Cancer	
SF-268	77.63
SF-295	67.23
SF-539	74.14
SNB-19	68.90
SNB-75	72.87
U251	69.04
Melanoma	
LOX IMVI	78.58
MALME-3M	60.68
M14	51.28
MDA-MB-435	46.15
SK-MEL-2	97.49
SK-MEL-28	74.37
SK-MEL-5	53.99
UACC-257	43.84
UACC-62	72.04
Ovarian Cancer	
IGROV1	88.38
OVCAR-3	84.00
OVCAR-4	46.07
OVCAR-5	125.82
OVCAR-8	70.42
NCI/ADR-RES	68.41
SK-OV-3	91.45
Renal Cancer	
786-0	76.57
A498	141.45
ACHN	67.87
CAKI-1	80.59
RXF 393	64.99
SN12C	111.71
TK-10	89.33
UO-31	66.32
Prostate Cancer	
PC-3	42.19
DU-145	88.46
Breast Cancer	
MCF7	44.00
MDA-MB-231/ATCC	76.40
HS 578T	56.00
BT-549	79.45
T-47D	57.84
MDA-MB-468	66.97
Mean	68.15
Delta	58.05
Range	131.35

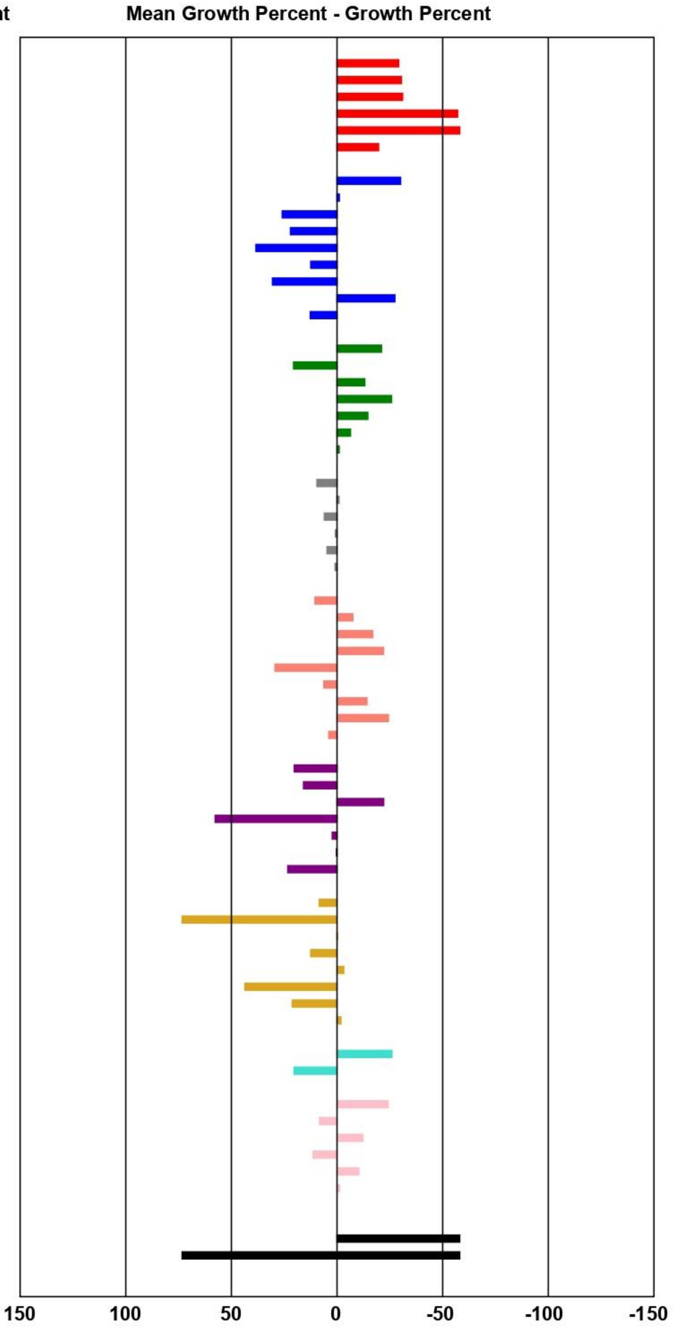

Figure S112. One dose mean graph for compound **8e** (NSC 834379) at 10 μM

Developmental Therapeutics Program	NSC: D-832914 / 1	Conc: 1.00E-5 Molar	Test Date: Oct 18, 2021
One Dose Mean Graph	Experiment ID: 2110OS01		Report Date: Apr 01, 2022

Panel/Cell Line	Growth Percent
Leukemia	
CCRF-CEM	94.21
HL-60(TB)	98.66
K-562	93.71
MOLT-4	85.54
RPMI-8226	84.51
SR	82.30
Non-Small Cell Lung Cancer	
A549/ATCC	93.26
EKVX	93.77
HOP-62	92.71
HOP-92	90.35
NCI-H226	87.71
NCI-H322M	98.35
NCI-H460	102.74
NCI-H522	83.31
Colon Cancer	
COLO 205	96.68
HCC-2998	100.79
HCT-116	85.63
HCT-15	90.88
HT29	101.56
KM12	97.48
SW-620	97.39
CNS Cancer	
SF-268	108.59
SF-295	99.05
SF-539	96.46
SNB-19	86.25
SNB-75	90.95
U251	94.21
Melanoma	
LOX IMVI	91.81
MALME-3M	113.92
M14	96.56
MDA-MB-435	98.18
SK-MEL-2	83.81
SK-MEL-28	104.31
SK-MEL-5	95.91
UACC-257	103.76
UACC-62	77.17
Ovarian Cancer	
IGROV1	104.29
OVCAR-3	116.72
OVCAR-4	99.46
OVCAR-5	98.60
OVCAR-8	97.80
NCI/ADR-RES	96.45
SK-OV-3	98.48
Renal Cancer	
786-0	89.13
A498	120.55
ACHN	97.30
CAKI-1	78.15
RXF 393	98.10
SN12C	95.67
TK-10	106.05
UO-31	92.72
Prostate Cancer	
PC-3	81.56
DU-145	115.70
Breast Cancer	
MCF7	87.43
MDA-MB-231/ATCC	100.84
HS 578T	88.07
BT-549	102.52
T-47D	89.06
MDA-MB-468	91.82
Mean	95.58
Delta	18.41
Range	43.38

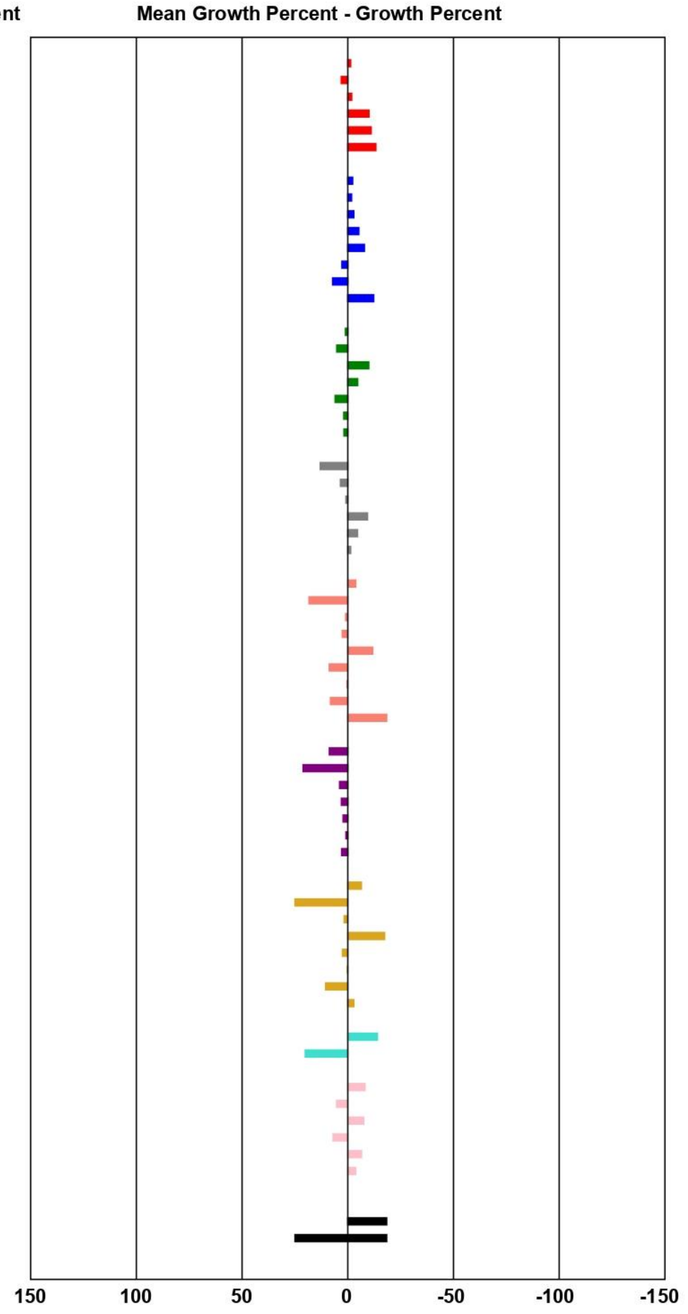

Figure S113. One dose mean graph for compound **8f** (NSC 832914) at 10 μM

Developmental Therapeutics Program	NSC: D-832918 / 1	Conc: 1.00E-5 Molar	Test Date: Oct 18, 2021
One Dose Mean Graph	Experiment ID: 2110OS01		Report Date: Apr 01, 2022

Panel/Cell Line	Growth Percent
Leukemia	
CCRF-CEM	107.89
HL-60(TB)	97.84
K-562	109.41
MOLT-4	105.11
RPMI-8226	109.35
SR	90.14
Non-Small Cell Lung Cancer	
A549/ATCC	110.64
EKVX	103.75
HOP-62	110.78
HOP-92	127.26
NCI-H226	107.83
NCI-H322M	105.70
NCI-H460	111.34
NCI-H522	91.55
Colon Cancer	
COLO 205	104.23
HCC-2998	102.96
HCT-116	106.09
HCT-15	97.93
HT29	111.50
KM12	100.85
SW-620	103.22
CNS Cancer	
SF-268	106.00
SF-295	109.99
SF-539	106.38
SNB-19	101.35
SNB-75	87.21
U251	104.51
Melanoma	
LOX IMVI	93.30
MALME-3M	110.58
M14	98.45
MDA-MB-435	100.30
SK-MEL-2	101.17
SK-MEL-28	108.44
SK-MEL-5	101.57
UACC-257	121.38
UACC-62	100.67
Ovarian Cancer	
IGROV1	116.98
OVCAR-3	112.01
OVCAR-4	112.70
OVCAR-5	108.64
OVCAR-8	104.11
NCI/ADR-RES	107.29
SK-OV-3	111.17
Renal Cancer	
786-0	99.88
A498	118.98
ACHN	103.16
CAKI-1	96.57
RXF 393	113.65
SN12C	99.60
TK-10	101.88
UO-31	96.51
Prostate Cancer	
PC-3	99.83
DU-145	120.32
Breast Cancer	
MCF7	96.48
MDA-MB-231/ATCC	115.94
HS 578T	104.35
BT-549	104.55
T-47D	100.29
MDA-MB-468	98.76
Mean	105.26
Delta	18.05
Range	40.05

Mean Growth Percent - Growth Percent

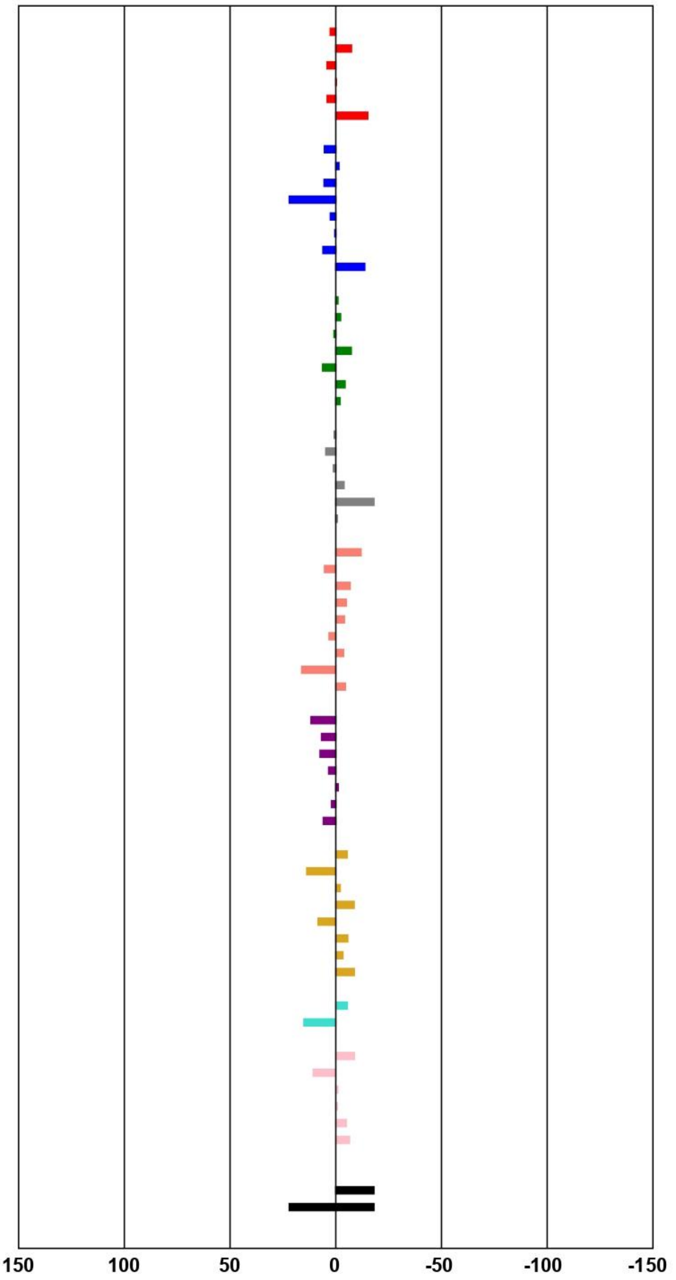

Figure S114. One dose mean graph for compound **8g** (NSC 832918) at 10 μM

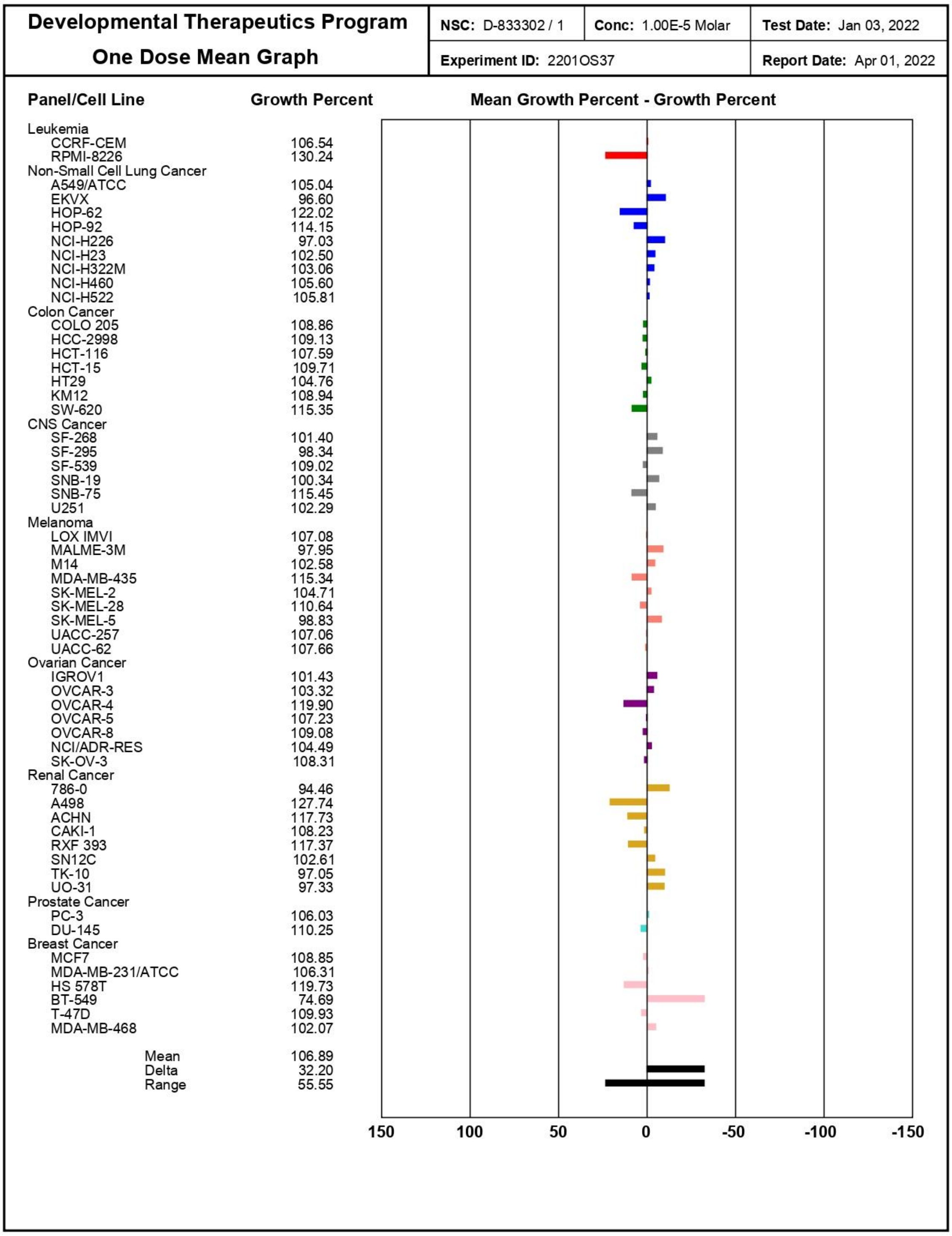

Figure S115. One dose mean graph for compound **8h** (NSC 833302) at 10 μM

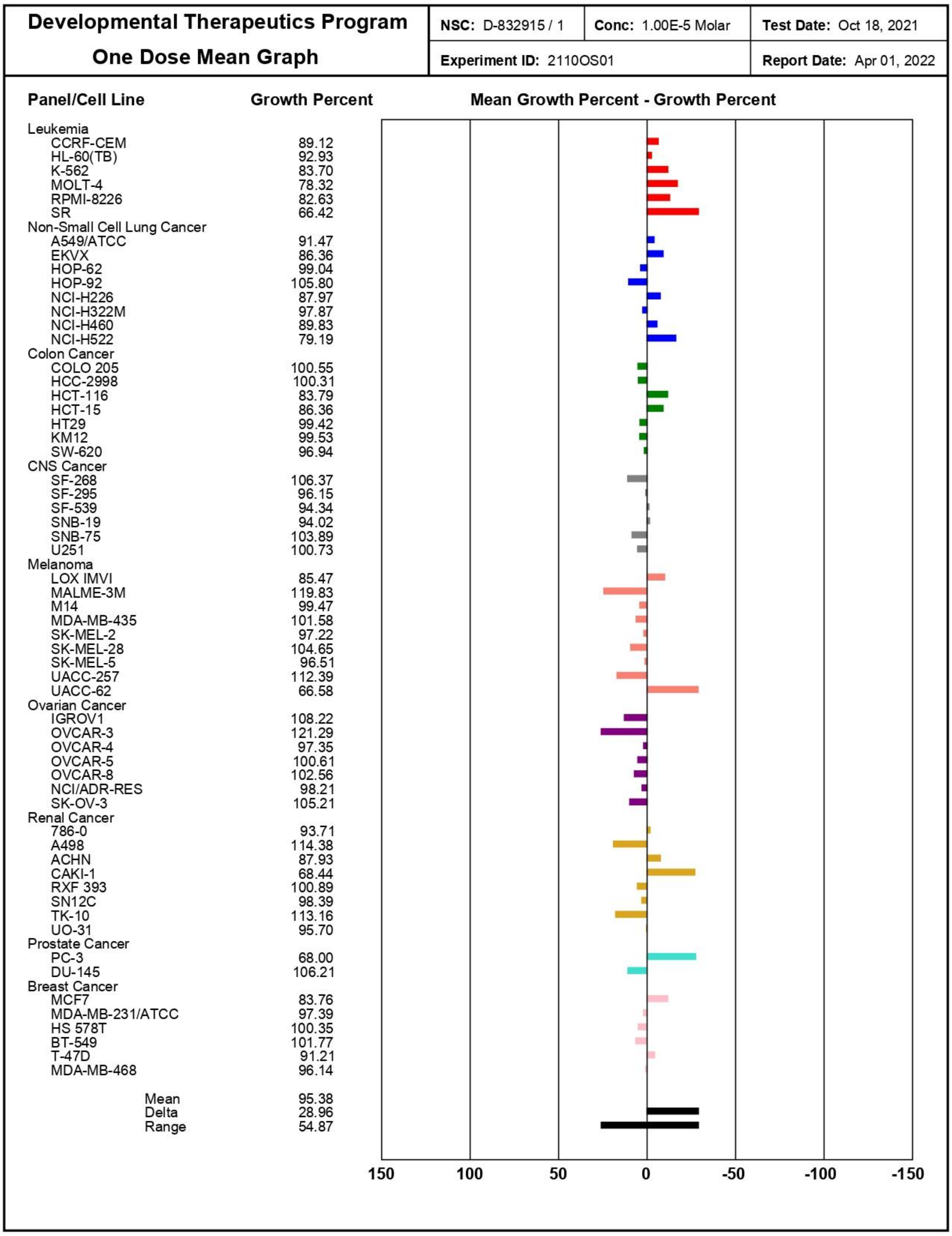

Figure S116. One dose mean graph for compound **8i** (NSC 832915) at 10 μM

Developmental Therapeutics Program | **NSC:** D-832919 / 1 | **Conc:** 1.00E-5 Molar | **Test Date:** Oct 18, 2021

One Dose Mean Graph | **Experiment ID:** 2110OS01 | **Report Date:** Apr 01, 2022

Panel/Cell Line	Growth Percent
Leukemia	
CCRF-CEM	105.25
HL-60(TB)	100.00
K-562	94.95
MOLT-4	102.56
RPMI-8226	99.12
SR	93.89
Non-Small Cell Lung Cancer	
A549/ATCC	107.51
EKVX	99.97
HOP-62	104.74
HOP-92	118.61
NCI-H226	99.85
NCI-H322M	96.39
NCI-H460	102.54
NCI-H522	95.05
Colon Cancer	
COLO 205	92.28
HCC-2998	101.84
HCT-116	100.23
HCT-15	99.72
HT29	102.48
KM12	100.93
SW-620	93.39
CNS Cancer	
SF-268	104.56
SF-295	106.65
SF-539	99.97
SNB-19	99.00
SNB-75	94.66
U251	102.88
Melanoma	
LOX IMVI	98.30
MALME-3M	110.03
M14	96.04
MDA-MB-435	101.35
SK-MEL-2	103.38
SK-MEL-28	99.85
SK-MEL-5	100.36
UACC-257	105.12
UACC-62	86.69
Ovarian Cancer	
IGROV1	108.49
OVCAR-3	111.02
OVCAR-4	100.69
OVCAR-5	100.92
OVCAR-8	96.90
NCI/ADR-RES	99.74
SK-OV-3	104.16
Renal Cancer	
786-0	99.42
A498	96.43
ACHN	104.62
CAKI-1	93.33
RXF 393	110.90
SN12C	99.04
TK-10	111.21
UO-31	90.68
Prostate Cancer	
PC-3	100.56
DU-145	110.21
Breast Cancer	
MCF7	95.98
MDA-MB-231/ATCC	106.05
HS 578T	102.53
BT-549	104.43
T-47D	98.73
MDA-MB-468	98.93
Mean	101.10
Delta	14.41
Range	31.92

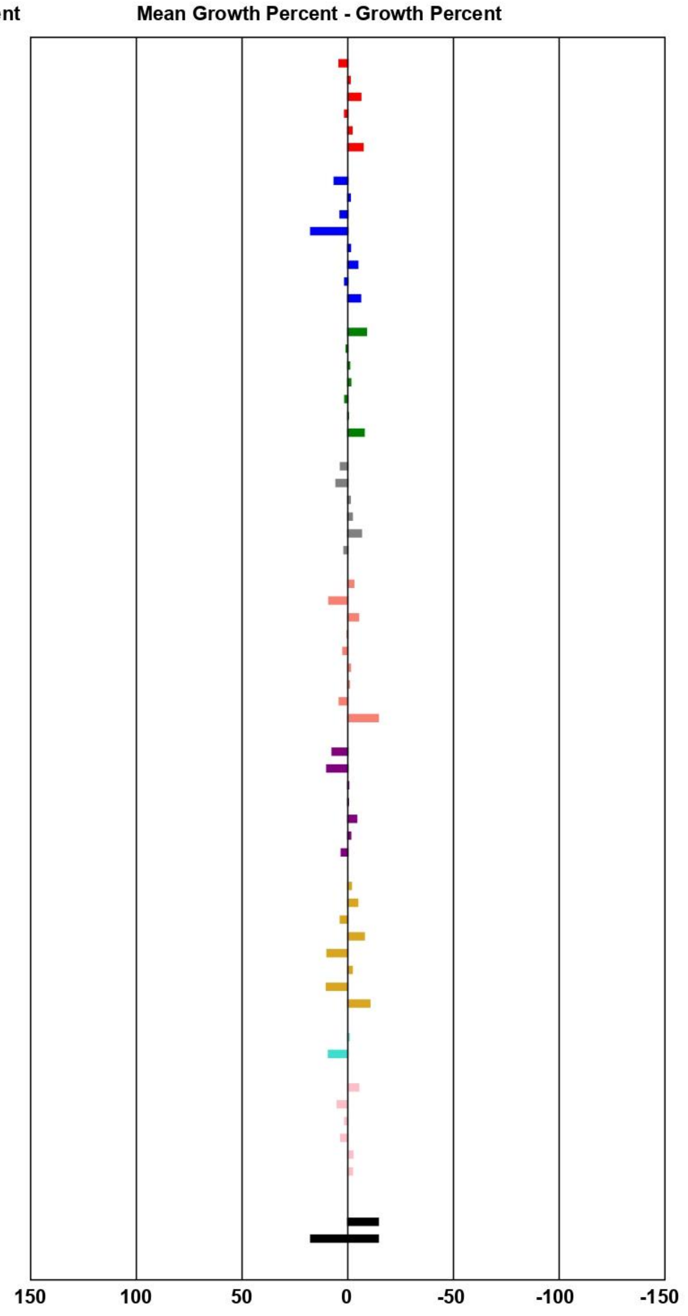

Figure S117. One dose mean graph for compound **8j** (NSC 832919) at 10 μM

Developmental Therapeutics Program	NSC: D-835585 / 1	Conc: 1.00E-5 Molar	Test Date: Feb 28, 2022
One Dose Mean Graph	Experiment ID: 2202OS75		Report Date: Apr 01, 2022

Panel/Cell Line	Growth Percent
Leukemia	
CCRF-CEM	91.72
HL-60(TB)	78.09
K-562	74.42
MOLT-4	72.87
RPMI-8226	57.39
SR	67.91
Non-Small Cell Lung Cancer	
A549/ATCC	88.93
EKVX	85.35
HOP-62	101.99
HOP-92	122.70
NCI-H226	84.61
NCI-H23	90.10
NCI-H322M	97.65
NCI-H460	90.53
NCI-H522	72.59
Colon Cancer	
COLO 205	101.67
HCC-2998	93.93
HCT-116	66.12
HCT-15	81.94
HT29	89.69
KM12	87.56
SW-620	113.23
CNS Cancer	
SF-268	99.70
SF-295	89.89
SF-539	91.93
SNB-19	92.81
SNB-75	97.31
U251	102.82
Melanoma	
LOX IMVI	79.03
MALME-3M	102.26
M14	89.50
MDA-MB-435	90.86
SK-MEL-2	80.89
SK-MEL-28	97.88
SK-MEL-5	90.08
UACC-257	94.97
UACC-62	71.09
Ovarian Cancer	
IGROV1	102.01
OVCAR-3	99.77
OVCAR-4	91.50
OVCAR-5	95.85
OVCAR-8	88.30
NCI/ADR-RES	84.38
SK-OV-3	102.77
Renal Cancer	
786-0	90.41
A498	136.06
ACHN	88.14
CAKI-1	88.76
SN12C	95.08
TK-10	103.92
UO-31	68.14
Prostate Cancer	
PC-3	73.74
DU-145	112.51
Breast Cancer	
MCF7	81.18
MDA-MB-231/ATCC	95.53
HS 578T	97.32
BT-549	110.53
T-47D	73.41
MDA-MB-468	82.28
Mean	90.57
Delta	33.18
Range	78.67

Mean Growth Percent - Growth Percent

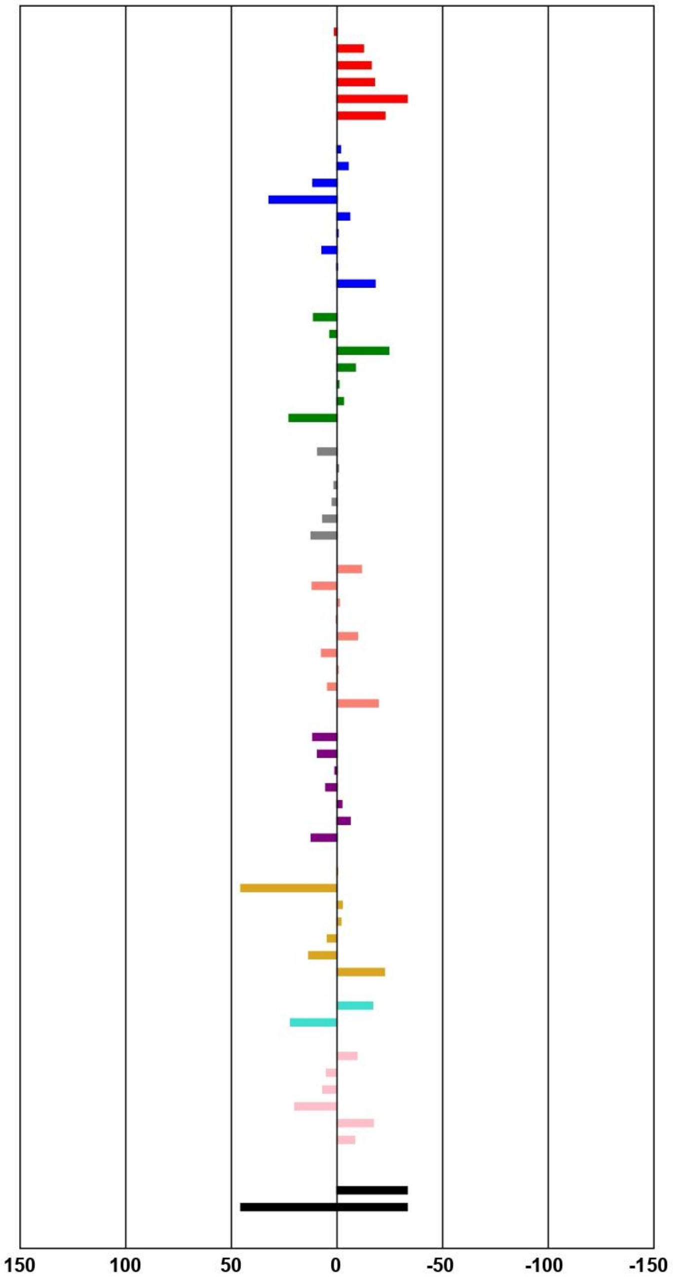

Figure S118. One dose mean graph for compound **8k** (NSC 835585) at 10 μM

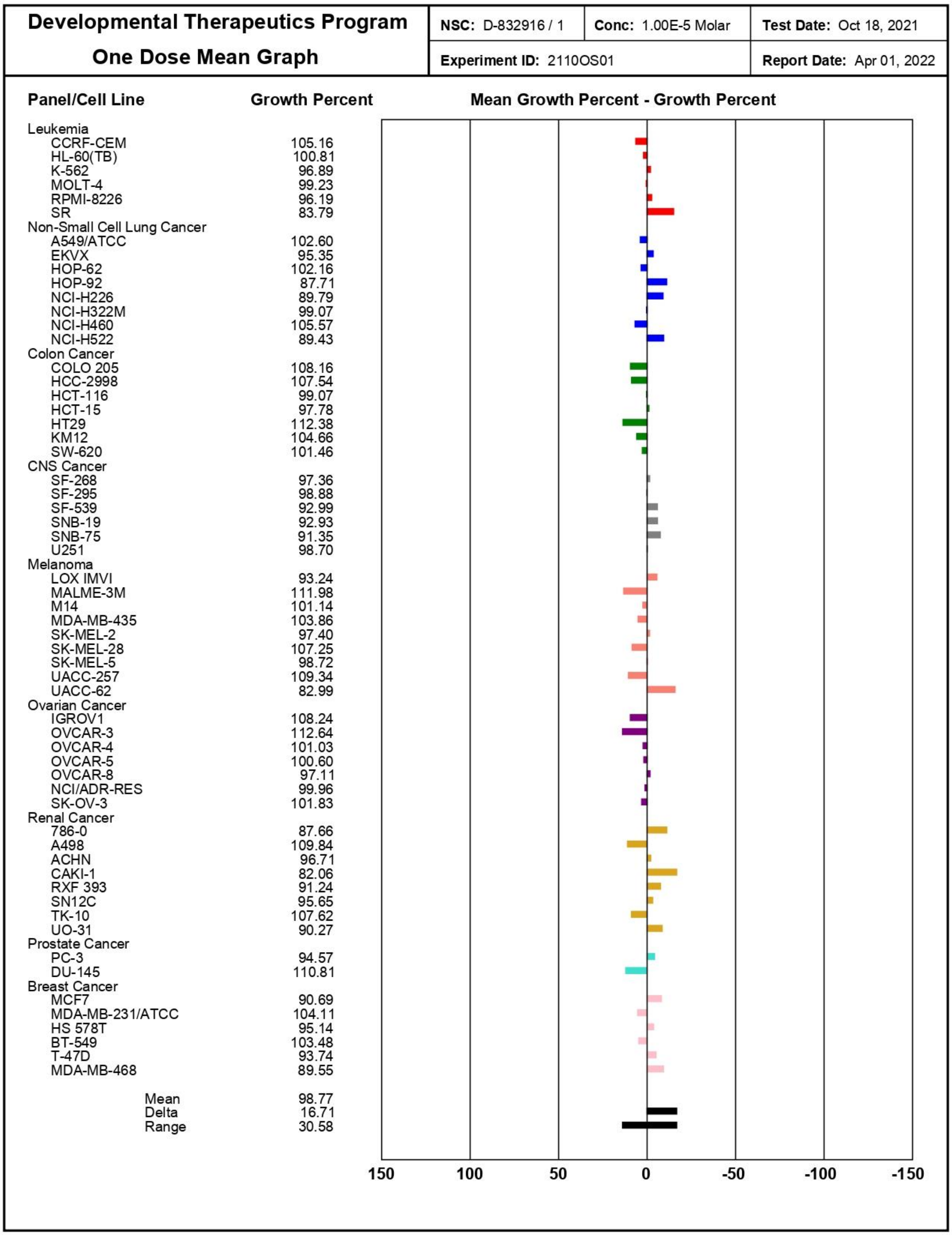

Figure S119. One dose mean graph for compound **8l** (NSC 832916) at 10 μM

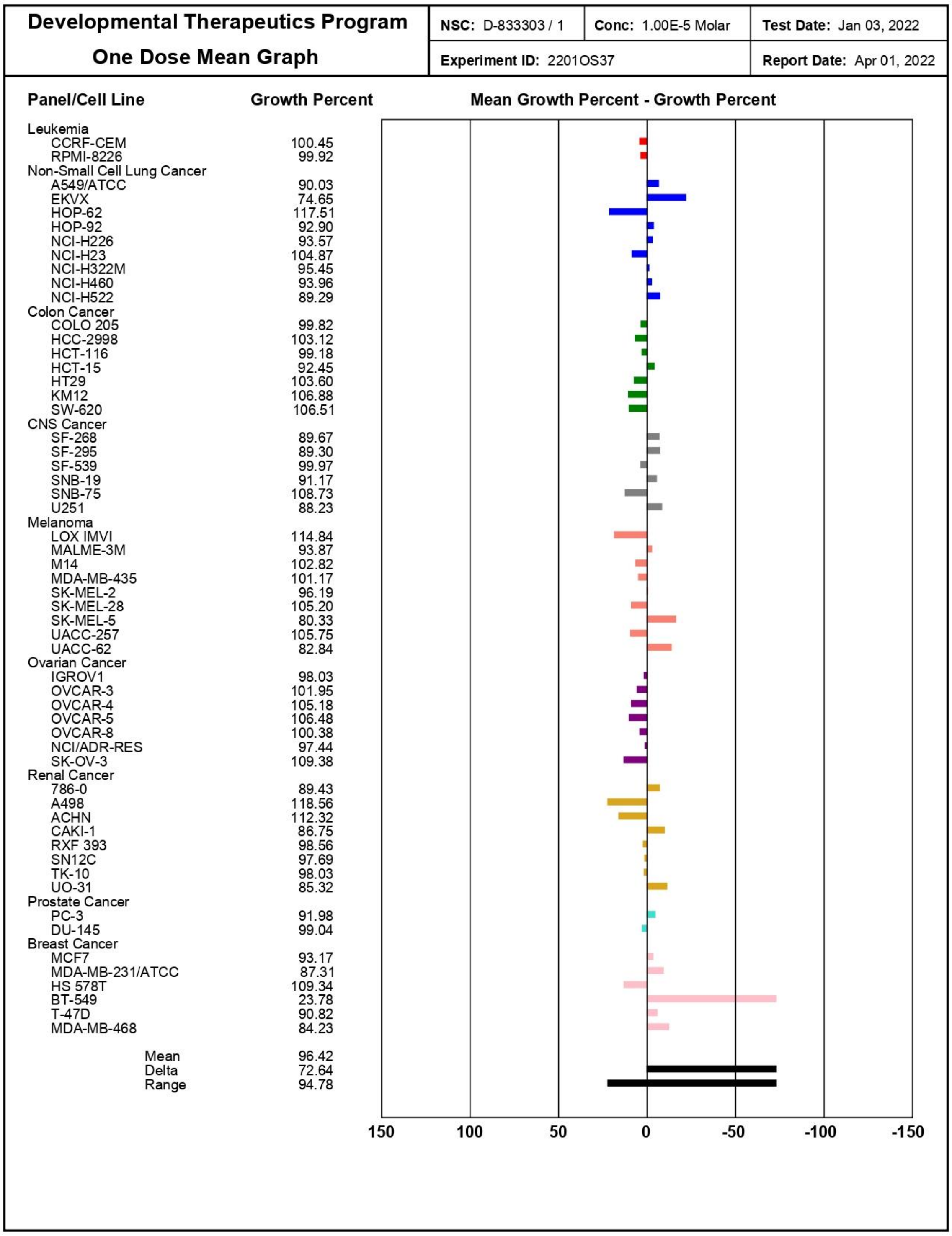

Figure S120. One dose mean graph for compound **8m** (NSC 833303) at 10 μM

Developmental Therapeutics Program	NSC: D-838214 / 1	Conc: 1.00E-5 Molar	Test Date: May 23, 2022
One Dose Mean Graph	Experiment ID: 2205OS30		Report Date: Jun 23, 2022

Panel/Cell Line	Growth Percent
Leukemia	
CCRF-CEM	54.76
HL-60(TB)	46.21
K-562	59.52
MOLT-4	39.51
RPMI-8226	50.71
SR	56.29
Non-Small Cell Lung Cancer	
A549/ATCC	60.62
EKVX	57.53
HOP-62	40.80
HOP-92	-0.83
NCI-H226	40.32
NCI-H23	69.70
NCI-H322M	77.85
NCI-H460	60.96
NCI-H522	74.93
Colon Cancer	
COLO 205	80.59
HCC-2998	86.93
HCT-116	40.11
HCT-15	65.82
HT29	72.69
KM12	76.56
CNS Cancer	
SF-268	65.99
SF-295	53.52
SF-539	32.61
SNB-19	41.67
SNB-75	56.82
U251	55.20
Melanoma	
MALME-3M	77.61
M14	81.01
MDA-MB-435	87.82
SK-MEL-2	86.53
SK-MEL-28	91.03
SK-MEL-5	77.57
UACC-257	85.38
UACC-62	55.56
Ovarian Cancer	
IGROV1	83.63
OVCAR-3	87.39
OVCAR-4	65.76
OVCAR-5	87.56
OVCAR-8	59.39
NCI/ADR-RES	50.98
SK-OV-3	82.00
Renal Cancer	
786-0	67.71
A498	128.44
ACHN	57.87
CAKI-1	47.39
RXF 393	53.41
SN12C	62.97
TK-10	95.20
UO-31	75.46
Prostate Cancer	
PC-3	35.33
DU-145	81.01
Breast Cancer	
MCF7	60.01
MDA-MB-231/ATCC	59.62
BT-549	68.83
T-47D	71.58
MDA-MB-468	47.77
Mean	64.72
Delta	65.55
Range	129.27

Mean Growth Percent - Growth Percent

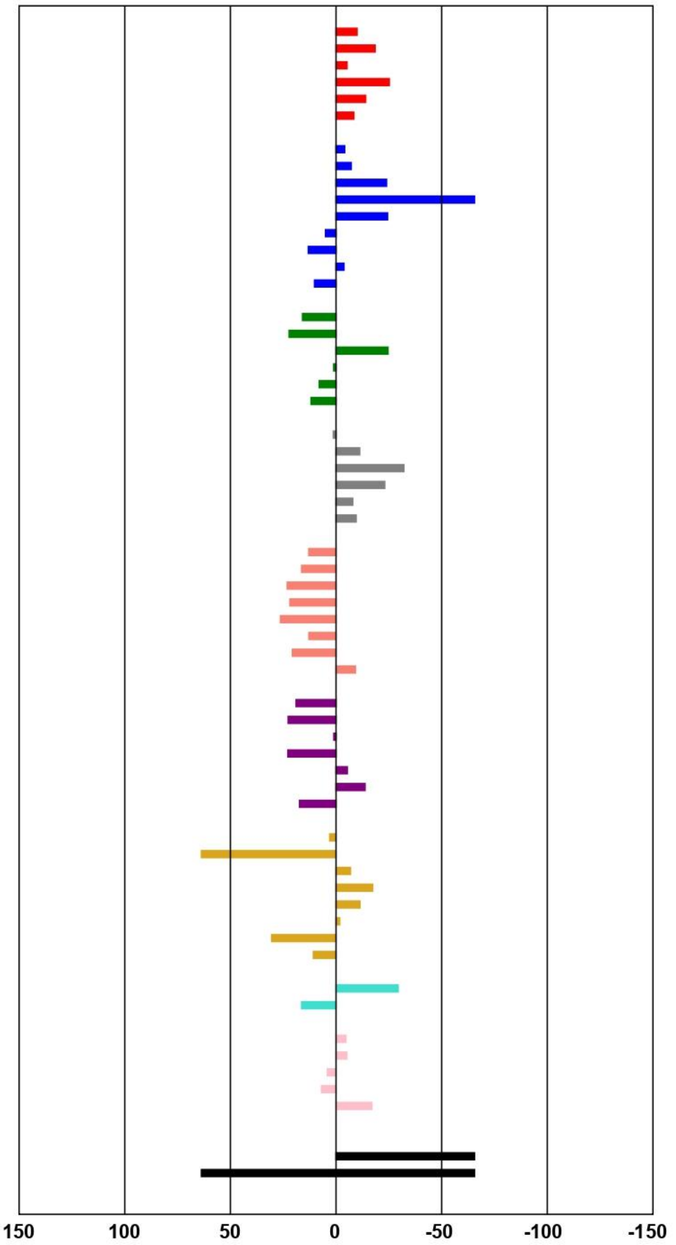

Figure S121. One dose mean graph for compound **10a** (NSC 838214) at 10 μM

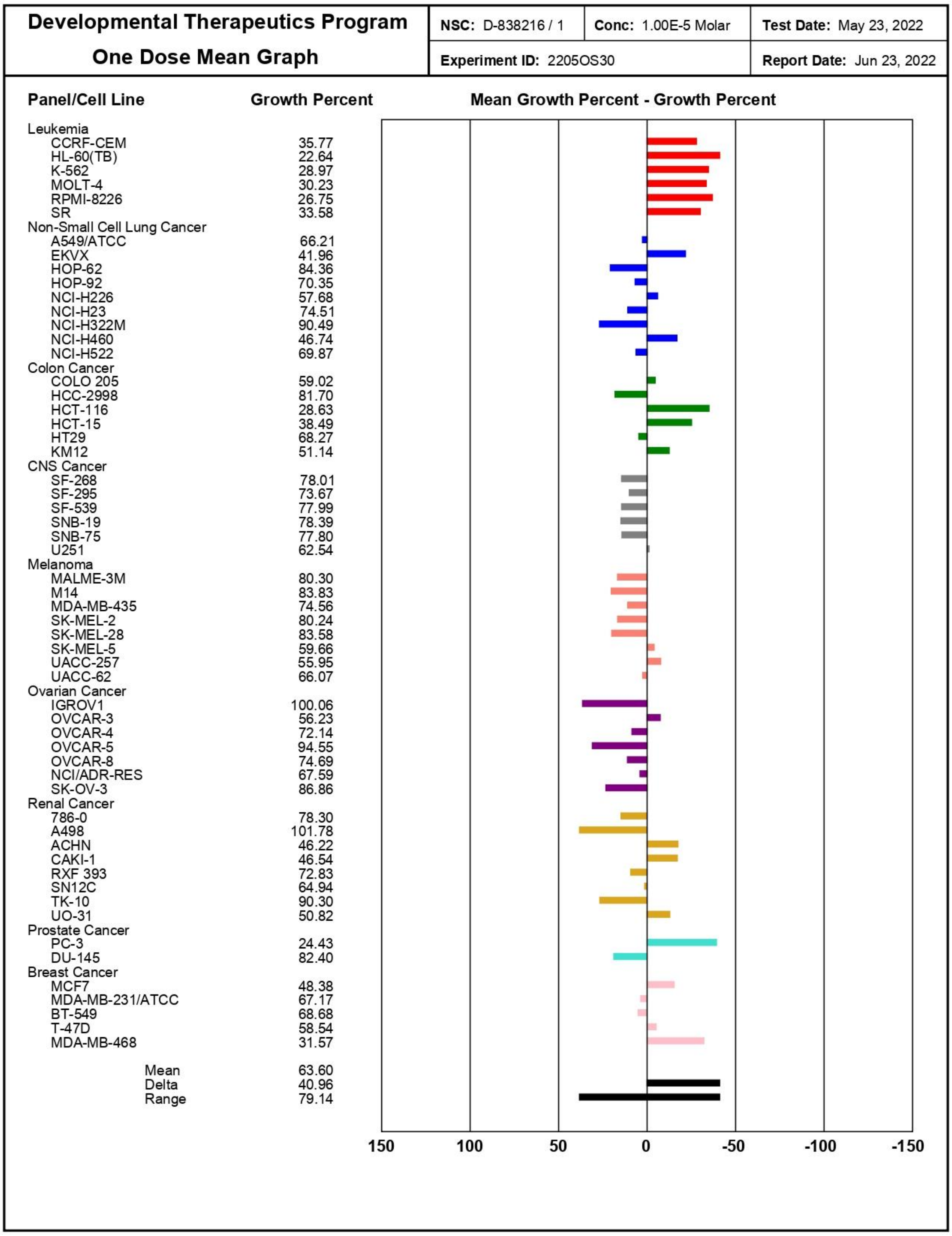

Figure S122. One dose mean graph for compound **10b** (NSC 838216) at 10 μM

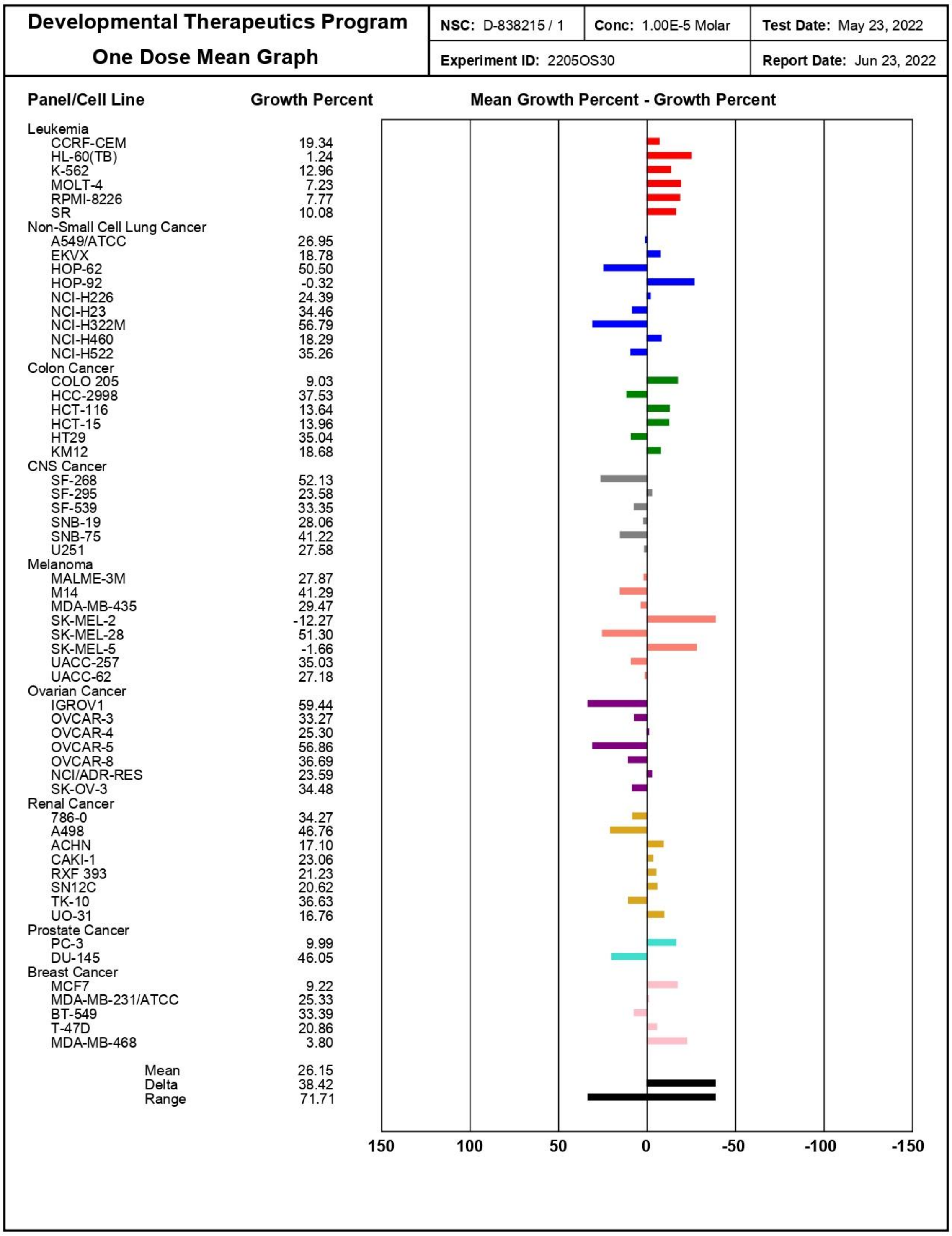

Figure S123. One dose mean graph for compound **10c** (NSC 838215) at 10 μM

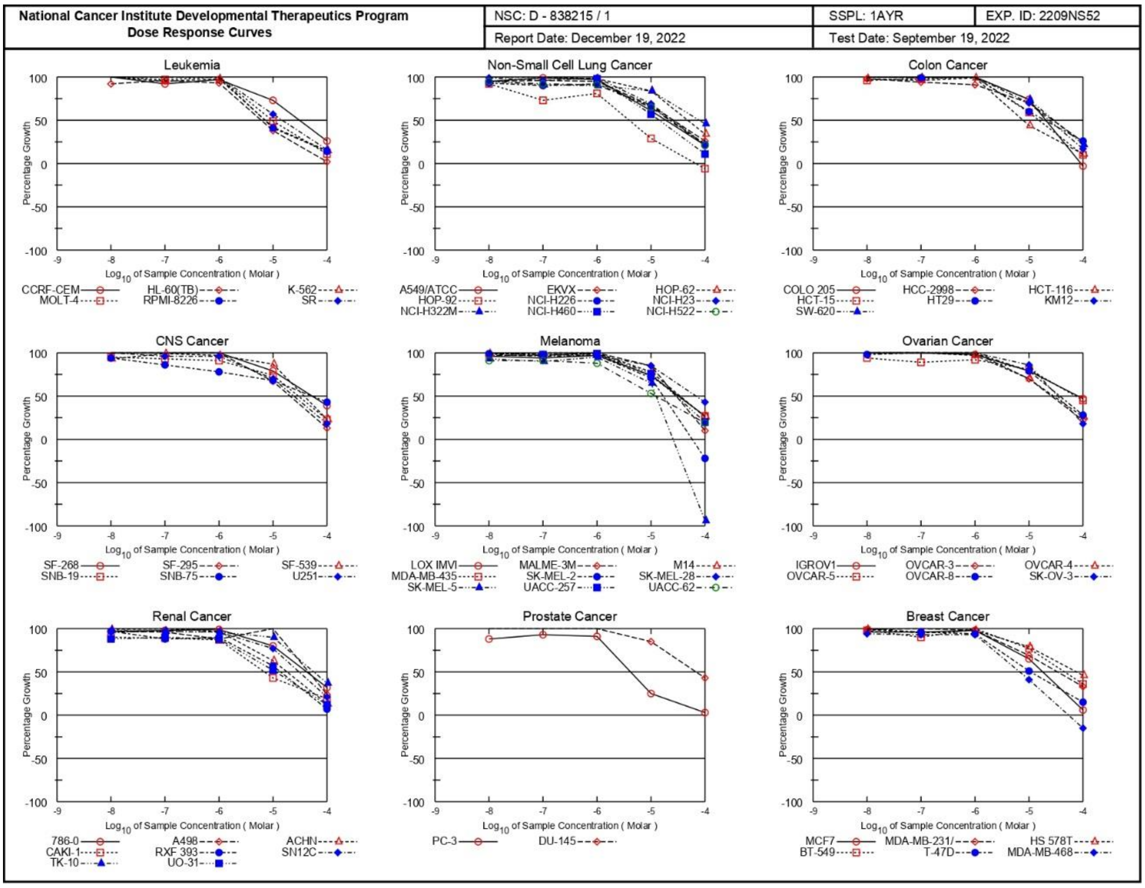

Figure S124. Dose response curves (percentage growth versus concentration of compound at NCI fixed protocol, µM) for tested compound **10c** (NSC 838215) against the nine different panels of NCI cancer cell lines at five concentrations (from 10^{-4} mol/L to 10^{-8} mol/L)

National Cancer Institute Developmental Therapeutics Program
In-Vitro Testing Results

NSC : D - 838215 / 1	Experiment ID : 2209NS52	Test Type : 08	Units : Molar
Report Date : December 19, 2022	Test Date : September 19, 2022	QNS :	MC :
COMI : MG_10b	Stain Reagent : SRB Dual-Pass Related	SSPL : 1AYR	

			Log10 Concentration													
	Time		Mean Optical Densities					Percent Growth								
Panel/Cell Line	Zero	Ctrl	-8.0	-7.0	-6.0	-5.0	-4.0	-8.0	-7.0	-6.0	-5.0	-4.0	GI50	TGI	LC50
Leukemia															
CCRF-CEM	0.482	2.204	2.239	2.074	2.156	1.739	0.934	102	92	97	73	26	3.10E-5	> 1.00E-4	> 1.00E-4
HL-60(TB)	0.593	2.287	2.159	2.223	2.164	1.229	0.632	92	96	93	38	2	5.95E-6	> 1.00E-4	> 1.00E-4
K-562	0.227	2.165	2.209	2.071	2.118	1.045	0.508	102	95	98	42	15	7.23E-6	> 1.00E-4	> 1.00E-4
MOLT-4	0.570	2.199	2.257	2.151	2.210	1.368	0.747	104	97	101	49	11	9.56E-6	> 1.00E-4	> 1.00E-4
RPMI-8226	0.778	2.810	2.907	2.882	2.864	1.605	1.071	105	104	103	41	14	7.07E-6	> 1.00E-4	> 1.00E-4
SR	0.428	2.319	2.313	2.355	2.322	1.505	0.723	100	102	100	57	16	1.47E-5	> 1.00E-4	> 1.00E-4
Non-Small Cell Lung Cancer															
A549/ATCC	0.360	2.397	2.304	2.379	2.338	1.605	0.778	95	99	97	61	21	1.88E-5	> 1.00E-4	> 1.00E-4
EKVX	0.599	1.794	1.734	1.749	1.738	1.403	0.910	95	96	95	67	26	2.62E-5	> 1.00E-4	> 1.00E-4
HOP-62	0.661	2.482	2.609	2.529	2.448	2.198	1.289	107	103	98	84	34	4.88E-5	> 1.00E-4	> 1.00E-4
HOP-92	1.343	1.890	1.846	1.744	1.784	1.501	1.256	92	73	81	29	-6	3.89E-6	6.55E-5	> 1.00E-4
NCI-H226	0.921	1.886	1.820	1.786	1.804	1.562	1.133	93	90	92	66	22	2.34E-5	> 1.00E-4	> 1.00E-4
NCI-H23	0.586	1.684	1.673	1.644	1.674	1.342	0.826	99	96	99	69	22	2.51E-5	> 1.00E-4	> 1.00E-4
NCI-H322M	0.694	1.916	1.854	1.804	1.798	1.718	1.253	95	91	90	84	46	7.71E-5	> 1.00E-4	> 1.00E-4
NCI-H460	0.245	2.636	2.695	2.778	2.582	1.597	0.505	102	106	98	57	11	1.39E-5	> 1.00E-4	> 1.00E-4
NCI-H522	0.878	2.690	2.597	2.550	2.532	2.060	1.265	95	92	91	65	21	2.22E-5	> 1.00E-4	> 1.00E-4
Colon Cancer															
COLO 205	0.361	1.557	1.660	1.638	1.686	1.249	0.350	109	107	111	74	-3	2.06E-5	9.13E-5	> 1.00E-4
HCC-2998	0.725	2.465	2.429	2.367	2.301	1.942	1.158	98	94	91	70	25	2.77E-5	> 1.00E-4	> 1.00E-4
HCT-116	0.310	2.796	2.747	2.706	2.776	1.413	0.581	98	96	99	44	11	7.89E-6	> 1.00E-4	> 1.00E-4
HCT-15	0.403	2.458	2.370	2.427	2.456	1.615	0.603	96	98	100	59	10	1.52E-5	> 1.00E-4	> 1.00E-4
HT29	0.322	2.339	2.355	2.325	2.351	1.539	0.837	101	99	101	60	26	1.98E-5	> 1.00E-4	> 1.00E-4
KM12	0.460	2.269	2.389	2.402	2.376	1.724	0.761	107	107	106	70	17	2.36E-5	> 1.00E-4	> 1.00E-4
SW-620	0.252	1.992	2.098	1.978	2.018	1.548	0.627	106	99	102	74	22	2.90E-5	> 1.00E-4	> 1.00E-4
CNS Cancer															
SF-268	0.976	2.665	2.769	2.661	2.666	2.309	1.636	106	100	100	79	39	5.32E-5	> 1.00E-4	> 1.00E-4
SF-295	0.762	2.297	2.311	2.325	2.329	1.783	0.958	101	102	102	67	13	2.03E-5	> 1.00E-4	> 1.00E-4
SF-539	0.981	2.666	2.556	2.643	2.615	2.441	1.372	93	99	97	87	23	3.78E-5	> 1.00E-4	> 1.00E-4
SNB-19	0.760	2.218	2.143	2.115	2.080	1.837	1.089	95	93	91	74	23	2.92E-5	> 1.00E-4	> 1.00E-4
SNB-75	1.280	2.271	2.213	2.136	2.057	1.952	1.710	94	86	78	68	43	5.33E-5	> 1.00E-4	> 1.00E-4
U251	0.321	1.826	1.858	1.770	1.767	1.380	0.597	102	96	96	70	18	2.46E-5	> 1.00E-4	> 1.00E-4
Melanoma															
LOX IMVI	0.420	2.472	2.398	2.343	2.402	1.937	0.976	96	94	97	74	27	3.24E-5	> 1.00E-4	> 1.00E-4
MALME-3M	0.720	1.470	1.451	1.446	1.469	1.359	0.793	97	97	100	85	10	2.93E-5	> 1.00E-4	> 1.00E-4
M14	0.451	1.768	1.752	1.769	1.804	1.482	0.795	99	100	103	78	26	3.48E-5	> 1.00E-4	> 1.00E-4
MDA-MB-435	0.514	2.390	2.420	2.330	2.371	1.892	1.024	102	97	99	73	27	3.21E-5	> 1.00E-4	> 1.00E-4
SK-MEL-2	1.139	2.562	2.543	2.533	2.522	2.156	0.887	99	98	97	71	-22	1.70E-5	5.80E-5	> 1.00E-4
SK-MEL-28	0.644	1.826	1.800	1.775	1.795	1.649	1.147	98	96	97	85	43	6.67E-5	> 1.00E-4	> 1.00E-4
SK-MEL-5	0.853	2.762	2.633	2.563	2.662	2.095	0.051	93	90	95	65	-94	1.24E-5	2.56E-5	5.29E-5
UACC-257	0.935	2.557	2.552	2.532	2.543	2.166	1.252	100	98	99	76	20	2.88E-5	> 1.00E-4	> 1.00E-4
UACC-62	0.971	2.743	2.590	2.578	2.528	1.918	1.311	91	91	88	53	19	1.26E-5	> 1.00E-4	> 1.00E-4
Ovarian Cancer															
IGROV1	0.756	2.535	2.567	2.537	2.495	2.156	1.590	102	100	98	79	47	7.97E-5	> 1.00E-4	> 1.00E-4
OVCAR-3	0.390	1.445	1.609	1.477	1.513	1.128	0.625	115	103	106	70	22	2.62E-5	> 1.00E-4	> 1.00E-4
OVCAR-4	1.095	2.486	2.527	2.486	2.446	2.069	1.454	103	100	97	70	26	2.83E-5	> 1.00E-4	> 1.00E-4
OVCAR-5	0.558	1.405	1.352	1.315	1.338	1.245	0.939	94	89	92	81	45	7.26E-5	> 1.00E-4	> 1.00E-4
OVCAR-8	0.462	2.454	2.409	2.446	2.470	2.033	1.026	98	100	101	79	28	3.72E-5	> 1.00E-4	> 1.00E-4
SK-OV-3	0.783	2.063	2.183	2.076	2.281	1.880	1.011	109	101	117	86	18	3.35E-5	> 1.00E-4	> 1.00E-4
Renal Cancer															
786-0	0.895	2.880	2.821	2.846	2.870	2.485	1.534	97	98	99	80	32	4.24E-5	> 1.00E-4	> 1.00E-4
A498	1.295	1.983	1.962	1.956	1.899	1.988	1.458	97	96	88	101	24	4.54E-5	> 1.00E-4	> 1.00E-4
ACHN	0.707	2.639	2.622	2.642	2.583	1.930	0.955	99	100	97	63	13	1.83E-5	> 1.00E-4	> 1.00E-4
CAKI-1	0.596	2.331	2.157	2.140	2.097	1.338	0.933	90	89	87	43	19	6.82E-6	> 1.00E-4	> 1.00E-4
RXF 393	1.184	1.911	1.888	1.825	1.840	1.598	1.235	97	88	90	57	7	1.38E-5	> 1.00E-4	> 1.00E-4
SN12C	1.270	3.260	3.179	3.192	3.184	2.811	1.694	96	97	96	77	21	3.08E-5	> 1.00E-4	> 1.00E-4
TK-10	0.988	2.724	2.683	2.674	2.659	2.548	1.626	98	97	96	90	37	5.63E-5	> 1.00E-4	> 1.00E-4
UO-31	0.786	2.382	2.198	2.216	2.187	1.623	0.976	88	90	88	52	12	1.15E-5	> 1.00E-4	> 1.00E-4
Prostate Cancer															
PC-3	0.720	2.615	2.387	2.485	2.436	1.191	0.778	88	93	91	25	3	4.14E-6	> 1.00E-4	> 1.00E-4
DU-145	0.503	2.081	2.265	2.198	2.190	1.849	1.175	112	107	107	85	43	6.71E-5	> 1.00E-4	> 1.00E-4
Breast Cancer															
MCF7	0.508	2.294	2.216	2.219	2.262	1.661	0.614	96	96	98	65	6	1.77E-5	> 1.00E-4	> 1.00E-4
MDA-MB-231/ATCC	0.634	1.380	1.373	1.387	1.366	1.147	0.880	99	101	98	69	33	3.34E-5	> 1.00E-4	> 1.00E-4
HS 578T	1.085	2.111	2.101	2.060	2.091	1.896	1.560	99	95	98	79	46	7.68E-5	> 1.00E-4	> 1.00E-4
BT-549	1.003	2.182	2.162	2.062	2.226	1.916	1.427	98	90	104	77	36	4.58E-5	> 1.00E-4	> 1.00E-4
T-47D	0.952	2.300	2.335	2.252	2.217	1.634	1.153	103	96	94	51	15	1.04E-5	> 1.00E-4	> 1.00E-4
MDA-MB-468	0.702	1.541	1.489	1.482	1.485	1.050	0.595	94	93	93	41	-15	6.83E-6	5.38E-5	> 1.00E-4

Figure S125. Cytotoxic activity of compound **10c** (NSC 838215) against the NCI 60 human cancer cell line at five doses.

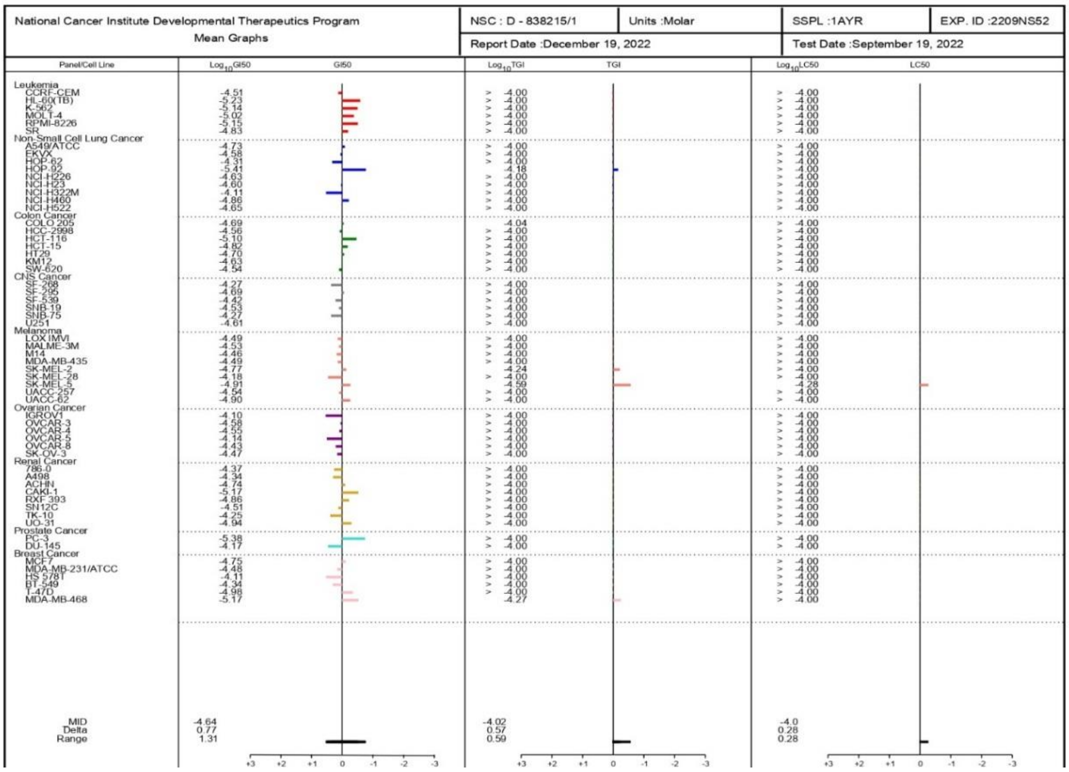

Figure S126. Mean graphs of log_{10} values (Molar) of GI_{50}, TGI and LC_{50} of compound **10c** (NSC 838215) obtained from the NCI 60 cell line experiments

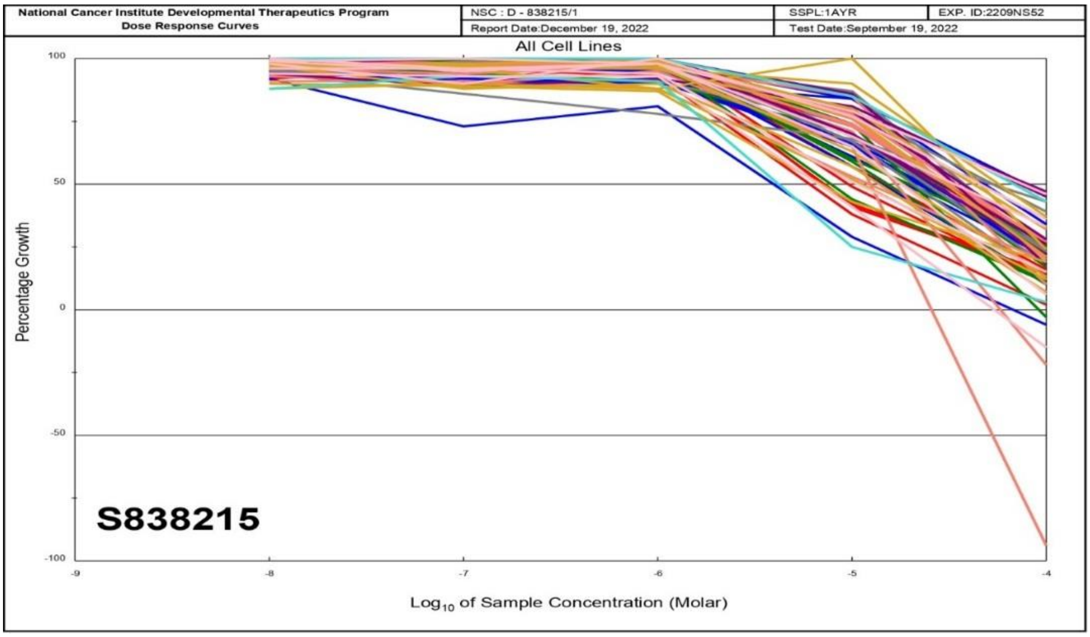

Figure S127. Dose response curves (percentage growth versus concentration of compound at NCI fixed protocol, μM) for tested compound **10c** (NSC 838215) against the nine different panels of NCI cancer cell lines at five concentrations (from 10^{-4} mol/L to 10^{-8} mol/L).

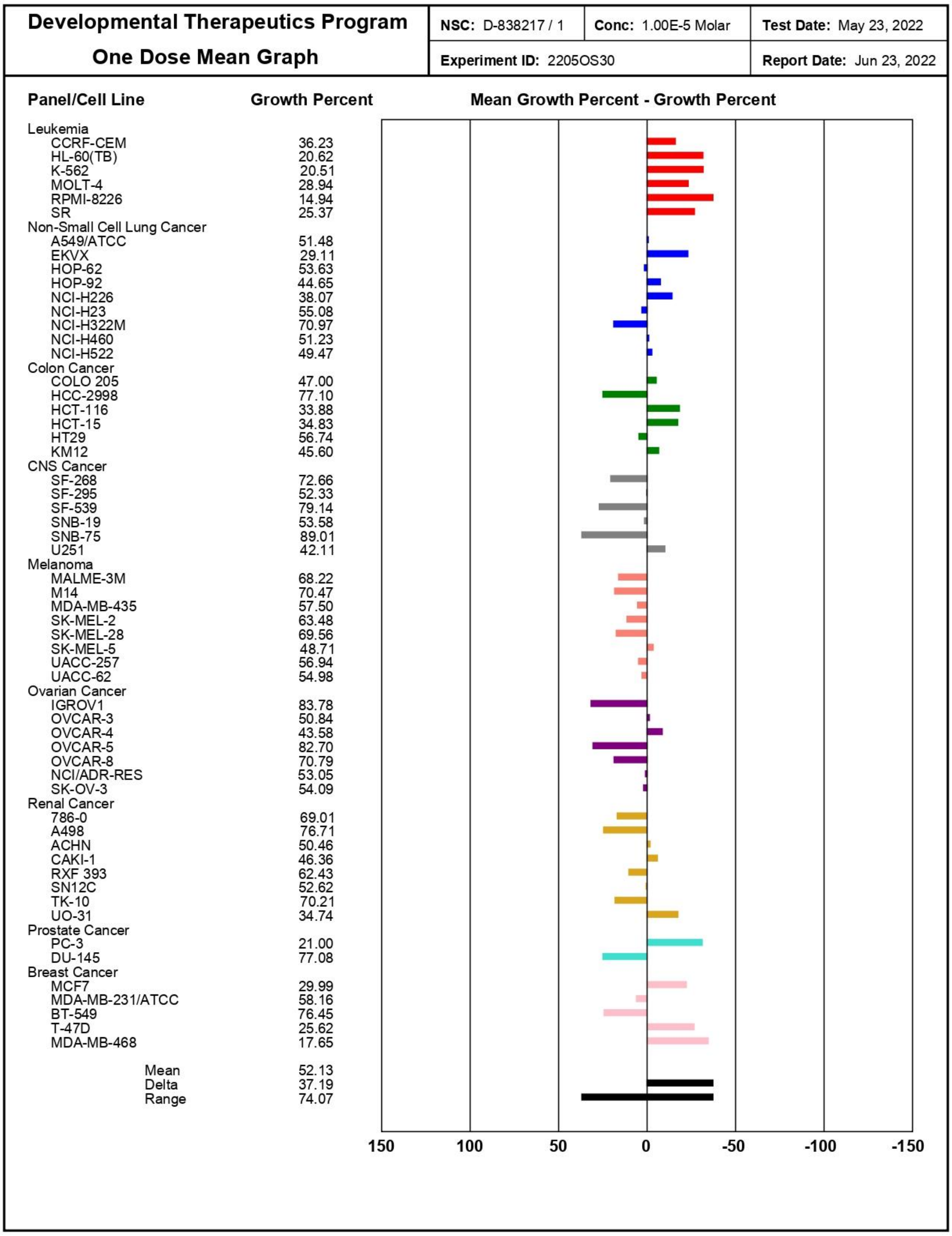

Figure S128. One dose mean graph for compound **10d** (NSC 838217) at 10 μM

Developmental Therapeutics Program | **NSC:** D-832910 / 1 | **Conc:** 1.00E-5 Molar | **Test Date:** Oct 18, 2021

One Dose Mean Graph | **Experiment ID:** 2110OS01 | **Report Date:** Apr 01, 2022

Panel/Cell Line	Growth Percent
Leukemia	
CCRF-CEM	108.74
HL-60(TB)	91.55
K-562	100.79
MOLT-4	101.64
RPMI-8226	110.78
SR	84.11
Non-Small Cell Lung Cancer	
A549/ATCC	108.08
EKVX	100.00
HOP-62	114.47
HOP-92	131.11
NCI-H226	107.63
NCI-H322M	97.67
NCI-H460	113.73
NCI-H522	88.56
Colon Cancer	
COLO 205	117.10
HCC-2998	105.50
HCT-116	109.43
HCT-15	91.94
HT29	113.77
KM12	99.13
SW-620	107.09
CNS Cancer	
SF-268	101.06
SF-295	110.08
SF-539	103.73
SNB-19	100.47
SNB-75	81.13
U251	104.06
Melanoma	
LOX IMVI	93.43
MALME-3M	108.50
M14	100.48
MDA-MB-435	95.02
SK-MEL-2	110.59
SK-MEL-28	99.12
SK-MEL-5	101.33
UACC-257	109.05
UACC-62	98.55
Ovarian Cancer	
IGROV1	107.25
OVCAR-3	118.22
OVCAR-4	108.87
OVCAR-5	104.91
OVCAR-8	102.37
NCI/ADR-RES	107.36
SK-OV-3	112.11
Renal Cancer	
786-0	100.31
A498	120.78
ACHN	103.14
CAKI-1	96.12
RXF 393	123.66
SN12C	104.25
TK-10	106.83
UO-31	90.32
Prostate Cancer	
PC-3	95.52
DU-145	117.11
Breast Cancer	
MCF7	97.54
MDA-MB-231/ATCC	106.66
HS 578T	104.03
BT-549	105.69
T-47D	99.27
MDA-MB-468	100.36
Mean	104.27
Delta	23.14
Range	49.98

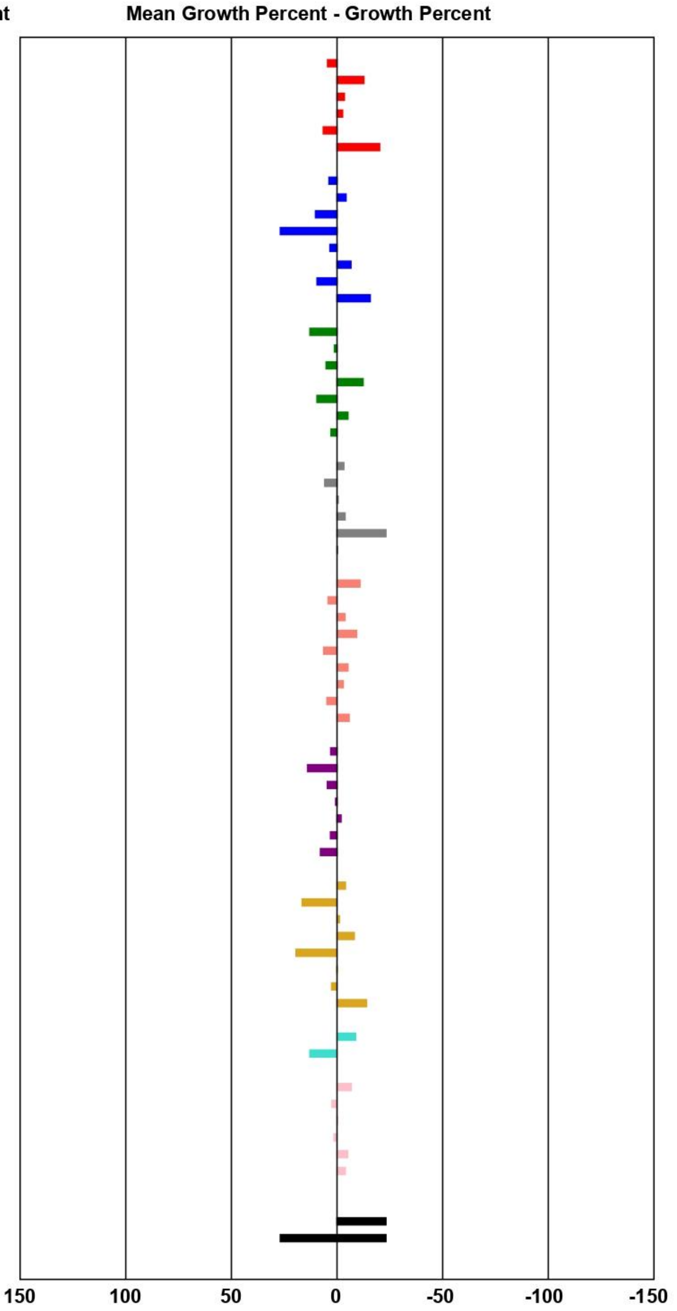

Figure S129. One dose mean graph for compound **12a** (NSC 832910) at 10 μM

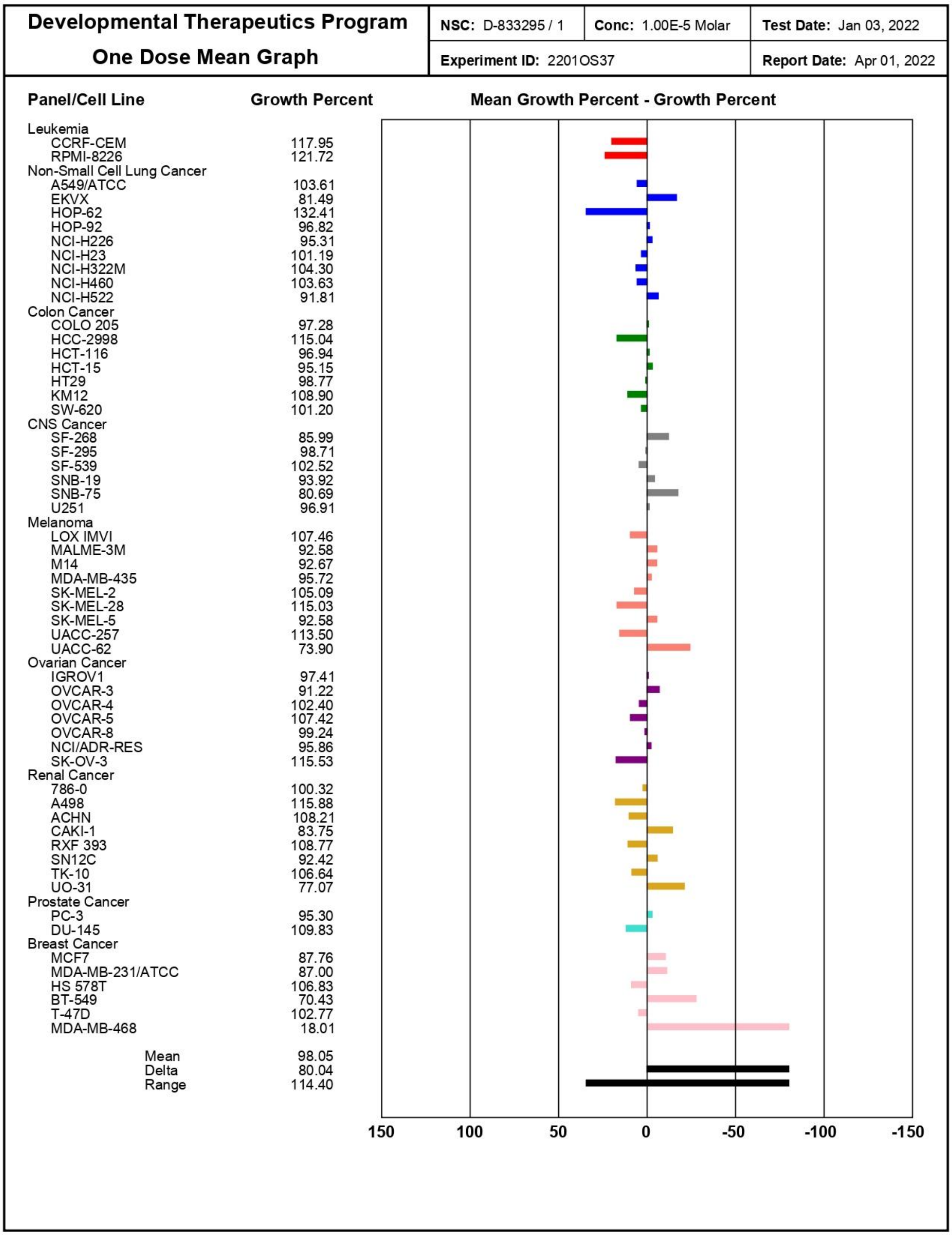

Figure S130. One dose mean graph for compound **12b** (NSC 833295) at 10 μM

Developmental Therapeutics Program	NSC: D-833298 / 1	Conc: 1.00E-5 Molar	Test Date: Jan 03, 2022
One Dose Mean Graph	Experiment ID: 2201OS37		Report Date: Apr 01, 2022

Panel/Cell Line	Growth Percent
Leukemia	
CCRF-CEM	102.70
RPMI-8226	104.34
Non-Small Cell Lung Cancer	
A549/ATCC	98.35
EKVX	84.79
HOP-62	142.09
HOP-92	98.54
NCI-H226	101.24
NCI-H23	105.44
NCI-H322M	101.70
NCI-H460	107.82
NCI-H522	88.82
Colon Cancer	
COLO 205	96.84
HCC-2998	109.51
HCT-116	87.01
HCT-15	87.97
HT29	77.62
KM12	101.07
SW-620	103.38
CNS Cancer	
SF-268	89.78
SF-295	94.15
SF-539	104.13
SNB-19	94.37
SNB-75	90.12
U251	95.17
Melanoma	
LOX IMVI	102.07
MALME-3M	93.99
M14	95.63
MDA-MB-435	96.90
SK-MEL-2	102.69
SK-MEL-28	114.23
SK-MEL-5	86.51
UACC-257	100.60
UACC-62	75.19
Ovarian Cancer	
IGROV1	105.10
OVCAR-3	100.76
OVCAR-4	97.91
OVCAR-5	114.75
OVCAR-8	100.85
NCI/ADR-RES	96.26
SK-OV-3	116.86
Renal Cancer	
786-0	94.63
A498	111.11
ACHN	110.23
CAKI-1	88.55
RXF 393	101.46
SN12C	91.34
TK-10	111.24
UO-31	77.23
Prostate Cancer	
PC-3	80.58
DU-145	102.63
Breast Cancer	
MCF7	89.44
MDA-MB-231/ATCC	91.08
HS 578T	108.21
BT-549	99.82
T-47D	99.01
MDA-MB-468	90.31
Mean	98.47
Delta	23.28
Range	66.90

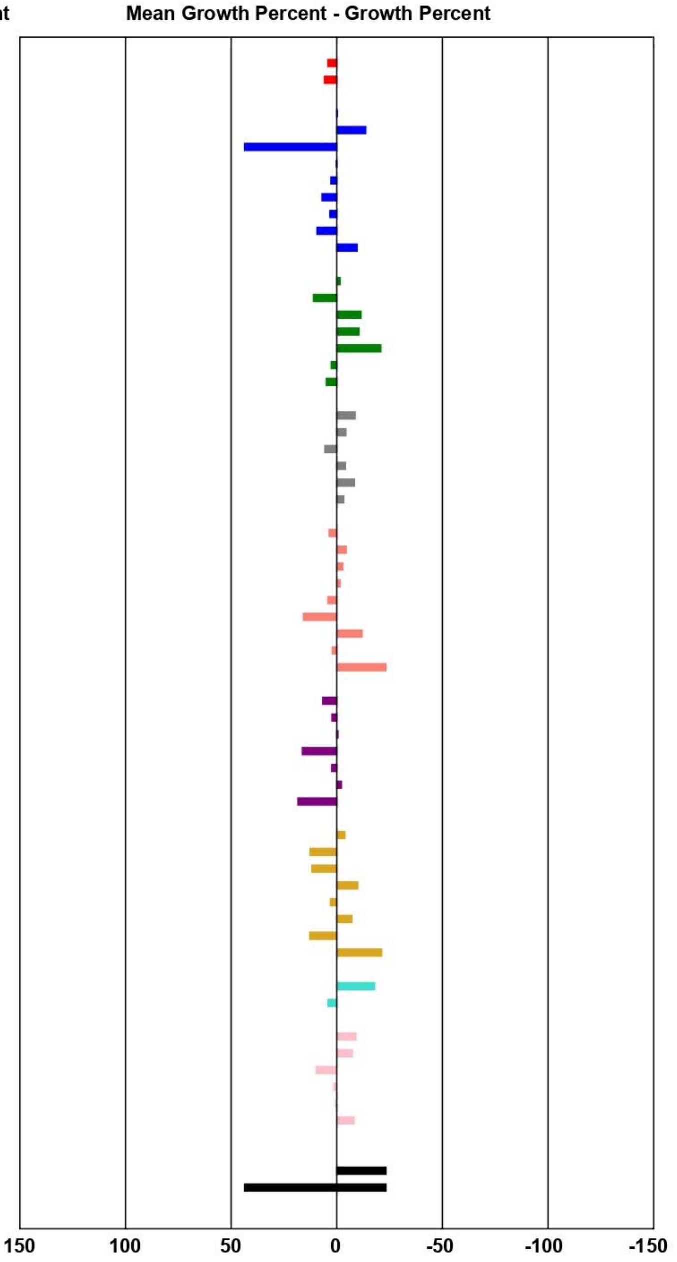

Figure S131. One dose mean graph for compound **12c** (NSC 833298) at 10 μM

Developmental Therapeutics Program | **NSC:** D-835583 / 1 | **Conc:** 1.00E-5 Molar | **Test Date:** Feb 28, 2022

One Dose Mean Graph | **Experiment ID:** 2202OS75 | **Report Date:** Apr 01, 2022

Panel/Cell Line	Growth Percent
Leukemia	
CCRF-CEM	101.81
HL-60(TB)	102.48
K-562	97.57
MOLT-4	106.47
RPMI-8226	101.09
SR	93.38
Non-Small Cell Lung Cancer	
A549/ATCC	96.40
EKVX	100.58
HOP-62	103.26
HOP-92	104.64
NCI-H226	97.74
NCI-H23	100.26
NCI-H322M	93.29
NCI-H460	123.13
NCI-H522	85.03
Colon Cancer	
COLO 205	113.61
HCC-2998	97.61
HCT-116	96.23
HCT-15	105.42
HT29	103.96
KM12	101.99
SW-620	106.98
CNS Cancer	
SF-268	108.13
SF-295	95.33
SF-539	96.16
SNB-19	101.51
SNB-75	83.13
U251	107.47
Melanoma	
LOX IMVI	90.43
MALME-3M	104.44
M14	104.96
MDA-MB-435	108.61
SK-MEL-2	107.11
SK-MEL-28	104.51
SK-MEL-5	95.09
UACC-257	106.26
UACC-62	87.93
Ovarian Cancer	
IGROV1	98.00
OVCAR-3	112.58
OVCAR-4	99.10
OVCAR-5	93.49
OVCAR-8	102.33
NCI/ADR-RES	86.57
SK-OV-3	99.07
Renal Cancer	
786-0	103.31
A498	104.47
ACHN	97.98
CAKI-1	96.78
SN12C	99.89
TK-10	100.56
UO-31	23.17
Prostate Cancer	
PC-3	100.56
DU-145	107.38
Breast Cancer	
MCF7	108.67
MDA-MB-231/ATCC	100.04
HS 578T	91.01
BT-549	102.90
T-47D	96.83
MDA-MB-468	93.99
Mean	99.20
Delta	76.03
Range	99.96

Mean Growth Percent - Growth Percent

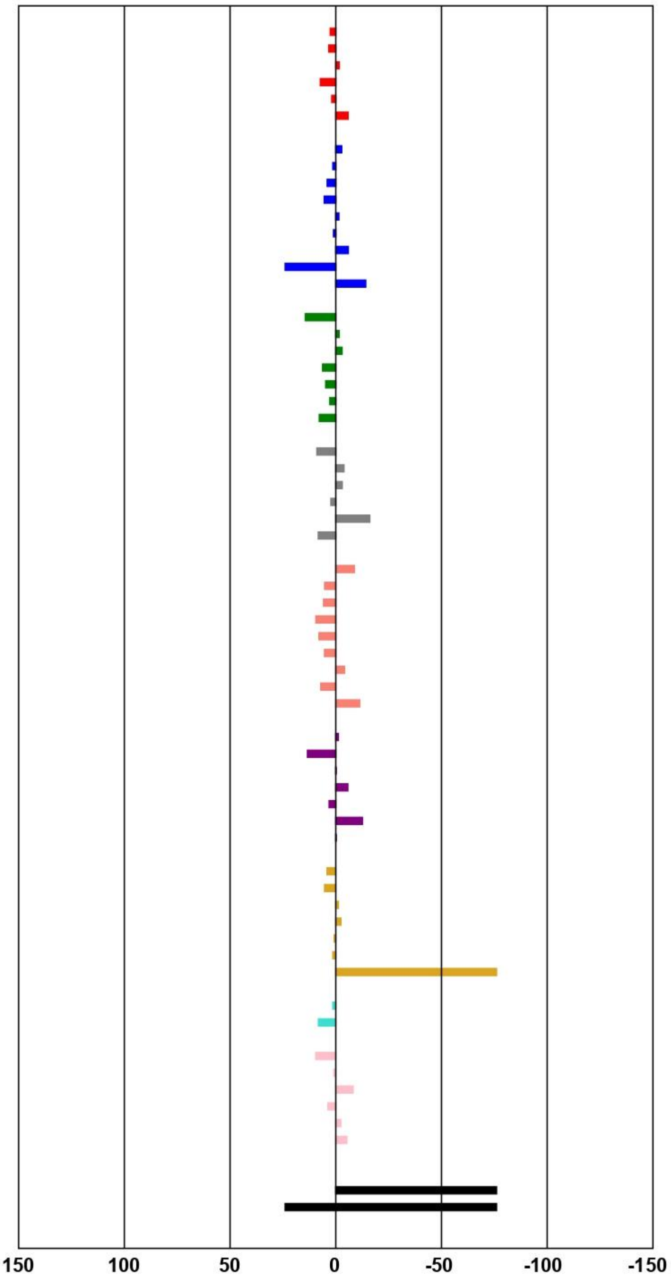

Figure S132. One dose mean graph for compound **12d** (NSC 835583) at 10 μM

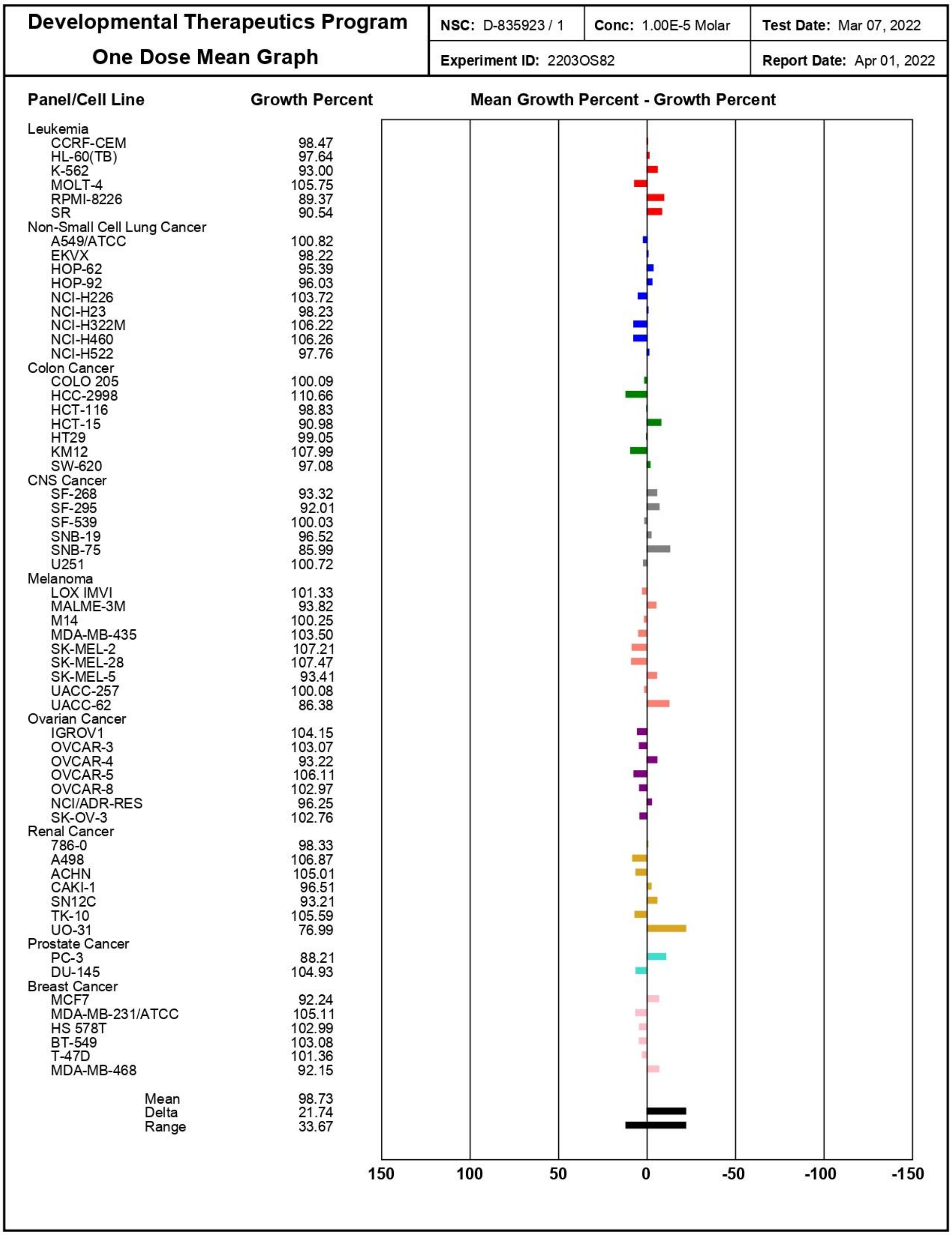

Figure S133. One dose mean graph for compound **12e** (NSC 835923) at 10 μM

Developmental Therapeutics Program	NSC: D-834380 / 1	Conc: 1.00E-5 Molar	Test Date: Feb 14, 2022
One Dose Mean Graph	Experiment ID: 2202OS68		Report Date: Apr 01, 2022

Panel/Cell Line	Growth Percent
**Leukemia**	
CCRF-CEM	106.27
HL-60(TB)	99.88
K-562	97.57
MOLT-4	105.75
RPMI-8226	87.82
SR	102.03
**Non-Small Cell Lung Cancer**	
A549/ATCC	85.04
EKVX	101.77
HOP-62	103.69
HOP-92	115.43
NCI-H226	132.27
NCI-H23	101.08
NCI-H322M	121.21
NCI-H460	102.94
NCI-H522	106.91
**Colon Cancer**	
COLO 205	98.41
HCC-2998	100.48
HCT-116	95.71
HCT-15	101.46
HT29	102.88
KM12	99.49
SW-620	101.21
**CNS Cancer**	
SF-268	94.77
SF-295	100.24
SF-539	87.91
SNB-19	99.69
SNB-75	78.85
U251	93.80
**Melanoma**	
LOX IMVI	88.07
MALME-3M	91.50
M14	99.39
MDA-MB-435	92.73
SK-MEL-2	110.39
SK-MEL-28	116.36
SK-MEL-5	94.82
UACC-257	90.32
UACC-62	98.81
**Ovarian Cancer**	
IGROV1	118.34
OVCAR-3	103.49
OVCAR-4	86.98
OVCAR-5	137.87
OVCAR-8	90.44
NCI/ADR-RES	89.31
SK-OV-3	107.73
**Renal Cancer**	
786-0	97.00
A498	99.17
ACHN	100.01
CAKI-1	93.49
RXF 393	115.05
SN12C	105.31
TK-10	98.86
UO-31	79.47
**Prostate Cancer**	
PC-3	99.01
DU-145	104.49
**Breast Cancer**	
MCF7	89.94
MDA-MB-231/ATCC	93.87
HS 578T	84.00
BT-549	90.09
T-47D	74.49
MDA-MB-468	106.51
Mean	99.53
Delta	25.04
Range	63.38

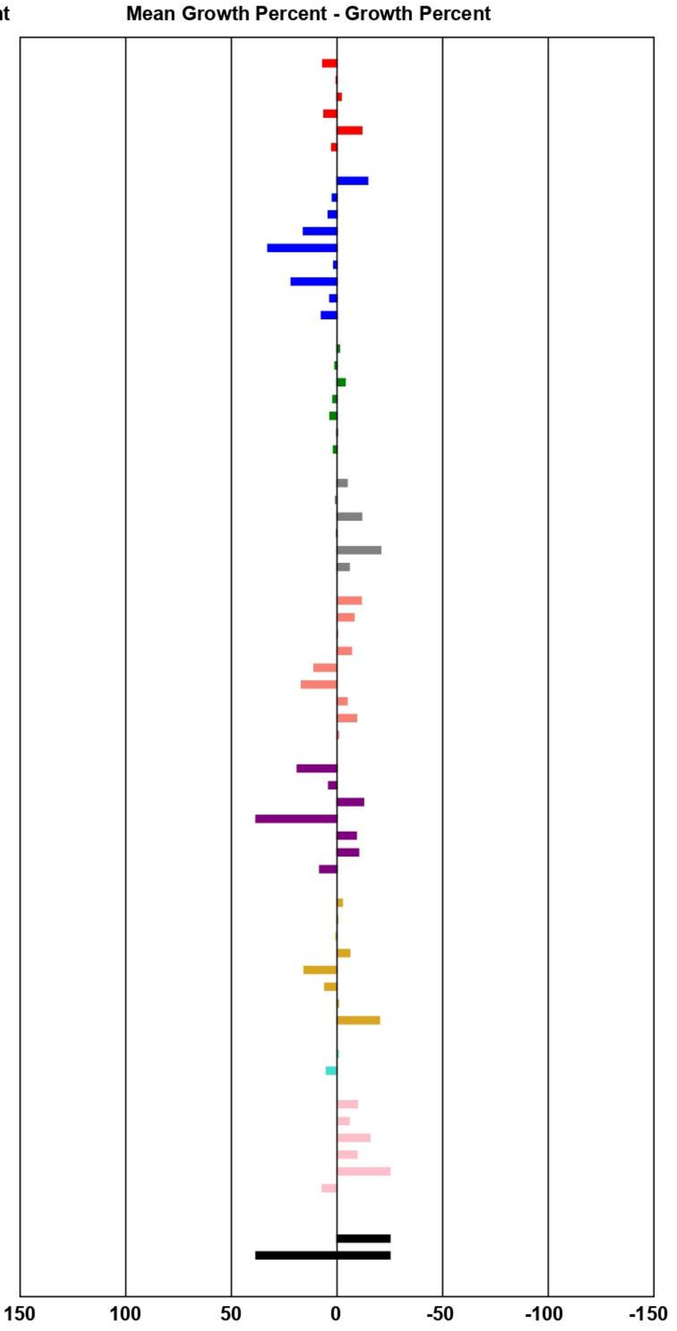

Figure S134. One dose mean graph for compound **12f** (NSC 834380) at 10 μM

Table S1. GI % mean of the target compounds (**6a-i** and **8a-m**) across the NCI-60 human cancer cell line panel

Subpanel cell lines	Growth Inhibition Percentage (GI %)																				
	**6a**	**6b**	**6c**	**6d**	**6e**	**6f**	**6g**	**6h**	**6i**	**8a**	**8b**	**8c**	**8d**	**8e**	**8f**	**8g**	**8h**	**8i**	**8j**	**8k**	**8l**	**8m**
**Leukemia**																						
CCRF-CEM	52	-	40	20	60	33	-	-	56	-	26	85	-	61	-	-	-	11	-	-	-	-
HL-60(TB)	123	-	75	34	111	68	-	-	69	-	32	94	21	62	-	-	-	-	-	22	-	-
K-562	59	-	-	20	50	-	-	-	66	-	-	82	-	63	-	-	-	16	-	26	-	-
MOLT-4	75	-	-	25	71	-	-	-	89	-	-	99	-	89	14	-	-	22	-	27	-	-
RPMI-8226	78	22	-	35	64	-	-	-	73	-	-	92	-	90	15	-	-	17	-	43	-	-
SR	68	-	-	14	34	-	-	-	64	-	-	87	-	52	18	-	-	34	-	32	16	-
**NSC Lung Cancer**																						
A549/ATCC	25	13	51	13	19	31	-	-	52	-	12	**66**	14	**62**	-	-	-	-	-	11	-	-
EKVX	26	15	30	11	-	25	-	-	44	-	25	**72**	15	33	-	-	-	14	-	15	-	25
HOP-62	-	-	19	-	-	-	-	-	-	-	-	19	-	-	-	-	-	-	-	-	-	-
HOP-92	32	-	29	27	28	13	-	-	12	-	21	30	-	-	-	-	-	-	-	-	12	-
NCI-H226	24	-	25	11	15	14	-	-	30	-	-	30	-	-	12	-	-	12	-	15	-	-
NCI-H322M	18	-	-	-	-	-	-	-	15	-	-	35	-	-	-	-	-	-	-	-	-	-
NCI-H460	40	20	58	20	46	43	-	-	51	-	-	**87**	21	59	-	-	-	-	-	-	-	-
NCI-H522	43	17	**63**	23	32	43	-	-	43	-	40	**62**	15	19	-	-	-	21	-	27	11	11
**Colon Cancer**																						
COLO 205	35	19	**62**	-	37	45	-	-	50	-	13	**118**	23	53	-	-	-	-	-	-	-	-
HCC-2998	15	-	24	-	11	-	-	-	38	-	-	57	-	11	-	-	-	-	-	-	-	-
HCT-116	59	17	**66**	21	**60**	**60**	-	-	**65**	-	34	**81**	14	45	14	-	-	16	-	34	-	-
HCT-15	51	30	**62**	17	42	53	-	-	59	-	34	**85**	35	58	-	-	-	14	-	18	-	-
HT29	45	30	**60**	17	37	44	-	-	44	-	39	**65**	33	46	-	-	-	-	-	-	-	-
KM 12	42	-	44	-	16	40	-	-	54	-	-	**82**	-	38	-	-	-	-	-	12	-	-
SW-620	26	19	48	-	54	38	-	-	23	-	-	**72**	20	33	-	-	-	-	-	-	-	-
**CNS Cancer**																						
SF-268	20	-	23	-	-	17	-	-	27	-	-	42	-	22	-	-	-	-	-	-	-	-
SF-295	25	-	27	-	-	18	11	-	41	-	12	**79**	-	33	-	-	-	-	-	-	-	11
SF-539	-	-	-	-	-	-	-	-	31	-	-	**64**	-	26	-	-	-	-		-	-	-
SNB-19	30	12	27	-	13	11	-	-	28	-	22	**85**	-	31	14	-	-	-	-	-	-	-
SNB-75	34	29	17	33	13	16	-	-	18	-	-	27	35	27	-	13	-	-	-	-	-	-
U251	21	-	54	-	19	40	-	-	28	-	21	58	-	31	-	-	-	-	-	-	-	12
**Melanoma**																						
LOX IMVI	15	-	18	12	11	-	-	-	37	-	14	59	-	11	-	-	-	11	-	21	-	-
MALME-3M	11	-	30	-	-	18	-	-	40	-	-	84	-	39	-	-	-	-	-	-	-	-
M14	50	-	28	13	17	24	-	-	49	-	14	**72**	-	49	-	-	-	-	-	-	-	-
MDA-MB-435	27	-	29	-	17	-	-	-	51	-	11	78	-	54	-	-	-	-	-	-	-	-
SK-MEL-2	43	-	24	23	15	-	-	-	15	-	20	70	-	-	16	-	-	-	-	19	-	-
SK-MEL-28	19	-	-	-	-	-	-	-	36	-	-	56	-	26	-	-	-	-	-	-	-	-
SK-MEL-5	40	17	**66**	-	23	**79**	-	-	29	-	29	**72**	16	46	-	-	-	-	-	-	-	20
UACC-257	28	-	50	-	16	42	-	-	44	-	11	**72**	-	56	-	-	-	-	-	-	-	-
UACC-62	56	23	45	36	28	36	-	-	44	-	40	**78**	24	28	-	-	-	33	13	29	17	17
**Ovarian Cancer**																						
IGROV1	-	-	-	-	-	-	-	-	27	-	-	40	-	12	-	-	-	-	-	-	-	-
OVCAR-3	38	17	44	-	15	31	-	-	19	-	-	48	16	16	-	-	-	-	-	-	-	-
OVCAR-4	26	-	32	12	17	25	-	-	58	-	16	**71**	-	54	-	-	-	-	-	-	-	-
OVCAR-5	14	-	-	-	-	-	-	-	15	-	-	42	-	-	-	-	-	-	-	-	-	-
OVCAR-8	17	20	30	-	-	14	-	-	12	-	13	39	13	30	-	-	-	-	-	12	-	-
NCI/ADR-RES	27	-	24	-	-	14	-	-	40	-	12	**74**	-	32	-	-	-	-	-	16	-	-
SK-OV-3	-	-	29	-	-	-	-	-	-	-	-	56	-	-	-	-	-		-	-	-	-
**Renal Cancer**																						
786-0	21	-	15	13	17	12	-	-	-	-	20	33	-	23	11	-	-	-	-	-	16	11
A498	-	-	-	-	-	-	-	-	-	-	-	33	-	-	-	-	-	-	-	-	-	-
ACHN	31	-	40	11	14	20	-	-	38	-	-	**78**	-	32	-	-	-	12	-	12	-	-
CAKI-1	54	31	43	36	30	32	-	-	12	-	26	50	30	19	22	-	-	32	-	11	18	13
RXF 393	22	-	-	-	14	-	-	-	-	-	17	-	-	35	-	-	-	-	-	-	-	-
SN 12C	25	-	31	-	15	13	-	-	12	-	-	46	-	-	-	-	-	-	-	-	-	-
TK-10	-	-	43	-	-	22	-	-	-	-	-	20	-	11	-	-	-	-	-	-	-	-
UO-31	45	19	51	14	24	40	26	21	43	-	30	**71**	16	34	-	-	-	-	-	32	-	15
**Prostate Cancer**																						
PC-3	**71**	43	**76**	33	**68**	**71**	11	-	**68**	-	35	**78**	43	58	18	-	-	32	-	26	-	-
DU-145	12	-	-	-	-	-	-	-	-	-	-	42	-	12	-	-	-	-	-	-	-	-
**Breast Cancer**																						
MCF7	44	32	58	33	43	47	-	-	**65**	-	25	**83**	35	56	13	-	-	16	-	19	-	-
MDA-MB-231	16	-	24	-	-	15	-	-	31	-	19	47	-	24	-	-	-	-	-	-	-	13
HS 578T	-	-	-	-	-	-	--	-	32	-	-	28	-	44	12	-	-	-	-	-	-	-
BT-549	22	11	40	-	-	27	-	-	20	-	13	57	-	21	-	-	25	-	-	-	-	**76**
T-47D	48	21	**67**	30	30	49	-	-	40	-	27	**70**	16	42	11	-	-	-	-	27	-	-
MDA-MB-468	50	20	**60**	30	23	53	11	-	39	-	17	**67**	18	33	-	-	-	-	-	18	-	16
GI Mean	33	-	34	12	22	24	-	-	36	-	14	**63**	-	32	-	-	-	-	-	-	-	-

(**-**) = GI % mean less than 10

Table S2. GI % mean of the target compounds (**10a-d** and **12a-f**) across the NCI-60 human cancer cell line panel

Subpanel cell lines	Growth Inhibition Percentage (GI %)									
	**10a**	**10b**	**10c**	**10d**	**12a**	**12b**	**12c**	**12d**	**12e**	**12f**
**Leukemia**										
CCRF-CEM	45	64	80	64	-	-	-	-	-	-
HL-60(TB)	54	77	99	79	-	-	-	-	-	-
K-562	40	71	87	79	-	-	-	-	-	-
MOLT-4	60	70	93	71	-	-	-	-	-	-
RPMI-8226	49	73	92	85	-	-	-	-	11	12
SR	44	66	90	75	16	-	-	-	-	-
**NSC Lung Cancer**										
A549/ATCC	39	34	73	48	-	-	-	-	-	15
EKVX	42	58	81	71	-	19	-	-	-	-
HOP-62	59	16	49	46	-	-	-	-	-	-
HOP-92	101	30	100	55	-	-	-	-	-	-
NCI-H226	60	42	76	62	-	-	-	-	-	-
NCI-H322M	22	-	43	29	-	-	-	-	-	-
NCI-H460	39	53	82	49	-	-	-	-	-	-
NCI-H522	25	30	65	51	11	-	11	15	-	-
**Colon Cancer**										
COLO 205	19	41	91	53	-	-	-	-	-	-
HCC-2998	13	18	62	23	-	-	-	-	-	-
HCT-116	60	71	86	66	-	-	13	-	-	-
HCT-15	34	62	86	65	-	-	12	-	-	-
HT29	27	32	65	43	-	-	22	-	-	-
KM 12	23	49	81	54	-	-	-	-	-	-
SW-620	-	-	-	-	-	-	-	-	-	-
**CNS Cancer**										
SF-268	34	22	48	27	-	14	-	-	-	-
SF-295	46	26	76	48	-	-	-	-	-	-
SF-539	67	22	67	21	-	-	-	-	-	12
SNB-19	58	22	72	46	-	-	-	-	-	-
SNB-75	43	22	59	11	19	19	-	17	14	21
U251	45	22	72	58	-	-	-	-	-	-
**Melanoma**										
LOX IMVI	22	-	-	-	-	-	-	-	-	12
MALME-3M	19	20	72	32	-	-	-	-	-	-
M14	12	16	59	30	-	-	--	-	-	-
MDA-MB-435	13	25	71	42	-	-	--	-	-	-
SK-MEL-2	41	20	112	37	-	-	--	-	-	-
SK-MEL-28	-	16	49	30	-	-	-	-	-	-
SK-MEL-5	22	40	102	51	-	13	13	-	-	-
UACC-257	15	44	65	43	-	-	-	-	-	-
UACC-62	44	34	73	45	-	26	25	12	14	-
**Ovarian Cancer**										
IGROV1	16	-	41	16	-	-	-	-	-	-
OVCAR-3	13	44	67	49	-	-	-	-	-	-
OVCAR-4	34	28	75	56	-	-	-	-	-	13
OVCAR-5	12	-	43	17	-	-	-	-	-	-
OVCAR-8	41	25	63	29	-	-	-	-	-	-
NCI/ADR-RES	49	32	76	47	-	-	-	13	-	11
SK-OV-3	18	13	66	46	-	-	-	-	-	-
**Renal Cancer**										
786-0	42	22	66	31	-	-	-	-	-	-
A498	-	-	53	23	-	-	-	-	-	-
ACHN	42	54	83	50	-	-	-	-	-	-
CAKI-1	53	53	77	54	-	16	11	-	-	-
RXF 393	47	27	79	38	-	-	-	-	-	-
SN 12C	37	35	79	47	-	-	-	-	-	-
TK-10	-	-	63	30	-	-	-	-	-	-
UO-31	25	49	83	65	-	23	23	77	23	21
**Prostate Cancer**										
PC-3	65	76	90	79	-	-	19	-	12	-
DU-145	19	18	54	23	-	-	-	-	-	-
**Breast Cancer**										
MCF7	40	52	91	70	-	12	11	-	-	-
MDA-MB-231	40	33	75	42	-	13	-	-	-	-
HS 578T	-	-	-	-	-	-	-	-	-	16
BT-549	31	31	67	24	-	30	-	-	-	-
T-47D	28	41	79	74	-	-	-	-	-	25
MDA-MB-468	52	68	96	82	-	82	-	-	-	-
GI Mean	35	36	74	48	-	-	-	-	-	-

(-) = GI % mean less than 10

Table S3. Validation of docking process by re-docking of the co-crystallized ligands of the three PDB codes (1M17, 2JIV and 4LQM) in the active sites

Co-crystallized ligand	PDB code	RMSD	2D	3D
Erlotinib	1M17	1.25	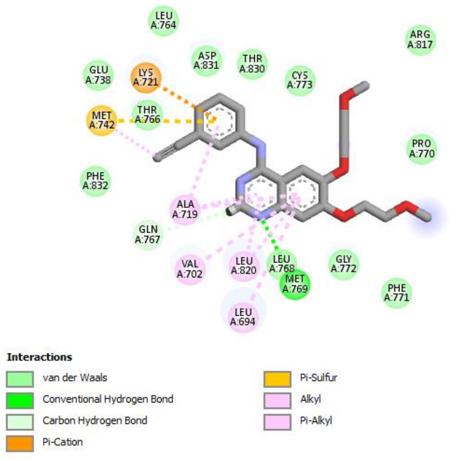	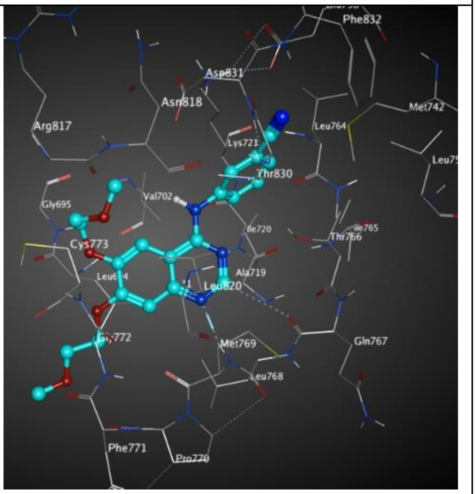
HKI	2JIV	1.83	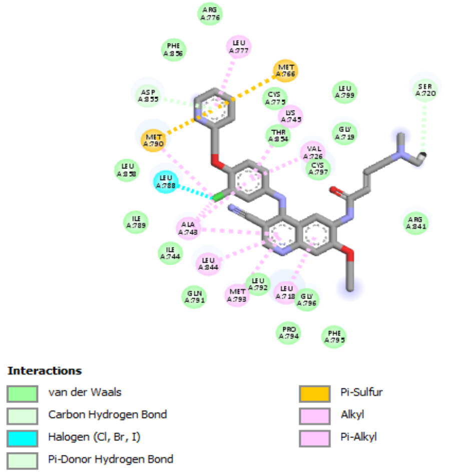	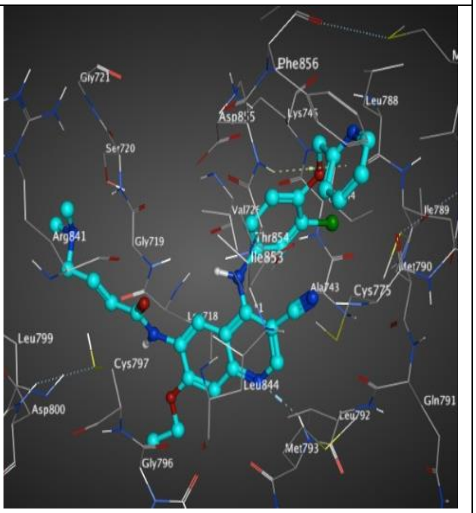
PD-168393	4LQM	1.64	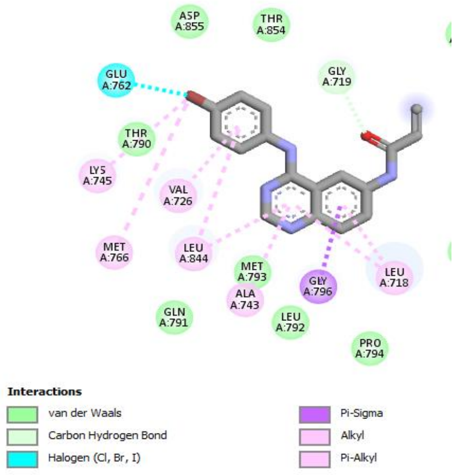	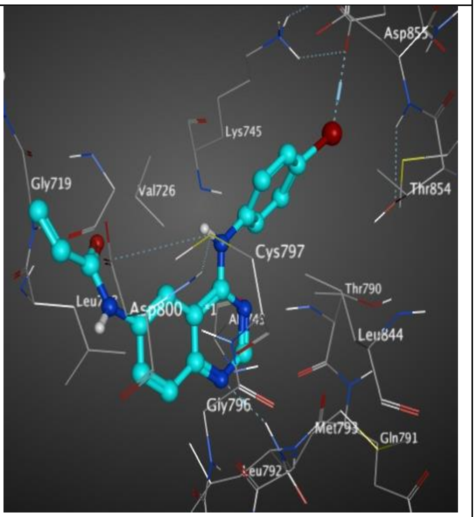

Table S4. 2D and 3D docking poses of erlotinib in the active sites of PDB codes (1M17, 2JIV and 4LQM)

Erlotinib	2D	3D
1M17	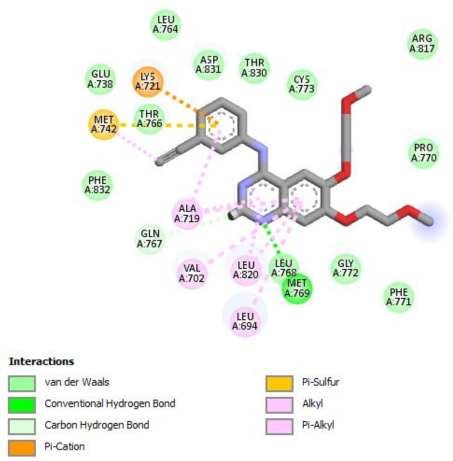	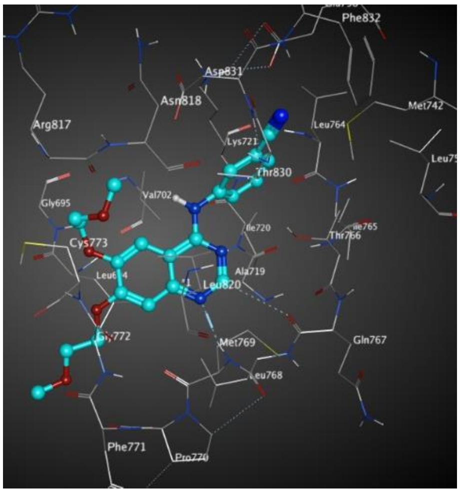
2JIV	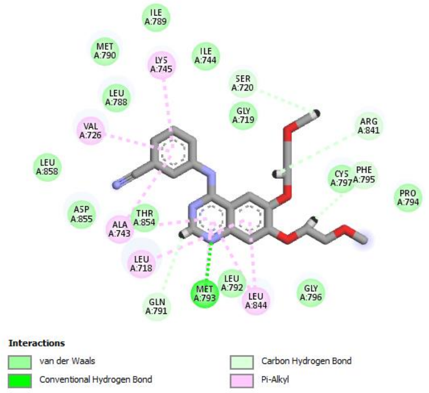	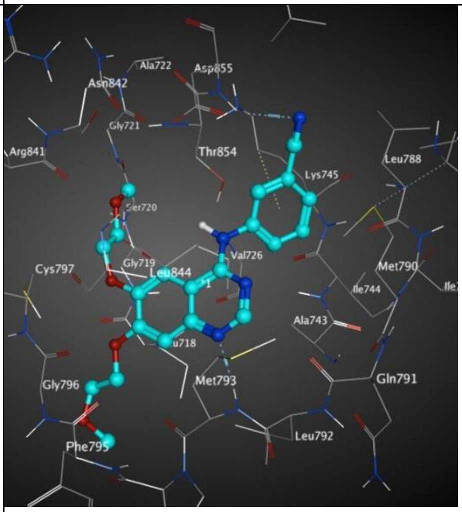
4LQM	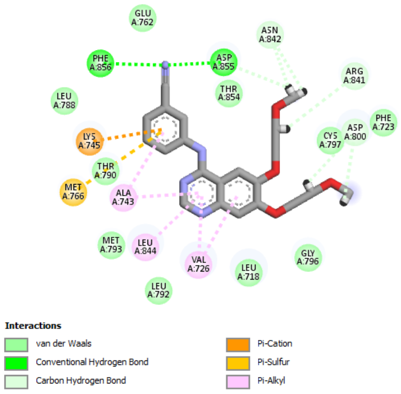	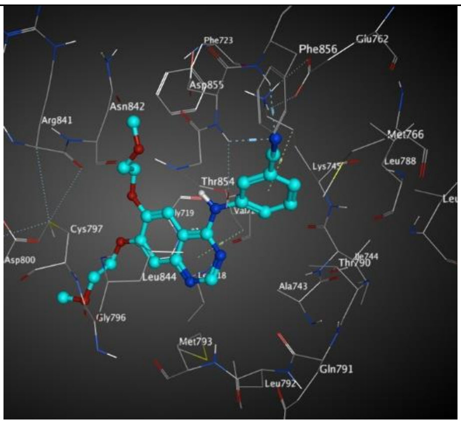

Table S5. Docking scores of *R*-isomer compound **10c** in the active sites of PDB codes (1M17, 2JIV and 4LQM)

Compound	PDB code	Docking score (Kcal/mol)	Amino acids bind with		
			Hydrophobic head	HHQ core	Hydrophobic tail
**10c**	1M17	-5.34	Val702, Lys721, Met742, Asp831	Leu699, eu768, Met769, Leu820	Cys773
**10c**	2JIV	-6.13	Val726, Ala743, Leu788, Met790	Arg841, Asn842, Asp855, Leu844	Cys797
**10c**	4LQM	-6.31	Val726, Arg841, Leu844, Asp855	Phe723	Cys797

Table S6. Docking scores of *R*-isomer compound **10d** in the active sites of PDB codes (1M17, 2JIV and 4LQM)

Compound	PDB code	Docking score (Kcal/mol)	Amino acids bind with		
			Hydrophobic head	HHQ core	Hydrophobic tail
**10d**	1M17	-5.70	Val702, Ala719, Lys721, Leu764, Thr830	Leu694, Phe699, Lys721, Leu820, Asp831	Cys773
**10d**	2JIV	-6.24	Cys797, Arg841, Leu844	Val726, Ala743, Lys745, Cys775 Met790, Met793, Leu844	Lys745, Met766, Leu777, Leu788, Met790
**10d**	4LQM	-6.39	Val726, Ala743, Lys745, Met766, Leu788, Thr854	Leu718, Leu792, Met793, Leu844, Asp855	Cys773

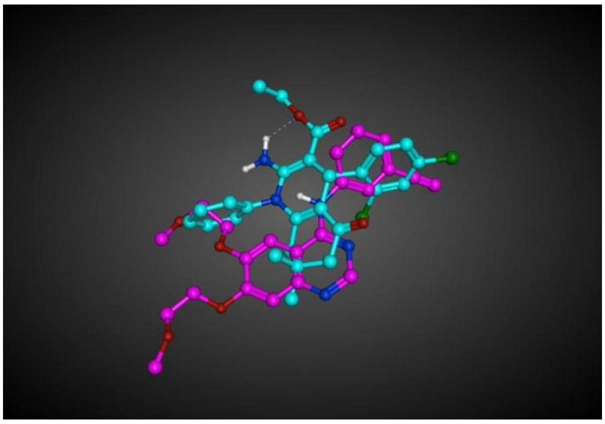

Figure S135. 3D alignment of the *S*-enantiomer of compound **10c** (cyan) with the original ligand (erlotinib) (purple) (PDB code: 1M17).

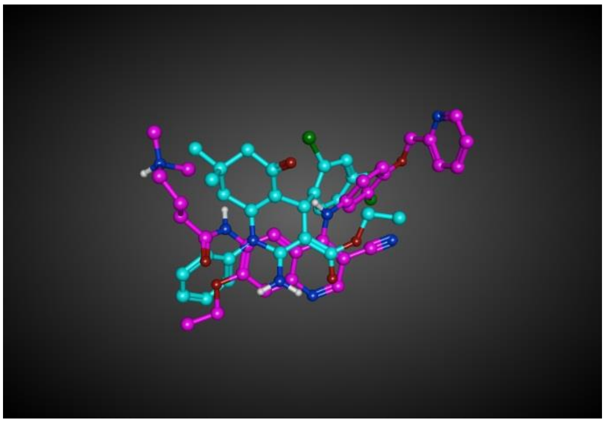

Figure S136. 3D alignment of the *S*-enantiomer of compound **10c (**cyan) with the original ligand (HKI) (purple) (PDB code: 2JIV).

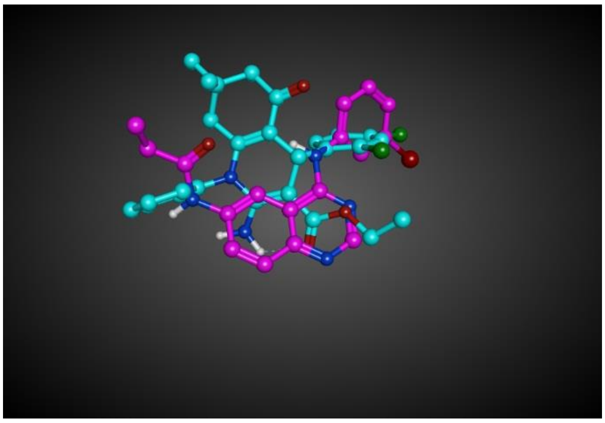

Figure S137. 3D alignment of the *S*-enantiomer of compound **10c (**cyan) with the original ligand (PD-168393**)** (purple) (PDB code: 4LQM).

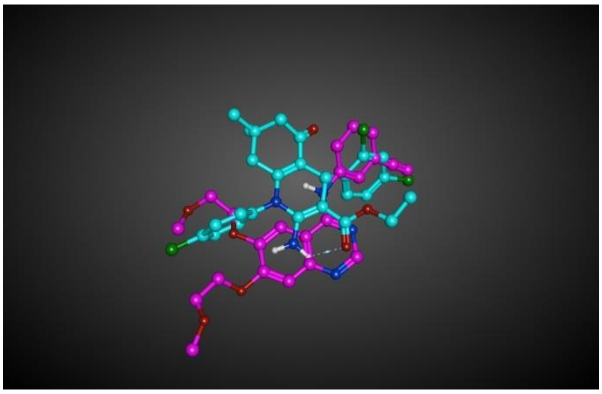

Figure S138. 3D alignment of the *S*-enantiomer of compound **10d** (cyan) with the original ligand (erlotinib) (purple) (PDB code: 1M17).

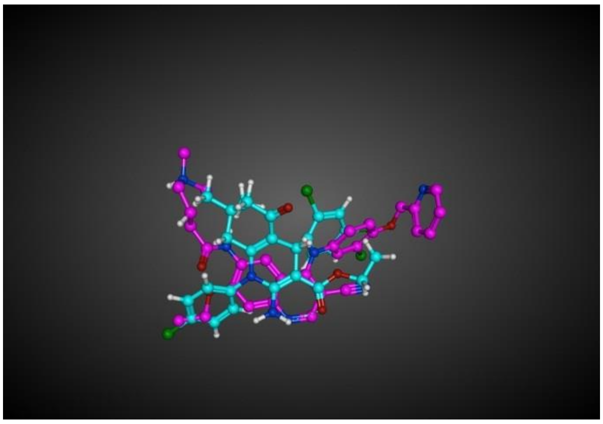

Figure S139. 3D alignment of the *S*-enantiomer of compound **10d** (cyan) with the original ligand (HKI) (purple) (PDB code: 2JIV).

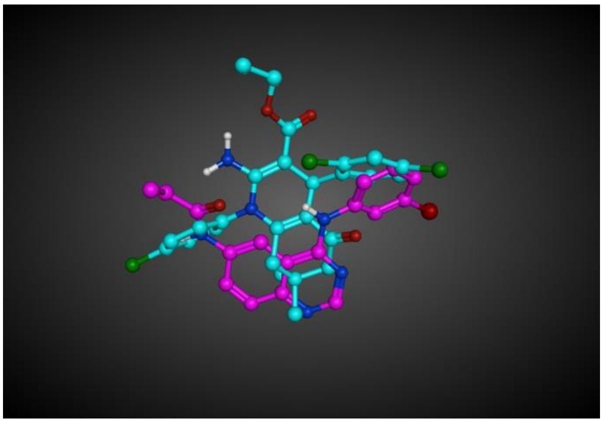

Figure S140. 3D alignment of the *S*-enantiomer of compound **10d** (cyan) with the original ligand (PD-168393**)** (purple) (PDB code: 4LQM).

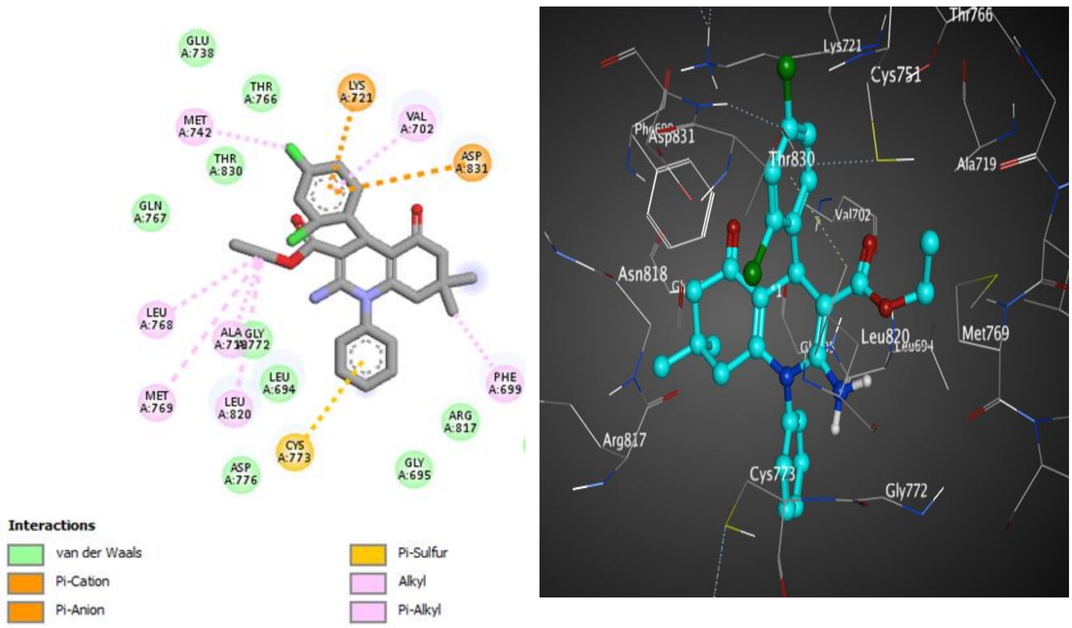

Figure S141. The 2D (left) and 3D (right) pose for docking interactions of *R*-isomer of compound **10c** within the active site of wild type EGFR (PDB code: 1M17).

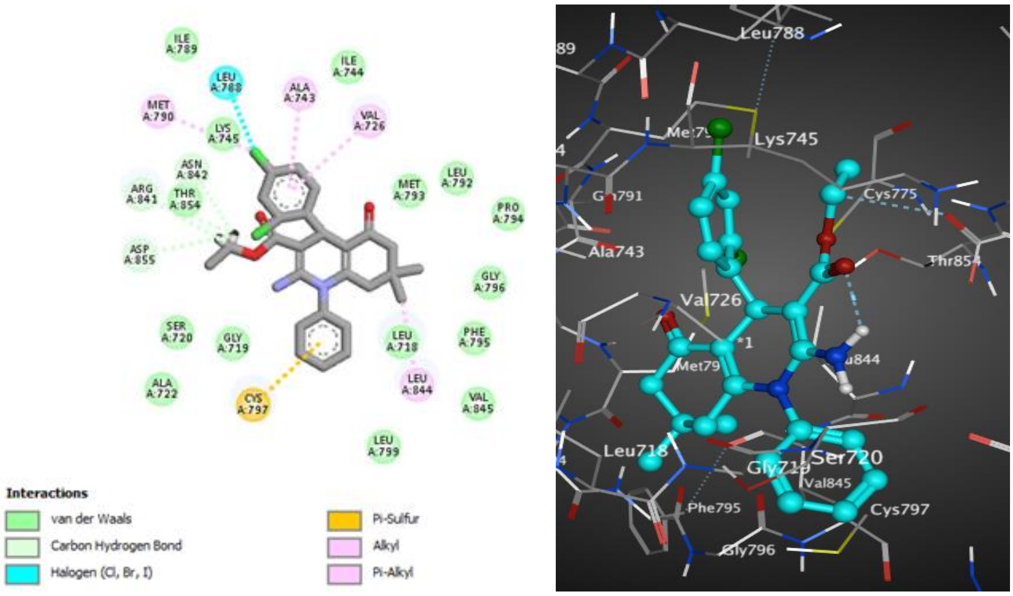

Figure S142. The 2D (left) and 3D (right) pose for docking interactions of *R*-isomer of compound **10c** within the active site of T790M mutant EGFR (PDB code: 2JIV).

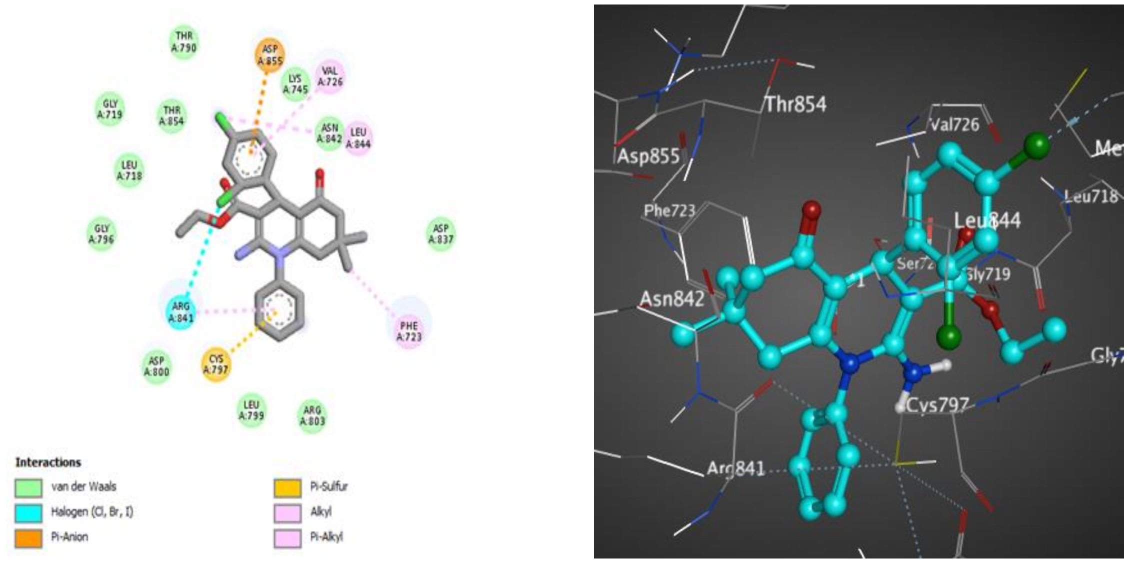

Figure S143. The 2D (left) and 3D (right) pose for docking interactions of *R*-isomer of compound **10c** within the active site of L858R mutant EGFR (PDB code: 4LQM).

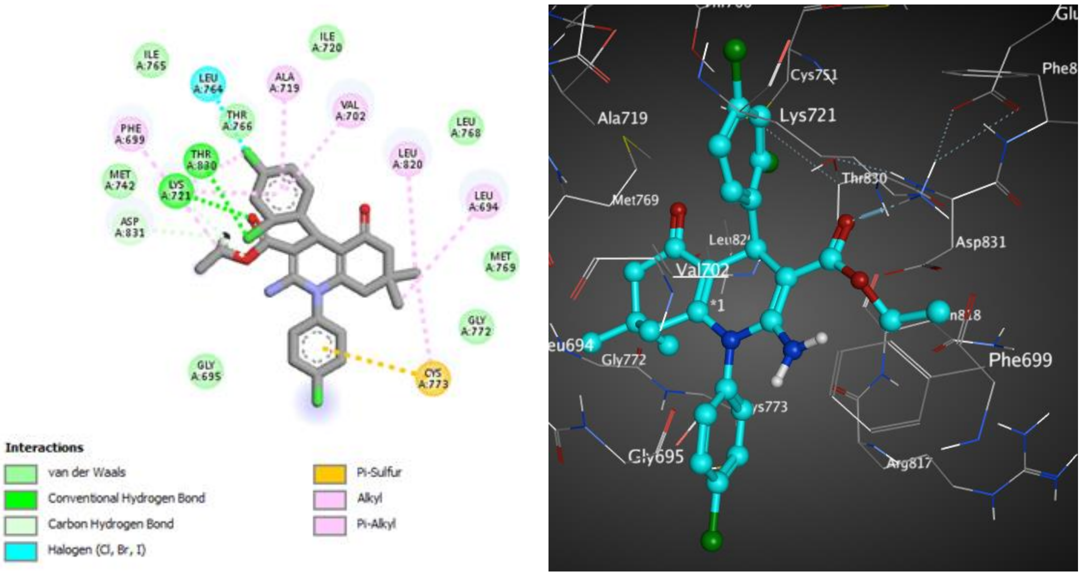

Figure S144. The 2D (left) and 3D (right) pose for docking interactions of *R*-isomer of compound **10d** within the active site of wild type EGFR (PDB code: 1M17).

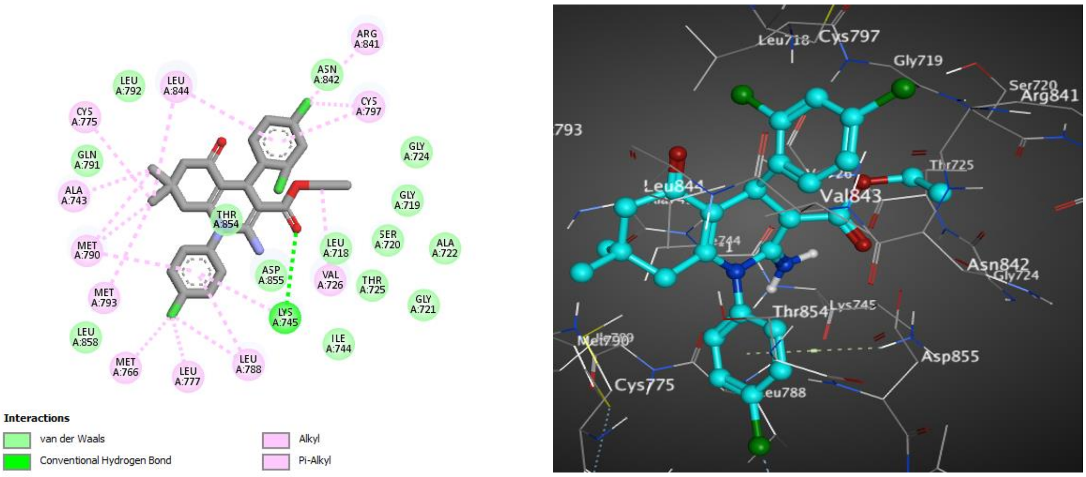

Figure S145. The 2D (left) and 3D (right) pose for docking interactions of *R*-isomer of compound **10d** within the active site of T790M mutant EGFR (PDB code: 2JIV).

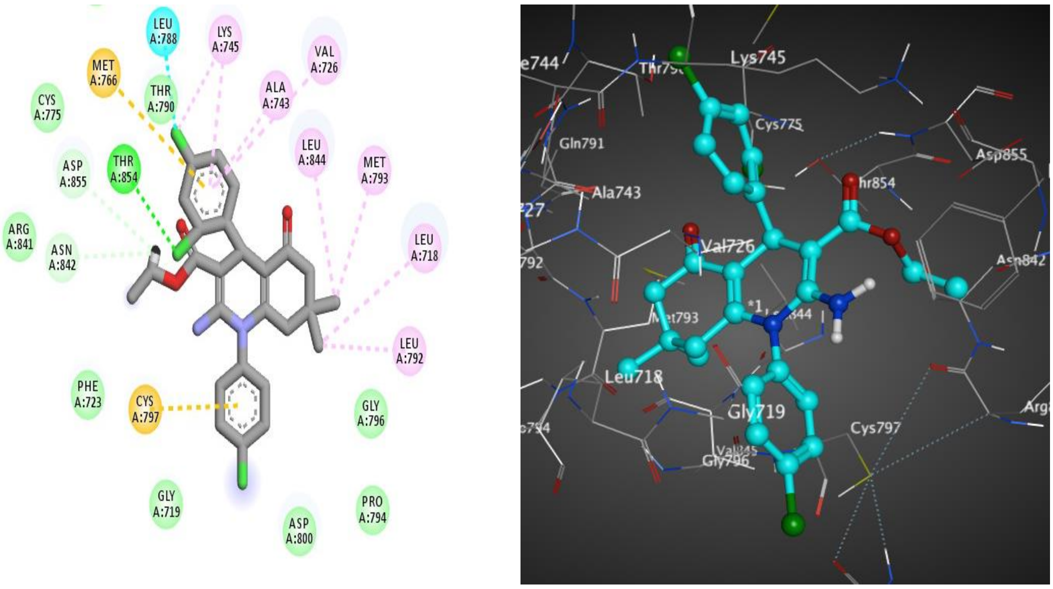

Figure S146. The 2D (left) and 3D (right) pose for docking interactions of *R*-isomer of compound **10d** within the active site of L858R mutant EGFR (PDB code: 4LQM).

X-ray crystallography

A colorless prism crystal of compound **6f** (accession number 2163585) having approximate dimensions of 0.600 x 0.600 x 0.600 mm was mounted on a glass fiber. All measurements were made on a Rigaku R-AXIS RAPID diffractometer using graphite monochromated Mo-Ka radiation. The crystal-to-detector distance was 127.40 mm. Of the 11569 reflections collected, 5449 were unique (R_{int} = 0.0357); equivalent reflections were merged. The linear absorption coefficient, m, for Mo-Ka radiation is 18.595 cm^{-1}. An empirical absorption correction was applied, which resulted in transmission factors ranging from 0.174 to 0.328. The data were corrected for Lorentz and polarization effects. The charge flipping method solved the structure and expanded using Fourier techniques. The final cycle of full-matrix least-squares refinement on F^2 was based on 5449 observed reflections and 298 variable parameters and converged (largest parameter shift was 0.00 times its esd) with unweighted and weighted agreement factors [1, 2].

Biology

- *In vitro* preliminary anticancer activity against 60 NCI cell lines

The cancer screening panel's human tumor cell lines are cultured in RPMI 1640 medium with 5% fetal bovine serum and two mM L-glutamine. Cells are dropped into 96-well microtiter plates in 100 µL with plating densities ranging from 3–8 × 10^3 cells per well [3]. The plates are then incubated for 48 hours after drug addition at 37 °C, 5% CO_2, and 95% air. The experiment is completed for adhering cells by adding cold TCA that fixes the cells in place, and they are then incubated for 60 min at 4 °C. Each well receives 100 µL of 0.4% (w/v) sulforhodamine B (SRB) solution in 1% acetic acid [4]. At 25 °C, plates are incubated for 10 minutes. The absorbance is measured on an automated reader operating at 515 nm. The percentage growth of the treated cells compared to the untreated control cells is computed using the seven absorbance measurements.

- Enzyme inhibition assay of $EGFR^{WT}$, $EGFR^{T790M}$, and $EGFR^{L858R}$

$EGFR^{WT}$, $EGFR^{T790M}$ and $EGFR^{L858R}$ kinase inhibitory assay was performed for the target compounds **6a, 6c, 6d, 6e, 6f, 6i, 8b, 8c, 8e, 10a, 10b, 10c** and **10d** with erlotinib as a reference inhibitor. This test utilized the homogeneous time-resolved fluorescence (HTRF) assay with the $EGFR^{WT}$, $EGFR^{T790M,}$ and $EGFR^{L858R}$ mutations. First, the investigated substances were incubated with $EGFR^{WT}$, $EGFR^{T790M,}$ and $EGFR^{L858R}$ and their substrates for 5 min in an enzymatic buffer. To start the enzymatic process, 1.65 µM of ATP was added [5]. The assay was carried out at room temperature for 30 minutes. The addition of detection reagents, including EDTA, stopped the process. For each concentration, all assays were performed in triplicate, and the relative inhibition (%) of inhibitors was then calculated compared to the control with no inhibitor. Then the IC_{50} values and their standard deviation (SD) for the tested compounds and the reference drug were determined.

☒ Annexin V-FITC apoptosis assay

A protein known as annexin V-fluorescein isothiocyanate (Annexin V-FITC) binds phosphatidyl serine PS, which can be seen by staining. They used Annexin V-FITC and a propidium iodide counterstain (PI). Apoptosis was first induced by incubating cells in 96-microwell plates at a density of 1.2–1.8104 cells/well with the addition of the test substance (100 µL) for 24 hours. Then, 1-5 × 10^5 cells were centrifuged and resuspended in 500 µL of binding buffer. PI (5 µL) and Annexin V-FITC (5 µL) were added, and the mixture was incubated at room temperature for 5 minutes in the dark. Annexin-V-FITC binding was analyzed using A FACS Calibur flow cytometer (BD Biosciences, San Jose, CA) [6].

☒ Cellular mechanistic analysis

The examination of the cell cycle was done using flow cytometry. The test compound was applied to HOP-92 cells before they were cultured for 24 hours at 37 °C, 5% CO_2. After gathering and centrifuging the cell pellets, they were fixed in 70% (v/v) ethanol under the ice for 15 minutes. Pellets were once more collected and then incubated in a solution of 50 µg/mL propidium iodide (PI) and 0.1 mg/mL RNAse. Cell cycle distributions were computed following analysis using flow cytometry on an FC500 cytometer (Beckman Coulter) following an hour of incubation at room temperature [7, 8].

Docking protocol

The interactions of the newly synthesized targets **10c** and **10d**, illustrating the highest $EGFR^{WT}$, $EGFRT^{790M}$ and $EGFRT^{L858R}$ inhibitory activities, were examined and docked within the active sites of the target enzymes to study their binding modes and orientations using $EGFR^{WT}$ (PDB code: 1M17) [9], $EGFR^{T790M}$ (PDB code: 2JIV) [10] and $EGFR^{L858R}$ (PDB code: 4LQM) [11] using MOE-Dock software version 2020.09. The 2D structures of the newly synthesized **10c** and **10d** were drawn using Chem. Draw. The protonated 3D structure was employed using standard bond lengths and angles. Then, energy minimization was applied, and the MOE file was then saved for the subsequent docking process. The co-crystallized structures of $EGFR^{WT}$, $EGFRT^{790M}$ and $EGFRT^{L858R}$ with their ligands, were downloaded (PDB codes: 1M17, 2JIV and 2JIV, respectively) from the Protein Data Bank. All minimizations were performed using MOE Amber10:EHC force field. The preparation of the enzyme structure was performed for molecular docking using the Protonate 3D protocol with the default options in MOE. The London dG scoring function and Triangle Matcher placement method were used in the docking protocol. First, validation of the docking processes was established by docking the native ligands, followed by docking derivatives **10c** and **10d** within the ATP-binding sites of $EGFR^{WT}$, $EGFRT^{790M}$ and $EGFRT^{L858R}$. Visualization of interactions between ligands and binding sites was accomplished *via* discovery studio visualizer (BIOVIA-2021.DS2021Client) [12, 13].

References

[1]M. Shaldam, H. Tawfik, H. Elmansi, F. Belal, K. Yamaguchi, M. Sugiura, G. Magdy, Synthesis, crystallographic, DNA binding, and molecular docking/dynamic studies of a privileged chalcone-sulfonamide hybrid scaffold as a promising anticancer agent, Journal of Biomolecular Structure and Dynamics 40 (2022) 1-15.

[2]A. Babar, A. Saeed, S. Fatima, M. Bolte, N. Arshad, U. Parveen, T. Hökelek, H.R. El-Seedi, Synthesis, X-ray, DFT, Hirshfeld surface analysis, molecular docking, urease inhibition, antioxidant , cytotoxicity, DNA protection, and DNA binding properties of 5-(tert-butyl)-N-(2, 4-dichlorophenyl)-1 H-1, 2, 4-triazol-3-amine, Structural Chemistry 34 (2023) 1-17.

[3]S.E. Ahmadi, R.M. Shabestari, M. Safa, A straightforward microfluidic-based approach toward optimizing transduction efficiency of HIV-1-derived lentiviral vectors in BCP-ALL cells, Biotechnology Reports 38 (2023) 77-92.

[4]M.H. Nazmy, R.A. Mekheimer, M.E. Shoman, M. Abo-Elsebaa, M. Abd-Elmonem, K.U. Sadek, Controlled microwave-assisted reactions: A facile synthesis of polyfunctionally substituted phthalazines as dual EGFR and PI3K inhibitors in CNS SNB-75 cell line, Bioorganic Chemistry 122 (2022) 105-140.

[5]H.H. Elganzory, F.M. Alminderej, M.N. El-Bayaa, H.M. Awad, E.S. Nossier, W.A .El-Sayed, Design, Synthesis, Anticancer Activity and Molecular Docking of New 1, 2, 3-Triazole-Based Glycosides Bearing 1, 3, 4-Thiadiazolyl, Indolyl and Arylacetamide Scaffolds, Molecules 27 (2022) 60-69.

[6]M.H. Saad, T.F. El-Moselhy, E.-D. S, Nabaweya ,A.B. Mehany, A. Belal, M.A. Abourehab, H.O. Tawfik, M.H. El-Hamamsy, Discovery of new symmetrical and asymmetrical nitrile-containing 1, 4-dihydropyridine derivatives as dual kinases and P-glycoprotein inhibitors: synthesis, in vitro assays, and in silico studies, Journal of Enzyme Inhibition and Medicinal Chemistry 37 (2022) 2489-2511.

[7]J.Y. Lee, H. Yang, D. Kim, K.Z. Kyaw, R. Hu, Y. Fan, S.K. Lee, Antiproliferative Activity of a New Quinazolin-4 (3 H)-One Derivative via Targeting Aurora Kinase A in Non-Small Cell Lung Cancer, Pharmaceuticals 15 (2022) 69-88.

[8]S. Yang, C. Wang, L. Shi, J. Chang, Y. Zhang, J. Meng, W. Liu, J. Zeng, R. Zhang, Y. Shao, Design, synthesis and biological evaluation of novel diarylpyridine derivatives as tubulin polymerisation inhibitors, Journal of Enzyme Inhibition and Medicinal Chemistry 37 (2022) 2755-2764.

[9]A. Musa, S.K. Ihmaid, D.L. Hughes, M.A. Said, H.S. Abulkhair, A.H. El-Ghorab, M.A. Abdelgawad, K. Shalaby, M.E. Shaker, K.S. Alharbi, The anticancer and EGFR-TK/CDK-9 dual inhibitory potentials of new synthetic pyranopyrazole and pyrazolone derivatives: X-ray crystallography, in vitro, and in silico mechanistic investigations, Journal of Biomolecular Structure and Dynamics 41 (2023) 1-15.

[10]R.A. Kardile, A.P. Sarkate, D.K. Lokwani, S.V. Tiwari, R. Azad, S.R. Thopate, Design, synthesis, and biological evaluation of novel quinoline derivatives as small molecule mutant EGFR inhibitors targeting resistance in NSCLC: In vitro screening and ADME predictions, European Journal of Medicinal Chemistry 245 (2023) 1-18.

[11]M.A. Shaheen, A.A. El-Emam, N.S. El-Gohary, Design, synthesis and biological evaluation of new series of hexahydroquinoline and fused quinoline derivatives as potent inhibitors of wild-type EGFR and mutant EGFR (L858R and T790M), Bioorganic Chemistry 105 (2020) 104274.

[12]S.K. Paul, K. Dutta Chowdhury, S.R. Dey, A. Paul, R. Haldar, Exploring the possibility of drug repurposing for cancer therapy targeting human lactate dehydrogenase A: a computational approach, Journal of Biomolecular Structure and Dynamics 40 (2022) 1-10.

[13]Y.I. El-Gazzar, H.R. Ghaiad, A.M. El Kerdawy, R.F. George, H.H. Georgey, K.M. Youssef, H.I. El-Subbagh, New quinazolinone-based derivatives as DHFR/EGFR-TK inhibitors: Synthesis, molecular modeling simulations, and anticancer activity, Archiv der Pharmazie 356 (2023) 1-26.
